# Genome and metabolic network of “*Candidatus* Phaeomarinobacter ectocarpi” Ec32, a new candidate genus of *Alphaproteobacteria* frequently associated with brown algae

**DOI:** 10.3389/fgene.2014.00241

**Published:** 2014-07-25

**Authors:** Simon M. Dittami, Tristan Barbeyron, Catherine Boyen, Jeanne Cambefort, Guillaume Collet, Ludovic Delage, Angélique Gobet, Agnès Groisillier, Catherine Leblanc, Gurvan Michel, Delphine Scornet, Anne Siegel, Javier E. Tapia, Thierry Tonon

**Affiliations:** ^1^Sorbonne Universités, UPMC Univ. Paris 06, UMR 8227, Integrative Biology of Marine Models, Station Biologique de RoscoffRoscoff, France; ^2^CNRS, UMR 8227, Integrative Biology of Marine Models, Station Biologique de RoscoffRoscoff, France; ^3^CNRS, IRISA UMR 6074Rennes, France; ^4^IRISA UMR 6074, Université de Rennes 1Rennes, France; ^5^INRIA, Centre Rennes-Bretagne-Atlantique, Projet DylissRennes, France; ^6^Departamento de Ecología, Facultad de Ciencias Biológicas, Pontificia Universidad Católica de ChileSantiago, Chile

**Keywords:** holobiont, algal-bacterial interactions, genome sequencing, metabolic network, symbiosis, transporters, vitamins, phytohormones

## Abstract

*Rhizobiales* and related orders of *Alphaproteobacteria* comprise several genera of nodule-inducing symbiotic bacteria associated with plant roots. Here we describe the genome and the metabolic network of “*Candidatus* Phaeomarinobacter ectocarpi” Ec32, a member of a new candidate genus closely related to *Rhizobiales* and found in association with cultures of the filamentous brown algal model *Ectocarpus*. The “*Ca.* P. ectocarpi” genome encodes numerous metabolic pathways that may be relevant for this bacterium to interact with algae. Notably, it possesses a large set of glycoside hydrolases and transporters, which may serve to process and assimilate algal metabolites. It also harbors several proteins likely to be involved in the synthesis of algal hormones such as auxins and cytokinins, as well as the vitamins pyridoxine, biotin, and thiamine. As of today, “*Ca*. P. ectocarpi” has not been successfully cultured, and identical 16S rDNA sequences have been found exclusively associated with *Ectocarpus*. However, related sequences (≥97% identity) have also been detected free-living and in a *Fucus vesiculosus* microbiome barcoding project, indicating that the candidate genus “*Phaeomarinobacter*” may comprise several species, which may colonize different niches.

## Introduction

Most eukaryotes are known to live in association with bacteria and have established mutualistic relationships with several of them. The importance of these associations is becoming increasingly evident, especially in well-established benchmark models such as the human gut (Ray, 2012), or plant root systems (Turner et al., 2013). Here, bacteria are known to play roles for instance in nutrient assimilation or in pathogen resistance. Studies of systems of hosts and their symbiotic bacteria, frequently referred to as the holobionts, have provided important functional insights into the biology of these organisms. With respect to macroalgae, the importance of mutualistic relationships between algae and bacterial biofilms is well-established (Bartsch et al., 2008; Wahl et al., 2012), yet studies considering the algal-bacterial holobiont are still rare (Dittami et al., 2014). This is despite the fact that macroalgae, and especially large kelp-forest forming brown algal species, constitute important structural elements of coastal ecosystems. They provide shelter and breeding grounds for fish species and marine invertebrates (Santelices, 2007), but are also of direct economic interest, e.g., as a source of alginates or for the production of biofuels (Wei et al., 2013).

Given the difficulties cultivating and experimenting with large kelp-forming brown algae, and considering the large phylogenetic distance between brown algae and other commonly studied multicellular eukaryotes (including land plants and red- and green algae) of approximately 1.7 billion years (Parfrey et al., 2011), a model organism was selected for this lineage: the small filamentous brown alga *Ectocarpus siliculosus* (Charrier et al., 2008). This species was chosen because it is closely related to the kelp-forming Laminariales, both groups having separated approximately 100 million years ago (Silberfeld et al., 2010), and because it has a small genome, is easy to cultivate in the laboratory, and possesses a short life cycle which makes it suitable for genetic studies (Peters et al., 2004). Today, numerous tools have been established for this model, comprising its complete genome sequence (Cock et al., 2010), genetic maps (Heesch et al., 2010), a mutant collection (Le Bail et al., 2011), transcriptomics (Le Bail et al., 2008; Dittami et al., 2009), and proteomics (Contreras et al., 2008). Yet, as of today, very little knowledge is available about the bacteria associated with this model system. Indeed, the only published data currently available on the influence of bacteria on *Ectocarpus* are studies carried out by M. Pedersén over 40 years ago (Pedersén, 1968, 1969, 1973). They showed that antibiotic-treated *Ectocarpus fasciculatus*, a sister species of *E. siliculosus*, which separated from the latter approximately 19 million years ago (Dittami et al., 2012), exhibited poor growth and abnormal morphology, but that these effects could be reversed by the addition of cytokinins.

Here we address the question of algal-bacterial associations in the brown algal model *Ectocarpus* by analyzing the nearly complete genome of a bacterium that was sequenced together with *E. siliculosus*. We show that this bacterium belongs to a new, mainly marine, genus closely related to *Rhizobiales*—an order comprising numerous soil bacteria that enter mutualistic relationships with plant roots. Despite the fact that we have not been able to culture this bacterium, for which we propose the name “*Candidatus* Phaeomarinobacter ectocarpi,” we found it to be frequently associated with brown algae, and the analysis of its genome, as well as the reconstruction of its metabolic network, enabled us to form several hypotheses about the biology of this organism and the interactions it may have with *Ectocarpus*. This type of knowledge contributes to our fundamental understanding of the functioning of algal-bacterial holobionts, but may also prove useful in the context of the sustainable utilization of algae as a natural resource.

## Materials and methods

### Genome sequence, annotation, and metabolic network reconstruction

The genome sequence of “*Ca.* P. ectocarpi” was obtained in the course of the *E. siliculosus* genome project (Cock et al., 2010). It was assembled together with the algal genome and was available from the download section of the *Ectocarpus* genome portal as sctg_1 (http://bioinformatics.psb.ugent.be/orcae/overview/Ectsi). Sctg_1 was identified as bacterial contaminant based on the lack of introns and its circularity, and removed from the published dataset. To identify possible plasmids belonging to the same genome TBLASTN searches using known plasmid replication initiators were carried out against the complete *E. siliculosus* genome database, but yielded no results. Scgt_1 was oriented according to the DnaA protein, and a first round of automatic annotations was generated using the RAST server (Aziz et al., 2008). These annotations were used for functional comparisons between different bacteria with SEED viewer (Overbeek et al., 2005). The generated GenBank file with the automatic annotations was then used in Pathway Tools version 17.5 (Karp et al., 2010) for metabolic network reconstruction including gap-filling and transporter prediction.

Manual annotation was performed for selected metabolic pathways and gene families. Candidate genes were identified using bi-directional BLASTP searches with characterized protein sequences retrieved from the UniProt database. In addition, we used the transporter classification database (TCDB) as reference for transporters, and the carbohydrate active enzyme (CAZYme) database CAZY (Lombard et al., 2014) as reference for CAZYmes. Finally, candidate sequences were compared to the genome of *Zobellia galactanivorans* Dsij^T^ (accession FP476056), a genome of a marine bacterium for which all protein sequences were subject to expert annotation. All of our manual annotations were incorporated both into the final genome release and the draft metabolic network. The resulting curated metabolic network is available in Pathway Tools via the SRI Registry of Pathway/Genome Databases and on the public Pathway Tools server of the Station Biologique de Roscoff (http://pwt.sb-roscoff.fr/). The manually annotated “*Ca.* P. ectocarpi” genome was deposited at the European Nucleotide Archive (ENA) under the accession number HG966617.

### Comparison and complementarity of “*Ca.* P. ectocarpi” and *E. siliculosus* metabolic networks

In order to identify potential complementarities between the “*Ca.* P. ectocarpi” metabolic network and the metabolic network of the alga it was sequenced with, the following analyses were carried out. For *E. siliculosus*, an SBML file of its metabolic network was downloaded from the EctoGEM website (http://ectogem.irisa.fr/; Prigent et al. pers. com.). In the context of this study, we chose EctoGEM-*combined*, a version of EctoGEM without functional gap-filling, which we will refer to as the “non-gap filled algal network.” This was important for our analysis as we aimed to identify possible gaps in EctoGEM that may be filled by reactions carried out by the bacterium. An SBML version of the “*Ca.* P. ectocarpi” metabolic network was then extracted from Pathway Tools and merged with the non-gap filled algal network using MeMerge (http://mobyle.biotempo.univ-nantes.fr/cgi-bin/portal.py#forms::memerge). In the context of this study, we refer to this merged network as the “holobiont network.” Following the procedure outlined on the EctoGEM website, we used Meneco 1.4.1 (https://pypi.python.org/pypi/meneco) to test the capacity of the holobiont network to produce 50 target metabolites that have previously been observed in xenic *E. siliculosus* cultures (Gravot et al., 2010; Dittami et al., 2011) from the nutrients found in the Provasoli culture medium as source metabolites. The exact list of target and source metabolites is available from the EctoGEM website. Results obtained for the holobiont network were also compared to EctoGEM 1.0, the gap-filled and manually curated version of the *E. siliculosus* network, which we refer to as the “manually curated algal network” in this study.

### Taxonomic position and distribution of “*Ca.* P. ectocarpi”

Phylogenetic analyses with the predicted “*Ca.* P. ectocarpi” 16S rDNA sequence were carried out with selected representative sequences of known orders of *Alphaproteobacteria*. Sequences were aligned using MAFFT (Katoh et al., 2002), and conserved positions manually selected in Jalview 2.8 (Waterhouse et al., 2009). The final alignment comprised 236 sequences with 1243 nucleotide positions. Neighbor-joining (NJ) analyses were carried out using MEGA5 (Tamura et al., 2011), the Kimura 2-parameter substitution model, and 500 bootstrap replicates. They were complemented by maximum likelihood (ML) analyses using RAXML 7.4.2 (Stamatakis, 2006) and the GTR+G+I substitution model, which was estimated to be the most suitable for ML analyses of our dataset using MEGA5. ML analyses were carried out with 100 bootstrap replicates. A second alignment comprising an extended set of 790 sequences was also generated and used in parallel. Results for this latter analysis and all sequence accessions are available in Data sheet 1. The tree topology obtained was compared with results from RDP-classifier (Wang et al., 2007). To explore the distribution of “*Ca*. Phaeomarinobacter,” related sequences were searched for via BLAST in the NCBI nr, 16S rDNA, and EnvDB databases, in the megx.net databases version r6 (Kottmann et al., 2010), in the Global Ocean Survey database (Parthasarathy et al., 2007), and in selected marine metagenome and metabarcoding experiments deposited in the NCBI and ENA short read archives.

### Attempts to culture “*Ca.* P. ectocarpi”

Several unsuccessful attempts were made to isolate and cultivate “*Ca.* P. ectocarpi” after the discovery of the bacterial genome. These experiments were carried out with the same antibiotic-treated culture of *E. siliculosus* strain Ec32 (CCAP accession 1310/4, isolated from San Juan de Marcona, Peru) also used for the sequencing of the *E. siliculosus* genome (Cock et al., 2010). This culture had been treated with 720 μg/mL penicillin, 360 μg/mL streptomycin, and 72 μg/mL chloramphenicol for at least 2 weeks, before it was transferred to autoclaved natural seawater and treated once more with 100 μg/mL cefotaxime, 180 μg/mL penicillin, 90 μg/mL streptomycine, and 18 μg/mL chloramphenicol. Finally, the culture was used to produce algal biomass in Provasoli-enriched (Starr and Zeikus, 1993) and autoclaved natural seawater with added 180 μg/mL penicillin, 90 μg/mL streptomycin, 18 μg/mL chloramphenicol. Prior to DNA extraction, samples of the culture were transferred to agar plates (autoclaved seawater with added Provasoli-nutrients, 0.1% sucrose, 1.5% agar) and no bacterial growth was detected after incubation of these plates at room temperature for several weeks. As shown by the sequencing of the nearly complete genome of “*Ca*. P. ectocarpi” along with the genome of *E. siliculosus*, the former bacterium was still present in the algal cultures at this time and constituted the only major bacterial contaminant. The antibiotic-treated cultures were then once more transferred to autoclaved Provasoli-enriched seawater without added antibiotics and used in the attempt to isolate “*Ca.* P. ectocarpi” according to the procedure described below.

Ground algal cultures were transferred to approximately 5 ml of liquid Zobell medium (Zobell, 1941) and, after 1 week at room temperature, aliquots of the medium were plated on Zobell agar plates. After 4 weeks, the ground *E. siliculosus* culture in Zobell medium was plated once more on both Zobell and M13 (Schlesner, 1989) agar plates (again at room temperature). In a parallel attempt, non-ground filaments from the same antibiotic-treated cultures were used to directly inoculate 5 ml aliquots of liquid Zobell and modified YEB medium (5 g/L peptone, 1 g/L yeast extract, 5 g/L sucrose, 0.24 g/L MgSO_4_, in filtered autoclaved seawater, pH 7.2), which were incubated at 20°C under continuous shaking for several weeks. However, none of these attempts resulted in the isolation of bacterial cultures.

## Results

### “*Ca.* phaeomarinobacter”—a candidate genus closely related to *rhizobiales* frequently found in association with brown algae

So far, full-length 16S rDNA sequences with 100% identity to “*Ca.* P. ectocarpi” were found exclusively in the antibiotics treated cultures used for the sequencing of the *E. siliculosus* genome (see section Attempts to Culture “*Ca.* P. ectocarpi”). However, closely related sequences likely to belong to the same genus, i.e., sequences exhibiting 97–99% identity (Stackebrandt and Gobel, 1994) were found in selected marine samples (Table 1). Notably, a nearly complete 16S rDNA sequence with 99.3% identity to that of “*Ca.* P. ectocarpi” was identified on oil slicks at the surface of the Gulf of Mexico (Redmond and Valentine, 2012). Another two bacteria featuring 97.5 and 94.7% 16S rDNA sequence identity, GMD21A06 and GMD21D06, were isolated from the Sargasso Sea and cultivated in microdroplets (Zengler et al., 2002). BLAST searches carried out against the NCBI 16S rDNA sequence database yielded [*Rhodopseudomonas*] *julia* KR-11-67^T^ (DSM 11549; AB087720) as best hit. The 16S rDNA sequence of the strain KR-11-67^T^ indicates that this strain belongs to the genus *Rhodobium*. Using the EzTaxon database, the first hit for the 16S rDNA sequence of “*Ca*. P. ectocarpi” Ec32 was the unclassified *Rhizobiales Parvibaculum indicum* P31^T^ (FJ182044; Lai et al., 2011) with 92% identity. BLAST searches against several metagenome and metabarcoding databases (Table 1) did not reveal “*Ca.* Phaeomarinobacter”-like sequences in datasets for large kelp species, but in one data set for *Ectocarpus* and one for *Fucus*, respectively. The *Ectocarpus* data set corresponds to Illumina 16S rDNA metabarcoding experiments of bacteria present in 20 different algal cultures, and amplicons with 100% identity to that of “*Ca*. P. ectocarpi” were detected in five cultures from different locations. The *Fucus* data set corresponds to a metabarcoding experiment based on 454-sequencing. Here samples were collected from the Kiel bight (Baltic Sea), and “*Ca. Phaeomarinobacter*”-like sequences were detected in 8 of 78 samples. Interestingly, in the *Fucus* data set, two different sequences were present: a more abundant sequence with 99% identity to the 16S rDNA sequence of “*Ca* P. ectocarpi,” and a second sequence with only 97% identity, suggesting the existence of different species associated with algae within this candidate genus.

**Table 1:** **Occurrence of “*Ca.* Phaeomarinobacter”-related 16S rDNA sequences (≥97% identity) in public genomic and metagenomic samples**.

**Origin**	**Sample type**	**Identity**	**Accession**
San Juan de Marcona, Peru^*^	*Ectocarpus* sp. culture	100% (1467 bp)	ENA: HG966617^*^
Port Aransas, USA	*Ectocarpus* sp. culture	100% (404 bp)	ENA: PRJEB5542
Kingsbridge, UK	*Ectocarpus* sp. culture	100% (404 bp)	ENA: PRJEB5542
Terenez, France	*Ectocarpus* sp. culture	100% (404 bp)	ENA: PRJEB5542
Hopkins Rive Falls, Australia	*Ectocarpus* sp. culture	100% (404 bp)	ENA: PRJEB5542
Kiel Bight, Germany	*Fucus vesiculosus* surface	99% (318 bp)	SRA: SRP015929
Gulf of Mexico	Surface oil slicks	99% (1322 bp)	JN018674
Sargasso Sea	Bacterioplankton	98% (1326 bp)	AY162106
Kiel Bight, Germany	*Fucus vesiculosus* surface	97% (318 bp)	SRA: SRP015929

* Indicates the genome sequence analyzed here.

**Figure 1:**
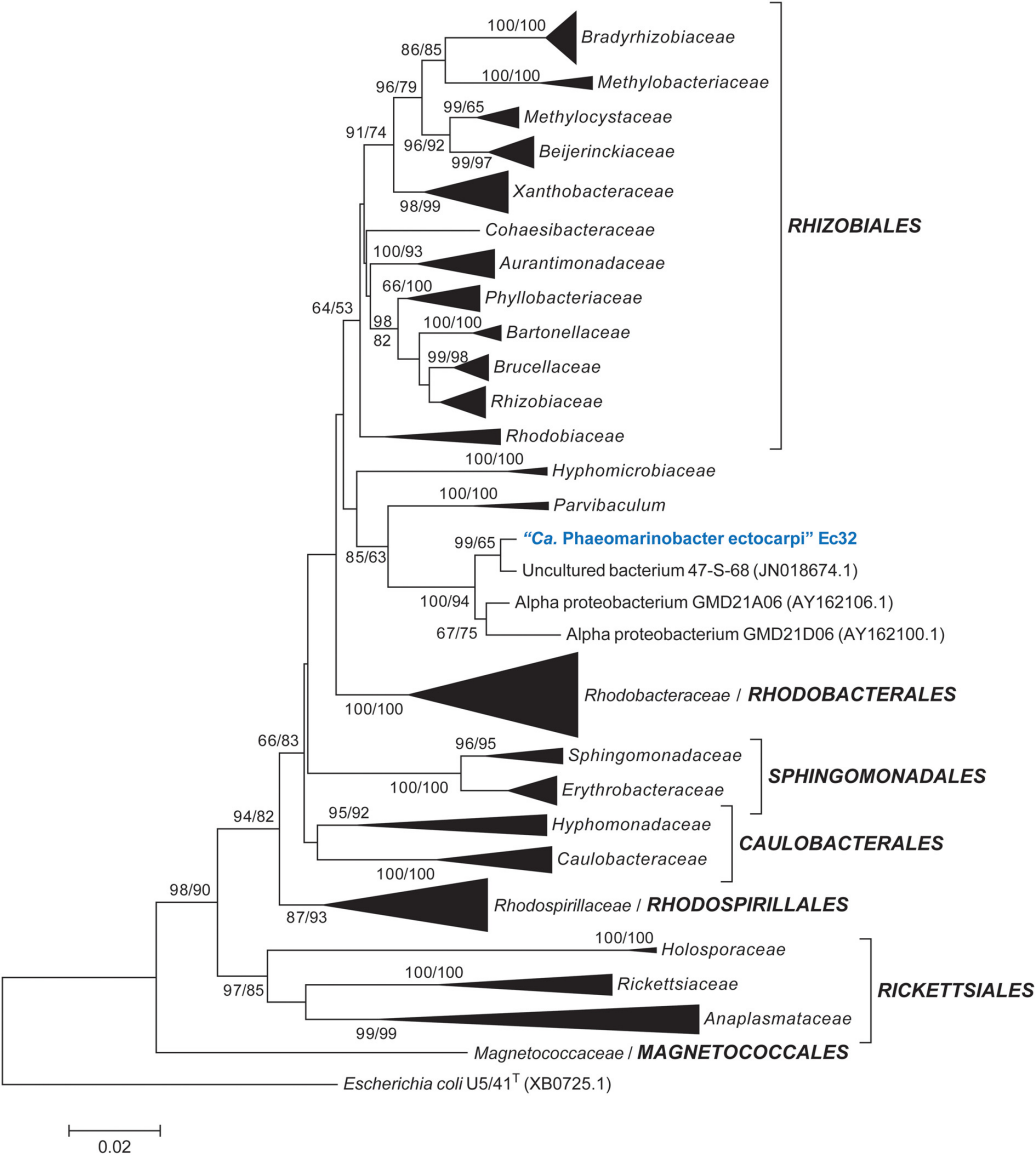
**Taxonomic position of “*Ca.* Phaeomarinobacter ectocarpi” Ec32 within the *Alphaproteobacteria*.** The figure shows a neighbor-joining tree of 236 16S rDNA sequences with bootstrap support values obtained for this and a corresponding maximum likelihood tree, respectively (only values ≥50% are shown). Hyper-variable regions were masked from the alignment. The *Gammaproteobacterium Escherichia coli* was used as outgroup. A more exhaustive tree of *Alphaproteobacteria* based on 790 taxa is available in Data sheet 1.

Altogether, these BLAST analyses indicate that “*Ca.* P. ectocarpi” belongs to the class *Alphaproteobacteria*. To determine the exact taxonomic position of “*Ca*. P. ectocarpi” within the *Alphaproteobacteria*, two phylogenetic analyses were performed: one with a representative sample of 236 full-length 16S rDNA sequences comprising all orders of the class, and a second, extended analysis, comprising all available families. In the resulting phylogenetic trees, “*Ca*. P. ectocarpi” was located in a well-supported clade composed of sequences from the uncultured bacterial clone 47-S-68 and of the *Alphaproteobacteria* GMD21A06 and GMD21D06 (Figure 1). It was linked to the species *Parvibaculum* via a node with moderate support (85 and 63% in NJ and ML analyses respectively) in the reduced phylogenetic tree (Figure 1) but not in the complete tree (Data sheet 1). Given that the genus *Parvibaculum* is currently classified as *Rhizobiales*, and in agreement with the automatic classification obtained via RDP classifier, we could assume that “*Ca.* Phaeomarinobacteraceae” also belongs to the order of *Rhizobiales*. However, as seen from the phylogenetic tree presented by Gruber-Vodicka et al. (2011), and the lack of bootstrap support for an expanded order of *Rhizobiales* (including *Parvibaculum*) in our analyses (Figure 1, Data sheet 1), we can conclude that the clade including “*Ca*. P. ectocarpi” and its relatives likely represents a new order. In any case it represents a new family, “*Ca.* Phaeomarinobacteraceae” fam. nov., including “*Ca.* Phaeomarinobacter spp.” with species “*Ca.* Phaeomarinobacter ectocarpi,” and the strains “*Ca.* Phaeomarinobacter sp.” GMD21A06 and GMD21D06.

### A compact and functional genome without the characteristics of nodule-forming *rhizobiales*

**Figure 2:**
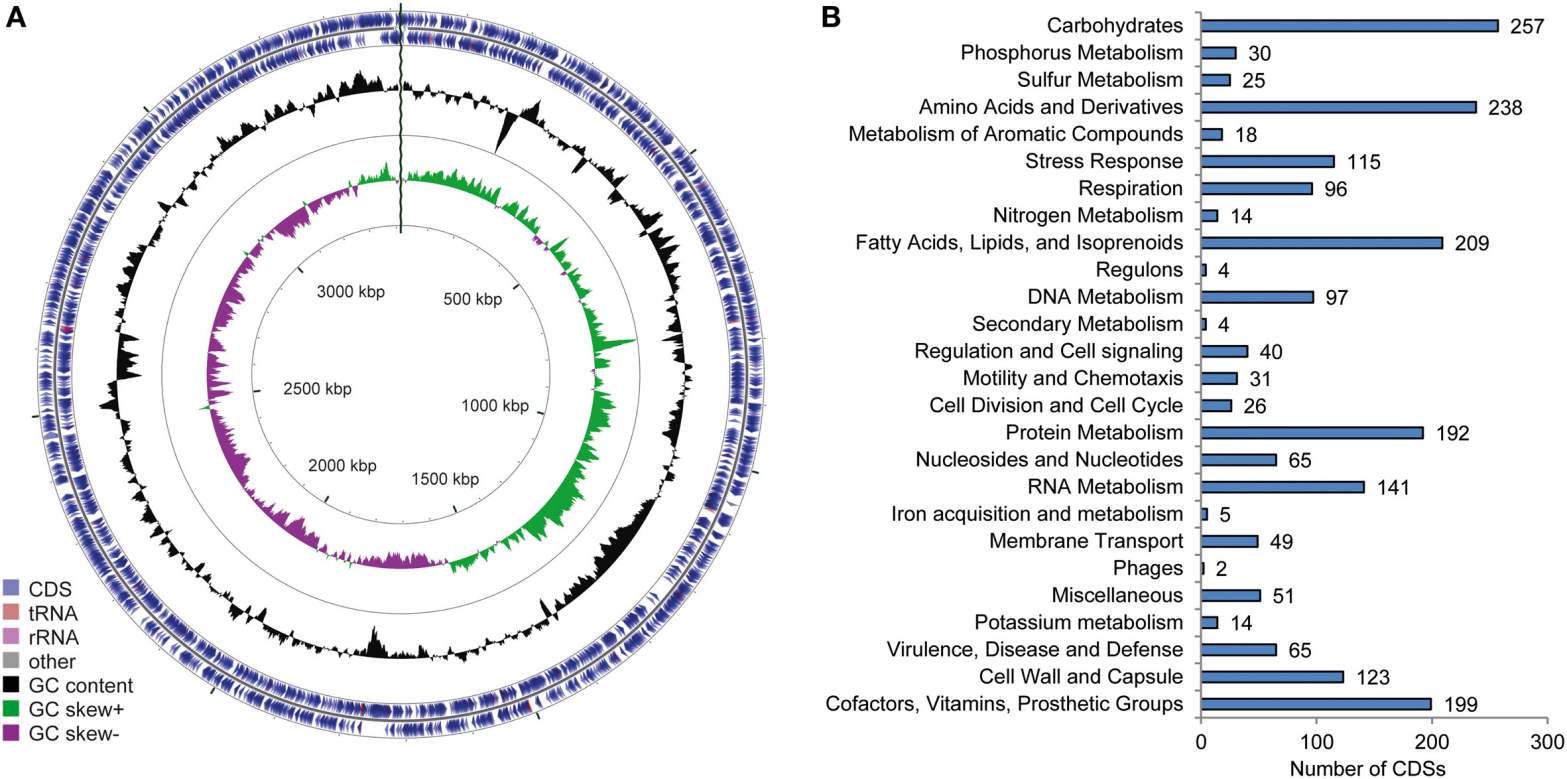
**Overview of the “*Ca.* Phaeomarinobacter ectocarpi” Ec32 genome. (A)** illustration of the genome structure generated using CGView (Stothard and Wishart, 2005); **(B)** summary of subsystems identified using RAST (Aziz et al., 2008).

The circular genome of “*Ca*. P. ectocarpi” has a total size of 3.4 Mbp and contains 3298 predicted open reading frames (Table 2, Figure 2). No plasmid replication initiator sequences were found in the *E. siliculosus* genome data, providing a loose indication of the absence of functional plasmids in the bacterium. At the time of submission, the metabolic network of “*Ca.* P. ectocarpi” comprised 1558 enzymatic reactions organized in 279 pathways with a rather complete set of genes and pathways related to primary metabolism. They include the TCA cycle (PWY-5913, PWY-6969), glycolysis (GLYCOLYSIS), the pentose phosphate pathway (NONOXIPENT-PWY, P21-PWY), purine and pyrimidine de novo synthesis (PWY-7227, PWY-7226, PWY-7184), fatty acid biosynthesis (PWY-4381, PWY-5971, PWY-6282) including cyclopropane fatty acids (PWY0-541) and fatty acid elongation (FASYN-ELONG-PWY), and the synthesis of all major amino acids (IND-AMINO-ACID-SYN). Details on each of these pathways can be found at http://pwt.sb-roscoff.fr/.

**Table 2:** **Key statistics of the “*Ca.* Phaeomarinobacter ectocarpi” Ec32 genome and of the reconstruction of its metabolic network**.

**Features**	**Value**
Assembly size (bp)	3,415,905
G+C content	59%
No. of protein-coding sequences	3298
Average CDS length (bp)	919
ORFs manually annotated	402
No. of predicted tRNA genes	40
No. of predicted rRNA operons	1
No. of predicted metabolic pathways	279
No. of predicted enzymes	1161
No. of predicted enzymatic reactions	1558
No. of predicted transporters	217
No. of predicted transport reactions	77

According to the RAST SEED viewer, the closest available genome to “*Ca*. P. ectocarpi” is that of *Parvibaculum lavamentivorans* DS-1^T^ (91% identity in the 16S rDNA sequence), a bacterium isolated from a sewage treatment plant and of economic interest due to its capacity to degrade linear alkylbenzenesulfonate (Schleheck et al., 2011). A functional comparison of the *P. lavamentivorans* and “*Ca*. P. ectocarpi” genomes showed that both share 1523 predicted functions, while 322 are specific to *P. lavamentivorans* and 142 are specific to “*Ca.* P. ectocarpi.” Among the functions encoded specifically by “*Ca.* P. ectocarpi” we found genes involved in potassium homeostasis and bacterial chemotaxis, while, unlike in *P. lavamentivorans*, we found no genes encoding gene transfer agents (see Data sheet 2).

Considering that “*Ca*. P. ectocarpi” is closely related to *Rhizobiales*, which comprise root nodule forming species, we searched for genomic features specifically related to nodule formation, notably NOD, NIF, and FIX genes. Only three putative operons containing a few of these genes were detected: one comprising NifR, NodV, as well as three nitrogen regulation proteins (Phect2856-Phect2861); one comprising fixNOQP (Phect807-804); and one comprising fixGHIS (Phect803-800). While deletions at least in the latter two operons lead to defective symbiotic nitrogen fixation in nodule-forming *Rhizobiales* (Preisig et al., 1996), these operons may also be present in free-living *Rhizobiales* as is the case, e.g., in *P. lavamentivorans*. Furthermore, no symbiotic islands were found, i.e., large clusters of symbiosis-related genes typical for nodule-forming *Rhizobiales* in absence of plasmids (MacLean et al., 2007).

### Automatic analysis of “*Ca.* P. ectocarpi” and *E. siliculosus* metabolic networks does not reveal clear evidence of complementarity

To identify possible complementarities between the metabolism of “*Ca*. P. ectocarpi” and that of the alga it was sequenced with, we merged the “*Ca*. P. ectocarpi” metabolic network with a non-gap filled network of *E. siliculosus*. This holobiont network was then compared to the non-gap filled algal network with respect to its capacity to produce metabolites previously detected in *E. siliculosus* cultures. While the non-gap filled algal network alone was able to produce 25 of the 50 detected compounds, the holobiont network was able to produce 33 compounds, i.e., 8 additional compounds. These additional compounds were palmitoleic acid (CPD-9245), cis-vaccenate (CPD-9247), D-glycerate (GLYCERATE), glycolate (GLYCOLLATE), L-histidine (HIS), palmitate (PALMITATE), stearate (STEARIC_ACID), and L-tyrosine (TYR). The biosynthetic pathways for all of these 8 metabolites were manually examined in both the bacterium and the alga.

With respect to the production of the fatty acids palmitoleic acid, cis-vaccenate, palmitate, and stearate, the “*Ca*. P. ectocarpi” network comprised the reaction EC 2.3.1.180 catalyzed by Phect3123 and Phect2285, which was missing in the non-gap filled algal network. However, we were able to manually identify Esi0069_0107 as a good candidate gene with this activity in the alga. “*Ca.* P. ectocarpi” is furthermore able to produce glycerate via the reaction EC 1.1.1.81, but a gene encoding a 3-phospho-D-glycerate phosphatase had been added to the manually curated algal network, and could account for the production of this metabolite by *E. siliculosus*. Finally, the bacterial metabolic network contains the tyrosine biosynthesis I pathway (TYRSYN), but the manual annotation of genes involved in the tyrosine biosynthesis II pathway (PWY-3461) in the alga allowed completing this alternative pathway in the manually curated algal network (Prigent et al. pers. com.). These data thus suggest that at least 6 of the 8 compounds that became producible by merging the algal and bacterial networks could also be synthesized by the alga without the bacterium.

For the remaining 2 compounds that became producible in the holobiont network compared to the non-gap filled algal network, possible candidate genes in *E. siliculosus* were found, but assigning an exact function to these genes was difficult based on sequence homology. This was the case for glycolate, which can be produced by “*Ca*. P. ectocarpi” from glyoxylate via the activity of the protein encoded by Phect1668. In *E. siliculosus* a potential candidate gene for this reaction could be Esi0002_0012, but well-characterized stramenopile glyoxylate reductases are not available to confirm this hypothesis. The situation is similar for L-histidine. Here the *E. siliculosus* genome is missing a histidinol phosphate phosphatase present in “*Ca*. P. ectocarpi” (Phect785), but the specificity of phosphatases based on sequence homology is difficult to deduce, and the *E. siliculosus* genome encodes several unknown phosphatases. Thus, although metabolic interactions between *E. siliculosus* and “*Ca*. P. ectocarpi” cannot be excluded for the production of these compounds, our analysis did not provide clear indications supporting a bacterial role in the production of the 50 target metabolites considered.

### A wide array of transporters for uptake and excretion of nutrients and metabolites

A total of 217 predicted membrane transporters were identified (Data sheet 3), and divided into three categories according to their structure and function: pumps (primary active transporters), channels, and secondary transporters.

Primary active transporters in “*Ca*. P. ectocarpi” comprise mainly ABC transporters (73 proteins). ABC proteins depend on ATP to transport various substances (e.g., ions, peptides, nucleosides, amino acids, carbohydrates, and proteins). In “*Ca*. P. ectocarpi,” the genes encoding several ABC transporters are organized in clusters. For example, the cluster Phect395-Phect399 is related to a cobalamin (vitamin B12) import system. It is composed of the ABC transporter complex BtuCDF (Phect396-Phect398), an ATP:Cob(I)alamin adenosyltransferase (EC2.5.1.17, Phect395), and a cobalamin-specific TonB-dependent receptor (BtuB, Phect 399) (Butzin et al., 2013). In the same way, the cluster Phect1895-Phect1901 is related to a phosphate transport system (PST). It is composed of an ATP-binding protein (Phect1899, PstB), two transmembrane proteins (Phect1897, PstC; and Phect1898, PstA), and a solute-binding protein (Phect1895, PstS). The cluster also comprises a phosphate-specific transport system accessory protein PhoU (Phect1900), and a phosphate regulon transcriptional regulatory protein PhoB (Phect1901). Among the primary active transporters, we found a proton translocating NADH:quinone oxidoreductase complex I in charge of energy transduction. This complex is composed of only the 14 central subunits of respiratory chain complexes and is organized in a cluster (Phect2755-Phect2768). However, these subunits are sufficient to perform all bioenergetic functions (Bogachev and Verkhovsky, 2005).

With respect to secondary transporters, the major facilitator superfamily (MFS) and resistance-nodulation-cell division (RND) superfamily are the most represented (21 and 20 proteins, respectively). The MFS is a large and diverse group of secondary transporters including uniporters, symporters, and antiporters. MFS proteins facilitate the transport across cytoplasmic or internal membranes of a variety of substrates including ions, sugar-phosphates, drugs, neurotransmitters, nucleosides, amino acids, and peptides. In the “*Ca.* P. ectocarpi” genome, six loci encode proteins related to sugar transport (Phect208, Phect229, Phect230, Phect372, Phect2869, and Phect3041), one locus is related to multidrug efflux (Phect272), one locus encodes a probable 3-phenylpropionic acid transporter (Phect372), and 14 loci are uncharacterized (Phect111, Phect135, Phect930, Phect931, Phect976, Phect1451, Phect1452, Phect1453, Phect1462, Phect2092, Phect2417, Phect2418, Phect3215, and Phect3222).

Finally, we identified a total of 30 channel/pore proteins in “*Ca*. P. ectocarpi.” Transport systems of this type facilitate diffusion through transmembrane aqueous pores or channels by an energy-independent process, without evidence for a carrier-mediated mechanism. For example, Phect1483, Phect2180, Phect2444, and Phect2910 encode small-conductance mechanosensitive channels that participate in the regulation of osmotic pressure changes within the cell.

### Carbohydrate active enzymes

**Figure 3:**
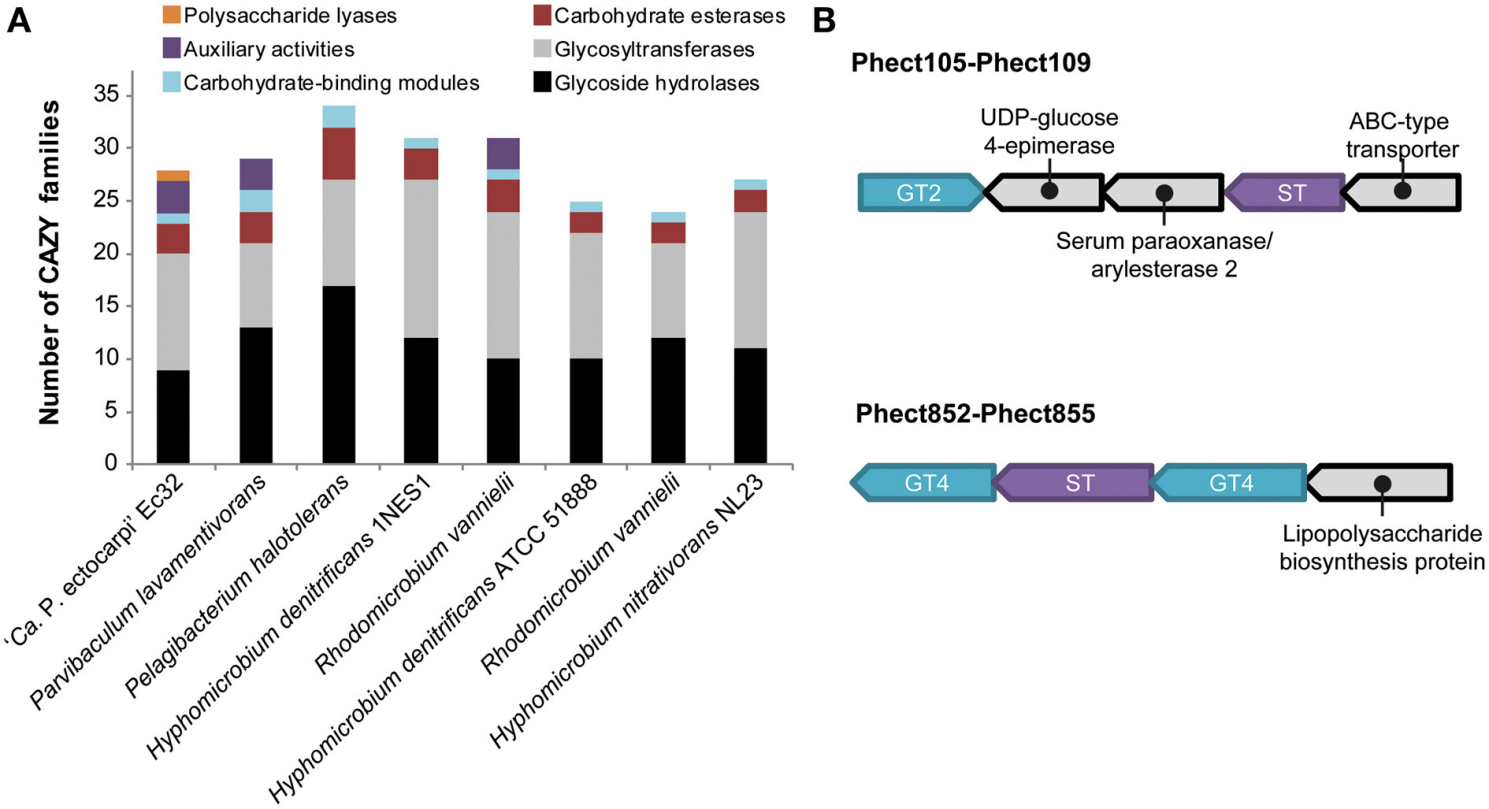
**Carbohydrate modifying enzymes. (A)** Number of CAZY families in the genome of “*Ca*. Phaeomarinobacter ectocarpi” Ec32 and selected *Rhizobiales*. **(B)** Organization of genes possibly involved in the degradation of sulfated fucans. GT, glycosyltransferase; ST, sulfotransferase.

CAZYmes regroup enzymes involved in the degradation or creation of glycosidic bonds and the modification of sugar moieties. In the “*Ca.* P. ectocarpi” genome, 66 CAZYmes belonging to 29 CAZY families were found. Among them, 9 glycoside hydrolase (GH) and 11 glycosyltransferase (GT) families have been identified (see Data sheet 3 for detailed annotations). These numbers are comparable to other phylogenetically close (Figure 1) *Alphaproteobacteria*, such as members of the clusters *Hyphomicrobiaceae* and *Parvibaculum*, which contain 10–17 GH and 8–15 GT families (Figure 3A, Data sheet 4).

Enzymes essential to the biosynthesis of bacterial glycogen are absent from the “*Ca.* P. ectocarpi” genome: there is no glycogen/starch synthase (families GT3 and GT5), no 1,4-alpha-glucan branching enzyme GlgB (GH13_9 subfamily), and no 4-α-glucanotransferase (GH77) (Ball and Morell, 2003). A glycogen phosphorylase (GT35 family, the key enzyme for the recycling of intracellular glycogen) is also missing. In contrast, “*Ca*. P. ectocarpi” possesses an alpha-amylase (Phect3079, EC 3.2.1.1, family GH13) likely to help degrade extracellular carbon sources, and the complete trehalose biosynthesis I pathway (TRESYN-PWY). Endogenous trehalose is likely recycled by an exo-acting enzyme, a GH15 trehalase (Phect47, EC 3.2.1.28), releasing two molecules of glucose. “*Ca*. P. ectocarpi” also possesses candidate genes for the degradation of chitin and chitosan. We found three enzymes of the CE4 family (Phect350, Phect3110, and Phect1064; the latter encoding a chitin deacetylase, EC 3.5.1.41), as well as a GH46 chitosanase (Phect2482, EC 3.2.1.132). These enzymes may act in synergy to degrade chitin: the CE4 enzymes convert N-acetyl-D-glucosamine into D-glucosamine residues, resulting in chitosan motives in the polysaccharide chain, which become substrates for the GH46 chitosanase. The GH3 beta-N-acetylhexosaminidase Phect3011 (EC 3.2.1.52) is also likely involved in chitin/chitosan catabolism. Interestingly, no homologs of characterized alginate lyases were found in “*Ca*. P. ectocarpi.” Nonetheless this bacterium features a protein (encoded by Phect1448) highly similar to non-classified polysaccharide lyases from diverse *Alphaproteobacteria* such as *Maricaulis maris* MCS10.

The “*Ca*. P. ectocarpi” genome contains 10 sulfatases (EC 3.1.6.-): eight formylglycine-dependent sulfatases (Phect92, Phect373, Phect661, Phect1492, Phect679, Phect1786, Phect2576, and Phect2896), and two alkyl sulfatases (Phect38 and Phect1167). Some of the formylglycine-dependent sulfatases may be involved in the degradation of sulfated polysaccharides, such as sulfated fucans produced by brown algae (Michel et al., 2010b). This hypothesis is strengthened by the presence of a GH29 alpha-L-fucosidase (Phect1478, EC 3.2.1.51, GH29 family). This enzyme could act in synergy with sulfatases to release fucose from sulfated fucose-containing polysaccharides or oligosaccharides, and constitutes a unique feature of “*Ca*. P. ectocarpi” with respect to other *Alphaproteobacteria*. Furthermore, we found two sulfotransferases (Phect108 and Phect853). These genes are localized in clusters including glycosyltransferases from families GT2 and GT4, and other carbohydrate-related proteins (UDP-glucose 4-epimerase, lipopolysaccharide protein) (Figure 3B). Therefore, these sulfotransferases are most likely involved in the biosynthesis of endogenous sulfated exopolysaccharides.

### “*Ca.* P. ectocarpi” and *E. siliculosus* have similar capacities to produce vitamins

The metabolic network of “*Ca*. P. ectocarpi” was examined with respect to its potential for vitamin production, and the retrieved pathways were assessed manually. “*Ca*. P. ectocarpi” is able to produce vitamin B1 (thiamine, PWY-6894), B2 (flavine, RIBOSYN2-PWY), B6 (pyridoxine, PWY0-845), B7 (biotine, BIOTIN-BIOSYNTHESIS-PWY), C (ascorbate, PWY3DJ-35471 and PWY-6415), and K2 (menaquinone; PWY-5849, PWY-5839, and MENAQUINONESYN-PWY). Several of the genes involved in these pathways were predicted to be organized in transcriptional units.

In order to establish if these vitamins could be of physiological interest for brown algae, and in particular *E. siliculosus*, the ability of the latter alga to produce these vitamins was investigated. Corresponding genes were searched for in the algal genome (Cock et al., 2010) as well as in a recent metabolic network reconstruction (http://ectogem.irisa.fr/, Prigent et al., pers. com.) and compared to our results for “*Ca.* P. ectocarpi.” This analysis indicated that all of these vitamins can be produced by *E. siliculosus* independently of the bacterium. Thiamine is an important co-factor for catabolism of amino acids and sugars, and several proteins in the *Ectocarpus* genome were found to contain a domain of the superfamily thiamin diphosphate-binding fold (THDP-binding), indicating that these enzymes depend on thiamin as a cofactor. However, *E. siliculosus* also features a bacteria-like thiamine pyrophosphatase synthesis pathway (PWY-6894), and no genes involved in thiamine transport have been identified in the algal genome. Flavin is a precursor for the synthesis of flavine adenine dinucleotide (FAD) and flavine mononucleotide (FMN), and the algal genome contains numerous flavoproteins and proteins with FAD binding domains. However, several enzymes similar to those involved in bacterial/plant, fungal, and mammalian pathways for flavin synthesis were identified in *E. siliculosus* (RIBOSYN2-PWY). Pyridoxine is degraded by the pyridoxal salvage pathway to produce pyridoxal phosphate, a co-factor important for many reactions related to amino acid metabolism (transamination, deamination, and decarboxylation). In *E. siliculosus* the salvage pathway for the synthesis of this compound has been identified (PLPSAL-PWY). Biotin is a vitamin involved in sugar and fatty acid metabolism, and several biotin-dependent carboxylases, i.e., enzymes featuring a biotin-binding site (IPR001882), have been annotated in the *E. siliculosus* genome. Again the algal genome encodes two enzymes likely to catalyze the three enzymatic reactions necessary to synthesize biotin from 8-amino-7-oxononanoate (Esi0392_0016, a bifunctional dethiobiotin synthetase/7,8-diamino-pelargonic acid aminotransferase; Esi0019_0088, a biotin synthase) (PWY0-1507). Ascorbate is an essential vitamin in plants where it serves as antioxidant in chloroplasts and as a cofactor for some hydroxylase enzymes (Smirnoff, 1996), and we found an L-galactose (plant-type) pathway for ascorbate synthesis in *E. siliculosus* (PWY-882). Lastly, the *E. siliculosus* genome encodes several methyltransferases potentially involved in the last step of vitamin K2 synthesis, in particular for menaquinol-6, -7 and -8 (Esi0009_0155, Esi0182_0017, and Esi0626_0001).

In contrast to the aforementioned vitamins, vitamin B12 cannot be produced by either “*Ca*. P. ectocarpi” or *E. siliculosus.* The “*Ca.* P. ectocarpi” genome encodes only a few genes similar to those involved in aerobic or anaerobic cobalamin synthesis, and the aforementioned presence of a vitamin-B12 importer indicates that “*Ca.* P. ectocarpi” may itself be vitamin-B12 auxotroph. In the same vein, it has been recently described that *E. siliculosus* is not able to produce vitamin B12, but that it can grow without external source of this compound. However, the *E. siliculosus* genome contains numerous vitamin B12-dependent enzymes (Helliwell et al., 2011), suggesting that vitamin B12 may nevertheless be beneficial for the alga. Finally, the absence of a gene coding for a 2-dehydropantoate 2-reductase (EC 1.1.1.169) in both “*Ca.* P. ectocarpi” and *E. siliculosus* suggests that neither of these organisms is able to synthesize vitamin B5 (pantothenic acid).

### Bacterial growth factors may influence algal growth and development

Auxin (indole-3-acetic acid, IAA) is an important plant hormone for which several biosynthetic pathways have been described in the green lineage and in bacteria (Woodward and Bartel, 2005; Nafisi et al., 2007; Sugawara et al., 2009). These pathways generally produce auxin from tryptophan (Trp) via different intermediates such as indole-3-pyruvate, tryptamine, indole-3-acetonitrile, or indole-3-acetamide. The “*Ca.* P. ectocarpi” genome encodes several genes involved in the synthesis of auxin from these intermediates (PWY-3161, PWY-5025, PWY-5026), but genes necessary to produce these intermediates from Trp were not found. In cultures of *E. siliculosus*, however, several forms of auxin were detected despite the probable absence of key enzymes for its synthesis in the algal genome (Le Bail et al., 2010). We therefore examined the possibility of synergistic auxin production by both “*Ca.* P. ectocarpi” and *E. siliculosus*.

**Figure 4:**
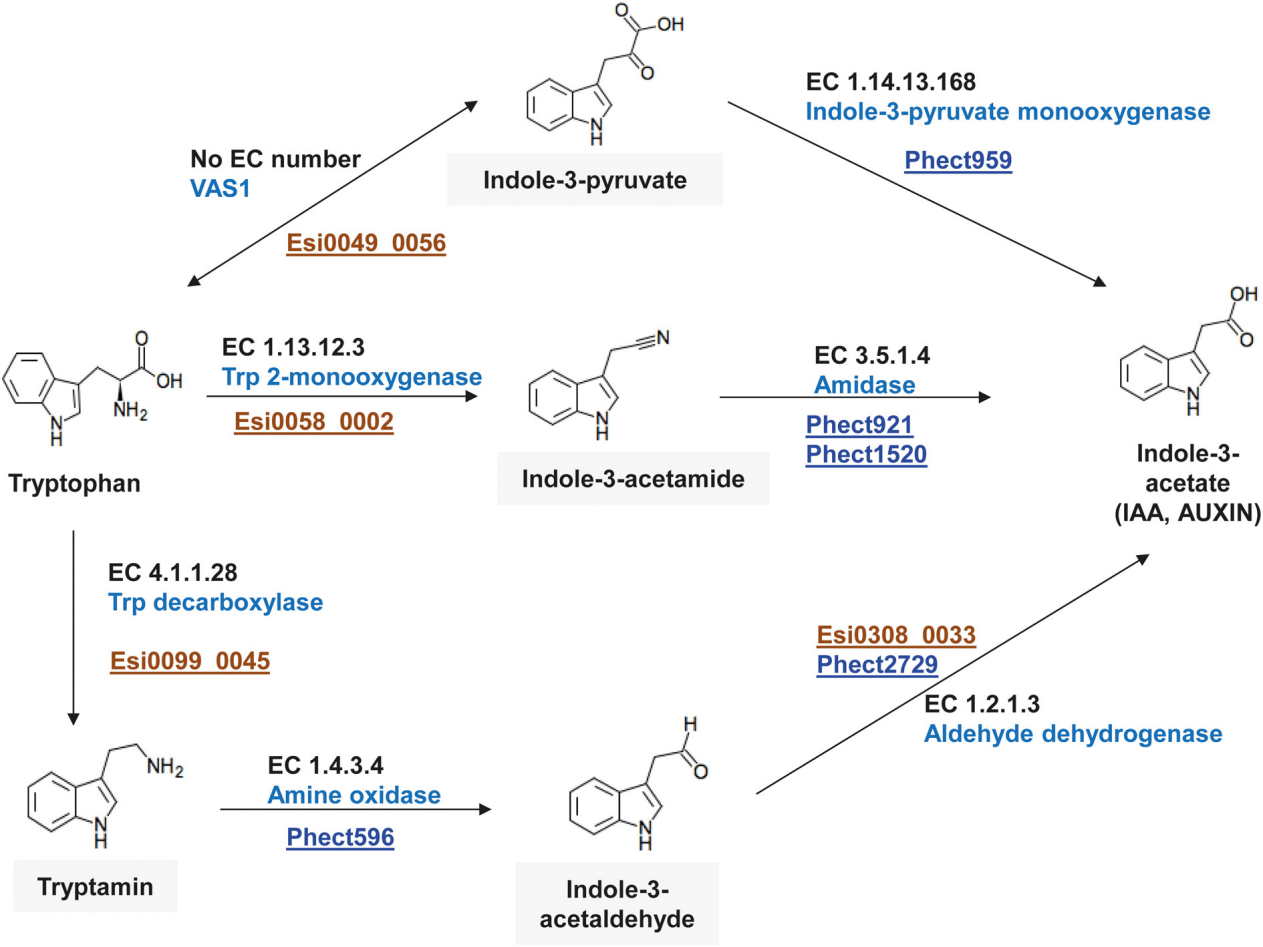
**Genes involved in tryptophan-dependent auxin synthesis in “*Ca.* Phaeomarinobacter ectocarpi” Ec32 (blue loci) and *E. siliculosus* (brown loci)**.

Three possible pathways were identified (Figure 4), all of them using Trp as substrate. In each case the first step involves an *E. siliculosus*-encoded enzyme to produce the intermediate that is then further metabolized by the bacterium. The first candidate pathway involves an ortholog of the pyridoxal-phosphate-dependent aminotransferase VAS1 (Esi0049_0056). This enzyme has been characterized in *Arabidopsis thaliana* and catalyzes the reversible conversion between indole-3-pyruvate and Trp (Zheng et al., 2013). Indole-3-pyruvate can then be transformed to auxin via the activity of the bacterial indole-3-monooxygenase (Phect959). In the second candidate pathway, Trp is transformed to indole-3-acetamide via the activity of a Trp-2-monoxygenase (Esi0058_0002) and a bacterial amidase (Phect929 or Phect1520). The last candidate pathway comprises three reactions: tryptamine is produced via the activity of a Trp decarboxylase (Esi0099_0045), and acts as a substrate for a bacterial amine oxidase (Phect596) producing indole-3-acetaldehyde. An aldehyde dehydrogenase such as Phect2729 may then convert indole-3-acetaldehyde to auxin.

In addition to these three cooperative pathways “*Ca*. P. ectocarpi” also possesses an ortholog of an indole synthase (Phect 1840, 43% of amino acid sequence identity with its *A. thaliana* ortholog), which may be implicated in a Trp-independent auxin biosynthesis pathway with indole-3-glycerol phosphate as substrate, although the different steps of this pathway remain to be elucidated (Zhang et al., 2008). Regardless of the biosynthetic pathway, auxin produced by “*Ca.* P. ectocarpi” may be exported from bacterial cells by members of the auxin efflux carrier family encoded by the bacterium, such as Phect1023 and Phect3211.

Cytokinins are another important actor in plant development and have functions related to auxin (El-Showk et al., 2013). We therefore examined if the “*Ca.* P. ectocarpi” genome encoded the enzymes required to produce cytokinins. A well-known example of a cytokinin-producing bacterium is *Rhodococcus fascians*. This microorganism is a phytopathogenic actinomycete with a broad host range, and causes severe commercial losses in the ornamental plant industry because it triggers malformations of shoots, referred to as leafy galls. In strain *R. fascians* D188, the virulence determinants are encoded by a large conjugative linear plasmid, pFiD188, and the pathology is induced by the secretion of a mix of six synergistically acting cytokinins (Pertry et al., 2010). Based on genes described to be important for *R. fascians* to produce cytokinins, we only found two loci in “*Ca.* P. ectocarpi” (Phect1727 and Phect255), both similar to the cytochrome P450 monooxygenase fasA.

In *A. thaliana*, however, cytokinin biosynthesis is controlled by different genes (Frébort et al., 2011). Here ATP/ADP isopentenyltransferase (IPT) genes encode the rate-limiting enzymes in cytokinin biosynthesis. Eight IPT genes (AtIPT1 and AtIPT3 through AtIPT9) are involved in the synthesis of the cytokinin precursor isopentenyladenine from dimethylallyl pyrophosphate. Two cytochrome P450 monooxygenases (CYP735A1 and CYP735A2) then catalyze the hydroxylation of isopentenyladenine, and the LONELY GUY (LOG) gene family is responsible for the conversion of cytokinin from an inactive to an active form. Homologs for genes involved in each of these three steps have been identified in the “*Ca.* P. ectocarpi” genome: an IPT (Phect427), the two aforementioned cytochrome P450 monooxygenases, and finally two LOG homologs (Phect2557 and Phect613). Other enzyme activities, such as those of zeatin isomerases (ZIs) or zeatin reductases (ZRs), have also been described to be involved in cytokinin biosynthesis, but no sequences are available for the corresponding enzymes, making it impossible to check for the presence of these genes in the bacterial genome.

## Discussion

“*Ca.* P. ectocarpi” Ec32, is a member of a new candidate species, genus, and family closely related to the order of *Rhizobiales*. So far, only a few sequences corresponding to this genus have been found, all of them in aquatic environments, and frequently associated with the brown algae *Fucus* and *Ectocarpus*. This indicates that members of “*Ca.* Phaeomarinobacter” are most likely specialized on this ecological niche. Several genera of *Rhizobiales* are known to form mutualistic relationships with terrestrial plants, but similar relationships with aquatic members closely related to this order have not yet been described. With this in mind, we here examine the possibility of mutualistic relationships between “*Ca.* P. ectocarpi” and *E. siliculosus*.

### Beneficial effects of bacteria on the alga: growth factors?

A long-known beneficial effect of bacteria on algae is the production of growth factors. Experiments performed by Pedersén (1968, 1973), suggested that *E. fasciculatus*, a sister species of *E. siliculosus*, depends on bacterial cytokinins for normal growth and development in standard culture media. Here we have demonstrated that, at least from a genomic point of view, “*Ca.* P. ectocarpi” has the capacity to produce such cytokinins via a pathway similar to that *of A. thaliana*. Furthermore, “*Ca.* P. ectocarpi” was the only major bacterial “contaminant” in our antibiotic-treated algal cultures based on sequence data. If *E. siliculosus*, like *E. fasciculatus*, depends on bacterial cytokinins, and given that during the optimization of the protocol for antibiotic treatments any conditions that did not yield well-growing cultures were discarded, this may not be a coincidence: this procedure may indeed have led to the active selection of an algal culture containing at least one bacterium able to produce these compounds.

A second potential positive effect of “*Ca.* P. ectocarpi” on *E. siliculosus* may be the synthesis of auxin. In a previous study, Le Bail et al. (2010) detected auxin in antibiotics-treated cultures of *E. siliculosus*, and demonstrated this hormone to play a role in cell differentiation, but its biosynthetic pathway was only partially reconstructed. Although the existence of new specific enzymes or other derived pathways to synthesize auxin in *E. siliculosus* cannot be excluded, our analyses show that auxin synthesis may occur by “*Ca.* P. ectocarpi” or synergistically between *E. siliculosus* and the bacterium, assuming that intermediates can be exchanged between both organisms. In the light of the high antibiotic-resistance of “*Ca.* P. ectocarpi” and the fact that it does not grow on Zobell medium, which is commonly used to verify if an algal strain is bacteria-free, the presence of “*Ca.* P. ectocarpi” provides one possible explanation for the previous observation of auxin in *E. siliculosus* cultures.

While the advantage for alga-associated bacteria of being able to produce algal growth factors and thus to control the growth of their substrate and source of energy is evident, an important question is how an alga could benefit from evolving a dependence on these factors. Given that growth factors act as regulators and not directly in metabolic processes, we can speculate that these factors may function or have functioned as signals between algae and bacteria: if the presence of a bacterium has direct (positive) effects on the metabolism or on other aspects of algal physiology, then perceiving bacteria-produced growth factors may help the alga to adjust and optimize its metabolism and growth depending on the surrounding bacterial flora. In the following section, we will discuss the possibility of such direct positive interactions between “*Ca.* P. ectocarpi” and *E. siliculosus*.

### Possible metabolic interaction points – from nitrogen assimilation to vitamins

With the aid of genomic analyses and metabolic network reconstruction, we examined candidate processes that may underlie positive effects of “*Ca.* P. ectocarpi” on *Ectocarpus*. The findings we obtained using this approach were mixed. For example, the “*Ca.* P. ectocarpi” Ec32 genome did not contain features typical for the genomes of nodule-forming *Rhizobiales* such as symbiosis islands, and we did not find any evidence for the presence of a symbiotic plasmid. However, at least some of the operons responsible for nitrogen fixation in nodule-forming *Rhizobiales* were present, thus neither supporting nor excluding a role of “*Ca.* P. ectocarpi” in algal nutrient assimilation. Similarly, the automatic analysis of the complementarity between the metabolic networks of “*Ca.* P. ectocarpi” and *E. siliculosus* did not reveal any confirmed metabolic reactions of the bacterium that complete gaps in the network of the alga. On the other hand, this analysis only assessed the producibility of a limited set of target metabolites and the minimal set of reactions needed to produce them, excluding any generic reactions in either of the networks. “*Ca.* P. ectocarpi” possesses a wide variety of transporters as typical also for *Rhizobiales* (Boussau et al., 2004). Transporters have previously been suggested to play key roles in inter-species interactions of *Rhizobiales* (MacLean et al., 2007). Some of these transporters may, for example, be involved in the exchange of vitamins. Although our results indicate that *E. siliculosus* and “*Ca.* P. ectocarpi” have similar capacities to produce vitamins, this does not exclude beneficial effect of bacteria-produced vitamins on the alga and/or vice versa. Indeed, *E. siliculosus* is frequently cultivated in Provasoli-enriched seawater medium, which comprises thiamine and biotin (compounds producible by both the bacterium and the alga). Additional experiments with truly axenic algal cultures would be required to verify if these vitamins actually benefit algal growth.

### Algae—an energy source for bacteria

Beneficial effects of *Ectocarpus* on “*Ca.* P. ectocarpi,” on the other hand, are evident. Marine *Alphaproteobacteria* are known to contain few CAZYmes. A prominent example for this is the SAR11 clade, which contains only the essential CAZYmes, allowing it to thrive under oligotrophic conditions (Teeling et al., 2012). However, *Alphaproteobacteria* are generally not able to perform photosynthesis and are thus dependent on an external source of carbohydrates and energy. This is also true for “*Ca*. P. ectocarpi.” For instance, this bacterium does not produce glycogen. However, it has the capacity to synthesize trehalose, a sugar used by bacteria as compatible osmolyte or as structural component (Argüelles, 2000). It may furthermore use trehalose as osmoprotector, as suggested for *Rhizobium etli* (Reina-Bueno et al., 2012).

A particularity of the “*Ca*. P. ectocarpi” genome is that, unlike many *Alphaproteobacteria* (Data sheet 4), it also encodes all enzymes required to recycle trehalose (GT20, GH15, trehalose-6-phosphatase) (Brown et al., 2011; Schleheck et al., 2011; Huo et al., 2012; Martineau et al., 2013). This indicates that “*Ca*. P. ectocarpi” may also utilize trehalose synthesized by *E. siliculosus* (Michel et al., 2010a) as a carbon source. It is furthermore predicted to have the capacity to degrade sulfated fucans, which, along with cellulose and alginate, represent a main component of brown algal cell walls (Michel et al., 2010b; Popper et al., 2011). Finally, the non-classified polysaccharide lyase found in the genome of “*Ca.* P. ectocarpi” constitutes a unique feature as none of the other selected *Alphaproteobacteria* contain homologous proteins. This protein may also be involved in the degradation of components from the cell wall of brown algae.

In addition to the degradation of brown algal polysaccharides, “*Ca*. P. ectocarpi” is also able to degrade other external sources of carbon such as chitin or chitosan. The former compound consists of beta-1,4-N-acetylglucosamine residues and is the main component of the cell wall / exoskeleton of fungi, diatoms, and crustaceans. Chitosan is a deacylated form of chitin and also naturally occurs in fungal cell walls. Although the “*Ca*. P. ectocarpi” genome does not contain any GH18 chitinase, chitin and chitosan may be degraded through an alternative pathway using a CE4 N-acetylglucosamine deacetylase, a GH46 chitosanase, and a GH3 beta-N-acetylhexosaminidase. This pathway may be an originality of “*Ca*. P. ectocarpi,” as none of the other examined *Alphaproteobacteria* contain all three enzymes (Data sheet 4; Brown et al., 2011; Schleheck et al., 2011; Huo et al., 2012; Martineau et al., 2013). Furthermore, the alpha-amylase (GH13) found in “*Ca*. P. ectocarpi,” coupled with other enzymes from additional associated bacteria, may serve the degradation of starch from green or red algae, or bacterial glycogen.

## Conclusion and prospects

“*Ca.* Phaeomarinobacter” and brown algae are frequent companions. Although we did not find any indication that “*Ca.* P. ectocarpi” and *Ectocarpus* are mandatory symbionts, both organisms have a clear potential to interact on several levels and even form a mutualistic relationship. As we know that algal-bacterial interactions play key roles in algal biology (Goecke et al., 2010; Hollants et al., 2013), further exploring these relationships is of utmost importance to understanding how these organisms function. Here we show that genomic analyses combined with metabolic network reconstruction provide a useful tool to start addressing this challenge. These methods will complement our ongoing effort to isolate bacterial strains from algal cultures, as an important advantage of these approaches is that they are not limited to cultivable bacteria. This provides an opportunity to catch a glimpse of the hidden bacterial diversity and its potential biological functions in algae. In this sense, genomics and next generation sequencing have increased the depth in which we can perceive and study holobiont systems in a way similar to the development of microscopy a few 100 years ago. As we improve the quality of the available metabolic networks, e.g., via better reconstruction pipelines and via targeted experiments assessing the function of yet unknown enzymes or transporters, we believe these approaches will further gain in importance.

### Conflict of interest statement

The authors declare that the research was conducted in the absence of any commercial or financial relationships that could be construed as a potential conflict of interest.

## Supplementary material

Data sheet 1 **16S rRNA sequences and complete phylogenetic tree.** The superscript letter ^T^ in the “strain” column indicates type species and type strains, respectively. The genera included between brackets means that the strains are probably misnamed. The taxonomic classification used was obtained from the EzTaxon server (http://www.ezbiocloud.net/eztaxon). The maximum likelihood tree showing the position of “*Ca.* Phaeomarinobacter ectocarpi” Ec32 within the *Alphaproteobacteria* was based on an alignment with masked hyper-variable regions. The *Gammaproteobacterium Escherichia coli* was used as outgroup.

Data sheet 2 **Functional comparison of the “*Ca.* Phaeomarinobacter ectocarpi” Ec32 and of the *Parvibaculum lavamentivorans* DS-1^**T**^ genomes carried out using the RAST platform**.

Data sheet 3 **Summary of manually annotated transporters and CAZYmes**.

Data sheet 4 **CAZYmes in “*Ca.* Phaeomarinobacter ectocarpi” and phylogenetically closely related *Alphaproteobacteria*.** The total number of genes per family is indicated for each CAZY class. A stronger red background indicates a higher number of genes.
