# Supplementary material for: Genome and metabolic network of “Candidatus Phaeomarinobacter ectocarpi” Ec32, a new candidate genus of Alphaproteobacteria frequently associated with brown algae
Source: Front Genet. 2014 Jul 25;5:241. doi: 10.3389/fgene.2014.00241 (PMC4110880; doi:10.3389/fgene.2014.00241)

Data sheet 1. 16S rRNA sequences and complete phylogenetic tree.

Table of sequence accessions: The superscript letter ^{T} in the "strain" column indicates type species and type strains, respectively. The genera included between brackets means that the strains are probably misnamed. The taxonomic classification used was obtained from the EzTaxon server (http://www.ezbiocloud.net/eztaxon).

Taxonomic classification	Strain	Sequence accession
	"*Candidatus* Phaeomarinobacter ectocarpii" 32	HG966617.1
	Uncultured bacterium 47-S-68	JN018674.1
	Alpha proteobacterium GMD21A06	AY162106.1
	Alpha proteobacterium GMD21D06	AY162100.1
RHIZOBIALES		
Rhizobiaceae	*Agrobacterium albertimagni* AOL15^{T}	AF316615.1
	Agrobacterium larrymoorei AF3.10^{T}	NR_026519.1
	Agrobacterium rhizogenes NBRC 13257^{T}	AB680381.1
	Agrobacterium rubi NBRC 13261	AB680385.1
	Agrobacterium tumefaciens^{T} B6^{T}	D14500.1
	Agrobacterium viscosum CICC10215	AY794055.1
	Blastobacter capsulatus Muller 216^{T}	X73042.1
	Ensifer adhaerens^{T} A^{T}	NR_042482.1
	Ensifer garamanticus ORS 1400^{T}	AY500255.1
	Ensifer mexicanus ITTG-R7^{T}	DQ411930.1
	Ensifer numidicus ORS 1407^{T}	AY500254.1
	Ensifer sojae CCBAU 05684^{T}	AJQT01000154.1
	Rhizobium giardinii H152^{T}	U86344.1
	Rhizobium huautlense SO2^{T}	NR_024863.1
	Rhizobium indigoferae AS1.3054^{T}	AF364068.1
	Rhizobium leguminosarum^{T} viciae 3Hoq18^{T}	U29386.1
	Rhizobium phaseoli ATCC 14482^{T}	EF141340.1
	Sinorhizobium americanum CFNEI 156^{T}	AF506513.3
	Sinorhizobium arboris TTR 38^{T}	NR_037001.1
	Sinorhizobium fredii^{T} PRC 205^{T}	D14516.1
	Sinorhizobium kostiense TTR 15^{T}	NR_104565.1
	Sinorhizobium kummerowiae AS1.3046^{T}	AF364067.1
Parvibaculum family	*Parvibaculum hydrocarboniclasticum* EPR92^{T}	GU574708.1
	Parvibaculum indicum P31^{T}	FJ182044.1
	Parvibaculum lavamentivorans^{T} DS-1^{T}	AY387398.1
Breoghania family	*Breoghania corrubedonensis*^{T} UBF-P1^{T}	NR_104495.1
Bartonnellaceae	*Bartonella acomydis* KS2-1^{T}	AB602533.1
	Bartonella alsatica IBS 382^{T}	AJ002139.1
	Bartonella australis Aust	DQ538394.1
	Bartonella bacilliformis^{T} KC583^{T}	M65249.1
	Bartonella birtlesii IBS 325^{T}	AF204274.1
	Daeguia caeni^{T} K107^{T}	EF532794.1
	[*Wolbachia*] *melophagi* MO6	X89110.1
Brucellaceae	*Brucella Abortus* 544^{T}	AM158979.1
	Brucella canis RM6	L37584.1
	Brucella ceti NCTC 12891^{T}	AM158982.1
	Brucella inopinata BO1^{T}	EU053207.1
	Brucella melitensis^{T} 16M^{T}	AY594215.1
	Brucella neotomae 5K33^{T}	AY594216.1
	Mycoplana dimorpha^{T} TK0055^{T}	D12786.1
	Mycoplana ramosa TK0053^{T}	EU022308.1
	Ochrobactrum anthropi^{T} LMG 3331^{T}	NR_074243.1

	Ochrobactrum ciceri Ca-34^{T}	DQ647056.2
	Ochrobactrum cytisi ESC1^{T}	AY776289.1
	Ochrobactrum gallinifaecis Iso 196^{T}	AJ519939.1
	Ochrobactrum grignonense Oga9a^{T}	NR_028901.1
	Paenochrobactrum gallinarii^{T} Sa25^{T}	FN391023.1
	Paenochrobactrum glaciei Pi26^{T}	AB369864.1
	Pseudochrobactrum asaccharolyticum^{T} CCUG 46016^{T}	NR_042474.1
	Pseudochrobactrum kiredjianiae CCUG 49584^{T}	NR_042519.1
	Pseudochrobactrum lubricantis KSS 7.8^{T}	NR_104538.1
	Pseudochrobactrum saccharolyticum JUgrA^{T}	NR_042473.1
Shinella family	*Shinella daejeonensis* MJ02^{T}	GQ241319.1
	Shinella fusca DC-196^{T}	FM177879.1
	Shinella granuli^{T} Ch06^{T}	AB187585.1
	Shinella kummerowiae CCBAU 25048^{T}	EF070131.1
	Shinella yambaruensisgi MS4^{T}	AB285481.1
	Shinella zoogloeoides I-16-M^{T}	X74915.1
Phyllobacteriaceae	*Aliihoeflea aestuarii*^{T} N8^{T}	EF660756.3
	Aminobacter aganoensis TH-3^{T}	AJ011760.1
	Aminobacter aminovorans^{T} L383^{T}	AJ011759.1
	Aminobacter anthyllidis STM4645^{T}	NR_108530.1
	Aminobacter ciceronei C147	AF246220.1
	Aminobacter lissarensis CC495^{T}	AF107722.1
	Aminobacter niigataensis DM81^{T}	AJ011761.1
	Aquamicrobium aerolatum Sa14^{T}	FM210786.1
	Aquamicrobium aestuarii G210^{T}	NR_108709.1
	Aquamicrobium ahrensii 905	NR_108146.1
	Aquamicrobium defluvii^{T} NKK^{T}	Y15403.1
	Aquamicrobium lusatiense S1^{T}	NR_025312.1
	Aquamicrobium segne 1006	NR_108145.1
	[*Carbophilus*] *carboxidus*^{T} Z-1171^{T}	NR_104931.1
	Chelativorans multitrophicus^{T} DSM 9103^{T}	EF457243.1
	Chelativorans oligotrophicus LPM4^{T}	EF457242.1
	Hoeflea halophila JG120-1^{T}	NR_108835.1
	Hoeflea marina^{T} A43^{T}	AY598817.1
	Hoeflea phototrophica DFL-43^{T}	JF957616.1
	Hoeflea siderophila Hf1^{T}	EU670237.1
	Hoeflea suaedae YC6898^{T}	NR_109036.1
	Mesorhizobium chacoense PR5^{T}	AJ278249.1
	Mesorhizobium huakuii 103^{T}	D13431.1
	Mesorhizobium loti^{T} ATCC 33669^{T}	D14514.1
	Mesorhizobium septentrionale SDW014^{T}	NR_025252.1
	Mesorhizobium temperatum SDW018^{T}	AF508208.1
	Nitratireductor aquibiodomus^{T} NL21^{T}	AF534573.1
	Nitratireductor aquimarinus CL-SC21^{T}	HQ176467.1
	Nitratireductor basaltis J3^{T}	NR_044414.1
	Nitratireductor indicus C115^{T}	GU447302.1
	Nitratireductor kimnyeongensis KY 101^{T}	AM498744.1
	Phyllobacterium catacumbae CSC19^{T}	AY636000.1
	Phyllobacterium endophyticum PEPV15^{T}	NR_109517.1
	Phyllobacterium ifriqiyense STM 370^{T}	AY785325.1
	Phyllobacterium leguminum ORS 1419^{T}	AY785323.1
	Phyllobacterium myrsinacearum^{T} STM 948^{T}	AY785315.1
	Pseudaminobacter defluvii THI 051	D32248.1
	Pseudaminobacter salicylatoxidans^{T} BN12^{T}	AF072542.1
	Thermovum composti^{T} Nis3^{T}	AB563785.2
Rhodobiaceae	*Lutibaculum baratangense*^{T} AMV1^{T}	FN297835.1

	Rhodobium gokarnense JA173^{T}	AM180706.1
	Rhodobium orientis^{T} MB312^{T}	D30792.1
Cohaesibacteraceae	*Ahrensia kielensis*^{T} B9^{T}	D88524.1
	Cohaesibacter gelatinilyticus^{T} CL-GR15^{T}	NR_043777.1
	Cohaesibacter haloalkalitolerans JC131^{T}	NR_108886.1
	Cohaesibacter marisflavi DQHS21^{T}	GQ200200.2
	Pseudahrensia aquimaris^{T} HDW-32^{T}	NR_108837.1
Devosia family	*Cucumibacter marinus*^{T} CL-GR60^{T}	AUCR01000008.1:7262-8729
	Devosia insulae DS-56^{T}	EF012357.1
	Devosia limi R-21940^{T}	AJ786801.1
	Devosia neptuniae J1^{T}	AF469072.1
	Devosia riboflavina^{T} 4R3337^{T}	AJ549086.1
	Devosia soli GH2-10^{T}	DQ303125.1
	Maritalea mobilis E6^{T}	EU255260.1
	Maritalea myrionectae^{T} CL-SK30^{T}	EF988631.1
	Maritalea porphyrae LCM-3^{T}	AB583774.1
	Pelagibacterium halotolerans^{T} B2^{T}	NR_102924.1
	Pelagibacterium luteolum 1 C16 27^{T}	EF540455.1
	Vasilyevaea enhydra^{T} 9b^{T}	GQ221761.1
	Vasilyevaea mishustinii 17^{T}	FJ560749.1
Xanthobacteraceae	*Ancylobacter aquaticus*^{T} Orskov^{T}	M62790.1
	Ancylobacter dichloromethanicus DM16^{T}	EU589386.1
	Ancylobacter oerskovii NS05^{T}	AM778407.1
	Ancylobacter polymorphus AS 1.2800^{T}	AY211516.1
	Ancylobacter rudongensis AS1.1761^{T}	AY056830.1
	Ancylobacter vacuolatus AS1.2807^{T}	AY211515.1
	Angulomicrobium amanitiformis NCIMB 1785^{T}	AJ535709.1
	Angulomicrobium tetraedrale^{T} Z-2821^{T}	AJ535708.1
	Aquabacter spiritensis^{T} SPL-1^{T}	NR_104747.1
	Azorhizobium caulinodans^{T} ORS 571^{T}	D11342.1
	Azorhizobium doebereinerae BR5401^{T}	AF391130.1
	Azorhizobium oxalatiphilum NS12^{T}	FR799325.1
	Methylorhabdus multivorans^{T} DM13^{T}	AF004845.1
	Starkeya koreensis Jip08^{T}	AB166877.1
	Starkeya novella^{T} IAM 12100^{T}	D32247.1
	Xanthobacter agilis SA35^{T}	X94198.1
	Xanthobacter aminooxidans 14a^{T}	AF399969.1
	Xanthobacter autotrophicus^{T} 7c^{T}	X94201.1
	Xanthobacter flavus JW	X94206.1
	Xanthobacter polyaromaticivorans 127W^{T}	AB106864.1
Prosthecomicrobium family	*Kaistia adipata*^{T} Chj404^{T}	NR_042723.1
	Kaistia dalseonensis B6-8^{T}	NR_108142.1
	Kaistia defluvii B6-12^{T}	NR_108143.1
	Kaistia geumhonensis B1-1^{T}	NR_108141.1
	Kaistia granuli Ko04^{T}	NR_041362.1
	Prosthecomicrobium pneumaticum^{T} 1a^{T}	AB017203.1
Pleomorphomonas family	*Pleomorphomonas diazotrophica* R5-392^{T}	NR_109585.1
	Pleomorphomonas koreensis Y9^{T}	AB127972.1
	Pleomorphomonas oryzae^{T} F-7^{T}	AB159680.1
	[*Prosthecomicrobium*] *hirschii* 16^{T}	HM037994.1
Pseudoxanthobacter family	*Amorphus coralli*^{T} RS.Sph.026^{T}	DQ097300.1
	Amorphus orientalis YIM D10^{T}	FJ998414.1
	Amorphus suaedae YC6899^{T}	KC006961.1
	Pseudoxanthobacter soli^{T} CC-CC4^{T}	EF465533.1
Beijerinckiaceae	*Beijerinckia derxii* derxii Q13^{T}	AJ563933.1

	Beijerinckia doebereinerae LMG 2819^{T}	EU401905.1
	Beijerinckia indica^{T} indica Starkey11^{T}	AJ563930.1
	Beijerinckia mobilis WR237^{T}	AB119200.1
	Methylocapsa acidiphila^{T} B2^{T}	AJ278726.1
	Methylocapsa aurea KYG^{T}	FN433469.1
	Methylocella palustris^{T} K^{T}	Y17144.1
	Methylocella silvestris BL2^{T}	AJ491847.1
	Methylocella tundrae Y1	AJ563928.1
	Methyloferula stellata^{T} AR4^{T}	ARWA01000001.1
	Methylorosula polaris^{T} V-022^{T}	EU586035.1
	Methylovirgula ligni^{T} BW863^{T}	NR_044611.1
	Rhodoblastus acidophilus^{T} 7050^{T}	FR733696.1
	Rhodoblastus sphagnicola RS^{T}	AM040096.1
Methylocystaceae	*Methylocystis bryophila* H2s^{T}	FN422003.1
	Methylocystis echinoides IMET 10491^{T}	AJ458473.1
	Methylocystis heyeri H2^{T}	AM283543.1
	Methylocystis hirsuta CSC1^{T}	NR_043754.1
	Methylocystis parvus^{T} OBBP^{T}	Y18945.1
	Methylocystis rosea SV97^{T}	AJ414656.1
	Methylosinus sporium NR3K	EF619620.1
	Methylosinus trichosporium^{T} OB3b^{T}	Y18947.1
Methylobacteriaceae	*Bosea eneae* CRIB-11	DQ440825.1
	Bosea massiliensis 63287^{T}	NR_025118.1
	Bosea minatitlanensis AMX51^{T}	AF273081.1
	Bosea thiooxidans^{T} BI-42^{T}	AF508803.1
	Bosea vestrisii 34635^{T}	AF288306.1
	Camelimonas abortus UK34	FR851926.1
	Camelimonas lactis^{T} M 2040^{T}	FN430422.1
	Chelatococcus asaccharovorans^{T} TE2^{T}	AJ294349.1
	Chelatococcus daeguensis K106^{T}	EF584507.1
	Chelatococcus sambhunathii HT4^{T}	DQ322070.2
	Methylobacterium adhaesivum AR27^{T}	AM040156.1
	Methylobacterium aminovorans TH-15^{T}	AB175629.1
	Methylobacterium aquaticum GR16^{T}	AJ635303.1
	Methylobacterium organophilum^{T} XX^{T}	AB175638.1
	Methylobacterium populi BJ001^{T}	AJ549955.1
	Microvirga aerilata 5420S-16^{T}	GQ421849.1
	Microvirga flocculans TFB^{T}	AB098515.1
	Microvirga lotononidis WSM3557^{T}	HM362432.1
	Microvirga lupini Lut6^{T}	EF191408.1
	Microvirga subterranea^{T} FaiI4^{T}	NR_104766.1
	Salinarimonas ramus SL014B-41A4^{T}	GU125653.2
	Salinarimonas rosea^{T} YIM YD3^{T}	EU878006.2
Blastochloris family	*Blastochloris gulmargensis* JA248^{T}	AM502287.1
	Blastochloris sulfoviridis P1^{T}	D86514.1
	Blastochloris viridis^{T} F^{T}	AF084495.1
"Aurantimonadaceae"	*Aurantimonas coralicida*^{T} WP1^{T}	AJ786361.1
	Aurantimonas litoralis HTCC2156^{T}	AY178863.1
	Aurantimonas manganoxydans SI85-9A1^{T}	U53824.1
	Aureimonas altamirensis^{T} IARI-ABL-26	KC581669.1
	Aureimonas ferruginea CC-CFT023^{T}	JQ864240.1
	Aureimonas frigidaquae CW5^{T}	EF373540.1
	Aureimonas jatrophae L7-484^{T}	JQ346805.1
	Aureimonas phyllosphaerae L9-753^{T}	JQ346806.1
	Aureimonas rubiginis CC-CFT034^{T}	JQ864241.1
	Aureimonas ureolyica 5715S-12^{T}	DQ883810.1

	Fulvimarina pelagi^{T} HTCC2506^{T}	AY178860.1
Hyphomicrobiaceae	*Ancalomicrobium adetum*^{T} 4a:2^{T}	NR_104726.1
	Dichotomicrobium thermohalophilum^{T} DSM 5002^{T}	NR_104742.1
	Filomicrobium fusiforme^{T} 128^{T}	Y14313.1
	Filomicrobium insigne SLG5B-19^{T}	NR_044095.1
	Hyphomicrobium chloromethanicum CM2^{T}	AF198623.1
	Hyphomicrobium denitrificans X^{T}	Y14308.1
	Hyphomicrobium facile H-526^{T}	Y14309.1
	Hyphomicrobium hollandicum KB-677^{T}	Y14303.1
	Hyphomicrobium vulgare^{T} NQ-521 B^{T}	AB543807.2
	Pedomicrobium americanum ACM 3090	X97692.1
	Pedomicrobium australicum IFAM WD1355	X97694.1
	Pedomicrobium ferrugineum^{T} S-122^{T}	GU269548.1
	Pedomicrobium manganicum ACM 3038	X97691.1
	Rhodomicrobium udaipurense JA755	HE863941.1
	Rhodomicrobium vannielii^{T} DSM 162^{T}	FN666247.1
Rhodoligotrophos family	*Nordella oligomobilis* N21^{T}	AF370880.1
	Rhodoligotrophos appendicifer^{T} 120-1^{T}	AB617575.1
Anderseniella family	*Anderseniella baltica*^{T} BA141^{T}	AM712634.1
Afifella family	*Afifella marina*^{T} BN 126^{T}	D30790.1
	Afifella pfennigii AR2102^{T}	EU445271.1
	[*Rhodopseudomonas*] *julia* KR-11-67^{T}	AB087720.1
Bradyrhizobiaceae	*Afipia birgiae* 34632^{T}	AF288304.1
	Afipia broomeae C-20	AY568506.2
	Afipia clevelandensis B-91-007353^{T}	M69186.1
	Afipia felis^{T} B-91-007352^{T}	M65248.1
	Afipia massiliensis 34633^{T}	NR_025646.1
	Bradyrhizobium arachidis CCBAU 051107^{T}	HM107167.1
	Bradyrhizobium betae PL7HG1^{T}	AY372184.1
	Bradyrhizobium daqingense CCBAU 15774^{T}	KJ184551.1
	Bradyrhizobium japonicum^{T} SEMIA 5079	AF234888.2
	Bradyrhizobium jicamae PAC68^{T}	NR_043036.1
	Nitrobacter alkalicus AN1^{T}	AF069956.1
	Nitrobacter hamburgensis X14^{T}	L11663.1
	Nitrobacter winogradskyi^{T} Nb-255^{T}	AM114522.1
	Oligotropha carboxidovorans^{T} OM5^{T}	FR733697.1
	Pseudolabrys taiwanensis^{T} CC-BB4^{T}	DQ062742.1
	Rhodoplanes cryptolactis DSM 9987^{T}	AB087718.1
	Rhodoplanes elegans AS130^{T}	D25311.1
	Rhodoplanes piscinae JA266^{T}	AM712913.1
	Rhodoplanes pokkaliisoli JA415^{T}	NR_104542.1
	Rhodoplanes roseus^{T} 941^{T}	D25313.1
	Rhodoplanes serenus TUT3530^{T}	NR_040936.1
	Rhodopseudomonas faecalis gc^{T}	AF123085.2
	Rhodopseudomonas harwoodiae JA531^{T}	NR_108486.1
	Rhodopseudomonas palustris^{T} ATH 2.1.6^{T}	D25312.1
	Rhodopseudomonas rhenobacensis Rb^{T}	AB087719.1
	Seliberia stellata^{T} B-1340^{T}	HE795128.1
	Tardiphaga robiniae^{T} R-45977^{T}	FR753034.1
Labrys family	*Labrys methylaminiphilus* JLW10^{T}	NR_041336.1
	Labrys miyagiensis G24116	AB236171.1
	Labrys monachus^{T} 42^{T}	AJ535707.1
	Labrys okinawaensis MAFF 210191^{T}	AB236169.1
	Labrys wisconsinensis W1215-PCA-4^{T}	EF382666.1
Bauldia family	*Bauldia consociata* 11^{T}	FJ560750.1
	Bauldia litoralis^{T} 524-16^{T}	GQ221764.1

Stappia family	*Labrenzia aggregata* B1^{T}	D88520.1
	Labrenzia alba 5OM6^{T}	AJ878875.1
	Labrenzia alexandrii^{T} DFL-11^{T}	ACCU01000015.1
	Labrenzia marina mano18^{T}	AY628423.1
	Nesiotobacter exalbescens^{T} LA33B^{T}	AF513441.1
	Pannonibacter indicus HT23^{T}	EF608175.3
	Pannonibacter phragmitetus^{T} C6-19^{T}	AJ400704.1
	Polymorphum gilvum^{T} SL003B-26A1^{T}	NR_074240.1
	Pseudovibrio ascidiaceicola F423^{T}	AB175663.1
	Pseudovibrio axinellae Ad2^{T}	JN167515.1
	Pseudovibrio denitrificans^{T} DN34^{T}	AY486423.1
	Pseudovibrio japonicus WSF2^{T}	AB246748.1
	Roseibium denhamense^{T} OCh 254^{T}	D85832.1
	Roseibium hamelinense OCh 368^{T}	D85836.1
	Stappia indica B106^{T}	EU726271.1
	Stappia stellulata^{T} 2M	D88525.1
	Stappia taiwanensis CC-SPIO-10^{T}	FR828537.1
Methylopila family	*Agaricicola taiwanensis*^{T} CC-SBABM117^{T}	FJ594057.1
	Albibacter methylovorans^{T} DM10^{T}	AF273213.1
	Hansschlegelia beijingensis PG04^{T}	NR_109533.1
	Hansschlegelia plantiphila^{T} S1^{T}	DQ404188.1
	Hansschlegelia zhihuaia S 113^{T}	DQ916067.1
	Methylopila capsulata^{T} IM1^{T}	AF004844.1
	Methylopila helvetica DM9^{T}	AF227126.1
	Methylopila jiangsuensis JZL-4^{T}	FJ502233.3
	Methylopila musalis MUSA^{T}	NR_109539.1
	Methylosulfonomonas methylovora^{T} M2^{T}	U62893.1
Martelella family	*Martelella endophytica* YC6887^{T}	HM800924.1
	Martelella mediterranea^{T} MACL11^{T}	AY649762.1
Meganema family	*Meganema perideroedes*^{T} Gr1^{T}	50788076AJ578476.1
Rhizomicrobium family	*Rhizomicrobium electricum* Mfc52^{T}	NR_108115.1
	Rhizomicrobium palustre^{T} A48^{T}	AB081581.3
CAULOBACTERALES		
Caulobacteraceae	*Asticcacaulis benevestitus* Z-0023^{T}	AM087199.1
	Asticcacaulis biprosthecium R-7290^{T}	AJ247193.1
	Asticcacaulis excentricus^{T} R-7289^{T}	AJ247194.1
	Asticcacaulis solisilvae CGM1-3EN^{T}	JX144961.1
	Asticcacaulis taihuensis T3-B7^{T}	AY500141.1
	Brevundimonas abyssalis TAR-001^{T}	AB688113.1
	Brevundimonas aveniformis EMB102^{T}	DQ372984.1
	Brevundimonas bacteroides CB7^{T}	AJ227782.1
	Brevundimonas basaltis J22^{T}	EU143355.1
	Brevundimonas diminuta^{T} AJ 2067^{T}	AB021415.1
	Caulobacter daechungensis H-E3-2^{T}	JX861096.1
	Caulobacter henricii CB 4^{T}	AJ227758.1
	Caulobacter mirabilis FWC 38^{T}	AJ227774.1
	Caulobacter segnis TK0059^{T}	AB023427.1
	Caulobacter vibrioides^{T} Stove CB51^{T}	AJ009957.1
	Phenylobacterium composti 4T-6^{T}	EU022524.1
	Phenylobacterium conjunctum FWC 21^{T}	AJ227767.1
	Phenylobacterium falsum AC-49^{T}	AJ717391.1
	Phenylobacterium haematophilum LMG 11050^{T}	AJ244650.1
	Phenylobacterium immobile^{T} E^{T}	Y18216.1
RHODOBACTERALES		
Rhodobacteraceae	*Actibacterium mucosum*^{T} R46^{T}	HE590855.1
	Albidovulum inexpectatum^{T} FRR-10^{T}	AF465833.1

Albidovulum xiamenense YBY-7^{T}	HQ709061.1
Albimonas donghaensis^{T} DS2^{T}	DQ280370.1
Albimonas pacifica p-50-3^{T}	JN837486.1
Amaricoccus kaplicensis^{T} Ben101^{T}	U88041.1
Amaricoccus macauensis Ben104^{T}	U88042.1
Amaricoccus tamworthensis Ben103^{T}	U88044.1
Amaricoccus veronensis Ben102^{T}	U88043.1
Antarctobacter heliothermus^{T} EL-219^{T}	Y11552.1
Celeribacter baekdonensis L-6^{T}	HM997022.1
Celeribacter neptunius^{T} H 14^{T}	FJ535354.1
Citreicella aestuarii AD8^{T}	FJ230833.2
Citreicella marina CK-I3-6^{T}	EU928765.2
Citreicella thiooxidans^{T} CHLG 1^{T}	AY639887.1
Citreimonas salinaria^{T} CL-SP20^{T}	AY962295.1
Defluviimonas aestuarii BS14^{T}	JN642270.2
Defluviimonas denitrificans^{T} D9-3^{T}	AM403214.1
Dinoroseobacter shibae^{T} DFL 12^{T}	NR_074166.1
Donghicola eburneus^{T} SW-277^{T}	DQ667965.1
Donghicola xiamenensis Y-2^{T}	DQ120728.1
Falsirhodobacter halotolerans^{T} JA744^{T}	HE662814.1
Gemmobacter aquatilis^{T} DSM 3857^{T}	FR733676.1
Gemmobacter caeni DCA-1^{T}	FJ386516.1
Gemmobacter changlensis JA139^{T}	AM399030.1
Gemmobacter fontiphilus JS43^{T}	FJ906694.2
Gemmobacter lanyuensis Orc-4^{T}	JN104393.1
Haematobacter massiliensis Framboise^{T}	DQ342309.1
Haematobacter missouriensis^{T} H1892^{T}	DQ342315.1
Hasllibacter halocynthiae^{T} KME 002^{T}	FJ638616.1
Huaishuia halophila^{T} ZXM137^{T}	FJ436725.1
Hwanghaeicola aestuarii^{T} Y26^{T}	FJ230842.1
Jannaschia aquimarina GSW-M26^{T}	HQ596528.1
Jannaschia donghaensis DSW-17^{T}	EF202612.1
Jannaschia helgolandensis^{T} Hel 10^{T}	AJ438157.1
Jannaschia pohangensis H1-M8^{T}	DQ643999.1
Jannaschia rubra 4SM3^{T}	AJ748747.1
Jhaorihella thermophila^{T} CC-MHSW-1^{T}	EU287912.1
Ketogulonicigenium robustum X6L^{T}	AF136850.1
Ketogulonicigenium vulgare^{T} 266-13B	AF136846.1
Leisingera aquimarina 29^{T}	AM900415.1
Leisingera methylohalidivorans^{T} MB2^{T}	AY005463.1
Leisingera nanhaiensis NH52F^{T}	FJ232451.1
Lentibacter algarum^{T} ZXM100^{T}	FJ436732.1
Litoreibacter albidus^{T} Sh18^{T}	AB518881.1
Litoreibacter arenae GA2-M15^{T}	EU342372.1
Litoreibacter halocynthiae P-MA1-7^{T}	JX644172.1
Litoreibacter janthinus Sd1^{T}	AB518880.1
Litoreibacter meonggei MA1-1^{T}	JN021667.1
Litorimicrobium taeanense^{T} G4^{T}	GQ232737.2
Litorisediminicola beolgyonensis^{T} BB-MW24^{T}	JQ807220.1
Loktanella agnita R10SW5^{T}	AY682198.1
Loktanella atrilutea IG8^{T}	AB246747.1
Loktanella cinnabarina LL-001^{T}	AB688112.1
Loktanella rosea Fg36^{T}	AY682199.1
Loktanella salsilacus^{T} AC 50^{T}	AJ440997.1
Lutimaribacter litoralis KU5D5^{T}	AB627076.1
Lutimaribacter pacificus W11-2B^{T}	DQ659449.2

Lutimaribacter saemankumensis^{T} SMK-117^{T}	EU336981.1
Mameliella alba^{T} JLT354-W^{T}	EU734592.1
Maribius pelagius B5-6^{T}	DQ514326.1
Maribius salinus^{T} CL-SP27^{T}	AY906863.1
Marinosulfonomonas methylotropha^{T} PSCH4^{T}	U62894.1
Marinovum algicola^{T} FF3^{T}	AB289592.1
Maritimibacter alkaliphilus^{T} HTCC 2654^{T}	DQ915443.3
Marivita byunsanensis SMK-114^{T}	FJ467624.1
Marivita cryptomonadis^{T} CL-SK44^{T}	EU512919.1
Marivita geojedonensis DPG-138^{T}	JN885198.1
Marivita hallyeonensis DPG-28^{T}	JF260872.1
Marivita litorea CL-JM1^{T}	EU512918.1
Methylarcula marina^{T} h1^{T}	AF030436.1
Methylarcula terricola h37^{T}	AF030437.1
Nautella italica^{T} 11^{T}	AM904562.1
Nereida ignava^{T} 2SM4^{T}	AJ748748.1
Oceanibulbus indoliflex^{T} HEL-45^{T}	ABID01000001.1
Oceanicella actignis^{T} PRQ-67^{T}	JQ864435.1
Oceanicola batsensis HTCC 2597^{T}	AY424898.1
Oceanicola granulosus^{T} HTCC 2516^{T}	AY424896.1
Oceanicola litoreus M-M22^{T}	JX291104.1
Oceanicola marinus AZO-C^{T}	DQ822569.1
Oceanicola nitratireducens JLT1210^{T}	EU581832.1
Octadecabacter antarcticus 307^{T}	U14583.1
Octadecabacter arcticus^{T} 238^{T}	U73725.1
Pacificibacter maritimus^{T} D1^{T}	AB558927.1
Palleronia marisminoris^{T} B33^{T}	AY926462.1
Paracoccus alkenifer A901	Y13827.1
Paracoccus aminophilus DM-15^{T}	AY014176.1
Paracoccus bengalense JJJ^{T}	AJ864469.1
Paracoccus denitrificans^{T} 381^{T}	X69159.1
Paracoccus homiensis DD-R11^{T}	DQ342239.1
Pararhodobacter aggregans^{T} D1-19^{T}	AM403160.1
Pelagibaca bermudensis^{T} HTCC2601^{T}	DQ178660.1
Pelagicola litoralis^{T} CL-ES2^{T}	EF192392.2
Pelagicola litorisediminis D1-W8^{T}	KC708867.1
Pelagimonas varians^{T} SH4-1^{T}	FJ882053.1
Phaeobacter arcticus 20188^{T}	DQ514304.1
Phaeobacter caeruleus 13^{T}	AM943630.1
Phaeobacter daeponensis TF-218^{T}	DQ981486.1
Phaeobacter gallaeciensis^{T} BS107^{T}	Y13244.1
Phaeobacter inhibens T5^{T}	AY177712.2
Phaeobacter leonis 306^{T}	HE661585.1
Piezobacter thermophilus^{T} 108^{T}	AB468958.1
Planktotalea frisia^{T} SH6-1^{T}	FJ882052.1
Pleomorphobacterium xiamenense^{T} CLW^{T}	HQ709062.1
Pontibaca methylaminivorans^{T} GRP21^{T}	AJ505788.2
Ponticoccus litoralis^{T} CL-GR66^{T}	EF211829.3
Poseidonocella pacifica^{T} Sd3-10^{T}	AB576005.1
Poseidonocella sedimentorum Sd3-23^{T}	AB576006.1
Primorskyibacter sedentarius^{T} Sd3-18^{T}	AB550558.1
Profundibacterium mesophilum^{T} KAUST-100406-0324^{T}	JF776971.1
Pseudorhodobacter antarcticus ZS3-33^{T}	FJ196030.1
Pseudorhodobacter aquimaris HDW-19^{T}	GU086365.1
Pseudorhodobacter ferrugineus^{T} A7^{T}	D88522.1
Pseudorhodobacter wandonensis WT-MW11^{T}	JN247434.1

Pseudoruegeria aquimaris^{T} SW-255^{T}	DQ675021.1
Pseudoruegeria haliotis WM67^{T}	KC196070.1
Pseudoruegeria lutimaris HD-43^{T}	FJ374173.1
Rhodobaca barguzinensis VKM B-2406^{T}	EF554833.1
Rhodobaca bogoriensis^{T} LBB1^{T}	AF248638.1
Rhodobacter blasticus ATCC 33485^{T}	DQ342322.1
Rhodobacter capsulatus^{T} C10^{T}	DQ342320.1
Rhodobacter megalophilus JA194^{T}	AM421024.1
Rhodobacter sphaeroides 2.4.1^{T}	D16425.1
Rhodobacter veldkampii 51^{T}	D16421.1
Rhodovulum adriaticum JA245	AM696698.1
Rhodovulum iodosum N1^{T}	Y15011.1
Rhodovulum marinum JA202	AM696693.1
Rhodovulum robiginosum N2^{T}	Y15012.1
Rhodovulum strictum JCM 9221	AB079635.2
Rhodovulum sulfidophilum^{T} W4^{T}	D16423.1
Roseibaca ekhonensis^{T} EL-50^{T}	AJ605746.2
Roseibacterium elongatum^{T} OCh 323^{T}	AB061273.1
Roseicitreum antarcticum^{T} ZS2-28^{T}	FJ196006.1
Roseicyclus mahoneyensis^{T} ML6^{T}	AJ315682.2
Roseinatronobacter monicus ROS 10	DQ659237.1
Roseinatronobacter thiooxidans^{T} ALG 1^{T}	AF249749.1
Roseisalinus antarcticus^{T} EL-88^{T}	AJ605747.2
Roseivivax halodurans^{T} OCh 239^{T}	D85829.1
Roseivivax halotolerans OCh 210^{T}	D85831.1
Roseivivax isoporae sw-2^{T}	FJ593905.2
Roseivivax lentus S5-5^{T}	FJ875966.1
Roseivivax pacificus 22DY03^{T}	KC018453.1
Roseivivax sediminis YIM D21^{T}	HQ615878.1
Roseobacter denitrificans OCh 114^{T}	M59063.1
Roseobacter litoralis^{T} Och 149^{T}	X78312.1
Roseovarius aestuarii SMK-122^{T}	EU156066.1
Roseovarius halocynthiae MA1-10^{T}	HQ852039.1
Roseovarius mucosus 253-13	AJ294351.1
Roseovarius nubinhibens ISM^{T}	AF098495.1
Roseovarius tolerans^{T} EL-172^{T}	Y11551.1
Rubellimicrobium aerolatum 5715S-9^{T}	AB682444.1
Rubellimicrobium mesophilum MSL-20^{T}	AOSK01000116.1
Rubellimicrobium roseum YIM 48858^{T}	GU109478.1
Rubellimicrobium thermophilum^{T} C-lvk-R2A-2^{T}	AJ844281.1
Rubribacterium polymorphum^{T} Green^{T}	EU857676.1
Rubrimonas cliftonensis^{T} OCh317^{T}	D85834.1
Ruegeria atlantica^{T} 1480^{T}	AB255399.1
Ruegeria halocynthiae MA1-6^{T}	HQ852038.1
Ruegeria lacuscaerulensis^{T} ITI-1157^{T}	U77644.1
Ruegeria marina ZH17^{T}	FJ872535.1
Ruegeria mobilis MBIC01146^{T}	AB255401.1
Ruegeria pomeroyi DSS-10	AF434674.2
Ruegeria scottomollicae 4EP29^{T}	AM905330.1
Sagittula marina F028-2^{T}	HQ336489.1
Sagittula stellata^{T} E-37^{T}	U58356.1
Salinihabitans flavidus^{T} ISL-46^{T}	FJ265707.1
Salipiger mucosus^{T} A3^{T}	AY527274.1
Sediminimonas qiaohouensis^{T} YIM B024^{T}	EU878003.1
Seohaeicola saemankumensis^{T} SD-15^{T}	EU221274.1
Shimia biformata CC-AMW-C^{T}	KC169813.1

	Shimia haliotis WM35^{T}	KC196071.1
	Shimia isoporae SW-6^{T}	FJ976449.2
	Shimia marina^{T} CL-TA03^{T}	AY962292.1
	Sulfitobacter delicatus Ivanova 022-2-77^{T}	AY180103.1
	Sulfitobacter donghicola DSW-25^{T}	EF202614.1
	Sulfitobacter dubius Ivanova Z218^{T}	AY180102.1
	Sulfitobacter marinus SW-265^{T}	DQ683726.1
	Sulfitobacter pontiacus^{T} ChLG-10^{T}	Y13155.1
	Tabrizicola aquatica^{T} RCRI19^{T}	HQ392507.3
	Tateyamaria omphalii^{T} MKT107^{T}	AB193438.1
	Tateyamaria pelophila SAM4^{T}	AJ968651.1
	Tetracoccus cechii^{T} Czech^{T}	Y09610.1
	Thalassobacter stenotrophicus^{T} 5SM22^{T}	AJ631302.1
	Thalassobius aestuarii TF-212	DQ535898.1
	Thalassobius gelatinovorus B6^{T}	D88523.1
	Thalassobius maritimus GSW-6^{T}	HM748766.1
	Thalassobius mediterraneus^{T} XSM19^{T}	AJ878874.1
	Thalassococcus halodurans^{T} UST050418-052^{T}	DQ397336.1
	Thalassococcus lentus YCS-24^{T}	JX090308.2
	Thioclava dalianensis DLFJ1-1^{T}	JQ844756.1
	Thioclava pacifica^{T} TL 2^{T}	AY656719.1
	Tranquillimonas alkanivorans^{T} A34^{T}	AB302386.1
	Tropicibacter multivorans MD5^{T}	FR727679.1
	Tropicibacter naphthalenivorans^{T} C02^{T}	AB302370.1
	Tropicibacter phthalicus KU27E1^{T}	AB636139.1
	Tropicimonas aquimaris DPG-21^{T}	HQ340608.1
	Tropicimonas isoalkanivorans^{T} B51^{T}	AB302379.1
	Tropicimonas sediminicola M97^{T}	JF748735.1
	Vadicella arenosi^{T} Sd3-24^{T}	AB564595.1
	Wenxinia marina^{T} HY34^{T}	DQ640643.1
	Yangia pacifica^{T} DX5-10^{T}	AJ877265.1
Hyphomonadaceae	*Algimonas ampicilliniresistens* 14A-2-7^{T}	AB795010.1
	Algimonas porphyrae^{T} 0C-2-2^{T}	AB689189.1
	Glycocaulis abyssi^{T} MCS33^{T}	AJ227811.1
	Hellea balneolensis^{T} 26III	AY576758.1
	Henriciella aquimarina P38^{T}	EU819081.1
	Henriciella litoralis SD10^{T}	FJ230835.1
	Henriciella marina^{T} Iso4^{T}	EF660760.1
	Hirschia baltica^{T} DSM 5838^{T}	AJ421782.1
	Hirschia litorea M-M23^{T}	JQ995780.1
	Hirschia maritima GSW-2^{T}	ARKC01000008.1
	Hyphomonas adhaerens MHS-3^{T}	AF082790.1
	Hyphomonas hirschiana VP-5^{T}	AF082794.1
	Hyphomonas jannaschiana VP2^{T}	AJ227814.1
	Hyphomonas oceanitis SCH-89^{T}	AF082797.1
	Hyphomonas polymorpha^{T} PS728^{T}	AJ227813.1
	Litorimonas taeanensis^{T} G5^{T}	FJ230838.1
	Maricaulis maris^{T} CM 11^{T}	AJ227802.1
	Maricaulis parjimensis MCS 25^{T}	AJ227808.2
	Maricaulis salignorans MCS 18^{T}	AJ227806.1
	Maricaulis virginensis VC-5^{T}	AJ301667.1
	Maricaulis washingtonensis MCS 6^{T}	AJ227804.1
	Marinicauda pacifica^{T} p-1km-3^{T}	JQ045549.1
	Oceanicaulis alexandrii^{T} C116-18^{T}	AJ309862.1
	Oceanicaulis stylophorae GISW-4^{T}	HM035090.1
	Ponticaulis koreensis^{T} GSW-23^{T}	FM202497.1

	Robiginitomaculum antarcticum^{T} IMCC3195^{T}	EF495229.1
KORDIIMONADALES		
Kordiimonadaceae	*Eilatimonas milleporae*^{T} MD2^{T}	HQ288781.1
	Kordiimonas aestuarii 101-1^{T}	JF714701.2
	Kordiimonas aquimaris MEBiC06554^{T}	GU289640.1
	Kordiimonas gwangyangensis^{T} GW14-5^{T}	AY682384.1
	Kordiimonas lacus S3-22^{T}	FJ847942.1
Rhodothalassium family	*Rhodothalassium salexigens*^{T} WS 68^{T}	M59070.1
KILONIELLALES		
Kiloniellaceae	*Kiloniella laminariae*^{T} LD81^{T}	AM749667.1
RHODOSPIRILLALES		
Rhodospirillaceae	[*Azospirillum*] *amazonense* Am 14(Y1)^{T}	X79735.1
	Azospirillum canadense DS2^{T}	DQ393891.1
	Azospirillum fermentarium CC-LY743^{T}	JX843282.1
	Azospirillum formosense CC-Nfb-7^{T}	GU256444.1
	Azospirillum lipoferum^{T} 59b^{T}	Z29619.1
	Caenispirillum bisanense^{T} K92^{T}	EF100694.1
	Caenispirillum sp. AK4^{T}	FN995238.2
	Constrictibacter antarcticus^{T} 262-8^{T}	AB510913.1
	Defluviicoccus vanus^{T} Ben 114^{T}	AF179678.1
	Desertibacter roseus^{T} 2622^{T}	EU833987.1
	Fodinicurvata fenggangensis YIM D812^{T}	FJ357427.1
	Fodinicurvata sediminis^{T} YIM D82^{T}	FJ357426.1
	Inquilinus ginsengisoli Gsoil 080^{T}	AB245352.1
	Inquilinus limosus^{T} AU476^{T}	AY043374.1
	Insolitispirillum peregrinum^{T} LMG 4340^{T}	EF612767.1
	Limimonas halophila^{T} Ia16^{T}	JN605361.1
	Magnetospira thiophila^{T} MMS-1^{T}	EU861390.1
	Magnetospirillum gryphiswaldense^{T} MSR-1^{T}	Y10109.1
	Magnetospirillum magnetotacticum MS-1^{T}	Y10110.1
	Magnetovibrio blakemorei^{T} MV-1^{T}	L06455.1
	Marispirillum indicum^{T} B142^{T}	EU642410.1
	Novispirillum itersonii^{T} Giesberger^{T}	EF612765.1
	Pelagibius litoralis^{T} CL-UU02^{T}	DQ401091.1
	Phaeospirillum chandramohanii JA145^{T}	AM779061.1
	Phaeospirillum cystidoformans JA317^{T}	AM901294.1
	Phaeospirillum fulvum^{T} KK^{T}	D14433.1
	Phaeospirillum molischianum 5^{T}	FR733695.1
	Phaeospirillum tilakii JA492^{T}	FN675262.1
	Phaeovibrio sulfidiphilus^{T} JA480^{T}	FN391894.1
	Rhodocista centenaria^{T} SW	NR_074105.1
	Rhodocista pekingensis JA405	FM177580.1
	Rhodospira trueperi^{T} 8316^{T}	X99671.1
	Rhodospirillum oryzae JA318^{T}	AM901295.1
	Rhodospirillum photometricum DSM 122^{T}	AJ222662.1
	Rhodospirillum rubrum^{T} S.1^{T}	NR_074249.1
	Rhodospirillum sulfurexigens JA143^{T}	AM710622.1
	Rhodovibrio salinarum^{T} NCIMB 2243^{T}	D14432.1
	Rhodovibrio sodomensis DSI^{T}	FR733704.1
	Roseospira goensis JA135^{T}	AM283537.1
	Roseospira marina CE2105^{T}	AJ298879.1
	Roseospira mediosalina^{T} L1-66^{T}	AJ000989.1
	Roseospira navarrensis SE3104^{T}	AJ298880.1
	Roseospira visakhapatnamensis JA131^{T}	AM282560.1
	Roseospirillum parvum^{T} 930I^{T}	AJ011919.1
	Skermanella aerolata B26	JN377672.1

	Skermanella parooensis^{T} ACM 2042^{T}	X90760.1
	Skermanella stibiiresistens SB22^{T}	HQ315828.1
	Skermanella xinjiangensis 10-1-101^{T}	EU586202.1
	Telmatospirillum siberiense^{T} 26-4b1^{T}	AF524863.1
	Terasakiella pusilla^{T} IF6^{T}	AB006768.1
	Thalassospira alkalitolerans MBE#61^{T}	AB786710.1
	Thalassospira lucentensis^{T} VBW014	KC534149.1
	Thalassospira mesophila MBE#74^{T}	AB786711.1
	Thalassospira profundimaris WP0211^{T}	AY186195.1
	Thalassospira tepidiphila 1-1B^{T}	AB265822.1
	Tistlia consotensis^{T} USBA 355^{T}	CBKU010000188.1
Zavarzinia family	*Oleomonas sagaranensis*^{T} AT18^{T}	AB677960.1
	Zavarzinia compransoris^{T} Z-1155^{T}	JX986958.1
Dongia family	*Dongia mobilis*^{T} LM22^{T}	FJ455532.1
Thalassobaculum family	*Nisaea denitrificans*^{T} DR41-21^{T}	AUFM01000008.1
	Nisaea nitritireducens DR41-18^{T}	DQ665839.1
	Oceanibaculum indicum^{T} P24^{T}	EU656113.1
	Oceanibaculum pacificum MC2UP-L3^{T}	FJ463255.1
	Thalassobaculum litoreum^{T} CL-GR58^{T}	EF203900.2
	Thalassobaculum salexigens CZ41 10a^{T}	AUIR01000011.1
Ferrovibrio family	*Ferrovibrio denitrificans*^{T} Sp-1^{T}	GQ365620.1
	Oceanibacterium hippocampi^{T} BFLP-8^{T}	FN687912.1
Stella family	*Stella humosa*^{T} DSM 5900^{T}	AJ535710.1
	Stella vacuolata DSM 5901^{T}	AJ535711.1
Tistrella family	*Tistrella bauzanensis* BZ78^{T}	GQ240228.1
	Tistrella mobilis^{T} TISTR 1108^{T}	AB071665.1
Reyranella family	*Reyranella massiliensis*^{T} 521^{T}	EF394922.1
	Reyranella soli KIS14-15^{T}	JX260424.1
Acetobacteraceae	*Acetobacter aceti*^{T} AS 1.1809^{T}	X74066.1
	Acetobacter cerevisiae LMG 1625^{T}	AJ419843.1
	Acetobacter fabarum R-36330^{T}	AM905849.1
	Acetobacter indonesiensis 5H-1^{T}	AB032356.1
	Acetobacter nitrogenifigens RG1^{T}	AY669513.2
	Acidicaldus organivorans^{T} Y008^{T}	AY140238.1
	Acidiphilium acidophilum MS Silver^{T}	D86511.1
	Acidiphilium angustum KLB^{T}	D30772.1
	Acidiphilium cryptum^{T} Lhet2^{T}	D30773.1
	Acidiphilium multivorum AIU 301^{T}	AB006711.1
	Acidiphilium rubrum OP^{T}	D30776.1
	Acidisoma sibiricum TPB606^{T}	AM947653.1
	Acidisoma tundrae^{T} WM1^{T}	AM947652.1
	Acidisphaera rubrifaciens^{T} HS-AP3^{T}	D86512.1
	Acidocella aluminiidurans AL46^{T}	AB362219.2
	Acidocella aminolytica 101^{T}	D30771.1
	Acidocella facilis^{T} PW2^{T}	D30774.1
	Acidomonas methanolica^{T} MB58^{T}	X77468.1
	Ameyamaea chiangmaiensis^{T} AC04^{T}	AB303366.1
	Asaia astilbes T-6133^{T}	AB485740.1
	Asaia bogorensis^{T} 71^{T}	AB025928.1
	Asaia lannensis AB92^{T}	AB286050.1
	Asaia platycodi T-683^{T}	AB485739.1
	Asaia spathodeae GB23-2^{T}	AB511277.2
	Belnapia moabensis^{T} CP2CT^{T}	AJ871428.1
	Belnapia rosea CPCC 100156^{T}	HQ641379.1
	Belnapia soli PB-K8^{T}	JN171665.1
	Commensalibacter intestini^{T} A911^{T}	EU409601.1

	Craurococcus roseus^{T} NS130^{T}	D85828.1
	Elioraea tepidiphila^{T} TU-7^{T}	EF519867.1
	Endobacter medicaginis^{T} M1MS02^{T}	JQ436923.1
	Gluconacetobacter asukensis K8617-1-1b^{T}	AB627120.1
	Gluconacetobacter azotocaptans CFN-Ca54^{T}	AF192761.1
	Gluconacetobacter johannae CFN-Cf55^{T}	AF111841.1
	Gluconacetobacter kakiaceti G5-1^{T}	AB607833.1
	Gluconacetobacter liquefaciens^{T} AsaiG-1^{T}	X75617.1
	Gluconobacter kanchanaburiensis AD92^{T}	AB459530.1
	Gluconobacter nephelii RBY-1^{T}	AB540148.2
	Gluconobacter oxydans^{T} 1^{T}	X73820.1
	Gluconobacter uchimurae zw160-2^{T}	AB193244.1
	Gluconobacter wancherniae AC42^{T}	AB511060.1
	Granulibacter bethesdensis^{T} CGDNIH1^{T}	AY788950.1
	Humitalea rosea^{T} W37^{T}	HQ882802.1
	Komagataeibacter hansenii AS-1.1811^{T}	X75620.2
	Komagataeibacter intermedius TF2^{T}	Y14694.1
	Komagataeibacter kombuchae RG3^{T}	AY688433.2
	Komagataeibacter nataicola 81 Nata Organism^{T}	AB166743.1
	Komagataeibacter xylinus^{T} Bertrand^{T}	X75619.1
	Kozakia baliensis^{T} Yo-3^{T}	AB056321.1
	Neoasaia chiangmaiensis^{T} AC28^{T}	AB208549.1
	Neokomagataea tanensis AH13^{T}	AB513364.1
	Neokomagataea thailandica^{T} AH11^{T}	AB513363.1
	Paracraurococcus ruber^{T} NS89^{T}	D85827.1
	Rhodopila globiformis^{T} 7950^{T}	D86513.1
	Rhodovarius lipocyclicus^{T} 3187^{T}	AJ633644.1
	Rhodovastum atsumiense^{T} G2-11^{T}	AB381935.1
	Roseococcus suduntuyensis SHET^{T}	EU012448.1
	Roseococcus thiosulatophilus^{T} RB-3^{T}	X72908.1
	Roseomonas aerilata 5420S-30^{T}	AB682446.1
	Roseomonas aestuarii JC17^{T}	AB682256.1
	Roseomonas frigidaquae CW67^{T}	EU290160.2
	Roseomonas gilardii^{T} 5424^{T}	AY150045.1
	Roseomonas terrae DS-48^{T}	EF363716.1
	Rubritepida flocculans^{T} H-8^{T}	AF465832.1
	Saccharibacter floricola^{T} S-877^{T}	AB110421.1
	Swaminathania salitolerans^{T} PA51^{T}	AF459454.1
	Tanticharoenia sakaeratensis^{T} AC37^{T}	AB304087.1
Elstera family	*Elstera litoralis*^{T} Dia-1^{T}	EU678309.1
Alysiosphaera family	"*Candidatus* Alysiosphaera europeae"	AY428766.1
	Geminicoccus roseus^{T} D2-3T^{T}	AM403172.1
SNEATHIELLALES		
Sneathiellaceae	*Sneathiella chinensis*^{T} CBMAI 737^{T}	DQ219355.2
	Sneathiella glossodoripedis MKT133^{T}	AB289439.1
PARVULARCULALES		
Parvularculaceae	*Parvularcula bermudensis* HTCC 2503^{T}	NR_074158.1
	Parvularcula dongshanensis SH25^{T}	JQ778314.1
	Parvularcula lutaonensis CC-MMS-1^{T}	EU346850.1
SPHINGOMONADALES		
Sphingomonadaceae	*Blastomonas natatoria*^{T} R-14558^{T}	AB024288.1
	Hephaestia caeni^{T} ERB1-3^{T}	FJ948169.1
	Novosphingobium acidiphilum FSW06-204d^{T}	EU336977.1
	Novosphingobium aquaticum FNE08-86^{T}	JN399173.1
	Novosphingobium barchaimii LL02^{T}	JN695619.1
	Novosphingobium capsulatum^{T} 28^{T}	D16147.1

	Novosphingobium fuchskuhlense FNE08-7^{T}	JN399172.1
	Novosphingobium nitrogenifigens Y88^{T}	DQ448852.1
	Parasphingopyxis lamellibrachiae^{T} JAMH 0132^{T}	AB524074.1
	Sandaracinobacter sibiricus^{T} RB16-17^{T}	Y10678.1
	Sandarakinorhabdus limnophila^{T} so42^{T}	AY902680.1
	Sphingobium abikonense IAM 12404^{T}	AB021416.1
	Sphingobium amiense YT^{T}	AB047364.1
	Sphingobium baderi LL03^{T}	JN695620.1
	Sphingobium boeckii 469^{T}	JN591315.1
	Sphingobium yanoikuyae^{T} AB 1105^{T}	AGZU01000016.1
	Sphingomicrobium astaxanthinifaciens CC-AMO-30B^{T}	JX235675.1
	Sphingomicrobium flavum CC-AMZ-30N^{T}	JX393854.1
	Sphingomicrobium lutaoense^{T} CC-TBT-3^{T}	EU564841.1
	Sphingomicrobium marinum CC-AMZ-30M^{T}	JX235672.1
	Sphingomonas abaci C42^{T}	AJ575817.1
	Sphingomonas aerolata NW12^{T}	AJ429240.1
	Sphingomonas asaccharolytica Y-345^{T}	Y09639.1
	Sphingomonas changbaiensis V2M44^{T}	EU682685.1
	Sphingomonas paucimobilis^{T} Allen^{T}	D16144.1
	Sphingopyxis alaskensis RB2256^{T}	Z73631.1
	Sphingopyxis bauzanensis BZ30^{T}	GQ131578.1
	Sphingopyxis chilensis S37^{T}	AF367204.1
	Sphingopyxis granuli Kw07^{T}	AY563034.1
	Sphingopyxis macrogoltabida^{T} 203^{T}	D13723.1
	Sphingorhabdus flavimaris SW-151^{T}	AY554010.1
	Sphingorhabdus litoris FR1093^{T}	DQ781321.2
	Sphingorhabdus marina FR1087^{T}	DQ781320.2
	Sphingorhabdus planktonica^{T} 585^{T}	JN381068.1
	Sphingosinicella microcystinivorans^{T} Y2^{T}	AB084247.1
	Sphingosinicella soli KSL-125^{T}	DQ087403.1
	Sphingosinicella vermicomposti YC7378^{T}	FJ442859.2
	Sphingosinicella xenopeptidilytica 3-2W4^{T}	AY950663.2
	Stakelama pacifica^{T} JLT832^{T}	EU581829.1
	Stakelama sediminis CJ70^{T}	EU099873.2
	Zymomonas mobilis francensis AN0101^{T}	FR749909.1
	Zymomonas mobilis mobilis^{T} ATCC 10988^{T}	AF281031.1
	Zymomonas mobilis pomaceae ZanA^{T}	AF281032.1
Erythrobacteraceae	*Altererythrobacter aestuarii* KYW147^{T}	FJ997597.1
	Altererythrobacter dongtanensis JM27^{T}	GU166344.1
	Altererythrobacter epoxidivorans^{T} JCS350^{T}	DQ304436.1
	Altererythrobacter gangjinensis KJ7^{T}	JF751048.2
	Altererythrobacter ishigakiensis NITE-AP48^{T}	AB363004.1
	Citromicrobium bathoceanense^{T} JF-1^{T}	Y16267.1
	Citromicrobium bathyomarinum JL354^{T}	DQ104408.1
	Croceicoccus marinus^{T} E4A9^{T}	EF623998.1
	Erythrobacter aquimaris SW-140	AY461443.1
	Erythrobacter citreus RE35F	AF118020.1
	Erythrobacter flavus SW-46^{T}	AF500004.1
	Erythrobacter gaetbuli SW-161^{T}	AY562220.1
	Erythrobacter longus^{T} OCh101^{T}	AF465835.1
	Lutibacterium anuloederans^{T} LC8^{T}	AY026916.1
	Porphyrobacter cryptus ALC-2^{T}	AF465834.1
	Porphyrobacter dokdonensis DSW-74^{T}	DQ011529.1
	Porphyrobacter donghaensis SW-132^{T}	AY559428.1
	Porphyrobacter neustonensis^{T} DSM 9434^{T}	AB033327.1
	Porphyrobacter sanguineus A91^{T}	AB021493.1

	Porphyrobacter tepidarius OT3^{T}	AB033328.1
MICAVIBRIO order		
Micavibrio family	*Micavibrio aeruginosavorus* ARL-13	DQ186612.1
RICKETTSIALES		
Rickettsiaceae	*Orientia chuto* Dubai^{T}	HM852447.1
	Orientia tsutsugamushi^{T} Karp^{T}	D38623.1
	Rickettsia aeschlimanni Mc16^{T}	U74757.1
	Rickettsia bellii 369L42-1^{T}	L36103.1
	Rickettsia montanensis Tick^{T}	L36215.1
	Rickettsia prowazekii^{T} Brein1^{T}	M21789.1
	Rickettsia rickettsii R^{T}	L36217.1
	Rickettsia tamurae AT-1^{T}	AY049981.1
Anaplasmataceae	*Anaplasma marginale*^{T} Virginia	AF309866.1
	Anaplasma phagocytophilum Webster^{T}	U02521.1
	Ehrlichia canis^{T} TWN1	EU106856
	Ehrlichia chaffeensis Arkansas^{T}	M73222.1
	Ehrlichia ewingii Stillwater^{T}	M73227.1
	Ehrlichia muris AS145^{T}	U15527.1
	Ehrlichia risticii Illinois^{T}	M21290.1
	Ehrlichia ruminantium Welgevonden^{T}	NR_074155.1
	Neorickettsia helminthoeca^{T}	ENAU12457
	Neorickettsia sennetsu Miyayama^{T}	NR_074386.1
	Wolbachia pipientis^{T} Mansonella sp.	AJ628417.1
Holosporaceae	*Holospora obtusa*	X58198.1
	Holospora undulata HU1	ARPM01000348.1
MAGNETOCOCCALES		
Magnetococcaceae	*Magnetococcus marinus*^{T} MC-1^{T}	NR_074371.1
GAMMAPROTEOBACTERIA	*Escherichia coli*^{T} K-12_MG1655 (outgroup)	NC_000913.3

Complete maximum likelihood tree showing the position of "*Ca.* Phaeomarinobacter ectocarpi" Ec32 within the *Alphaproteobacteria*. Hyper-variable regions were masked from the alignment. The *Gammaproteobacterium Escherichia coli* was used as outgroup.

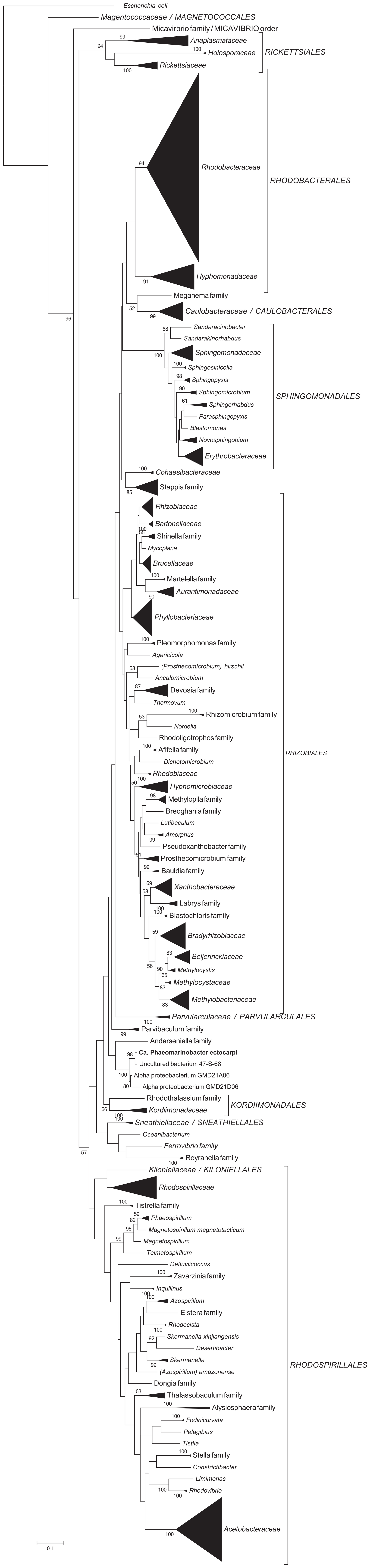

Supplement: Data sheet 1 — 16S rRNA sequences and complete phylogenetic tree. The superscript letter T in the “strain” column indicates type species and type strains, respectively. The genera included between brackets means that the strains are probably misnamed. The taxonomic classification used was obtained from the EzTaxon server (http://www.ezbiocloud.net/eztaxon). The maximum likelihood tree showing the position of “Ca. Phaeomarinobacter ectocarpi” Ec32 within the Alphaproteobacteria was based on an alignment with masked hyper-variable regions. The Gammaproteobacterium Escherichia coli was used as outgroup. [file DataSheet1.PDF]
