# Supplementary material for: Genome and metabolic network of “Candidatus Phaeomarinobacter ectocarpi” Ec32, a new candidate genus of Alphaproteobacteria frequently associated with brown algae
Source: Front Genet. 2014 Jul 25;5:241. doi: 10.3389/fgene.2014.00241 (PMC4110880; doi:10.3389/fgene.2014.00241)
Supplement: Data sheet 2 — Functional comparison of the “Ca. Phaeomarinobacter ectocarpi” Ec32 and of the Parvibaculum lavamentivorans DS-1T genomes carried out using the RAST platform. [file DataSheet2.PDF]

Data sheet 2. Functional comparison of the "*Ca.* P. ectocarpi" Ec32 and the *P. lavamentivorans* DS-1^T genomes carried out using the RAST platform.

Category	Subcategory	Subsystem	Role
**"*Ca.* P. ectocarpi" only**			
Amino Acids and Derivatives	Alanine, serine, and glycine	Alanine biosynthesis	Valine--pyruvate aminotransferase (EC 2.6.1.66)
Amino Acids and Derivatives	Alanine, serine, and glycine	Glycine cleavage system	Sodium/glycine symporter GlyP
Amino Acids and Derivatives	Arginine; urea cycle, polyamines	Arginine and Ornithine Degradation	Ornithine cyclodeaminase (EC 4.3.1.12)
Amino Acids and Derivatives	Arginine; urea cycle, polyamines	Polyamine Metabolism	Putrescine transport ATP-binding protein PotA (TC 3.A.1.11.1)
Amino Acids and Derivatives	Branched-chain amino acids	Branched-Chain Amino Acid Biosynthesis	Leucine-responsive regulatory protein, regulator for leucine (or lrp) regulon and high-affinity branched-chain amino acid transport system
Amino Acids and Derivatives	Glutamine, glutamate, aspartate, asparagine; ammonia assimilation	Glutamate and Aspartate uptake in Bacteria	Glutamate Aspartate periplasmic binding protein precursor GltI (TC 3.A.1.3.4)
Amino Acids and Derivatives	Glutamine, glutamate, aspartate, asparagine; ammonia assimilation	Glutamate and Aspartate uptake in Bacteria	Glutamate Aspartate transport system permease protein GltJ (TC 3.A.1.3.4)
Amino Acids and Derivatives	Glutamine, glutamate, aspartate, asparagine; ammonia assimilation	Glutamate and Aspartate uptake in Bacteria	Glutamate Aspartate transport system permease protein GltK (TC 3.A.1.3.4)
Amino Acids and Derivatives	Glutamine, glutamate, aspartate, asparagine; ammonia assimilation	Glutamate and Aspartate uptake in Bacteria	Sodium/glutamate symport protein
Amino Acids and Derivatives	Lysine, threonine, methionine, and cysteine	Methionine Biosynthesis	Methionine ABC transporter ATP-binding protein
Carbohydrates	Central carbohydrate metabolism	Glycolysis and Gluconeogenesis	Fructose-bisphosphate aldolase class II (EC 4.1.2.13)
Carbohydrates	Central carbohydrate metabolism	Glycolysis and Gluconeogenesis	Phosphoenolpyruvate synthase (EC 2.7.9.2)
Carbohydrates	Central carbohydrate metabolism	Glyoxylate bypass	Malate synthase G (EC 2.3.3.9)
Carbohydrates	Central carbohydrate metabolism	Pentose phosphate pathway	Transketolase, C-terminal section (EC 2.2.1.1)
Carbohydrates	Central carbohydrate metabolism	Pentose phosphate pathway	Transketolase, N-terminal section (EC 2.2.1.1)
Carbohydrates	Central carbohydrate metabolism	Pyruvate Alanine Serine Interconversions	Omega-amino acid--pyruvate aminotransferase (EC 2.6.1.18)
Carbohydrates	CO2 fixation	Photorespiration (oxidative C2 cycle)	Malyl-CoA lyase (EC 4.1.3.24)
Carbohydrates	Monosaccharides	D-ribose utilization	Ribose 5-phosphate isomerase B (EC 5.3.1.6)
Carbohydrates	no subcategory	VC0266	Hypothetical protein VC0266 (sugar utilization related?)
Carbohydrates	Organic acids	Glycerate metabolism	D-glycerate 3-kinase (EC 2.7.1.31), plant type
Carbohydrates	Organic acids	Lactate utilization	Predicted L-lactate dehydrogenase, Fe-S oxidoreductase subunit YkgE
Carbohydrates	Organic acids	Lactate utilization	Predicted L-lactate dehydrogenase, Iron-sulfur cluster-binding subunit YkgF
Carbohydrates	Organic acids	Lactate utilization	Predicted L-lactate dehydrogenase, hypothetical protein subunit YkgG
Carbohydrates	Organic acids	Methylcitrate cycle	2-methylcitrate dehydratase (EC 4.2.1.79)
Carbohydrates	Organic acids	Methylcitrate cycle	2-methylcitrate synthase (EC 2.3.3.5)
Carbohydrates	Organic acids	Methylcitrate cycle	Methylisocitrate lyase (EC 4.1.3.30)
Cell Wall and Capsule	Capsular and extracellular polysacchrides	Capsular heptose biosynthesis	D-glycero-D-manno-heptose 1-phosphate guanosyltransferase
Cell Wall and Capsule	Capsular and extracellular polysacchrides	Capsular heptose biosynthesis	Phosphoheptose isomerase (EC 5.3.1.-)
Cell Wall and Capsule	Capsular and extracellular polysacchrides	CMP-N-acetylneuraminate Biosynthesis	N-acetylneuraminate synthase (EC 2.5.1.56)
Cell Wall and Capsule	Capsular and extracellular polysacchrides	CMP-N-acetylneuraminate Biosynthesis	UDP-N-acetylglucosamine 2-epimerase (EC 5.1.3.14)
Cell Wall and Capsule	Capsular and extracellular polysacchrides	Exopolysaccharide Biosynthesis	Glycosyl transferase, group 1 family protein
Cell Wall and Capsule	Capsular and extracellular polysacchrides	Exopolysaccharide Biosynthesis	Tyrosine-protein kinase EpsD (EC 2.7.10.2)
Cell Wall and Capsule	Capsular and extracellular polysacchrides	Exopolysaccharide Biosynthesis	Undecaprenyl-phosphate galactosephosphotransferase (EC 2.7.8.6)
Cell Wall and Capsule	Capsular and extracellular polysacchrides	Legionaminic Acid Biosynthesis	Bacillosamine/Legionaminic acid biosynthesis aminotransferase PglE
Cell Wall and Capsule	Capsular and extracellular polysacchrides	Legionaminic Acid Biosynthesis	Legionaminic acid cytidylyltransferase (EC 2.7.7.43)
Cell Wall and Capsule	Capsular and extracellular polysacchrides	Rhamnose containing glycans	Teichoic acid export ATP-binding protein TagH (EC 3.6.3.40)
Cell Wall and Capsule	Gram-Negative cell wall components	Lipoprotein sorting system	Lipoprotein releasing system transmembrane protein LolC
Cell Wall and Capsule	Gram-Negative cell wall components	LOS core oligosaccharide biosynthesis	ADP-L-glycero-D-manno-heptose-6-epimerase (EC 5.1.3.20)
Cell Wall and Capsule	Gram-Negative cell wall components	LOS core oligosaccharide biosynthesis	UDP-galactopyranose mutase (EC 5.4.99.9)
Clustering-based subsystems	DNA metabolism	CBSS-272943.3.peg.263	FIG056333: sensor
Clustering-based subsystems	Flagella protein?	CBSS-323098.3.peg.2823	MotA/TolQ/ExbB proton channel family protein, probably associated with flagella
Clustering-based subsystems	May be related to ADP-phosphoribose and NAD-dependent acetylation	CBSS-216591.1.peg.168	Glutathione S-transferase N-terminal domain protein (EC 2.5.1.18)
Clustering-based subsystems	May be related to ADP-phosphoribose and NAD-dependent acetylation	CBSS-216591.1.peg.168	MaoC domain protein dehydratase
Clustering-based subsystems	May be related to ADP-phosphoribose and NAD-dependent acetylation	CBSS-216591.1.peg.168	Predicted aminoglycoside phosphotransferase
Clustering-based subsystems	no subcategory	A predicted deacylase that co-occurs with Ribosomal protein S6 glutaminyl transferase	FIG003737: Predicted deacylase
Clustering-based subsystems	no subcategory	A predicted deacylase that co-occurs with Ribosomal protein S6 glutaminyl transferase	Ribosomal protein S6 glutaminyl transferase
Clustering-based subsystems	no subcategory	A predicted deacylase that co-occurs with Ribosomal protein S6 glutaminyl transferase	hypothetical protein sometimes fused to ribosomal protein S6 glutaminyl transferase
Clustering-based subsystems	no subcategory	Bacterial RNA-metabolizing Zn-dependent hydrolases	Ferric uptake regulation protein FUR
Clustering-based subsystems	no subcategory	CBSS-211586.1.peg.2352	FIG005548: transport protein
Clustering-based subsystems	no subcategory	CBSS-211586.9.peg.2560	Acetate permease ActP (cation/acetate symporter)
Clustering-based subsystems	no subcategory	CBSS-211586.9.peg.2560	FIG152265: Sodium:solute symporter associated protein
Clustering-based subsystems	no subcategory	CBSS-296591.1.peg.2330	Alpha-1,3-N-acetylgalactosamine transferase PglA (EC 2.4.1.-)
Clustering-based subsystems	no subcategory	CBSS-296591.1.peg.2330	Lipid carrier : UDP-N-acetylgalactosaminyltransferase (EC 2.4.1.-)

Clustering-based subsystems	no subcategory	CBSS-296591.1.peg.2330	Putative glycosyltransferase
Clustering-based subsystems	no subcategory	CBSS-296591.1.peg.2330	UDP-N-acetylglucosamine 4,6-dehydratase (EC 4.2.1.-)
Clustering-based subsystems	no subcategory	CBSS-314276.3.peg.1499	Ribosomal large subunit pseudouridine synthase B (EC 4.2.1.70)
Clustering-based subsystems	no subcategory	CBSS-316056.14.peg.2759	FIG007785: exported protein
Clustering-based subsystems	no subcategory	CBSS-498211.3.peg.1415	FIG021952: putative membrane protein
Clustering-based subsystems	no subcategory	CBSS-83333.1.peg.566	Bacteriophage N4 adsorption protein A
Clustering-based subsystems	no subcategory	CBSS-83333.1.peg.566	Bacteriophage N4 adsorption protein B
Clustering-based subsystems	no subcategory	CBSS-83333.1.peg.876	FIG065221: Holliday junction DNA helicase
Clustering-based subsystems	no subcategory	CBSS-87626.3.peg.3639	2-octaprenyl-6-methoxyphenol hydroxylase (EC 1.14.13.-)
Clustering-based subsystems	no subcategory	Conserved gene cluster associated with Met-tRNA formyltransferase	tRNA:m(5)U-54 MTase gid
Clustering-based subsystems	pH adaptation potassium efflux	CBSS-272569.1.peg.3198	pH adaptation potassium efflux system protein D 3
Clustering-based subsystems	Probably GTP or GMP signaling related	CBSS-176299.4.peg.1292	GTP pyrophosphokinase (EC 2.7.6.5), (p)ppGpp synthetase II
Clustering-based subsystems	Probably GTP or GMP signaling related	CBSS-176299.4.peg.1292	Signal peptidase I (EC 3.4.21.89)
Clustering-based subsystems	Probably Ybbk-related hypothetical membrane proteins	CBSS-316057.3.peg.659	Putative activity regulator of membrane protease YbbK
Clustering-based subsystems	Probably Ybbk-related hypothetical membrane proteins	CBSS-316057.3.peg.659	Putative stomatin/prohibitin-family membrane protease subunit YbbK
Clustering-based subsystems	Putative Isoquinoline 1-oxidoreductase subunit	CBSS-314267.3.peg.390	Putative Isoquinoline 1-oxidoreductase subunit, Mll3835 protein
Clustering-based subsystems	Sulfatases and sulfatase modifying factor 1 (and a hypothetical)	Sulfatases and sulfatase modifying factor 1	Sulfatase modifying factor 1 precursor (C-alpha-formyglycine- generating enzyme 1)
Cofactors, Vitamins, Prosthetic Groups, Pigments	Biotin	Biotin biosynthesis	ATPase component BioM of energizing module of biotin ECF transporter
Cofactors, Vitamins, Prosthetic Groups, Pigments	Coenzyme A	Coenzyme A Biosynthesis	Aspartate 1-decarboxylase (EC 4.1.1.11)
Cofactors, Vitamins, Prosthetic Groups, Pigments	Coenzyme A	Coenzyme A Biosynthesis	Pantothenate kinase type III, CoaX-like (EC 2.7.1.33)
Cofactors, Vitamins, Prosthetic Groups, Pigments	Folate and pterines	5-FCL-like protein	5-FCL-like protein
Cofactors, Vitamins, Prosthetic Groups, Pigments	Folate and pterines	Folate biosynthesis cluster	Alternative dihydrofolate reductase 3
Cofactors, Vitamins, Prosthetic Groups, Pigments	no subcategory	Thiamin biosynthesis	Hydroxymethylpyrimidine ABC transporter, substrate-binding component
Cofactors, Vitamins, Prosthetic Groups, Pigments	Pyridoxine	Pyridoxin (Vitamin B6) Biosynthesis	Pyridoxal kinase (EC 2.7.1.35)
DNA Metabolism	DNA repair	DNA Repair Base Excision	ATP-dependent DNA ligase (EC 6.5.1.1) LigC
DNA Metabolism	DNA repair	DNA Repair Base Excision	DNA-3-methyladenine glycosylase II (EC 3.2.2.21)
DNA Metabolism	DNA repair	DNA repair system including RecA, MutS and a hypothetical protein	Protein Implicated in DNA repair function with RecA and MutS
DNA Metabolism	DNA repair	DNA repair, bacterial	ADA regulatory protein
DNA Metabolism	no subcategory	DNA structural proteins, bacterial	DNA-binding protein HU
Fatty Acids, Lipids, and Isoprenoids	Fatty acids	Acyl-CoA thioesterase II	TesB-like acyl-CoA thioesterase 1
Fatty Acids, Lipids, and Isoprenoids	Fatty acids	Fatty Acid Biosynthesis FASII	Biotin carboxyl carrier protein
Fatty Acids, Lipids, and Isoprenoids	no subcategory	Hopanes	Hopanoid-associated RND transporter, HpnN
Fatty Acids, Lipids, and Isoprenoids	no subcategory	Hopanes	Radical SAM protein required for addition of adenosine to hopane skeleton, HpnH
Fatty Acids, Lipids, and Isoprenoids	no subcategory	Hopanes	Squalene--hopene cyclase (EC 5.4.99.17)
Fatty Acids, Lipids, and Isoprenoids	no subcategory	Hopanes	Squalene-associated FAD-dependent desaturase, HpnE
Fatty Acids, Lipids, and Isoprenoids	Triacylglycerols	Triacylglycerol metabolism	Monoglyceride lipase (EC 3.1.1.23)
Membrane Transport	ABC transporters	ABC transporter oligopeptide (TC 3.A.1.5.1)	Oligopeptide ABC transporter, periplasmic oligopeptide-binding protein OppA (TC 3.A.1.5.1)
Membrane Transport	ABC transporters	ABC transporter oligopeptide (TC 3.A.1.5.1)	Oligopeptide transport system permease protein OppB (TC 3.A.1.5.1)
Membrane Transport	ABC transporters	ABC transporter oligopeptide (TC 3.A.1.5.1)	Oligopeptide transport system permease protein OppC (TC 3.A.1.5.1)
Membrane Transport	Cation transporters	Magnesium transport	Mg(2+) transport ATPase protein C
Membrane Transport	TRAP transporters	TRAP Transporter unknown substrate 9	TRAP-type uncharacterized transport system, fused permease component
Metabolism of Aromatic Compounds	Metabolism of central aromatic intermediates	Salicylate and gentisate catabolism	Salicylate hydroxylase (EC 1.14.13.1)
Miscellaneous	no subcategory	Broadly distributed proteins not in subsystems	YpfJ protein, zinc metalloprotease superfamily
Miscellaneous	no subcategory	Phosphoglycerate mutase protein family	Phosphoglycerate mutase family 2
Miscellaneous	Plant-Prokaryote DOE project	Iron-sulfur cluster assembly	PaaD-like protein (DUF59) involved in Fe-S cluster assembly
Miscellaneous	Plant-Prokaryote DOE project	Scaffold proteins for [4Fe-4S] cluster assembly (MRP family)	Iron-sulfur cluster-binding protein
Miscellaneous	Plant-Prokaryote DOE project	Single-Rhodanese-domain proteins	Glutathione S-transferase domain protein
Miscellaneous	Plant-Prokaryote DOE project	Single-Rhodanese-domain proteins	Rhodanese domain protein UPF0176, cyanobacterial/alphaproteobacterial subgroup
Motility and Chemotaxis	Flagellar motility in Prokaryota	Flagellar motility	Flagellar biosynthesis protein FlhF
Motility and Chemotaxis	Flagellar motility in Prokaryota	Flagellar motility	Flagellar synthesis regulator FleN
Motility and Chemotaxis	no subcategory	Bacterial Chemotaxis	Chemotaxis protein CheV (EC 2.7.3.-)
Motility and Chemotaxis	no subcategory	Bacterial Chemotaxis	Chemotaxis response regulator protein-glutamate methylesterase CheB (EC 3.1.1.61)
Motility and Chemotaxis	no subcategory	Bacterial Chemotaxis	Positive regulator of CheA protein activity (CheW)
Motility and Chemotaxis	no subcategory	Bacterial Chemotaxis	Signal transduction histidine kinase CheA (EC 2.7.3.-)
Nucleosides and Nucleotides	Purines	Purine conversions	GMP synthase [glutamine-hydrolyzing] (EC 6.3.5.2)
Nucleosides and Nucleotides	Purines	Purine conversions	Inosine-uridine preferring nucleoside hydrolase (EC 3.2.2.1)
Phages, Prophages, Transposable elements, Plasmids	Phages, Prophages	Phage packaging machinery	Phage terminase, large subunit

Phages, Prophages, Transposable elements, Plasmids	Phages, Prophages	Phage packaging machinery	Phage terminase, small subunit
Phosphorus Metabolism	no subcategory	Phosphate metabolism	Alkaline phosphatase (EC 3.1.3.1)
Potassium metabolism	no subcategory	Potassium homeostasis	Possible potassium-efflux system protein
Potassium metabolism	no subcategory	Potassium homeostasis	Potassium voltage-gated channel subfamily KQT
Protein Metabolism	Protein biosynthesis	Programmed frameshift	programmed frameshift-containing
Protein Metabolism	Protein processing and modification	N-linked Glycosylation in Bacteria	4-amino-6-deoxy-N-Acetyl-D-hexosaminyl-(Lipid carrier) acetyltrasferase
Protein Metabolism	Protein processing and modification	N-linked Glycosylation in Bacteria	4-keto-6-deoxy-N-Acetyl-D-hexosaminyl-(Lipid carrier) aminotransferase
Regulation and Cell signaling	no subcategory	LysR-family proteins in Escherichia coli	Hydrogen peroxide-inducible genes activator
Regulons	Atomic Regulons	ar-431-EC Molybdopterin-guanine dinucleotide biosynthesis	Molybdopterin-guanine dinucleotide biosynthesis protein MobA
Respiration	Electron accepting reactions	Anaerobic respiratory reductases	Anaerobic dehydrogenases, typically selenocysteine-containing
Respiration	no subcategory	Biogenesis of cytochrome c oxidases	Cytochrome oxidase biogenesis protein Surf1, facilitates heme A insertion
Respiration	no subcategory	Formate hydrogenase	Formate dehydrogenase-O, major subunit (EC 1.2.1.2)
RNA Metabolism	RNA processing and modification	ATP-dependent RNA helicases, bacterial	ATP-dependent RNA helicase Atu1833
RNA Metabolism	RNA processing and modification	tRNA modification Bacteria	COG1720: Uncharacterized conserved protein
RNA Metabolism	Transcription	Transcription initiation, bacterial sigma factors	RNA polymerase sigma-70 factor
Stress Response	no subcategory	Bacterial hemoglobins	Putative bacterial haemoglobin
Stress Response	no subcategory	Universal stress protein family	Universal stress protein family 1
Stress Response	Osmotic stress	Choline and Betaine Uptake and Betaine Biosynthesis	Choline-sulfatase (EC 3.1.6.6)
Stress Response	Osmotic stress	Choline and Betaine Uptake and Betaine Biosynthesis	L-proline glycine betaine ABC transport system permease protein ProV (TC 3.A.1.12.1)
Stress Response	Osmotic stress	Osmoregulation	Aquaporin Z
Stress Response	Oxidative stress	Glutaredoxins	Uncharacterized monothiol glutaredoxin ycf64-like
Stress Response	Oxidative stress	Glutathione: Biosynthesis and gamma-glutamyl cycle	5-oxoprolinase (EC 3.5.2.9)
Stress Response	Oxidative stress	Oxidative stress	Paraquat-inducible protein A
Sulfur Metabolism	Inorganic sulfur assimilation	Inorganic Sulfur Assimilation	4Fe-4S ferredoxin, iron-sulfur binding
Sulfur Metabolism	Organic sulfur assimilation	Alkanesulfonate assimilation	ABC-type nitrate/sulfonate/bicarbonate transport system, ATPase component
Sulfur Metabolism	Organic sulfur assimilation	Alkanesulfonate assimilation	Alkanesulfonate monooxygenase (EC 1.14.14.5)
Virulence, Disease and Defense	Resistance to antibiotics and toxic compounds	Arsenic resistance	Anion permease ArsB/NhaD-like
Virulence, Disease and Defense	Resistance to antibiotics and toxic compounds	Arsenic resistance	Arsenical resistance operon repressor
Virulence, Disease and Defense	Resistance to antibiotics and toxic compounds	Beta-lactamase	Beta-lactamase
Virulence, Disease and Defense	Resistance to antibiotics and toxic compounds	Beta-lactamase	Beta-lactamase class D
Virulence, Disease and Defense	Resistance to antibiotics and toxic compounds	Multidrug Resistance Efflux Pumps	Multi antimicrobial extrusion protein (Na(+)/drug antiporter), MATE family of MDR efflux pumps
Virulence, Disease and Defense	Resistance to antibiotics and toxic compounds	Resistance to chromium compounds	Chromate transport protein ChrA

P. lamentivorans only

Amino Acids and Derivatives	Alanine, serine, and glycine	Alanine biosynthesis	Branched-chain amino acid aminotransferase (EC 2.6.1.42)
Amino Acids and Derivatives	Alanine, serine, and glycine	Glycine and Serine Utilization	L-serine dehydratase (EC 4.3.1.17)
Amino Acids and Derivatives	Alanine, serine, and glycine	Glycine and Serine Utilization	L-serine dehydratase, alpha subunit (EC 4.3.1.17)
Amino Acids and Derivatives	Alanine, serine, and glycine	Glycine and Serine Utilization	L-serine dehydratase, beta subunit (EC 4.3.1.17)
Amino Acids and Derivatives	Alanine, serine, and glycine	Glycine Biosynthesis	Low-specificity L-threonine aldolase (EC 4.1.2.5)
Amino Acids and Derivatives	Alanine, serine, and glycine	Glycine cleavage system	Glycine cleavage system transcriptional activator GcvA
Amino Acids and Derivatives	Arginine; urea cycle, polyamines	Arginine and Ornithine Degradation	N-carbamoylputrescine amidase (3.5.1.53)
Amino Acids and Derivatives	Arginine; urea cycle, polyamines	Arginine Deiminase Pathway	Carbamate kinase (EC 2.7.2.2)
Amino Acids and Derivatives	Arginine; urea cycle, polyamines	Cyanophycin Metabolism	Cyanophycin synthase (EC 6.3.2.29)(EC 6.3.2.30)
Amino Acids and Derivatives	Arginine; urea cycle, polyamines	Cyanophycin Metabolism	Cyanophycinase 2 (EC 3.4.15.6)
Amino Acids and Derivatives	Arginine; urea cycle, polyamines	Polyamine Metabolism	Agmatine/putrescine antiporter, associated with agmatine catabolism
Amino Acids and Derivatives	Arginine; urea cycle, polyamines	Polyamine Metabolism	Putrescine carbamoyltransferase (EC 2.1.3.6)
Amino Acids and Derivatives	Arginine; urea cycle, polyamines	Polyamine Metabolism	Putrescine transport ATP-binding protein PotG (TC 3.A.1.11.2)
Amino Acids and Derivatives	Arginine; urea cycle, polyamines	Polyamine Metabolism	S-adenosylmethionine decarboxylase proenzyme (EC 4.1.1.50), prokaryotic class 1B
Amino Acids and Derivatives	Arginine; urea cycle, polyamines	Polyamine Metabolism	Spermidine synthase (EC 2.5.1.16)
Amino Acids and Derivatives	Arginine; urea cycle, polyamines	Urea carboxylase and Allophanate hydrolase cluster	Allophanate hydrolase (EC 3.5.1.54)
Amino Acids and Derivatives	Arginine; urea cycle, polyamines	Urea carboxylase and Allophanate hydrolase cluster	Urea carboxylase (EC 6.3.4.6)
Amino Acids and Derivatives	Arginine; urea cycle, polyamines	Urea decomposition	Urea carboxylase-related ABC transporter, ATPase protein
Amino Acids and Derivatives	Arginine; urea cycle, polyamines	Urea decomposition	Urea carboxylase-related ABC transporter, periplasmic substrate-binding protein
Amino Acids and Derivatives	Arginine; urea cycle, polyamines	Urea decomposition	Urea carboxylase-related ABC transporter, permease protein
Amino Acids and Derivatives	Arginine; urea cycle, polyamines	Urea decomposition	Urea carboxylase-related aminomethyltransferase (EC 2.1.2.10)
Amino Acids and Derivatives	Aromatic amino acids and derivatives	Chorismate: Intermediate for synthesis of Tryptophan, PAPA antibiotics, PABA, 3-hydroxyanthranilate and more.	Isochorismatase (EC 3.3.2.1)
Amino Acids and Derivatives	Aromatic amino acids and derivatives	Phenylalanine and Tyrosine Branches from Chorismate	Cyclohexadienyl dehydratase (EC 4.2.1.51)(EC 4.2.1.91)
Amino Acids and Derivatives	Glutamine, glutamate, aspartate, asparagine; ammonia assimilation	Glutamine, Glutamate, Aspartate and Asparagine Biosynthesis	glutamine synthetase family protein
Amino Acids and Derivatives	Lysine, threonine, methionine, and cysteine	Cysteine Biosynthesis	Sulfate and thiosulfate binding protein CysP

Amino Acids and Derivatives	Lysine, threonine, methionine, and cysteine	Cysteine Biosynthesis	Sulfate and thiosulfate import ATP-binding protein CysA (EC 3.6.3.25)
Amino Acids and Derivatives	Lysine, threonine, methionine, and cysteine	Cysteine Biosynthesis	Sulfate transport system permease protein CysT
Amino Acids and Derivatives	Lysine, threonine, methionine, and cysteine	Cysteine Biosynthesis	Sulfate transport system permease protein CysW
Amino Acids and Derivatives	Lysine, threonine, methionine, and cysteine	Methionine Degradation	2-Oxobutyrate oxidase, putative
Amino Acids and Derivatives	Lysine, threonine, methionine, and cysteine	Methionine Degradation	Methionine gamma-lyase (EC 4.4.1.11)
Amino Acids and Derivatives	Lysine, threonine, methionine, and cysteine	Methionine Salvage	1,2-dihydroxy-3-keto-5-methylthiopentene dioxygenase (EC 1.13.11.54)
Amino Acids and Derivatives	Lysine, threonine, methionine, and cysteine	Methionine Salvage	2,3-diketo-5-methylthiopentyl-1-phosphate enolase-phosphatase (EC 3.1.3.77)
Amino Acids and Derivatives	Lysine, threonine, methionine, and cysteine	Methionine Salvage	5'-methylthioadenosine phosphorylase (EC 2.4.2.28)
Amino Acids and Derivatives	Lysine, threonine, methionine, and cysteine	Methionine Salvage	Aspartate/tyrosine/aromatic aminotransferase
Amino Acids and Derivatives	Lysine, threonine, methionine, and cysteine	Methionine Salvage	Glutamine-dependent 2-keto-4-methylthiobutyrate transaminase
Amino Acids and Derivatives	Lysine, threonine, methionine, and cysteine	Methionine Salvage	Methylthioribose-1-phosphate isomerase (EC 5.3.1.23)
Amino Acids and Derivatives	Lysine, threonine, methionine, and cysteine	Methionine Salvage	Methylthioribulose-1-phosphate dehydratase (EC 4.2.1.109)
Amino Acids and Derivatives	Lysine, threonine, methionine, and cysteine	Threonine degradation	Threonine dehydrogenase and related Zn-dependent dehydrogenases
Amino Acids and Derivatives	Proline and 4-hydroxyproline	A Hypothetical Protein Related to Proline Metabolism	Pyrroline-5-carboxylate reductase (EC 1.5.1.2)
Carbohydrates	Central carbohydrate metabolism	Dehydrogenase complexes	Transcriptional regulator BkdR of isoleucine and valine catabolism operon
Carbohydrates	Central carbohydrate metabolism	Ethylmalonyl-CoA pathway of C2 assimilation	Crotonyl-CoA carboxylase/reductase, ethylmalonyl-CoA producing
Carbohydrates	Central carbohydrate metabolism	Ethylmalonyl-CoA pathway of C2 assimilation	Ethylmalonyl-CoA mutase, methylsuccinyl-CoA-forming
Carbohydrates	Central carbohydrate metabolism	Ethylmalonyl-CoA pathway of C2 assimilation	Malate synthase (EC 2.3.3.9)
Carbohydrates	Central carbohydrate metabolism	Ethylmalonyl-CoA pathway of C2 assimilation, GJO	Ethylmalonyl-CoA epimerase
Carbohydrates	Central carbohydrate metabolism	Glycolysis and Gluconeogenesis	Fructose-1,6-bisphosphatase, type I (EC 3.1.3.11)
Carbohydrates	Central carbohydrate metabolism	Glycolysis and Gluconeogenesis	Fructose-bisphosphate aldolase class I (EC 4.1.2.13)
Carbohydrates	Central carbohydrate metabolism	Glycolysis and Gluconeogenesis	Glucokinase (EC 2.7.1.2)
Carbohydrates	Central carbohydrate metabolism	Pyruvate Alanine Serine Interconversions	Alanine dehydrogenase (EC 1.4.1.1)
Carbohydrates	Central carbohydrate metabolism	Pyruvate metabolism I: anaplerotic reactions, PEP	D-malic enzyme (EC 1.1.1.83)
Carbohydrates	Central carbohydrate metabolism	TCA Cycle	Citrate lyase beta chain (EC 4.1.3.6)
Carbohydrates	Di- and oligosaccharides	Lactose and Galactose Uptake and Utilization	Beta-galactosidase (EC 3.2.1.23), LacA family
Carbohydrates	Di- and oligosaccharides	Lactose and Galactose Uptake and Utilization	Galactokinase (EC 2.7.1.6)
Carbohydrates	Di- and oligosaccharides	Lactose and Galactose Uptake and Utilization	Galactose-1-phosphate uridylyltransferase (EC 2.7.7.10)
Carbohydrates	Di- and oligosaccharides	Maltose and Maltodextrin Utilization	Maltodextrin glucosidase (EC 3.2.1.20)
Carbohydrates	Di- and oligosaccharides	Trehalose Biosynthesis	Malto-oligosyltrehalose trehalohydrolase (EC 3.2.1.141)
Carbohydrates	Di- and oligosaccharides	Trehalose Biosynthesis	Trehalose synthase (EC 5.4.99.16)
Carbohydrates	Fermentation	Acetyl-CoA fermentation to Butyrate	3-hydroxybutyryl-CoA dehydratase (EC 4.2.1.55)
Carbohydrates	Fermentation	Acetyl-CoA fermentation to Butyrate	Acetoacetyl-CoA reductase (EC 1.1.1.36)
Carbohydrates	Fermentation	Fermentations: Lactate	Acetate kinase (EC 2.7.2.1)
Carbohydrates	Fermentation	Fermentations: Lactate	Fructose-6-phosphate phosphoketolase (EC 4.1.2.22)
Carbohydrates	Fermentation	Fermentations: Lactate	Phosphate acetyltransferase (EC 2.3.1.8)
Carbohydrates	Fermentation	Fermentations: Lactate	Xylulose-5-phosphate phosphoketolase (EC 4.1.2.9)
Carbohydrates	Monosaccharides	D-gluconate and ketogluconates metabolism	5-keto-D-gluconate 5-reductase (EC 1.1.1.69)
Carbohydrates	Monosaccharides	D-ribose utilization	Ribokinase (EC 2.7.1.15)
Carbohydrates	Organic acids	Lactate utilization	L-lactate dehydrogenase (EC 1.1.2.3)
Carbohydrates	Polysaccharides	Glycogen metabolism	1,4-alpha-glucan (glycogen) branching enzyme, GH-13-type (EC 2.4.1.18)
Carbohydrates	Polysaccharides	Glycogen metabolism	4-alpha-glucanotransferase (amylomaltase) (EC 2.4.1.25)
Carbohydrates	Polysaccharides	Glycogen metabolism	Glycogen debranching enzyme (EC 3.2.1.-)
Carbohydrates	Polysaccharides	Glycogen metabolism	Malto-oligosyltrehalose synthase (EC 5.4.99.15)
Cell Wall and Capsule	Capsular and extracellular polysacchrides	dTDP-rhamnose synthesis	dTDP-Rha:A-D-GlcNAc-diphosphoryl polyprenol, A-3-L-rhamnosyl transferase WbbL
Cell Wall and Capsule	Capsular and extracellular polysacchrides	Rhamnose containing glycans	Glucose-1-phosphate thymidylyltransferase (EC 2.7.7.24)
Cell Wall and Capsule	Gram-Negative cell wall components	KDO2-Lipid A biosynthesis	Lipid A export ATP-binding/permease protein MsbA (EC 3.6.3.25)
Cell Wall and Capsule	Gram-Negative cell wall components	KDO2-Lipid A biosynthesis	UDP-3-O-[3-hydroxymyristoyl] glucosamine N-acyltransferase (EC 2.3.1.-)
Cell Wall and Capsule	Gram-Negative cell wall components	Lipoprotein sorting system	Lipoprotein releasing system transmembrane protein LolE
Cell Wall and Capsule	Gram-Negative cell wall components	LOS core oligosaccharide biosynthesis	Beta-1,3-glucosyltransferase
Clustering-based subsystems	Cell Division	Cell Division Subsystem including YidCD	Protein YidD
Clustering-based subsystems	DNA metabolism	CBSS-269801.1.peg.2186	Exodeoxyribonuclease V alpha chain (EC 3.1.11.5)
Clustering-based subsystems	DNA metabolism	CBSS-272943.3.peg.263	Phosphotransferase involved in threonylcarbamoyladenosine t(6)A37 formation in tRNA
Clustering-based subsystems	DNA metabolism	Rad50-Mre11 DNA repair cluster	DNA double-strand break repair Rad50 ATPase
Clustering-based subsystems	DNA metabolism	Rad50-Mre11 DNA repair cluster	DNA double-strand break repair protein Mre11
Clustering-based subsystems	DNA metabolism	Rad50-Mre11 DNA repair cluster	FIG036446: hypothetical protein
Clustering-based subsystems	Isoprenoid/cell wall biosynthesis: PREDICTED UNDECAPRENYL DIPHOSPHATE PHOSPHATASE	CBSS-83331.1.peg.3039	Intramembrane protease RasP/YluC, implicated in cell division based on FtsL cleavage
Clustering-based subsystems	Lysine Biosynthesis	A Glutathione-dependent Thiol Reductase Associated with a Step in Lysine Biosynthesis	FIG138056: a glutathione-dependent thiol reductase
Clustering-based subsystems	May be related to ADP-phosphoribose and NAD-dependent acetylation	CBSS-216591.1.peg.168	Alpha-ribazole-5'-phosphate phosphatase (EC 3.1.3.73)
Clustering-based subsystems	no subcategory	A Heme Biosynthetic Cluster	FIG000868: Homolog of E. coli HemY protein
Clustering-based subsystems	no subcategory	A Heme Biosynthetic Cluster	FIG006581: hypothetical protein
Clustering-based subsystems	no subcategory	A hypothetical related to DNA replication and repair	DNA polymerase IV-like protein ImuB

Clustering-based subsystems	no subcategory	A hypothetical related to DNA replication and repair	hypothetical protein related to DNA replication/repair
Clustering-based subsystems	no subcategory	Biotin carboxylase and ubiquinone oxidoreductase subunit cluster	FIG167255: hypothetical protein
Clustering-based subsystems	no subcategory	CBSS-160492.1.peg.550	21 kDa hemolysin precursor
Clustering-based subsystems	no subcategory	CBSS-160492.1.peg.550	Predicted endonuclease distantly related to archaeal Holliday junction resolvase
Clustering-based subsystems	no subcategory	CBSS-224911.1.peg.6954	FIG002283: Isochorismatase family protein
Clustering-based subsystems	no subcategory	CBSS-224911.1.peg.6954	FIG022068: Hypothetical protein
Clustering-based subsystems	no subcategory	CBSS-224911.1.peg.6954	FIG022886: Transcriptional regulator, LysR family
Clustering-based subsystems	no subcategory	CBSS-227377.1.peg.2035	FIG050068: DNA-binding protein
Clustering-based subsystems	no subcategory	CBSS-227377.1.peg.2035	FIG071147: hypothetical protein
Clustering-based subsystems	no subcategory	CBSS-291331.3.peg.3674	FIG001571: Hypothetical protein
Clustering-based subsystems	no subcategory	CBSS-291331.3.peg.3674	S-adenosyl-L-methionine dependent methyltransferase, similar to cyclopropane-fatty-acyl-phospholipid synthase
Clustering-based subsystems	no subcategory	CBSS-314269.3.peg.1840	Carbon monoxide dehydrogenase large chain (EC 1.2.99.2)
Clustering-based subsystems	no subcategory	CBSS-314269.3.peg.1840	Carbon monoxide dehydrogenase medium chain (EC 1.2.99.2)
Clustering-based subsystems	no subcategory	CBSS-314269.3.peg.1840	Carbon monoxide dehydrogenase small chain (EC 1.2.99.2)
Clustering-based subsystems	no subcategory	CBSS-314269.3.peg.1840	Carbon monoxide oxidation accessory protein CoxD
Clustering-based subsystems	no subcategory	CBSS-314269.3.peg.1840	Carbon monoxide oxidation accessory protein CoxE
Clustering-based subsystems	no subcategory	CBSS-314269.3.peg.1840	Carbon-monoxide dehydrogenase form II, large subunit (EC 1.2.99.2)
Clustering-based subsystems	no subcategory	CBSS-316056.14.peg.4707	NADPH-dependent glyceraldehyde-3-phosphate dehydrogenase (EC 1.2.1.13)
Clustering-based subsystems	no subcategory	CBSS-316273.3.peg.2709	FIG034376: Hypothetical protein
Clustering-based subsystems	no subcategory	CBSS-316273.3.peg.2709	FIG034458: Plasmid related protein
Clustering-based subsystems	no subcategory	CBSS-316273.3.peg.2709	FIG036757: Plasmid-related protein
Clustering-based subsystems	no subcategory	CBSS-316273.3.peg.2709	FIG049434: Periplasmic protein TonB, links inner and outer membranes
Clustering-based subsystems	no subcategory	CBSS-316273.3.peg.2709	FIG068798: Hypothetical protein
Clustering-based subsystems	no subcategory	CBSS-316273.3.peg.2709	FIG076210: Hypothetical protein
Clustering-based subsystems	no subcategory	CBSS-316273.3.peg.2709	FIG076676: Hypothetical protein
Clustering-based subsystems	no subcategory	CBSS-316273.3.peg.2709	Superfamily II DNA/RNA helicases, SNF2 family
Clustering-based subsystems	no subcategory	CBSS-783.1.peg.807	FIG020313: hypothetical protein
Clustering-based subsystems	no subcategory	Cell division-ribosomal stress proteins cluster	Hypoxanthine-guanine phosphoribosyltransferase (EC 2.4.2.8)
Clustering-based subsystems	no subcategory	Cell division-ribosomal stress proteins cluster	Peptidyl-tRNA hydrolase (EC 3.1.1.29)
Clustering-based subsystems	no subcategory	Choline uptake and conversion to betaine clusters	FIG023769: Choline transport related protein
Clustering-based subsystems	no subcategory	Choline uptake and conversion to betaine clusters	High-affinity choline uptake protein BetT
Clustering-based subsystems	no subcategory	Llipid A biosynthesis cluster	Outer membrane protein H precursor
Clustering-based subsystems	no subcategory	PFGI-1-like cluster 1	Chromosome partitioning ATPase in PFGI-1-like cluster, ParA-like
Clustering-based subsystems	no subcategory	PFGI-1-like cluster 1	FIG004780: hypothetical protein in PFGI-1-like cluster
Clustering-based subsystems	no subcategory	PFGI-1-like cluster 1	FIG034647: hypothetical protein in PFGI-1-like cluster
Clustering-based subsystems	no subcategory	PFGI-1-like cluster 1	FIG041388: hypothetical protein in PFGI-1-like cluster
Clustering-based subsystems	no subcategory	PFGI-1-like cluster 1	FIG141694: hypothetical protein in PFGI-1-like cluster
Clustering-based subsystems	no subcategory	PFGI-1-like cluster 1	FIG141751: hypothetical protein in PFGI-1-like cluster
Clustering-based subsystems	no subcategory	PFGI-1-like cluster 1	Integrase regulator R
Clustering-based subsystems	no subcategory	PFGI-1-like cluster 1	Protein with ParB-like nuclease domain in PFGI-1-like cluster
Clustering-based subsystems	no subcategory	PFGI-1-like cluster 1	Single-stranded DNA-binding protein in PFGI-1-like cluster
Clustering-based subsystems	no subcategory	PFGI-1-like cluster 1	Transcriptional regulator in PFGI-1-like cluster
Clustering-based subsystems	no subcategory	Stationary phase repair cluster	Lipoprotein NlpD
Clustering-based subsystems	Probably organic hydroperoxide resistance related hypothetical protein	CBSS-269482.1.peg.1294	Organic hydroperoxide resistance protein
Clustering-based subsystems	Probably organic hydroperoxide resistance related hypothetical protein	CBSS-269482.1.peg.1294	Organic hydroperoxide resistance transcriptional regulator
Clustering-based subsystems	Putative asociate of RNA polymerase sigma-54 factor rpoN	CBSS-316057.3.peg.1308	RNA polymerase sigma-70 factor, ECF family
Clustering-based subsystems	Three hypotheticals linked to lipoprotein biosynthesis	CBSS-188.1.peg.9880	COG1496: Uncharacterized conserved protein
Clustering-based subsystems	Three hypotheticals linked to lipoprotein biosynthesis	CBSS-188.1.peg.9880	COG1565: Uncharacterized conserved protein
Clustering-based subsystems	Three hypotheticals linked to lipoprotein biosynthesis	CBSS-188.1.peg.9880	FIG018229: hypothetical protein
Clustering-based subsystems	Three hypotheticals linked to lipoprotein biosynthesis	CBSS-188.1.peg.9880	Prolipoprotein diacylglyceryl transferase (EC 2.4.99.-)
Cofactors, Vitamins, Prosthetic Groups, Pigments	Biotin	Biotin biosynthesis	Biotin synthesis protein BioH
Cofactors, Vitamins, Prosthetic Groups, Pigments	Biotin	Biotin biosynthesis Experimental	tRNA (cytidine(34)-2'-O)-methyltransferase (EC 2.1.1.207)
Cofactors, Vitamins, Prosthetic Groups, Pigments	Coenzyme A	Coenzyme A Biosynthesis	Pantothenate kinase (EC 2.7.1.33)
Cofactors, Vitamins, Prosthetic Groups, Pigments	Coenzyme A	Coenzyme A Biosynthesis	Phosphopantothenoylcysteine decarboxylase (EC 4.1.1.36)
Cofactors, Vitamins, Prosthetic Groups, Pigments	Coenzyme A	Coenzyme A Biosynthesis	Phosphopantothenoylcysteine synthetase (EC 6.3.2.5)
Cofactors, Vitamins, Prosthetic Groups, Pigments	Folate and pterines	5-FCL-like protein	Phosphomethylpyrimidine kinase (EC 2.7.4.7)
Cofactors, Vitamins, Prosthetic Groups, Pigments	Folate and pterines	Molybdenum cofactor biosynthesis	Molybdenum cofactor biosynthesis protein MoaD
Cofactors, Vitamins, Prosthetic Groups, Pigments	Folate and pterines	Molybdenum cofactor biosynthesis	Molybdenum cofactor biosynthesis protein MoaE
Cofactors, Vitamins, Prosthetic Groups, Pigments	Folate and pterines	Molybdenum cofactor biosynthesis	Molybdenum transport ATP-binding protein ModC (TC 3.A.1.8.1)
Cofactors, Vitamins, Prosthetic Groups, Pigments	Folate and pterines	Molybdenum cofactor biosynthesis	Sulfur carrier protein adenylyltransferase ThiF

Cofactors, Vitamins, Prosthetic Groups, Pigments	no subcategory	Thiamin biosynthesis	Glycine oxidase ThiO (EC 1.4.3.19)
Cofactors, Vitamins, Prosthetic Groups, Pigments	Pyridoxine	Pyridoxin (Vitamin B6) Biosynthesis	Predicted transcriptional regulator of pyridoxine metabolism
Cofactors, Vitamins, Prosthetic Groups, Pigments	Quinone cofactors	Ubiquinone Biosynthesis - gjo	ABC1 family protein
Cofactors, Vitamins, Prosthetic Groups, Pigments	Riboflavin, FMN, FAD	Flavodoxin	Flavodoxin 2
Cofactors, Vitamins, Prosthetic Groups, Pigments	Riboflavin, FMN, FAD	Flavodoxin	NAD(P)H oxidoreductase YRKL (EC 1.6.99.-)
Cofactors, Vitamins, Prosthetic Groups, Pigments	Riboflavin, FMN, FAD	Riboflavin, FMN and FAD metabolism in plants	tRNA (5-methylaminomethyl-2-thiouridylate)-methyltransferase (EC 2.1.1.61)
DNA Metabolism	CRISPs	CRISPRs	CRISPR-associated protein Cas1
DNA Metabolism	CRISPs	CRISPRs	CRISPR-associated protein, Csn1 family
DNA Metabolism	DNA repair	2-phosphoglycolate salvage	Putative phosphatase YieH
DNA Metabolism	DNA repair	DNA Repair Base Excision	Exodeoxyribonuclease I (EC 3.1.11.1)
DNA Metabolism	DNA repair	DNA repair, bacterial	DNA-damage-inducible protein J
DNA Metabolism	DNA repair	DNA repair, bacterial	G:T/U mismatch-specific uracil/thymine DNA-glycosylase
DNA Metabolism	DNA repair	DNA repair, UvrABC system	Excinuclease ABC subunit B
DNA Metabolism	DNA repair	DNA repair, UvrABC system	Excinuclease ABC subunit C
DNA Metabolism	DNA replication	DNA replication strays	Error-prone repair homolog of DNA polymerase III alpha subunit (EC 2.7.7.7)
DNA Metabolism	DNA replication	DNA topoisomerases, Type I, ATP-independent	DNA topoisomerase III (EC 5.99.1.2) in PFGI-1-like cluster
DNA Metabolism	DNA uptake, competence	DNA processing cluster	Protein involved in catabolism of external DNA
DNA Metabolism	no subcategory	Restriction-Modification System	Putative DNA-binding protein in cluster with Type I restriction-modification system
Dormancy and Sporulation	no subcategory	Persister Cells	HipA protein
Fatty Acids, Lipids, and Isoprenoids	Fatty acids	Phospholipid and Fatty acid biosynthesis related cluster	(3R)-hydroxymyristoyl-[ACP] dehydratase (EC 4.2.1.-)
Fatty Acids, Lipids, and Isoprenoids	Fatty acids	Phospholipid and Fatty acid biosynthesis related cluster	3-hydroxydecanoyl-[ACP] dehydratase (EC 4.2.1.60)
Fatty Acids, Lipids, and Isoprenoids	Fatty acids	Phospholipid and Fatty acid biosynthesis related cluster	3-oxoacyl-[ACP] reductase (EC 1.1.1.100)
Fatty Acids, Lipids, and Isoprenoids	Fatty acids	Phospholipid and Fatty acid biosynthesis related cluster	3-oxoacyl-[ACP] synthase
Fatty Acids, Lipids, and Isoprenoids	Fatty acids	Phospholipid and Fatty acid biosynthesis related cluster	3-oxoacyl-[ACP] synthase (EC 2.3.1.41) FabV like
Fatty Acids, Lipids, and Isoprenoids	Fatty acids	Phospholipid and Fatty acid biosynthesis related cluster	FIG002571: 4-hydroxybenzoyl-CoA thioesterase domain protein
Fatty Acids, Lipids, and Isoprenoids	Fatty acids	Phospholipid and Fatty acid biosynthesis related cluster	FIG017861: hypothetical protein
Fatty Acids, Lipids, and Isoprenoids	Fatty acids	Phospholipid and Fatty acid biosynthesis related cluster	FIG018329: 1-acyl-sn-glycerol-3-phosphate acyltransferase
Fatty Acids, Lipids, and Isoprenoids	Fatty acids	Phospholipid and Fatty acid biosynthesis related cluster	FIG021862: membrane protein, exporter
Fatty Acids, Lipids, and Isoprenoids	Fatty acids	Phospholipid and Fatty acid biosynthesis related cluster	FIG022199: FAD-binding protein
Fatty Acids, Lipids, and Isoprenoids	Fatty acids	Phospholipid and Fatty acid biosynthesis related cluster	FIG027190: Putative transmembrane protein
Fatty Acids, Lipids, and Isoprenoids	Fatty acids	Phospholipid and Fatty acid biosynthesis related cluster	FIG035331: hypothetical protein
Fatty Acids, Lipids, and Isoprenoids	Fatty acids	Phospholipid and Fatty acid biosynthesis related cluster	FIG138576: 3-oxoacyl-[ACP] synthase (EC 2.3.1.41)
Fatty Acids, Lipids, and Isoprenoids	Fatty acids	Phospholipid and Fatty acid biosynthesis related cluster	FIG143263: Glycosyl transferase
Fatty Acids, Lipids, and Isoprenoids	Fatty acids	Phospholipid and Fatty acid biosynthesis related cluster	FIGfam138462: Acyl-CoA synthetase, AMP-(fatty) acid ligase
Fatty Acids, Lipids, and Isoprenoids	Fatty acids	Phospholipid and Fatty acid biosynthesis related cluster	Lysophospholipid acyltransferase
Fatty Acids, Lipids, and Isoprenoids	Fatty acids	Phospholipid and Fatty acid biosynthesis related cluster	Putative histidine ammonia-lyase protein
Fatty Acids, Lipids, and Isoprenoids	Isoprenoids	Isoprenoinds for Quinones	Geranylgeranyl diphosphate synthase (EC 2.5.1.29)
Fatty Acids, Lipids, and Isoprenoids	no subcategory	Polyhydroxybutyrate metabolism	D-beta-hydroxybutyrate dehydrogenase (EC 1.1.1.30)
Fatty Acids, Lipids, and Isoprenoids	Phospholipids	Glycerolipid and Glycerophospholipid Metabolism in Bacteria	Acyl carrier protein (ACP1)
Fatty Acids, Lipids, and Isoprenoids	Phospholipids	Glycerolipid and Glycerophospholipid Metabolism in Bacteria	Acyl carrier protein (ACP2)
Fatty Acids, Lipids, and Isoprenoids	Phospholipids	Glycerolipid and Glycerophospholipid Metabolism in Bacteria	Anaerobic glycerol-3-phosphate dehydrogenase subunit C (EC 1.1.5.3)
Iron acquisition and metabolism	no subcategory	Heme, hemin uptake and utilization systems in GramNegatives	ABC-type hemin transport system, ATPase component
Iron acquisition and metabolism	no subcategory	Heme, hemin uptake and utilization systems in GramNegatives	Ferric siderophore transport system, biopolymer transport protein ExbB
Iron acquisition and metabolism	no subcategory	Heme, hemin uptake and utilization systems in GramNegatives	Hemin ABC transporter, permease protein
Iron acquisition and metabolism	no subcategory	Heme, hemin uptake and utilization systems in GramNegatives	Hemin transport protein HmuS
Iron acquisition and metabolism	no subcategory	Heme, hemin uptake and utilization systems in GramNegatives	Outer membrane receptor proteins, mostly Fe transport
Iron acquisition and metabolism	no subcategory	Heme, hemin uptake and utilization systems in GramNegatives	Periplasmic hemin-binding protein
Iron acquisition and metabolism	no subcategory	Heme, hemin uptake and utilization systems in GramNegatives	Putative heme iron utilization protein
Iron acquisition and metabolism	no subcategory	Heme, hemin uptake and utilization systems in GramNegatives	TonB-dependent hemin , ferrichrome receptor
Membrane Transport	Cation transporters	Copper transport and blue copper proteins	Copper-containing nitrite reductase (EC 1.7.2.1)
Membrane Transport	Cation transporters	Copper transport and blue copper proteins	Nitric oxide -responding transcriptional regulator NnrR (Crp/Fnr family)
Membrane Transport	Cation transporters	Copper transport and blue copper proteins	NnrS protein involved in response to NO
Membrane Transport	Cation transporters	Copper transport and blue copper proteins	Pseudoazurin

Membrane Transport	Cation transporters	Copper Transport System	Conserved membrane protein in copper uptake, YcnI
Membrane Transport	Cation transporters	Magnesium transport	Cation-transporting ATPase, E1-E2 family
Membrane Transport	Protein secretion system, Type II	Widespread colonization island	ATP-dependent helicase, DEAD/DEAH box family, associated with Flp pilus assembly
Membrane Transport	TRAP transporters	TRAP Transporter collection	TRAP-type C4-dicarboxylate transport system, large permease component
Membrane Transport	TRAP transporters	TRAP Transporter collection	TRAP-type C4-dicarboxylate transport system, periplasmic component
Membrane Transport	TRAP transporters	TRAP Transporter collection	TRAP-type C4-dicarboxylate transport system, small permease component
Membrane Transport	Uni- Sym- and Antiporters	NhaA, NhaD and Sodium-dependent phosphate transporters	Sodium-dependent phosphate transporter
Metabolism of Aromatic Compounds	Anaerobic degradation of aromatic compounds	Acetophenone carboxylase 1	Acetophenone carboxylase subunit Apc1
Metabolism of Aromatic Compounds	Anaerobic degradation of aromatic compounds	Acetophenone carboxylase 1	Acetophenone carboxylase subunit Apc2
Metabolism of Aromatic Compounds	Anaerobic degradation of aromatic compounds	Acetophenone carboxylase 1	Acetophenone carboxylase subunit Apc3
Metabolism of Aromatic Compounds	Anaerobic degradation of aromatic compounds	Acetophenone carboxylase 1	Acetophenone carboxylase subunit Apc4
Metabolism of Aromatic Compounds	no subcategory	Aromatic Amin Catabolism	4-hydroxyphenylacetate 3-monooxygenase (EC 1.14.13.3)
Metabolism of Aromatic Compounds	no subcategory	Gentisare degradation	2-polyprenyl-6-methoxyphenol hydroxylase and related FAD-dependent oxidoreductases
Metabolism of Aromatic Compounds	Peripheral pathways for catabolism of aromatic compounds	Biphenyl Degradation	2-keto-4-pentenoate hydratase (EC 4.2.1.80)
Miscellaneous	no subcategory	Aromatic dioxygenase mess	2,3-dihydroxybiphenyl 1,2-dioxygenase (EC 1.13.11.39)
Miscellaneous	no subcategory	Aromatic dioxygenase mess	Large subunit toluate/benzoate dioxygenase
Miscellaneous	no subcategory	Aromatic dioxygenase mess	Small subunit toluate/benzoate dioxygenase
Miscellaneous	no subcategory	Bacillus subtilis scratch - gjo	ATP-dependent DNA ligase (EC 6.5.1.1) clustered with Ku protein, LigD
Miscellaneous	no subcategory	Bacillus subtilis scratch - gjo	Ku domain protein
Miscellaneous	no subcategory	Broadly distributed proteins not in subsystems	FIG001943: hypothetical protein YajQ
Miscellaneous	no subcategory	Broadly distributed proteins not in subsystems	UPF0225 protein YchJ
Miscellaneous	no subcategory	Muconate lactonizing enzyme family	L-alanine-DL-glutamate epimerase
Motility and Chemotaxis	Flagellar motility in Prokaryota	Flagellar motility	Rrf2 family transcriptional regulator
Motility and Chemotaxis	Flagellar motility in Prokaryota	Flagellum	Flagellar protein FlbB
Motility and Chemotaxis	Flagellar motility in Prokaryota	Flagellum	Flagellar regulatory protein FleQ
Nucleosides and Nucleotides	Detoxification	Nudix proteins (nucleoside triphosphate hydrolases)	Thiamin-phosphate pyrophosphorylase-like protein
Nucleosides and Nucleotides	no subcategory	dNTP triphosphohydrolase protein family	dNTP triphosphohydrolase, broad substrate specificity, subgroup 2
Nucleosides and Nucleotides	Purines	De Novo Purine Biosynthesis	Ribose-phosphate pyrophosphokinase (EC 2.7.6.1)
Nucleosides and Nucleotides	Purines	GMP synthase	GMP synthase [glutamine-hydrolyzing], ATP pyrophosphatase subunit (EC 6.3.5.2)
Nucleosides and Nucleotides	Purines	GMP synthase	GMP synthase [glutamine-hydrolyzing], amidotransferase subunit (EC 6.3.5.2)
Nucleosides and Nucleotides	Purines	Purine Utilization	Xanthine/uracil/thiamine/ascorbate permease family protein
Nucleosides and Nucleotides	Pyrimidines	pyrimidine conversions	Thymidine phosphorylase (EC 2.4.2.4)
Phages, Prophages, Transposable elements, Plasmids	Gene Transfer Agent (GTA)	Gene Transfer Agent	Gene Transfer Agent (GTA) ORFG06
Phages, Prophages, Transposable elements, Plasmids	Gene Transfer Agent (GTA)	Gene Transfer Agent	Gene Transfer Agent (GTA) ORFG07
Phages, Prophages, Transposable elements, Plasmids	Gene Transfer Agent (GTA)	Gene Transfer Agent	Gene Transfer Agent (GTA) ORFG08
Phages, Prophages, Transposable elements, Plasmids	Gene Transfer Agent (GTA)	Gene Transfer Agent	Gene Transfer Agent (GTA) ORFG10
Phages, Prophages, Transposable elements, Plasmids	Gene Transfer Agent (GTA)	Gene Transfer Agent	Gene Transfer Agent (GTA) ORFG10b
Phages, Prophages, Transposable elements, Plasmids	Gene Transfer Agent (GTA)	Gene Transfer Agent	Gene Transfer Agent (GTA) ORFG12
Phages, Prophages, Transposable elements, Plasmids	Gene Transfer Agent (GTA)	Gene Transfer Agent	Gene Transfer Agent FAD/FMN-containing dehydrogenase
Phages, Prophages, Transposable elements, Plasmids	Gene Transfer Agent (GTA)	Gene Transfer Agent	Gene Transfer Agent NlpC/P60 family peptidase
Phages, Prophages, Transposable elements, Plasmids	Gene Transfer Agent (GTA)	Gene Transfer Agent	Gene Transfer Agent capsid protein
Phages, Prophages, Transposable elements, Plasmids	Gene Transfer Agent (GTA)	Gene Transfer Agent	Gene Transfer Agent host specificity protein
Phages, Prophages, Transposable elements, Plasmids	Gene Transfer Agent (GTA)	Gene Transfer Agent	Gene Transfer Agent portal protein
Phages, Prophages, Transposable elements, Plasmids	Gene Transfer Agent (GTA)	Gene Transfer Agent	Gene Transfer Agent prohead protease
Phages, Prophages, Transposable elements, Plasmids	Gene Transfer Agent (GTA)	Gene Transfer Agent	Gene Transfer Agent tail protein
Phages, Prophages, Transposable elements, Plasmids	Gene Transfer Agent (GTA)	Gene Transfer Agent	Gene Transfer Agent tail tape measure

Phages, Prophages, Transposable elements, Plasmids	Gene Transfer Agent (GTA)	Gene Transfer Agent	Gene Transfer Agent terminase protein
Phages, Prophages, Transposable elements, Plasmids	Phages, Prophages	Phage capsid proteins	Phage major capsid protein
Phosphorus Metabolism	no subcategory	Alkylphosphonate utilization	Alkylphosphonate utilization operon protein PhnA
Phosphorus Metabolism	no subcategory	Alkylphosphonate utilization	PhnB protein
Phosphorus Metabolism	no subcategory	High affinity phosphate transporter and control of PHO regulon	Phosphate-specific outer membrane porin OprP
Phosphorus Metabolism	no subcategory	High affinity phosphate transporter and control of PHO regulon	Pyrophosphate-specific outer membrane porin OprO
Phosphorus Metabolism	no subcategory	Phosphate metabolism	Probable low-affinity inorganic phosphate transporter
Protein Metabolism	Protein biosynthesis	Ribosome LSU bacterial	LSU ribosomal protein L25p
Protein Metabolism	Protein biosynthesis	Ribosome SSU bacterial	SSU ribosomal protein S13p (S18e)
Protein Metabolism	Protein biosynthesis	Translation termination factors bacterial	Hypothetical protein YaeJ with similarity to translation release factor
Protein Metabolism	Protein biosynthesis	Translation termination factors bacterial	Peptide chain release factor 3
Protein Metabolism	Protein biosynthesis	Universal GTPases	GTP-binding and nucleic acid-binding protein YchF
Protein Metabolism	Protein degradation	Metallocarboxypeptidases (EC 3.4.17.-)	Thermostable carboxypeptidase 1 (EC 3.4.17.19)
Protein Metabolism	Protein degradation	Protein degradation	Deblocking aminopeptidase (EC 3.4.11.-)
Protein Metabolism	Protein degradation	Proteolysis in bacteria, ATP-dependent	ATP-dependent protease La (EC 3.4.21.53) Type II
Protein Metabolism	Protein folding	Protein chaperones	DnaJ-class molecular chaperone CbpA
Protein Metabolism	Protein processing and modification	G3E family of P-loop GTPases (metallocenter biosynthesis)	FKBP-type peptidyl-prolyl cis-trans isomerase SlyD (EC 5.2.1.8)
Protein Metabolism	Protein processing and modification	G3E family of P-loop GTPases (metallocenter biosynthesis)	Methylmalonyl-CoA mutase (EC 5.4.99.2)
Protein Metabolism	Protein processing and modification	G3E family of P-loop GTPases (metallocenter biosynthesis)	putative periplasmic protein kinase ArgK and related GTPases of G3E family
Protein Metabolism	Protein processing and modification	Ribosomal protein S5p acylation	Ribosomal-protein-L7p-serine acetyltransferase
Regulation and Cell signaling	no subcategory	HPr catabolite repression system	Phosphotransferase system, phosphocarrier protein HPr
Regulation and Cell signaling	no subcategory	Orphan regulatory proteins	Two-component nitrogen fixation transcriptional regulator FixJ
Regulation and Cell signaling	no subcategory	Orphan regulatory proteins	Two-component oxygen-sensor histidine kinase FixL
Regulation and Cell signaling	no subcategory	Stringent Response, (p)ppGpp metabolism	Adenine phosphoribosyltransferase (EC 2.4.2.7)
Regulation and Cell signaling	Programmed Cell Death and Toxin-antitoxin Systems	Murein hydrolase regulation and cell death	LysR family regulatory protein CidR
Regulation and Cell signaling	Programmed Cell Death and Toxin-antitoxin Systems	Toxin-antitoxin replicon stabilization systems	HigA protein (antitoxin to HigB)
Regulation and Cell signaling	Programmed Cell Death and Toxin-antitoxin Systems	Toxin-antitoxin replicon stabilization systems	HigB toxin protein
Regulation and Cell signaling	Programmed Cell Death and Toxin-antitoxin Systems	Toxin-antitoxin replicon stabilization systems	ParD protein (antitoxin to ParE)
Regulation and Cell signaling	Programmed Cell Death and Toxin-antitoxin Systems	Toxin-antitoxin replicon stabilization systems	ParE toxin protein
Regulation and Cell signaling	Programmed Cell Death and Toxin-antitoxin Systems	Toxin-antitoxin replicon stabilization systems	RelB/StbD replicon stabilization protein (antitoxin to RelE/StbE)
Regulation and Cell signaling	Programmed Cell Death and Toxin-antitoxin Systems	Toxin-antitoxin replicon stabilization systems	RelE/StbE replicon stabilization toxin
Regulation and Cell signaling	Programmed Cell Death and Toxin-antitoxin Systems	Toxin-antitoxin replicon stabilization systems	VapB protein (antitoxin to VapC)
Regulation and Cell signaling	Programmed Cell Death and Toxin-antitoxin Systems	Toxin-antitoxin replicon stabilization systems	VapC toxin protein
Regulation and Cell signaling	Programmed Cell Death and Toxin-antitoxin Systems	Toxin-antitoxin replicon stabilization systems	YafQ toxin protein
Respiration	ATP synthases	F0F1-type ATP synthase	ATP synthase F0 sector subunit a (EC 3.6.3.14)
Respiration	ATP synthases	F0F1-type ATP synthase	ATP synthase F0 sector subunit b (EC 3.6.3.14)
Respiration	ATP synthases	F0F1-type ATP synthase	ATP synthase F0 sector subunit b' (EC 3.6.3.14)
Respiration	ATP synthases	F0F1-type ATP synthase	ATP synthase F0 sector subunit c (EC 3.6.3.14)
Respiration	Electron donating reactions	CO Dehydrogenase	Aerobic carbon monoxide dehydrogenase molybdenum cofactor insertion protein CoxF
Respiration	Electron donating reactions	CO Dehydrogenase	Carbon monoxide oxidation accessory protein CoxG
Respiration	no subcategory	Biogenesis of c-type cytochromes	Predicted dye-decolorizing peroxidase (DyP), encapsulated subgroup
Respiration	no subcategory	Quinone oxidoreductase family	Putative NADP-dependent oxidoreductase PA1648
Respiration	no subcategory	Quinone oxidoreductase family	YhdH, a putative quinone oxidoreductase
RNA Metabolism	RNA processing and modification	Queuosine-Archaeosine Biosynthesis	Epoxyqueuosine (oQ) reductase QueG
RNA Metabolism	RNA processing and modification	Queuosine-Archaeosine Biosynthesis	NADPH-dependent 7-cyano-7-deazaguanine reductase (EC 1.7.1.-)
RNA Metabolism	Transcription	Transcription factors bacterial	Regulator of nucleoside diphosphate kinase
Stress Response	Cold shock	Cold shock, CspA family of proteins	Cold shock protein CspC
Stress Response	Cold shock	Cold shock, CspA family of proteins	Cold shock protein CspG
Stress Response	Detoxification	Glutathione-dependent pathway of formaldehyde detoxification	S-formylglutathione hydrolase (EC 3.1.2.12)
Stress Response	no subcategory	Universal stress protein family	Universal stress protein family
Stress Response	no subcategory	Universal stress protein family	Universal stress protein family 4
Stress Response	Osmotic stress	Betaine biosynthesis from glycine	Dimethylglycine N-methyltransferase
Stress Response	Osmotic stress	Betaine biosynthesis from glycine	Glycine N-methyltransferase (EC 2.1.1.20)
Stress Response	Osmotic stress	Betaine biosynthesis from glycine	Sarcosine N-methyltransferase
Stress Response	Oxidative stress	Glutaredoxins	Glutaredoxin
Stress Response	Oxidative stress	Glutathione: Biosynthesis and gamma-glutamyl cycle	Similar to 5-oxoprolinase (EC 3.5.2.9) and Methylhydantoinases A, B (EC 3.5.2.14), contradiction in experimental data (see Annotation)
Stress Response	Oxidative stress	Glutathione: Non-redox reactions	Distant similarity with leukotriene C4 synthase (microsomal glutathione S-transferase)
Stress Response	Oxidative stress	Glutathione: Non-redox reactions	Glutathione S-transferase, omega (EC 2.5.1.18)
Stress Response	Oxidative stress	Glutathione: Redox cycle	Glutathione peroxidase (EC 1.11.1.9)
Stress Response	Oxidative stress	Rubrerythrin	Hypothetical protein in Rubrerythrin cluster
Sulfur Metabolism	Inorganic sulfur assimilation	Inorganic Sulfur Assimilation	Ferredoxin
Virulence, Disease and Defense	Resistance to antibiotics and toxic compounds	Arsenic resistance	Arsenical-resistance protein ACR3
Virulence, Disease and Defense	Resistance to antibiotics and toxic compounds	Beta-lactamase	Beta-lactamase (EC 3.5.2.6)

Virulence, Disease and Defense	Resistance to antibiotics and toxic compounds	Cobalt-zinc-cadmium resistance	Heavy metal RND efflux outer membrane protein, CzcC family
Virulence, Disease and Defense	Resistance to antibiotics and toxic compounds	Cobalt-zinc-cadmium resistance	Non-specific DNA-binding protein Dps / Iron-binding ferritin-like antioxidant protein / Ferroxidase (EC 1.16.3.1)
Virulence, Disease and Defense	Resistance to antibiotics and toxic compounds	Copper homeostasis	Multidrug resistance transporter, Bcr/CflA family
Virulence, Disease and Defense	Resistance to antibiotics and toxic compounds	Multidrug efflux pump in Campylobacter jejuni (CmeABC operon)	Transcriptional repressor of CmeABC operon, CmeR
Virulence, Disease and Defense	Resistance to antibiotics and toxic compounds	Multidrug Resistance Efflux Pumps	Macrolide export ATP-binding/permease protein MacB (EC 3.6.3.-)
Virulence, Disease and Defense	Resistance to antibiotics and toxic compounds	Multidrug Resistance Efflux Pumps	Macrolide-specific efflux protein MacA
Virulence, Disease and Defense	Resistance to antibiotics and toxic compounds	Multidrug Resistance Efflux Pumps	Multidrug and toxin extrusion (MATE) family efflux pump YdhE/NorM, homolog
Virulence, Disease and Defense	Resistance to antibiotics and toxic compounds	Multidrug Resistance Efflux Pumps	RND efflux system, inner membrane transporter CmeB
Virulence, Disease and Defense	Resistance to antibiotics and toxic compounds	Multidrug Resistance Efflux Pumps	RND efflux system, outer membrane lipoprotein CmeC
Virulence, Disease and Defense	Resistance to antibiotics and toxic compounds	Multidrug Resistance Efflux Pumps	RND efflux system, outer membrane lipoprotein, NodT family
Virulence, Disease and Defense	Resistance to antibiotics and toxic compounds	Multidrug Resistance, Tripartite Systems Found in Gram Negative Bacteria	Inner membrane component of tripartite multidrug resistance system
Virulence, Disease and Defense	Resistance to antibiotics and toxic compounds	Multidrug Resistance, Tripartite Systems Found in Gram Negative Bacteria	Membrane fusion component of tripartite multidrug resistance system
Virulence, Disease and Defense	Resistance to antibiotics and toxic compounds	Multidrug Resistance, Tripartite Systems Found in Gram Negative Bacteria	Outer membrane component of tripartite multidrug resistance system

Shared between "*Ca.* P. ectocarpi" and "P. lamentivorans"

Amino Acids and Derivatives	Alanine, serine, and glycine	Alanine biosynthesis	Alanine racemase (EC 5.1.1.1)
Amino Acids and Derivatives	Alanine, serine, and glycine	Alanine biosynthesis	Cysteine desulfurase (EC 2.8.1.7)
Amino Acids and Derivatives	Alanine, serine, and glycine	Alanine biosynthesis	Cysteine desulfurase (EC 2.8.1.7), SufS subfamily
Amino Acids and Derivatives	Alanine, serine, and glycine	Alanine biosynthesis	Ferredoxin, 2Fe-2S
Amino Acids and Derivatives	Alanine, serine, and glycine	Alanine biosynthesis	Iron-sulfur cluster regulator IscR
Amino Acids and Derivatives	Alanine, serine, and glycine	Glycine Biosynthesis	Serine hydroxymethyltransferase (EC 2.1.2.1)
Amino Acids and Derivatives	Alanine, serine, and glycine	Glycine and Serine Utilization	Aminomethyltransferase (glycine cleavage system T protein) (EC 2.1.2.10)
Amino Acids and Derivatives	Alanine, serine, and glycine	Glycine and Serine Utilization	D-3-phosphoglycerate dehydrogenase (EC 1.1.1.95)
Amino Acids and Derivatives	Alanine, serine, and glycine	Glycine and Serine Utilization	Glycine cleavage system H protein
Amino Acids and Derivatives	Alanine, serine, and glycine	Glycine and Serine Utilization	Glycine dehydrogenase [decarboxylating] (glycine cleavage system P1 protein) (EC 1.4.4.2)
Amino Acids and Derivatives	Alanine, serine, and glycine	Glycine and Serine Utilization	Glycine dehydrogenase [decarboxylating] (glycine cleavage system P2 protein) (EC 1.4.4.2)
Amino Acids and Derivatives	Alanine, serine, and glycine	Glycine and Serine Utilization	Phosphoserine aminotransferase (EC 2.6.1.52)
Amino Acids and Derivatives	Alanine, serine, and glycine	Glycine and Serine Utilization	Phosphoserine phosphatase (EC 3.1.3.3)
Amino Acids and Derivatives	Alanine, serine, and glycine	Glycine and Serine Utilization	Serine hydroxymethyltransferase (EC 2.1.2.1)
Amino Acids and Derivatives	Alanine, serine, and glycine	Glycine and Serine Utilization	Seryl-tRNA synthetase (EC 6.1.1.11)
Amino Acids and Derivatives	Alanine, serine, and glycine	Glycine and Serine Utilization	Threonine dehydratase, catabolic (EC 4.3.1.19)
Amino Acids and Derivatives	Alanine, serine, and glycine	Glycine cleavage system	Aminomethyltransferase (glycine cleavage system T protein) (EC 2.1.2.10)
Amino Acids and Derivatives	Alanine, serine, and glycine	Glycine cleavage system	Glycine cleavage system H protein
Amino Acids and Derivatives	Alanine, serine, and glycine	Glycine cleavage system	Glycine dehydrogenase [decarboxylating] (glycine cleavage system P1 protein) (EC 1.4.4.2)
Amino Acids and Derivatives	Alanine, serine, and glycine	Glycine cleavage system	Glycine dehydrogenase [decarboxylating] (glycine cleavage system P2 protein) (EC 1.4.4.2)
Amino Acids and Derivatives	Alanine, serine, and glycine	Serine Biosynthesis	D-3-phosphoglycerate dehydrogenase (EC 1.1.1.95)
Amino Acids and Derivatives	Alanine, serine, and glycine	Serine Biosynthesis	Phosphoserine aminotransferase (EC 2.6.1.52)
Amino Acids and Derivatives	Alanine, serine, and glycine	Serine Biosynthesis	Phosphoserine phosphatase (EC 3.1.3.3)
Amino Acids and Derivatives	Alanine, serine, and glycine	Serine Biosynthesis	Serine hydroxymethyltransferase (EC 2.1.2.1)
Amino Acids and Derivatives	Arginine; urea cycle, polyamines	Arginine Biosynthesis -- gjo	Acetylglutamate kinase (EC 2.7.2.8)
Amino Acids and Derivatives	Arginine; urea cycle, polyamines	Arginine Biosynthesis -- gjo	Acetylornithine aminotransferase (EC 2.6.1.11)
Amino Acids and Derivatives	Arginine; urea cycle, polyamines	Arginine Biosynthesis -- gjo	Acetylornithine deacetylase (EC 3.5.1.16)
Amino Acids and Derivatives	Arginine; urea cycle, polyamines	Arginine Biosynthesis -- gjo	Argininosuccinate lyase (EC 4.3.2.1)
Amino Acids and Derivatives	Arginine; urea cycle, polyamines	Arginine Biosynthesis -- gjo	Argininosuccinate synthase (EC 6.3.4.5)
Amino Acids and Derivatives	Arginine; urea cycle, polyamines	Arginine Biosynthesis -- gjo	Glutamate N-acetyltransferase (EC 2.3.1.35)
Amino Acids and Derivatives	Arginine; urea cycle, polyamines	Arginine Biosynthesis -- gjo	N-acetyl-gamma-glutamyl-phosphate reductase (EC 1.2.1.38)
Amino Acids and Derivatives	Arginine; urea cycle, polyamines	Arginine Biosynthesis -- gjo	N-acetylglutamate synthase (EC 2.3.1.1)
Amino Acids and Derivatives	Arginine; urea cycle, polyamines	Arginine Biosynthesis -- gjo	N-succinyl-L,L-diaminopimelate desuccinylase (EC 3.5.1.18)
Amino Acids and Derivatives	Arginine; urea cycle, polyamines	Arginine Biosynthesis -- gjo	Ornithine carbamoyltransferase (EC 2.1.3.3)
Amino Acids and Derivatives	Arginine; urea cycle, polyamines	Arginine Biosynthesis extended	Acetylglutamate kinase (EC 2.7.2.8)
Amino Acids and Derivatives	Arginine; urea cycle, polyamines	Arginine Biosynthesis extended	Acetylornithine aminotransferase (EC 2.6.1.11)
Amino Acids and Derivatives	Arginine; urea cycle, polyamines	Arginine Biosynthesis extended	Acetylornithine deacetylase (EC 3.5.1.16)
Amino Acids and Derivatives	Arginine; urea cycle, polyamines	Arginine Biosynthesis extended	Argininosuccinate lyase (EC 4.3.2.1)
Amino Acids and Derivatives	Arginine; urea cycle, polyamines	Arginine Biosynthesis extended	Argininosuccinate synthase (EC 6.3.4.5)
Amino Acids and Derivatives	Arginine; urea cycle, polyamines	Arginine Biosynthesis extended	Glutamate N-acetyltransferase (EC 2.3.1.35)
Amino Acids and Derivatives	Arginine; urea cycle, polyamines	Arginine Biosynthesis extended	N-acetyl-gamma-glutamyl-phosphate reductase (EC 1.2.1.38)
Amino Acids and Derivatives	Arginine; urea cycle, polyamines	Arginine Biosynthesis extended	N-acetylglutamate synthase (EC 2.3.1.1)
Amino Acids and Derivatives	Arginine; urea cycle, polyamines	Arginine Biosynthesis extended	N-succinyl-L,L-diaminopimelate desuccinylase (EC 3.5.1.18)
Amino Acids and Derivatives	Arginine; urea cycle, polyamines	Arginine Biosynthesis extended	Ornithine carbamoyltransferase (EC 2.1.3.3)
Amino Acids and Derivatives	Arginine; urea cycle, polyamines	Arginine and Ornithine Degradation	Agmatine deiminase (EC 3.5.3.12)
Amino Acids and Derivatives	Arginine; urea cycle, polyamines	Arginine and Ornithine Degradation	Arginine decarboxylase (EC 4.1.1.19)
Amino Acids and Derivatives	Arginine; urea cycle, polyamines	Arginine and Ornithine Degradation	NADP-specific glutamate dehydrogenase (EC 1.4.1.4)
Amino Acids and Derivatives	Arginine; urea cycle, polyamines	Arginine and Ornithine Degradation	Ornithine carbamoyltransferase (EC 2.1.3.3)
Amino Acids and Derivatives	Arginine; urea cycle, polyamines	Arginine and Ornithine Degradation	Ornithine decarboxylase (EC 4.1.1.17)
Amino Acids and Derivatives	Arginine; urea cycle, polyamines	Polyamine Metabolism	ABC transporter, periplasmic spermidine putrescine-binding protein PotD (TC 3.A.1.11.1)
Amino Acids and Derivatives	Arginine; urea cycle, polyamines	Polyamine Metabolism	Agmatine deiminase (EC 3.5.3.12)
Amino Acids and Derivatives	Arginine; urea cycle, polyamines	Polyamine Metabolism	Arginine decarboxylase (EC 4.1.1.19)

Amino Acids and Derivatives	Arginine; urea cycle, polyamines	Polyamine Metabolism	Ornithine decarboxylase (EC 4.1.1.17)
Amino Acids and Derivatives	Arginine; urea cycle, polyamines	Polyamine Metabolism	Spermidine Putrescine ABC transporter permease component PotB (TC 3.A.1.11.1)
Amino Acids and Derivatives	Arginine; urea cycle, polyamines	Polyamine Metabolism	Spermidine Putrescine ABC transporter permease component potC (TC_3.A.1.11.1)
Amino Acids and Derivatives	Aromatic amino acids and derivatives	Chorismate: Intermediate for synthesis of Tryptophan, PAPA antibiotics, PABA, 3-hydroxyanthranilate and more.	Aminodeoxychorismate lyase (EC 4.1.3.38)
Amino Acids and Derivatives	Aromatic amino acids and derivatives	Chorismate: Intermediate for synthesis of Tryptophan, PAPA antibiotics, PABA, 3-hydroxyanthranilate and more.	Anthranilate phosphoribosyltransferase (EC 2.4.2.18)
Amino Acids and Derivatives	Aromatic amino acids and derivatives	Chorismate: Intermediate for synthesis of Tryptophan, PAPA antibiotics, PABA, 3-hydroxyanthranilate and more.	Anthranilate synthase, amidotransferase component (EC 4.1.3.27)
Amino Acids and Derivatives	Aromatic amino acids and derivatives	Chorismate: Intermediate for synthesis of Tryptophan, PAPA antibiotics, PABA, 3-hydroxyanthranilate and more.	Anthranilate synthase, aminase component (EC 4.1.3.27)
Amino Acids and Derivatives	Aromatic amino acids and derivatives	Chorismate: Intermediate for synthesis of Tryptophan, PAPA antibiotics, PABA, 3-hydroxyanthranilate and more.	Indole-3-glycerol phosphate synthase (EC 4.1.1.48)
Amino Acids and Derivatives	Aromatic amino acids and derivatives	Chorismate: Intermediate for synthesis of Tryptophan, PAPA antibiotics, PABA, 3-hydroxyanthranilate and more.	Isochorismate pyruvate-lyase (EC 4.-.-.-)
Amino Acids and Derivatives	Aromatic amino acids and derivatives	Chorismate: Intermediate for synthesis of Tryptophan, PAPA antibiotics, PABA, 3-hydroxyanthranilate and more.	Para-aminobenzoate synthase, amidotransferase component (EC 2.6.1.85)
Amino Acids and Derivatives	Aromatic amino acids and derivatives	Chorismate: Intermediate for synthesis of Tryptophan, PAPA antibiotics, PABA, 3-hydroxyanthranilate and more.	Para-aminobenzoate synthase, aminase component (EC 2.6.1.85)
Amino Acids and Derivatives	Aromatic amino acids and derivatives	Chorismate: Intermediate for synthesis of Tryptophan, PAPA antibiotics, PABA, 3-hydroxyanthranilate and more.	Phosphoribosylanthranilate isomerase (EC 5.3.1.24)
Amino Acids and Derivatives	Aromatic amino acids and derivatives	Chorismate: Intermediate for synthesis of Tryptophan, PAPA antibiotics, PABA, 3-hydroxyanthranilate and more.	Phosphoribosylformimino-5-aminoimidazole carboxamide ribotide isomerase (EC 5.3.1.16)
Amino Acids and Derivatives	Aromatic amino acids and derivatives	Chorismate: Intermediate for synthesis of Tryptophan, PAPA antibiotics, PABA, 3-hydroxyanthranilate and more.	Tryptophan synthase alpha chain (EC 4.2.1.20)
Amino Acids and Derivatives	Aromatic amino acids and derivatives	Chorismate: Intermediate for synthesis of Tryptophan, PAPA antibiotics, PABA, 3-hydroxyanthranilate and more.	Tryptophan synthase beta chain (EC 4.2.1.20)
Amino Acids and Derivatives	Aromatic amino acids and derivatives	Chorismate Synthesis	2-keto-3-deoxy-D-arabino-heptulosonate-7-phosphate synthase II (EC 2.5.1.54)
Amino Acids and Derivatives	Aromatic amino acids and derivatives	Chorismate Synthesis	3-dehydroquinate dehydratase II (EC 4.2.1.10)
Amino Acids and Derivatives	Aromatic amino acids and derivatives	Chorismate Synthesis	3-dehydroquinate synthase (EC 4.2.3.4)
Amino Acids and Derivatives	Aromatic amino acids and derivatives	Chorismate Synthesis	5-Enolpyruvylshikimate-3-phosphate synthase (EC 2.5.1.19)
Amino Acids and Derivatives	Aromatic amino acids and derivatives	Chorismate Synthesis	Chorismate mutase I (EC 5.4.99.5)
Amino Acids and Derivatives	Aromatic amino acids and derivatives	Chorismate Synthesis	Chorismate synthase (EC 4.2.3.5)
Amino Acids and Derivatives	Aromatic amino acids and derivatives	Chorismate Synthesis	Cyclohexadienyl dehydrogenase (EC 1.3.1.12)(EC 1.3.1.43)
Amino Acids and Derivatives	Aromatic amino acids and derivatives	Chorismate Synthesis	Prephenate dehydratase (EC 4.2.1.51)
Amino Acids and Derivatives	Aromatic amino acids and derivatives	Chorismate Synthesis	Shikimate 5-dehydrogenase I alpha (EC 1.1.1.25)
Amino Acids and Derivatives	Aromatic amino acids and derivatives	Chorismate Synthesis	Shikimate kinase I (EC 2.7.1.71)
Amino Acids and Derivatives	Aromatic amino acids and derivatives	Common Pathway For Synthesis of Aromatic Compounds (DAHP synthase to chorismate)	2-keto-3-deoxy-D-arabino-heptulosonate-7-phosphate synthase II (EC 2.5.1.54)
Amino Acids and Derivatives	Aromatic amino acids and derivatives	Common Pathway For Synthesis of Aromatic Compounds (DAHP synthase to chorismate)	3-dehydroquinate dehydratase II (EC 4.2.1.10)
Amino Acids and Derivatives	Aromatic amino acids and derivatives	Common Pathway For Synthesis of Aromatic Compounds (DAHP synthase to chorismate)	3-dehydroquinate synthase (EC 4.2.3.4)
Amino Acids and Derivatives	Aromatic amino acids and derivatives	Common Pathway For Synthesis of Aromatic Compounds (DAHP synthase to chorismate)	5-Enolpyruvylshikimate-3-phosphate synthase (EC 2.5.1.19)
Amino Acids and Derivatives	Aromatic amino acids and derivatives	Common Pathway For Synthesis of Aromatic Compounds (DAHP synthase to chorismate)	Chorismate synthase (EC 4.2.3.5)
Amino Acids and Derivatives	Aromatic amino acids and derivatives	Common Pathway For Synthesis of Aromatic Compounds (DAHP synthase to chorismate)	Shikimate 5-dehydrogenase I alpha (EC 1.1.1.25)
Amino Acids and Derivatives	Aromatic amino acids and derivatives	Common Pathway For Synthesis of Aromatic Compounds (DAHP synthase to chorismate)	Shikimate kinase I (EC 2.7.1.71)
Amino Acids and Derivatives	Aromatic amino acids and derivatives	Phenylalanine and Tyrosine Branches from Chorismate	Biosynthetic Aromatic amino acid aminotransferase beta (EC 2.6.1.57)
Amino Acids and Derivatives	Aromatic amino acids and derivatives	Phenylalanine and Tyrosine Branches from Chorismate	Chorismate mutase I (EC 5.4.99.5)
Amino Acids and Derivatives	Aromatic amino acids and derivatives	Phenylalanine and Tyrosine Branches from Chorismate	Cyclohexadienyl dehydrogenase (EC 1.3.1.12)(EC 1.3.1.43)
Amino Acids and Derivatives	Aromatic amino acids and derivatives	Phenylalanine and Tyrosine Branches from Chorismate	Prephenate dehydratase (EC 4.2.1.51)
Amino Acids and Derivatives	Aromatic amino acids and derivatives	Tryptophan synthesis	Aminodeoxychorismate lyase (EC 4.1.3.38)
Amino Acids and Derivatives	Aromatic amino acids and derivatives	Tryptophan synthesis	Anthranilate phosphoribosyltransferase (EC 2.4.2.18)
Amino Acids and Derivatives	Aromatic amino acids and derivatives	Tryptophan synthesis	Anthranilate synthase, amidotransferase component (EC 4.1.3.27)
Amino Acids and Derivatives	Aromatic amino acids and derivatives	Tryptophan synthesis	Anthranilate synthase, aminase component (EC 4.1.3.27)
Amino Acids and Derivatives	Aromatic amino acids and derivatives	Tryptophan synthesis	Indole-3-glycerol phosphate synthase (EC 4.1.1.48)
Amino Acids and Derivatives	Aromatic amino acids and derivatives	Tryptophan synthesis	Para-aminobenzoate synthase, amidotransferase component (EC 2.6.1.85)
Amino Acids and Derivatives	Aromatic amino acids and derivatives	Tryptophan synthesis	Para-aminobenzoate synthase, aminase component (EC 2.6.1.85)
Amino Acids and Derivatives	Aromatic amino acids and derivatives	Tryptophan synthesis	Phosphoribosylanthranilate isomerase (EC 5.3.1.24)

Amino Acids and Derivatives	Aromatic amino acids and derivatives	Tryptophan synthesis	Tryptophan synthase alpha chain (EC 4.2.1.20)
Amino Acids and Derivatives	Aromatic amino acids and derivatives	Tryptophan synthesis	Tryptophan synthase beta chain (EC 4.2.1.20)
Amino Acids and Derivatives	Branched-chain amino acids	Branched-Chain Amino Acid Biosynthesis	(R)-citramalate synthase (EC 2.3.1.182)
Amino Acids and Derivatives	Branched-chain amino acids	Branched-Chain Amino Acid Biosynthesis	2-isopropylmalate synthase (EC 2.3.3.13)
Amino Acids and Derivatives	Branched-chain amino acids	Branched-Chain Amino Acid Biosynthesis	3-isopropylmalate dehydratase large subunit (EC 4.2.1.33)
Amino Acids and Derivatives	Branched-chain amino acids	Branched-Chain Amino Acid Biosynthesis	3-isopropylmalate dehydratase small subunit (EC 4.2.1.33)
Amino Acids and Derivatives	Branched-chain amino acids	Branched-Chain Amino Acid Biosynthesis	3-isopropylmalate dehydrogenase (EC 1.1.1.85)
Amino Acids and Derivatives	Branched-chain amino acids	Branched-Chain Amino Acid Biosynthesis	Acetolactate synthase large subunit (EC 2.2.1.6)
Amino Acids and Derivatives	Branched-chain amino acids	Branched-Chain Amino Acid Biosynthesis	Acetolactate synthase small subunit (EC 2.2.1.6)
Amino Acids and Derivatives	Branched-chain amino acids	Branched-Chain Amino Acid Biosynthesis	Dihydroxy-acid dehydratase (EC 4.2.1.9)
Amino Acids and Derivatives	Branched-chain amino acids	Branched-Chain Amino Acid Biosynthesis	Ketol-acid reductoisomerase (EC 1.1.1.86)
Amino Acids and Derivatives	Branched-chain amino acids	Branched-Chain Amino Acid Biosynthesis	Threonine dehydratase, catabolic (EC 4.3.1.19)
Amino Acids and Derivatives	Branched-chain amino acids	Leucine Biosynthesis	2-isopropylmalate synthase (EC 2.3.3.13)
Amino Acids and Derivatives	Branched-chain amino acids	Leucine Biosynthesis	3-isopropylmalate dehydratase large subunit (EC 4.2.1.33)
Amino Acids and Derivatives	Branched-chain amino acids	Leucine Biosynthesis	3-isopropylmalate dehydratase small subunit (EC 4.2.1.33)
Amino Acids and Derivatives	Branched-chain amino acids	Leucine Biosynthesis	3-isopropylmalate dehydrogenase (EC 1.1.1.85)
Amino Acids and Derivatives	Glutamine, glutamate, aspartate, asparagine; ammonia assimilation	Glutamate dehydrogenases	NAD-specific glutamate dehydrogenase (EC 1.4.1.2)
Amino Acids and Derivatives	Glutamine, glutamate, aspartate, asparagine; ammonia assimilation	Glutamate dehydrogenases	NADP-specific glutamate dehydrogenase (EC 1.4.1.4)
Amino Acids and Derivatives	Glutamine, glutamate, aspartate, asparagine; ammonia assimilation	Glutamine, Glutamate, Aspartate and Asparagine Biosynthesis	Asparagine synthetase [glutamine-hydrolyzing] (EC 6.3.5.4)
Amino Acids and Derivatives	Glutamine, glutamate, aspartate, asparagine; ammonia assimilation	Glutamine, Glutamate, Aspartate and Asparagine Biosynthesis	Aspartate aminotransferase (EC 2.6.1.1)
Amino Acids and Derivatives	Glutamine, glutamate, aspartate, asparagine; ammonia assimilation	Glutamine, Glutamate, Aspartate and Asparagine Biosynthesis	Ferredoxin-dependent glutamate synthase (EC 1.4.7.1)
Amino Acids and Derivatives	Glutamine, glutamate, aspartate, asparagine; ammonia assimilation	Glutamine, Glutamate, Aspartate and Asparagine Biosynthesis	Glutamate racemase (EC 5.1.1.3)
Amino Acids and Derivatives	Glutamine, glutamate, aspartate, asparagine; ammonia assimilation	Glutamine, Glutamate, Aspartate and Asparagine Biosynthesis	Glutamate synthase [NADPH] large chain (EC 1.4.1.13)
Amino Acids and Derivatives	Glutamine, glutamate, aspartate, asparagine; ammonia assimilation	Glutamine, Glutamate, Aspartate and Asparagine Biosynthesis	Glutamate synthase [NADPH] small chain (EC 1.4.1.13)
Amino Acids and Derivatives	Glutamine, glutamate, aspartate, asparagine; ammonia assimilation	Glutamine, Glutamate, Aspartate and Asparagine Biosynthesis	Glutamine synthetase type I (EC 6.3.1.2)
Amino Acids and Derivatives	Glutamine, glutamate, aspartate, asparagine; ammonia assimilation	Glutamine, Glutamate, Aspartate and Asparagine Biosynthesis	NAD-specific glutamate dehydrogenase (EC 1.4.1.2)
Amino Acids and Derivatives	Glutamine, glutamate, aspartate, asparagine; ammonia assimilation	Glutamine, Glutamate, Aspartate and Asparagine Biosynthesis	NADP-specific glutamate dehydrogenase (EC 1.4.1.4)
Amino Acids and Derivatives	Glutamine, glutamate, aspartate, asparagine; ammonia assimilation	Glutamine synthetases	Glutamine synthetase type I (EC 6.3.1.2)
Amino Acids and Derivatives	Histidine Metabolism	Histidine Biosynthesis	ATP phosphoribosyltransferase (EC 2.4.2.17)
Amino Acids and Derivatives	Histidine Metabolism	Histidine Biosynthesis	ATP phosphoribosyltransferase regulatory subunit (EC 2.4.2.17)
Amino Acids and Derivatives	Histidine Metabolism	Histidine Biosynthesis	Histidinol dehydrogenase (EC 1.1.1.23)
Amino Acids and Derivatives	Histidine Metabolism	Histidine Biosynthesis	Histidinol-phosphate aminotransferase (EC 2.6.1.9)
Amino Acids and Derivatives	Histidine Metabolism	Histidine Biosynthesis	Imidazole glycerol phosphate synthase amidotransferase subunit (EC 2.4.2.-)
Amino Acids and Derivatives	Histidine Metabolism	Histidine Biosynthesis	Imidazole glycerol phosphate synthase cyclase subunit (EC 4.1.3.-)
Amino Acids and Derivatives	Histidine Metabolism	Histidine Biosynthesis	Imidazoleglycerol-phosphate dehydratase (EC 4.2.1.19)
Amino Acids and Derivatives	Histidine Metabolism	Histidine Biosynthesis	Phosphoribosyl-AMP cyclohydrolase (EC 3.5.4.19)
Amino Acids and Derivatives	Histidine Metabolism	Histidine Biosynthesis	Phosphoribosyl-ATP pyrophosphatase (EC 3.6.1.31)
Amino Acids and Derivatives	Histidine Metabolism	Histidine Biosynthesis	Phosphoribosylformimino-5-aminoimidazole carboxamide ribotide isomerase (EC 5.3.1.16)
Amino Acids and Derivatives	Lysine, threonine, methionine, and cysteine	Cysteine Biosynthesis	Adenylylsulfate kinase (EC 2.7.1.25)
Amino Acids and Derivatives	Lysine, threonine, methionine, and cysteine	Cysteine Biosynthesis	Cysteine synthase (EC 2.5.1.47)
Amino Acids and Derivatives	Lysine, threonine, methionine, and cysteine	Cysteine Biosynthesis	Phosphoadenylyl-sulfate reductase [thioredoxin] (EC 1.8.4.8)
Amino Acids and Derivatives	Lysine, threonine, methionine, and cysteine	Cysteine Biosynthesis	Serine acetyltransferase (EC 2.3.1.30)
Amino Acids and Derivatives	Lysine, threonine, methionine, and cysteine	Cysteine Biosynthesis	Sulfate adenylyltransferase subunit 1 (EC 2.7.7.4)
Amino Acids and Derivatives	Lysine, threonine, methionine, and cysteine	Cysteine Biosynthesis	Sulfate adenylyltransferase subunit 2 (EC 2.7.7.4)
Amino Acids and Derivatives	Lysine, threonine, methionine, and cysteine	Cysteine Biosynthesis	Sulfate permease
Amino Acids and Derivatives	Lysine, threonine, methionine, and cysteine	Cysteine Biosynthesis	Sulfite reductase [NADPH] hemoprotein beta-component (EC 1.8.1.2)
Amino Acids and Derivatives	Lysine, threonine, methionine, and cysteine	Lysine Biosynthesis DAP Pathway	2,3,4,5-tetrahydropyridine-2,6-dicarboxylate N-succinyltransferase (EC 2.3.1.117)
Amino Acids and Derivatives	Lysine, threonine, methionine, and cysteine	Lysine Biosynthesis DAP Pathway	4-hydroxy-tetrahydrodipicolinate reductase (EC 1.17.1.8)
Amino Acids and Derivatives	Lysine, threonine, methionine, and cysteine	Lysine Biosynthesis DAP Pathway	4-hydroxy-tetrahydrodipicolinate synthase (EC 4.3.3.7)
Amino Acids and Derivatives	Lysine, threonine, methionine, and cysteine	Lysine Biosynthesis DAP Pathway	Aspartate-semialdehyde dehydrogenase (EC 1.2.1.11)
Amino Acids and Derivatives	Lysine, threonine, methionine, and cysteine	Lysine Biosynthesis DAP Pathway	Aspartokinase (EC 2.7.2.4)
Amino Acids and Derivatives	Lysine, threonine, methionine, and cysteine	Lysine Biosynthesis DAP Pathway	Diaminopimelate decarboxylase (EC 4.1.1.20)
Amino Acids and Derivatives	Lysine, threonine, methionine, and cysteine	Lysine Biosynthesis DAP Pathway	Diaminopimelate epimerase (EC 5.1.1.7)
Amino Acids and Derivatives	Lysine, threonine, methionine, and cysteine	Lysine Biosynthesis DAP Pathway	N-succinyl-L,L-diaminopimelate aminotransferase alternative (EC 2.6.1.17)
Amino Acids and Derivatives	Lysine, threonine, methionine, and cysteine	Lysine Biosynthesis DAP Pathway	N-succinyl-L,L-diaminopimelate desuccinylase (EC 3.5.1.18)
Amino Acids and Derivatives	Lysine, threonine, methionine, and cysteine	Lysine Biosynthesis DAP Pathway, GJO scratch	2,3,4,5-tetrahydropyridine-2,6-dicarboxylate N-succinyltransferase (EC 2.3.1.117)
Amino Acids and Derivatives	Lysine, threonine, methionine, and cysteine	Lysine Biosynthesis DAP Pathway, GJO scratch	4-hydroxy-tetrahydrodipicolinate reductase (EC 1.17.1.8)

Amino Acids and Derivatives	Lysine, threonine, methionine, and cysteine	Lysine Biosynthesis DAP Pathway, GJO scratch	4-hydroxy-tetrahydrodipicolinate synthase (EC 4.3.3.7)
Amino Acids and Derivatives	Lysine, threonine, methionine, and cysteine	Lysine Biosynthesis DAP Pathway, GJO scratch	Aspartate-semialdehyde dehydrogenase (EC 1.2.1.11)
Amino Acids and Derivatives	Lysine, threonine, methionine, and cysteine	Lysine Biosynthesis DAP Pathway, GJO scratch	Aspartokinase (EC 2.7.2.4)
Amino Acids and Derivatives	Lysine, threonine, methionine, and cysteine	Lysine Biosynthesis DAP Pathway, GJO scratch	Diaminopimelate decarboxylase (EC 4.1.1.20)
Amino Acids and Derivatives	Lysine, threonine, methionine, and cysteine	Lysine Biosynthesis DAP Pathway, GJO scratch	Diaminopimelate epimerase (EC 5.1.1.7)
Amino Acids and Derivatives	Lysine, threonine, methionine, and cysteine	Lysine Biosynthesis DAP Pathway, GJO scratch	N-succinyl-L,L-diaminopimelate aminotransferase alternative (EC 2.6.1.17)
Amino Acids and Derivatives	Lysine, threonine, methionine, and cysteine	Lysine Biosynthesis DAP Pathway, GJO scratch	N-succinyl-L,L-diaminopimelate desuccinylase (EC 3.5.1.18)
Amino Acids and Derivatives	Lysine, threonine, methionine, and cysteine	Threonine and Homoserine Biosynthesis	Aspartate aminotransferase (EC 2.6.1.1)
Amino Acids and Derivatives	Lysine, threonine, methionine, and cysteine	Threonine and Homoserine Biosynthesis	Aspartate-semialdehyde dehydrogenase (EC 1.2.1.11)
Amino Acids and Derivatives	Lysine, threonine, methionine, and cysteine	Threonine and Homoserine Biosynthesis	Aspartokinase (EC 2.7.2.4)
Amino Acids and Derivatives	Lysine, threonine, methionine, and cysteine	Threonine and Homoserine Biosynthesis	Homoserine dehydrogenase (EC 1.1.1.3)
Amino Acids and Derivatives	Lysine, threonine, methionine, and cysteine	Threonine and Homoserine Biosynthesis	Homoserine kinase (EC 2.7.1.39)
Amino Acids and Derivatives	Lysine, threonine, methionine, and cysteine	Threonine and Homoserine Biosynthesis	Threonine synthase (EC 4.2.3.1)
Amino Acids and Derivatives	Lysine, threonine, methionine, and cysteine	Threonine degradation	FIG003492: Threonine dehydrogenase and related Zn-dependent dehydrogenases
Amino Acids and Derivatives	Lysine, threonine, methionine, and cysteine	Threonine degradation	Threonine dehydratase, catabolic (EC 4.3.1.19)
Amino Acids and Derivatives	Lysine, threonine, methionine, and cysteine	Threonine degradation	low-specificity D-threonine aldolase
Amino Acids and Derivatives	no subcategory	Creatine and Creatinine Degradation	Creatinine amidohydrolase (EC 3.5.2.10)
Carbohydrates	Central carbohydrate metabolism	Dehydrogenase complexes	2-oxoglutarate dehydrogenase E1 component (EC 1.2.4.2)
Carbohydrates	Central carbohydrate metabolism	Dehydrogenase complexes	Cytosol aminopeptidase PepA (EC 3.4.11.1)
Carbohydrates	Central carbohydrate metabolism	Dehydrogenase complexes	Dihydrolipoamide acetyltransferase component of pyruvate dehydrogenase complex (EC 2.3.1.12)
Carbohydrates	Central carbohydrate metabolism	Dehydrogenase complexes	Dihydrolipoamide dehydrogenase of 2-oxoglutarate dehydrogenase (EC 1.8.1.4)
Carbohydrates	Central carbohydrate metabolism	Dehydrogenase complexes	Dihydrolipoamide succinyltransferase component (E2) of 2-oxoglutarate dehydrogenase complex (EC 2.3.1.61)
Carbohydrates	Central carbohydrate metabolism	Dehydrogenase complexes	Pyruvate dehydrogenase E1 component alpha subunit (EC 1.2.4.1)
Carbohydrates	Central carbohydrate metabolism	Dehydrogenase complexes	Pyruvate dehydrogenase E1 component beta subunit (EC 1.2.4.1)
Carbohydrates	Central carbohydrate metabolism	Ethylmalonyl-CoA pathway of C2 assimilation	L-malyl-CoA/beta-methylmalyl-CoA lyase (EC 4.1.3.-)
Carbohydrates	Central carbohydrate metabolism	Ethylmalonyl-CoA pathway of C2 assimilation	Mesaconyl-CoA hydratase
Carbohydrates	Central carbohydrate metabolism	Ethylmalonyl-CoA pathway of C2 assimilation	Methylsuccinyl-CoA dehydrogenase, predicted by (Erb et al, 2007)
Carbohydrates	Central carbohydrate metabolism	Glycolate, glyoxylate interconversions	D-Lactate dehydrogenase, cytochrome c-dependent (EC 1.1.2.4)
Carbohydrates	Central carbohydrate metabolism	Glycolate, glyoxylate interconversions	Glyoxylate reductase (EC 1.1.1.26)
Carbohydrates	Central carbohydrate metabolism	Glycolate, glyoxylate interconversions	Glyoxylate reductase (EC 1.1.1.79)
Carbohydrates	Central carbohydrate metabolism	Glycolate, glyoxylate interconversions	Hydroxypyruvate reductase (EC 1.1.1.81)
Carbohydrates	Central carbohydrate metabolism	Glycolate, glyoxylate interconversions	Phosphoglycolate phosphatase (EC 3.1.3.18)
Carbohydrates	Central carbohydrate metabolism	Glycolysis and Gluconeogenesis	2,3-bisphosphoglycerate-independent phosphoglycerate mutase (EC 5.4.2.1)
Carbohydrates	Central carbohydrate metabolism	Glycolysis and Gluconeogenesis	6-phosphofructokinase (EC 2.7.1.11)
Carbohydrates	Central carbohydrate metabolism	Glycolysis and Gluconeogenesis	Enolase (EC 4.2.1.11)
Carbohydrates	Central carbohydrate metabolism	Glycolysis and Gluconeogenesis	Fructose-1,6-bisphosphatase, GlpX type (EC 3.1.3.11)
Carbohydrates	Central carbohydrate metabolism	Glycolysis and Gluconeogenesis	Glucose-6-phosphate isomerase (EC 5.3.1.9)
Carbohydrates	Central carbohydrate metabolism	Glycolysis and Gluconeogenesis	NAD-dependent glyceraldehyde-3-phosphate dehydrogenase (EC 1.2.1.12)
Carbohydrates	Central carbohydrate metabolism	Glycolysis and Gluconeogenesis	Phosphoglycerate kinase (EC 2.7.2.3)
Carbohydrates	Central carbohydrate metabolism	Glycolysis and Gluconeogenesis	Phosphoglycerate mutase (EC 5.4.2.1)
Carbohydrates	Central carbohydrate metabolism	Glycolysis and Gluconeogenesis	Pyruvate kinase (EC 2.7.1.40)
Carbohydrates	Central carbohydrate metabolism	Glycolysis and Gluconeogenesis	Pyruvate,phosphate dikinase (EC 2.7.9.1)
Carbohydrates	Central carbohydrate metabolism	Glycolysis and Gluconeogenesis	Triosephosphate isomerase (EC 5.3.1.1)
Carbohydrates	Central carbohydrate metabolism	Glyoxylate bypass	Aconitate hydratase (EC 4.2.1.3)
Carbohydrates	Central carbohydrate metabolism	Glyoxylate bypass	Citrate synthase (si) (EC 2.3.3.1)
Carbohydrates	Central carbohydrate metabolism	Glyoxylate bypass	Isocitrate lyase (EC 4.1.3.1)
Carbohydrates	Central carbohydrate metabolism	Glyoxylate bypass	Malate dehydrogenase (EC 1.1.1.37)
Carbohydrates	Central carbohydrate metabolism	Methylglyoxal Metabolism	Aldehyde dehydrogenase (EC 1.2.1.3)
Carbohydrates	Central carbohydrate metabolism	Methylglyoxal Metabolism	Hydroxyacylglutathione hydrolase (EC 3.1.2.6)
Carbohydrates	Central carbohydrate metabolism	Methylglyoxal Metabolism	Lactoylglutathione lyase (EC 4.4.1.5)
Carbohydrates	Central carbohydrate metabolism	Pentose phosphate pathway	Ribose 5-phosphate isomerase A (EC 5.3.1.6)
Carbohydrates	Central carbohydrate metabolism	Pentose phosphate pathway	Ribulose-phosphate 3-epimerase (EC 5.1.3.1)
Carbohydrates	Central carbohydrate metabolism	Pentose phosphate pathway	Transaldolase (EC 2.2.1.2)
Carbohydrates	Central carbohydrate metabolism	Pentose phosphate pathway	Transketolase (EC 2.2.1.1)
Carbohydrates	Central carbohydrate metabolism	Pyruvate Alanine Serine Interconversions	Alanine racemase (EC 5.1.1.1)
Carbohydrates	Central carbohydrate metabolism	Pyruvate Alanine Serine Interconversions	D-alanine aminotransferase (EC 2.6.1.21)
Carbohydrates	Central carbohydrate metabolism	Pyruvate Alanine Serine Interconversions	D-amino acid dehydrogenase small subunit (EC 1.4.99.1)
Carbohydrates	Central carbohydrate metabolism	Pyruvate metabolism I: anaplerotic reactions, PEP	NADP-dependent malic enzyme (EC 1.1.1.40)
Carbohydrates	Central carbohydrate metabolism	Pyruvate metabolism I: anaplerotic reactions, PEP	Phosphoenolpyruvate carboxykinase [ATP] (EC 4.1.1.49)
Carbohydrates	Central carbohydrate metabolism	Pyruvate metabolism I: anaplerotic reactions, PEP	Pyruvate kinase (EC 2.7.1.40)
Carbohydrates	Central carbohydrate metabolism	Pyruvate metabolism I: anaplerotic reactions, PEP	Pyruvate,phosphate dikinase (EC 2.7.9.1)
Carbohydrates	Central carbohydrate metabolism	Pyruvate metabolism II: acetyl-CoA, acetogenesis from pyruvate	Acetyl-CoA synthetase (ADP-forming) alpha and beta chains, putative
Carbohydrates	Central carbohydrate metabolism	Pyruvate metabolism II: acetyl-CoA, acetogenesis from pyruvate	Acetyl-coenzyme A synthetase (EC 6.2.1.1)
Carbohydrates	Central carbohydrate metabolism	Pyruvate metabolism II: acetyl-CoA, acetogenesis from pyruvate	Aldehyde dehydrogenase (EC 1.2.1.3)
Carbohydrates	Central carbohydrate metabolism	Pyruvate metabolism II: acetyl-CoA, acetogenesis from pyruvate	Dihydrolipoamide acetyltransferase component of pyruvate dehydrogenase complex (EC 2.3.1.12)

Carbohydrates	Central carbohydrate metabolism	Pyruvate metabolism II: acetyl-CoA, acetogenesis from pyruvate	Dihydrolipoamide dehydrogenase of pyruvate dehydrogenase complex (EC 1.8.1.4)
Carbohydrates	Central carbohydrate metabolism	Pyruvate metabolism II: acetyl-CoA, acetogenesis from pyruvate	NAD-dependent protein deacetylase of SIR2 family
Carbohydrates	Central carbohydrate metabolism	Pyruvate metabolism II: acetyl-CoA, acetogenesis from pyruvate	Pyruvate dehydrogenase E1 component alpha subunit (EC 1.2.4.1)
Carbohydrates	Central carbohydrate metabolism	Pyruvate metabolism II: acetyl-CoA, acetogenesis from pyruvate	Pyruvate dehydrogenase E1 component beta subunit (EC 1.2.4.1)
Carbohydrates	Central carbohydrate metabolism	TCA Cycle	2-oxoglutarate dehydrogenase E1 component (EC 1.2.4.2)
Carbohydrates	Central carbohydrate metabolism	TCA Cycle	Aconitate hydratase (EC 4.2.1.3)
Carbohydrates	Central carbohydrate metabolism	TCA Cycle	Citrate synthase (si) (EC 2.3.3.1)
Carbohydrates	Central carbohydrate metabolism	TCA Cycle	Dihydrolipoamide dehydrogenase of 2-oxoglutarate dehydrogenase (EC 1.8.1.4)
Carbohydrates	Central carbohydrate metabolism	TCA Cycle	Dihydrolipoamide dehydrogenase of pyruvate dehydrogenase complex (EC 1.8.1.4)
Carbohydrates	Central carbohydrate metabolism	TCA Cycle	Dihydrolipoamide succinyltransferase component (E2) of 2-oxoglutarate dehydrogenase complex (EC 2.3.1.61)
Carbohydrates	Central carbohydrate metabolism	TCA Cycle	Fumarate hydratase class II (EC 4.2.1.2)
Carbohydrates	Central carbohydrate metabolism	TCA Cycle	Isocitrate dehydrogenase [NADP] (EC 1.1.1.42)
Carbohydrates	Central carbohydrate metabolism	TCA Cycle	Malate dehydrogenase (EC 1.1.1.37)
Carbohydrates	Central carbohydrate metabolism	TCA Cycle	Succinate dehydrogenase flavoprotein subunit (EC 1.3.99.1)
Carbohydrates	Central carbohydrate metabolism	TCA Cycle	Succinate dehydrogenase iron-sulfur protein (EC 1.3.99.1)
Carbohydrates	Central carbohydrate metabolism	TCA Cycle	Succinyl-CoA ligase [ADP-forming] alpha chain (EC 6.2.1.5)
Carbohydrates	Central carbohydrate metabolism	TCA Cycle	Succinyl-CoA ligase [ADP-forming] beta chain (EC 6.2.1.5)
Carbohydrates	Di- and oligosaccharides	Trehalose Biosynthesis	Alpha,alpha-trehalose-phosphate synthase [UDP-forming] (EC 2.4.1.15)
Carbohydrates	Di- and oligosaccharides	Trehalose Biosynthesis	Glucoamylase (EC 3.2.1.3)
Carbohydrates	Di- and oligosaccharides	Trehalose Biosynthesis	Trehalose-6-phosphate phosphatase (EC 3.1.3.12)
Carbohydrates	Fermentation	Acetolactate synthase subunits	Acetolactate synthase large subunit (EC 2.2.1.6)
Carbohydrates	Fermentation	Acetolactate synthase subunits	Acetolactate synthase small subunit (EC 2.2.1.6)
Carbohydrates	Fermentation	Acetyl-CoA fermentation to Butyrate	3-hydroxyacyl-CoA dehydrogenase (EC 1.1.1.35)
Carbohydrates	Fermentation	Acetyl-CoA fermentation to Butyrate	3-hydroxybutyryl-CoA dehydrogenase (EC 1.1.1.157)
Carbohydrates	Fermentation	Acetyl-CoA fermentation to Butyrate	3-hydroxybutyryl-CoA epimerase (EC 5.1.2.3)
Carbohydrates	Fermentation	Acetyl-CoA fermentation to Butyrate	Acetyl-CoA acetyltransferase (EC 2.3.1.9)
Carbohydrates	Fermentation	Acetyl-CoA fermentation to Butyrate	Butyryl-CoA dehydrogenase (EC 1.3.8.1)
Carbohydrates	Fermentation	Acetyl-CoA fermentation to Butyrate	Electron transfer flavoprotein, alpha subunit
Carbohydrates	Fermentation	Acetyl-CoA fermentation to Butyrate	Electron transfer flavoprotein, beta subunit
Carbohydrates	Fermentation	Acetyl-CoA fermentation to Butyrate	Electron transfer flavoprotein-ubiquinone oxidoreductase (EC 1.5.5.1)
Carbohydrates	Fermentation	Acetyl-CoA fermentation to Butyrate	Enoyl-CoA hydratase (EC 4.2.1.17)
Carbohydrates	Fermentation	Butanol Biosynthesis	3-hydroxybutyryl-CoA dehydrogenase (EC 1.1.1.157)
Carbohydrates	Fermentation	Butanol Biosynthesis	Acetyl-CoA acetyltransferase (EC 2.3.1.9)
Carbohydrates	Fermentation	Butanol Biosynthesis	Butyryl-CoA dehydrogenase (EC 1.3.8.1)
Carbohydrates	Fermentation	Butanol Biosynthesis	Enoyl-CoA hydratase (EC 4.2.1.17)
Carbohydrates	Monosaccharides	D-gluconate and ketogluconates metabolism	Glucose dehydrogenase, PQQ-dependent (EC 1.1.5.2)
Carbohydrates	Monosaccharides	D-ribose utilization	Ribose 5-phosphate isomerase A (EC 5.3.1.6)
Carbohydrates	Monosaccharides	Mannose Metabolism	Mannose-1-phosphate guanylyltransferase (GDP) (EC 2.7.7.22)
Carbohydrates	Monosaccharides	Mannose Metabolism	Phosphomannomutase (EC 5.4.2.8)
Carbohydrates	One-carbon Metabolism	One-carbon metabolism by tetrahydropterines	5,10-methylenetetrahydrofolate reductase (EC 1.5.1.20)
Carbohydrates	One-carbon Metabolism	One-carbon metabolism by tetrahydropterines	5-formyltetrahydrofolate cyclo-ligase (EC 6.3.3.2)
Carbohydrates	One-carbon Metabolism	One-carbon metabolism by tetrahydropterines	Methenyltetrahydrofolate cyclohydrolase (EC 3.5.4.9)
Carbohydrates	One-carbon Metabolism	One-carbon metabolism by tetrahydropterines	Methylenetetrahydrofolate dehydrogenase (NADP+) (EC 1.5.1.5)
Carbohydrates	Sugar alcohols	Glycerol and Glycerol-3-phosphate Uptake and Utilization	Glycerol kinase (EC 2.7.1.30)
Carbohydrates	Sugar alcohols	Glycerol and Glycerol-3-phosphate Uptake and Utilization	Glycerol-3-phosphate dehydrogenase (EC 1.1.5.3)
Carbohydrates	Sugar alcohols	Glycerol and Glycerol-3-phosphate Uptake and Utilization	Glycerol-3-phosphate dehydrogenase [NAD(P)+] (EC 1.1.1.94)
Carbohydrates	Sugar alcohols	Glycerol and Glycerol-3-phosphate Uptake and Utilization	Glycerophosphoryl diester phosphodiesterase (EC 3.1.4.46)
Cell Division and Cell Cycle	no subcategory	Bacterial Cytoskeleton	Cell division protein DivIC (FtsB), stabilizes FtsL against RasP cleavage
Cell Division and Cell Cycle	no subcategory	Bacterial Cytoskeleton	Cell division protein FtsA
Cell Division and Cell Cycle	no subcategory	Bacterial Cytoskeleton	Cell division protein FtsI [Peptidoglycan synthetase] (EC 2.4.1.129)
Cell Division and Cell Cycle	no subcategory	Bacterial Cytoskeleton	Cell division protein FtsK
Cell Division and Cell Cycle	no subcategory	Bacterial Cytoskeleton	Cell division protein FtsL
Cell Division and Cell Cycle	no subcategory	Bacterial Cytoskeleton	Cell division protein FtsQ
Cell Division and Cell Cycle	no subcategory	Bacterial Cytoskeleton	Cell division protein FtsW
Cell Division and Cell Cycle	no subcategory	Bacterial Cytoskeleton	Cell division protein FtsZ (EC 3.4.24.-)
Cell Division and Cell Cycle	no subcategory	Bacterial Cytoskeleton	Cell division protein MraZ
Cell Division and Cell Cycle	no subcategory	Bacterial Cytoskeleton	Chromosome (plasmid) partitioning protein ParA
Cell Division and Cell Cycle	no subcategory	Bacterial Cytoskeleton	Chromosome (plasmid) partitioning protein ParB
Cell Division and Cell Cycle	no subcategory	Bacterial Cytoskeleton	Rod shape-determining protein MreB
Cell Division and Cell Cycle	no subcategory	Bacterial Cytoskeleton	Rod shape-determining protein MreC
Cell Division and Cell Cycle	no subcategory	Bacterial Cytoskeleton	Rod shape-determining protein MreD
Cell Division and Cell Cycle	no subcategory	Bacterial Cytoskeleton	Rod shape-determining protein RodA
Cell Division and Cell Cycle	no subcategory	Bacterial Cytoskeleton	Septum formation protein Maf
Cell Division and Cell Cycle	no subcategory	Macromolecular synthesis operon	Carbamoyl-phosphate synthase large chain (EC 6.3.5.5)
Cell Division and Cell Cycle	no subcategory	Macromolecular synthesis operon	Carbamoyl-phosphate synthase small chain (EC 6.3.5.5)
Cell Division and Cell Cycle	no subcategory	Macromolecular synthesis operon	DNA primase (EC 2.7.7.-)
Cell Division and Cell Cycle	no subcategory	Macromolecular synthesis operon	RNA polymerase sigma factor RpoD
Cell Division and Cell Cycle	no subcategory	Macromolecular synthesis operon	SSU ribosomal protein S21p

Cell Division and Cell Cycle	no subcategory	Macromolecular synthesis operon	Transamidase GatB domain protein
Cell Division and Cell Cycle	no subcategory	Macromolecular synthesis operon	TsaD/Kae1/Qri7 protein, required for threonylcarbamoyladenosine t(6)A37 formation in tRNA
Cell Division and Cell Cycle	no subcategory	YgjD and YeaZ	TsaB protein, required for threonylcarbamoyladenosine (t(6)A) formation in tRNA
Cell Division and Cell Cycle	no subcategory	YgjD and YeaZ	TsaD/Kae1/Qri7 protein, required for threonylcarbamoyladenosine t(6)A37 formation in tRNA
Cell Wall and Capsule	Capsular and extracellular polysacchrides	Capsular heptose biosynthesis	D-glycero-D-manno-heptose 1,7-bisphosphate phosphatase (EC 3.1.1.-)
Cell Wall and Capsule	Capsular and extracellular polysacchrides	Capsular heptose biosynthesis	Phosphoheptose isomerase 1 (EC 5.3.1.-)
Cell Wall and Capsule	Capsular and extracellular polysacchrides	Capsular heptose biosynthesis	dTDP-4-dehydrorhamnose 3,5-epimerase (EC 5.1.3.13)
Cell Wall and Capsule	Capsular and extracellular polysacchrides	Rhamnose containing glycans	UDP-glucose 4-epimerase (EC 5.1.3.2)
Cell Wall and Capsule	Capsular and extracellular polysacchrides	Rhamnose containing glycans	dTDP-4-dehydrorhamnose 3,5-epimerase (EC 5.1.3.13)
Cell Wall and Capsule	Capsular and extracellular polysacchrides	Rhamnose containing glycans	dTDP-glucose 4,6-dehydratase (EC 4.2.1.46)
Cell Wall and Capsule	Capsular and extracellular polysacchrides	dTDP-rhamnose synthesis	Glucose-1-phosphate cytidylyltransferase (EC 2.7.7.33)
Cell Wall and Capsule	Capsular and extracellular polysacchrides	dTDP-rhamnose synthesis	dTDP-4-dehydrorhamnose 3,5-epimerase (EC 5.1.3.13)
Cell Wall and Capsule	Capsular and extracellular polysacchrides	dTDP-rhamnose synthesis	dTDP-glucose 4,6-dehydratase (EC 4.2.1.46)
Cell Wall and Capsule	Gram-Negative cell wall components	LOS core oligosaccharide biosynthesis	ADP-heptose synthase (EC 2.7.-.-)
Cell Wall and Capsule	Gram-Negative cell wall components	LOS core oligosaccharide biosynthesis	ADP-heptose--lipooligosaccharide heptosyltransferase II (EC 2.4.1.-)
Cell Wall and Capsule	Gram-Negative cell wall components	LOS core oligosaccharide biosynthesis	D-glycero-D-manno-heptose 1,7-bisphosphate phosphatase (EC 3.1.1.-)
Cell Wall and Capsule	Gram-Negative cell wall components	LOS core oligosaccharide biosynthesis	D-glycero-beta-D-manno-heptose 7-phosphate kinase
Cell Wall and Capsule	Gram-Negative cell wall components	LOS core oligosaccharide biosynthesis	Phosphoheptose isomerase 1 (EC 5.3.1.-)
Cell Wall and Capsule	Gram-Negative cell wall components	Lipoprotein sorting system	Lipoprotein releasing system ATP-binding protein LolD
Cell Wall and Capsule	Gram-Negative cell wall components	Peptidoglycan lipid II flippase	Proposed peptidoglycan lipid II flippase MurJ
Cell Wall and Capsule	no subcategory	Murein Hydrolases	Beta N-acetyl-glucosaminidase (EC 3.2.1.52)
Cell Wall and Capsule	no subcategory	Murein Hydrolases	D-alanyl-D-alanine carboxypeptidase (EC 3.4.16.4)
Cell Wall and Capsule	no subcategory	Murein Hydrolases	Membrane-bound lytic murein transglycosylase A precursor (EC 3.2.1.-)
Cell Wall and Capsule	no subcategory	Murein Hydrolases	Membrane-bound lytic murein transglycosylase B precursor (EC 3.2.1.-)
Cell Wall and Capsule	no subcategory	Murein Hydrolases	N-acetylmuramoyl-L-alanine amidase (EC 3.5.1.28)
Cell Wall and Capsule	no subcategory	Murein Hydrolases	Soluble lytic murein transglycosylase precursor (EC 3.2.1.-)
Cell Wall and Capsule	no subcategory	Peptidoglycan Biosynthesis	Cell division protein FtsI [Peptidoglycan synthetase] (EC 2.4.1.129)
Cell Wall and Capsule	no subcategory	Peptidoglycan Biosynthesis	D-alanine--D-alanine ligase (EC 6.3.2.4)
Cell Wall and Capsule	no subcategory	Peptidoglycan Biosynthesis	D-alanyl-D-alanine carboxypeptidase (EC 3.4.16.4)
Cell Wall and Capsule	no subcategory	Peptidoglycan Biosynthesis	Glucosamine-1-phosphate N-acetyltransferase (EC 2.3.1.157)
Cell Wall and Capsule	no subcategory	Peptidoglycan Biosynthesis	Glutamate racemase (EC 5.1.1.3)
Cell Wall and Capsule	no subcategory	Peptidoglycan Biosynthesis	Glutamine synthetase type I (EC 6.3.1.2)
Cell Wall and Capsule	no subcategory	Peptidoglycan Biosynthesis	Membrane-bound lytic murein transglycosylase B precursor (EC 3.2.1.-)
Cell Wall and Capsule	no subcategory	Peptidoglycan Biosynthesis	Monofunctional biosynthetic peptidoglycan transglycosylase (EC 2.4.2.-)
Cell Wall and Capsule	no subcategory	Peptidoglycan Biosynthesis	Multimodular transpeptidase-transglycosylase (EC 2.4.1.129) (EC 3.4.-.-)
Cell Wall and Capsule	no subcategory	Peptidoglycan Biosynthesis	N-acetylglucosamine-1-phosphate uridyltransferase (EC 2.7.7.23)
Cell Wall and Capsule	no subcategory	Peptidoglycan Biosynthesis	Penicillin-binding protein 2 (PBP-2)
Cell Wall and Capsule	no subcategory	Peptidoglycan Biosynthesis	Phospho-N-acetylmuramoyl-pentapeptide-transferase (EC 2.7.8.13)
Cell Wall and Capsule	no subcategory	Peptidoglycan Biosynthesis	Rare lipoprotein A precursor
Cell Wall and Capsule	no subcategory	Peptidoglycan Biosynthesis	Rod shape-determining protein RodA
Cell Wall and Capsule	no subcategory	Peptidoglycan Biosynthesis	UDP-N-acetylenolpyruvoylglucosamine reductase (EC 1.1.1.158)
Cell Wall and Capsule	no subcategory	Peptidoglycan Biosynthesis	UDP-N-acetylglucosamine 1-carboxyvinyltransferase (EC 2.5.1.7)
Cell Wall and Capsule	no subcategory	Peptidoglycan Biosynthesis	UDP-N-acetylglucosamine--N-acetylmuramyl-(pentapeptide) pyrophosphoryl-undecaprenol N-acetylglucosamine transferase (EC 2.4.1.227)
Cell Wall and Capsule	no subcategory	Peptidoglycan Biosynthesis	UDP-N-acetylmuramate--alanine ligase (EC 6.3.2.8)
Cell Wall and Capsule	no subcategory	Peptidoglycan Biosynthesis	UDP-N-acetylmuramoylalanine--D-glutamate ligase (EC 6.3.2.9)
Cell Wall and Capsule	no subcategory	Peptidoglycan Biosynthesis	UDP-N-acetylmuramoylalanyl-D-glutamate--2,6-diaminopimelate ligase (EC 6.3.2.13)
Cell Wall and Capsule	no subcategory	Peptidoglycan Biosynthesis	UDP-N-acetylmuramoylalanyl-D-glutamyl-2,6-diaminopimelate--D-alanyl-D-alanine ligase (EC 6.3.2.10)
Cell Wall and Capsule	no subcategory	Peptidoglycan biosynthesis--gjo	D-alanine--D-alanine ligase (EC 6.3.2.4)
Cell Wall and Capsule	no subcategory	Peptidoglycan biosynthesis--gjo	UDP-N-acetylmuramate--alanine ligase (EC 6.3.2.8)
Cell Wall and Capsule	no subcategory	Peptidoglycan biosynthesis--gjo	UDP-N-acetylmuramoylalanine--D-glutamate ligase (EC 6.3.2.9)
Cell Wall and Capsule	no subcategory	Peptidoglycan biosynthesis--gjo	UDP-N-acetylmuramoylalanyl-D-glutamate--2,6-diaminopimelate ligase (EC 6.3.2.13)
Cell Wall and Capsule	no subcategory	Peptidoglycan biosynthesis--gjo	UDP-N-acetylmuramoylalanyl-D-glutamyl-2,6-diaminopimelate--D-alanyl-D-alanine ligase (EC 6.3.2.10)
Cell Wall and Capsule	no subcategory	Recycling of Peptidoglycan Amino Acids	N-acetylmuramoyl-L-alanine amidase (EC 3.5.1.28)
Cell Wall and Capsule	no subcategory	Recycling of Peptidoglycan Amino Sugars	Anhydro-N-acetylmuramic acid kinase (EC 2.7.1.-)
Cell Wall and Capsule	no subcategory	Recycling of Peptidoglycan Amino Sugars	Beta N-acetyl-glucosaminidase (EC 3.2.1.52)
Cell Wall and Capsule	no subcategory	UDP-N-acetylmuramate from Fructose-6-phosphate Biosynthesis	Glucosamine--fructose-6-phosphate aminotransferase [isomerizing] (EC 2.6.1.16)
Cell Wall and Capsule	no subcategory	UDP-N-acetylmuramate from Fructose-6-phosphate Biosynthesis	Glucosamine-1-phosphate N-acetyltransferase (EC 2.3.1.157)
Cell Wall and Capsule	no subcategory	UDP-N-acetylmuramate from Fructose-6-phosphate Biosynthesis	N-acetylglucosamine-1-phosphate uridyltransferase (EC 2.7.7.23)
Cell Wall and Capsule	no subcategory	UDP-N-acetylmuramate from Fructose-6-phosphate Biosynthesis	Phosphoglucosamine mutase (EC 5.4.2.10)

Cell Wall and Capsule	no subcategory	UDP-N-acetylmuramate from Fructose-6-phosphate Biosynthesis	UDP-N-acetylenolpyruvoylglucosamine reductase (EC 1.1.1.158)
Cell Wall and Capsule	no subcategory	UDP-N-acetylmuramate from Fructose-6-phosphate Biosynthesis	UDP-N-acetylglucosamine 1-carboxyvinyltransferase (EC 2.5.1.7)
Cell Wall and Capsule	no subcategory	YjeE	NAD(P)HX dehydratase
Cell Wall and Capsule	no subcategory	YjeE	NAD(P)HX epimerase
Cell Wall and Capsule	no subcategory	YjeE	TsaB protein, required for threonylcarbamoyladenosine (t(6)A) formation in tRNA
Cell Wall and Capsule	no subcategory	YjeE	TsaE protein, required for threonylcarbamoyladenosine t(6)A37 formation in tRNA
Clustering-based subsystems	Cell Division	Cell Division Subsystem including YidCD	Chromosomal replication initiator protein DnaA
Clustering-based subsystems	Cell Division	Cell Division Subsystem including YidCD	Chromosome (plasmid) partitioning protein ParA
Clustering-based subsystems	Cell Division	Cell Division Subsystem including YidCD	Chromosome (plasmid) partitioning protein ParB
Clustering-based subsystems	Cell Division	Cell Division Subsystem including YidCD	DNA gyrase subunit A (EC 5.99.1.3)
Clustering-based subsystems	Cell Division	Cell Division Subsystem including YidCD	DNA gyrase subunit B (EC 5.99.1.3)
Clustering-based subsystems	Cell Division	Cell Division Subsystem including YidCD	DNA polymerase III beta subunit (EC 2.7.7.7)
Clustering-based subsystems	Cell Division	Cell Division Subsystem including YidCD	DNA recombination and repair protein RecF
Clustering-based subsystems	Cell Division	Cell Division Subsystem including YidCD	GTPase and tRNA-U34 5-formylation enzyme TrmE
Clustering-based subsystems	Cell Division	Cell Division Subsystem including YidCD	Inner membrane protein translocase component YidC, long form
Clustering-based subsystems	Cell Division	Cell Division Subsystem including YidCD	LSU ribosomal protein L34p
Clustering-based subsystems	Cell Division	Cell Division Subsystem including YidCD	Ribonuclease P protein component (EC 3.1.26.5)
Clustering-based subsystems	Cell Division	Cell Division Subsystem including YidCD	rRNA small subunit 7-methylguanosine (m7G) methyltransferase GidB
Clustering-based subsystems	Cell Division	Cell Division Subsystem including YidCD	tRNA uridine 5-carboxymethylaminomethyl modification enzyme GidA
Clustering-based subsystems	Cell Division	cell division core of larger cluster	Cell division protein FtsA
Clustering-based subsystems	Cell Division	cell division core of larger cluster	Cell division protein FtsQ
Clustering-based subsystems	Cell Division	cell division core of larger cluster	Cell division protein FtsZ (EC 3.4.24.-)
Clustering-based subsystems	Cell Division	cell division core of larger cluster	UDP-N-acetylglucosamine--N-acetylmuramyl-(pentapeptide) pyrophosphoryl-undecaprenol N-acetylglucosamine transferase (EC 2.4.1.227)
Clustering-based subsystems	Central carbohydrate metabolism	Glyoxylate bypass cluster	Isocitrate lyase (EC 4.1.3.1)
Clustering-based subsystems	Clustering-based subsystems	Conenzyme B12 related Hypothetical: Clusters with cobST	Aerobic cobaltochelatase CobS subunit (EC 6.6.1.2)
Clustering-based subsystems	Clustering-based subsystems	Conenzyme B12 related Hypothetical: Clusters with cobST	Aerobic cobaltochelatase CobT subunit (EC 6.6.1.2)
Clustering-based subsystems	Clustering-based subsystems	Conenzyme B12 related Hypothetical: Clusters with cobST	FIG003437: hypothetical with DnaJ-like domain
Clustering-based subsystems	Cofactors	Riboflavin synthesis cluster	3,4-dihydroxy-2-butanone 4-phosphate synthase (EC 4.1.99.12)
Clustering-based subsystems	Cofactors	Riboflavin synthesis cluster	5-amino-6-(5-phosphoribosylamino)uracil reductase (EC 1.1.1.193)
Clustering-based subsystems	Cofactors	Riboflavin synthesis cluster	6,7-dimethyl-8-ribityllumazine synthase (EC 2.5.1.78)
Clustering-based subsystems	Cofactors	Riboflavin synthesis cluster	ATP phosphoribosyltransferase (EC 2.4.2.17)
Clustering-based subsystems	Cofactors	Riboflavin synthesis cluster	C-terminal domain of CinA type S
Clustering-based subsystems	Cofactors	Riboflavin synthesis cluster	Diaminohydroxyphosphoribosylaminopyrimidine deaminase (EC 3.5.4.26)
Clustering-based subsystems	Cofactors	Riboflavin synthesis cluster	GTP cyclohydrolase II (EC 3.5.4.25)
Clustering-based subsystems	Cofactors	Riboflavin synthesis cluster	Inner membrane protein YihY, formerly thought to be RNase BN
Clustering-based subsystems	Cofactors	Riboflavin synthesis cluster	NADH dehydrogenase (EC 1.6.99.3)
Clustering-based subsystems	Cofactors	Riboflavin synthesis cluster	Orotidine 5'-phosphate decarboxylase (EC 4.1.1.23)
Clustering-based subsystems	Cofactors	Riboflavin synthesis cluster	Phosphatidylglycerophosphatase A (EC 3.1.3.27)
Clustering-based subsystems	Cofactors	Riboflavin synthesis cluster	Phosphoribosyl-ATP pyrophosphatase (EC 3.6.1.31)
Clustering-based subsystems	Cofactors	Riboflavin synthesis cluster	Riboflavin synthase eubacterial/eukaryotic (EC 2.5.1.9)
Clustering-based subsystems	Cofactors	Riboflavin synthesis cluster	Ribulose-phosphate 3-epimerase (EC 5.1.3.1)
Clustering-based subsystems	Cofactors	Riboflavin synthesis cluster	Thiamine-monophosphate kinase (EC 2.7.4.16)
Clustering-based subsystems	Cofactors	Riboflavin synthesis cluster	Transcription termination protein NusB
Clustering-based subsystems	DNA metabolism	CBSS-272943.3.peg.263	FIG041266: ATP-dependent nuclease subunit B
Clustering-based subsystems	DNA metabolism	CBSS-272943.3.peg.263	FIG061771: ATP-dependent nuclease subunit A
Clustering-based subsystems	DNA metabolism	CBSS-272943.3.peg.263	FIG149041: Thioredoxin
Clustering-based subsystems	DNA metabolism	CBSS-272943.3.peg.263	Nucleotidyl transferase possibly involved in threonylcarbamoyladenosine formation
Clustering-based subsystems	DNA metabolism	CBSS-272943.3.peg.263	TsaE protein, required for threonylcarbamoyladenosine t(6)A37 formation in tRNA
Clustering-based subsystems	Hypothetical Related to Dihydroorate Dehydrogenase	Hypothetical Related to Dihydroorotate dehydrogenase	Dihydroorotate dehydrogenase (EC 1.3.3.1)
Clustering-based subsystems	Hypothetical Related to Dihydroorate Dehydrogenase	Hypothetical Related to Dihydroorotate dehydrogenase	FIG005495: hypothetical protein
Clustering-based subsystems	Isoprenoid/cell wall biosynthesis: PREDICTED UNDECAPRENYL DIPHOSPHATE PHOSPHATASE	CBSS-83331.1.peg.3039	1-deoxy-D-xylulose 5-phosphate reductoisomerase (EC 1.1.1.267)
Clustering-based subsystems	Isoprenoid/cell wall biosynthesis: PREDICTED UNDECAPRENYL DIPHOSPHATE PHOSPHATASE	CBSS-83331.1.peg.3039	1-hydroxy-2-methyl-2-(E)-butenyl 4-diphosphate synthase (EC 1.17.7.1)
Clustering-based subsystems	Isoprenoid/cell wall biosynthesis: PREDICTED UNDECAPRENYL DIPHOSPHATE PHOSPHATASE	CBSS-83331.1.peg.3039	Cell division protein FtsI [Peptidoglycan synthetase] (EC 2.4.1.129)
Clustering-based subsystems	Isoprenoid/cell wall biosynthesis: PREDICTED UNDECAPRENYL DIPHOSPHATE PHOSPHATASE	CBSS-83331.1.peg.3039	Penicillin-binding protein 2 (PBP-2)

Clustering-based subsystems	Isoprenoid/cell wall biosynthesis: PREDICTED UNDECAPRENYL DIPHOSPHATE PHOSPHATASE	CBSS-83331.1.peg.3039	Undecaprenyl diphosphate synthase (EC 2.5.1.31)
Clustering-based subsystems	Lysine, threonine, methionine, and cysteine	CBSS-84588.1.peg.1247	Cysteine desulfurase (EC 2.8.1.7)
Clustering-based subsystems	Lysine, threonine, methionine, and cysteine	CBSS-84588.1.peg.1247	D-alanyl-D-alanine carboxypeptidase (EC 3.4.16.4)
Clustering-based subsystems	Lysine, threonine, methionine, and cysteine	CBSS-84588.1.peg.1247	Diaminopimelate epimerase (EC 5.1.1.7)
Clustering-based subsystems	May be related to ADP-phosphoribose and NAD-dependent acetylation	CBSS-216591.1.peg.168	ADP-ribose pyrophosphatase (EC 3.6.1.13)
Clustering-based subsystems	May be related to ADP-phosphoribose and NAD-dependent acetylation	CBSS-216591.1.peg.168	Aspartate aminotransferase (EC 2.6.1.1)
Clustering-based subsystems	May be related to ADP-phosphoribose and NAD-dependent acetylation	CBSS-216591.1.peg.168	Aspartokinase (EC 2.7.2.4)
Clustering-based subsystems	May be related to ADP-phosphoribose and NAD-dependent acetylation	CBSS-216591.1.peg.168	Competence protein F homolog, phosphoribosyltransferase domain
Clustering-based subsystems	May be related to ADP-phosphoribose and NAD-dependent acetylation	CBSS-216591.1.peg.168	FIG004851: hypothetical protein
Clustering-based subsystems	May be related to ADP-phosphoribose and NAD-dependent acetylation	CBSS-216591.1.peg.168	FIG147869: Carbon-nitrogen hydrolase
Clustering-based subsystems	May be related to ADP-phosphoribose and NAD-dependent acetylation	CBSS-216591.1.peg.168	Glutaredoxin 3 (Grx2)
Clustering-based subsystems	May be related to ADP-phosphoribose and NAD-dependent acetylation	CBSS-216591.1.peg.168	Histone acetyltransferase HPA2 and related acetyltransferases
Clustering-based subsystems	May be related to ADP-phosphoribose and NAD-dependent acetylation	CBSS-216591.1.peg.168	MaoC-like dehydratase
Clustering-based subsystems	May be related to ADP-phosphoribose and NAD-dependent acetylation	CBSS-216591.1.peg.168	SAM-dependent methyltransferase, BioC-like
Clustering-based subsystems	May be related to amine metabolism	CBSS-314225.3.peg.1533	Putative uncharacterized protein ydbH
Clustering-based subsystems	May be related to amine metabolism	CBSS-314225.3.peg.1533	Putative uncharacterized protein ydbL, may be related to amine metabolism
Clustering-based subsystems	May be related to amine metabolism	CBSS-314225.3.peg.1533	Uncharacterized protein ynbE
Clustering-based subsystems	May be related to amine metabolism	CBSS-314225.3.peg.1533	probable lipoprotein STY1424
Clustering-based subsystems	Phosphate metabolism	PhoR-PhoB two-component regulatory system	Phosphate ABC transporter, periplasmic phosphate-binding protein PstS (TC 3.A.1.7.1)
Clustering-based subsystems	Phosphate metabolism	PhoR-PhoB two-component regulatory system	Phosphate regulon sensor protein PhoR (SphS) (EC 2.7.13.3)
Clustering-based subsystems	Phosphate metabolism	PhoR-PhoB two-component regulatory system	Phosphate regulon transcriptional regulatory protein PhoB (SphR)
Clustering-based subsystems	Probably GTP or GMP signaling related	CBSS-176299.4.peg.1292	DNA recombination and repair protein RecO
Clustering-based subsystems	Probably GTP or GMP signaling related	CBSS-176299.4.peg.1292	DNA-directed RNA polymerase omega subunit (EC 2.7.7.6)
Clustering-based subsystems	Probably GTP or GMP signaling related	CBSS-176299.4.peg.1292	GTP-binding protein Era
Clustering-based subsystems	Probably GTP or GMP signaling related	CBSS-176299.4.peg.1292	Guanosine-3',5'-bis(diphosphate) 3'-pyrophosphohydrolase (EC 3.1.7.2)
Clustering-based subsystems	Probably GTP or GMP signaling related	CBSS-176299.4.peg.1292	Holo-[acyl-carrier protein] synthase (EC 2.7.8.7)
Clustering-based subsystems	Probably GTP or GMP signaling related	CBSS-176299.4.peg.1292	Mll7752 protein
Clustering-based subsystems	Probably GTP or GMP signaling related	CBSS-176299.4.peg.1292	Ribonuclease III (EC 3.1.26.3)
Clustering-based subsystems	Probably organic hydroperoxide resistance related hypothetical protein	CBSS-269482.1.peg.1294	Homoserine kinase (EC 2.7.1.39)
Clustering-based subsystems	Putative Isoquinoline 1-oxidoreductase subunit	CBSS-314267.3.peg.390	Isoquinoline 1-oxidoreductase alpha subunit (EC 1.3.99.16)
Clustering-based subsystems	Putative Isoquinoline 1-oxidoreductase subunit	CBSS-314267.3.peg.390	Isoquinoline 1-oxidoreductase beta subunit (EC 1.3.99.16)
Clustering-based subsystems	RNA metabolism	Possible RNA degradation cluster	2-C-methyl-D-erythritol 2,4-cyclodiphosphate synthase (EC 4.6.1.12)
Clustering-based subsystems	RNA metabolism	Possible RNA degradation cluster	2-C-methyl-D-erythritol 4-phosphate cytidylyltransferase (EC 2.7.7.60)
Clustering-based subsystems	RNA metabolism	Possible RNA degradation cluster	6,7-dimethyl-8-ribityllumazine synthase (EC 2.5.1.78)
Clustering-based subsystems	RNA metabolism	Possible RNA degradation cluster	C-terminal domain of CinA type S
Clustering-based subsystems	RNA metabolism	Possible RNA degradation cluster	GTP-binding protein HflX
Clustering-based subsystems	RNA metabolism	Possible RNA degradation cluster	Lipoate synthase
Clustering-based subsystems	RNA metabolism	Possible RNA degradation cluster	Nitrogen regulation protein NtrB (EC 2.7.13.3)
Clustering-based subsystems	RNA metabolism	Possible RNA degradation cluster	Nitrogen regulation protein NtrC
Clustering-based subsystems	RNA metabolism	Possible RNA degradation cluster	Nitrogen regulation protein NtrX
Clustering-based subsystems	RNA metabolism	Possible RNA degradation cluster	Nitrogen regulation protein NtrY (EC 2.7.3.-)
Clustering-based subsystems	RNA metabolism	Possible RNA degradation cluster	RNA-binding protein Hfq
Clustering-based subsystems	RNA metabolism	Possible RNA degradation cluster	Trk system potassium uptake protein TrkA
Clustering-based subsystems	RNA metabolism	Possible RNA degradation cluster	tRNA dihydrouridine synthase A
Clustering-based subsystems	RNA metabolism	Possible RNA degradation cluster	tRNA dihydrouridine synthase B (EC 1.-.-.-)
Clustering-based subsystems	TldD cluster	CBSS-354.1.peg.2917	Cytoplasmic axial filament protein CafA and Ribonuclease G (EC 3.1.4.-)
Clustering-based subsystems	TldD cluster	CBSS-354.1.peg.2917	Rod shape-determining protein MreC
Clustering-based subsystems	TldD cluster	CBSS-354.1.peg.2917	Septum formation protein Maf
Clustering-based subsystems	TldD cluster	CBSS-354.1.peg.2917	TldD protein, part of TldE/TldD proteolytic complex
Clustering-based subsystems	Ubiquinol-cytochrome C chaperone locus	CBSS-176299.3.peg.2475	Ubiquinol-cytochrome C chaperone
Clustering-based subsystems	no subcategory	Aromatic conversions and predicted Co2 transporter cluster	3-dehydroquinate synthase (EC 4.2.3.4)
Clustering-based subsystems	no subcategory	Aromatic conversions and predicted Co2 transporter cluster	Co2 transporter containing CBS domains
Clustering-based subsystems	no subcategory	Aromatic conversions and predicted Co2 transporter cluster	Shikimate kinase I (EC 2.7.1.71)
Clustering-based subsystems	no subcategory	Aromatic conversions and predicted Co2 transporter cluster	Tyrosine recombinase XerD
Clustering-based subsystems	no subcategory	Bacterial RNA-metabolizing Zn-dependent hydrolases	Cell division protein FtsK
Clustering-based subsystems	no subcategory	Bacterial RNA-metabolizing Zn-dependent hydrolases	Metallo-beta-lactamase family protein, RNA-specific
Clustering-based subsystems	no subcategory	Bacterial RNA-metabolizing Zn-dependent hydrolases	Peptide deformylase (EC 3.5.1.88)
Clustering-based subsystems	no subcategory	Bacterial RNA-metabolizing Zn-dependent hydrolases	Polyribonucleotide nucleotidyltransferase (EC 2.7.7.8)
Clustering-based subsystems	no subcategory	Bacterial RNA-metabolizing Zn-dependent hydrolases	Ribosomal-protein-S18p-alanine acetyltransferase (EC 2.3.1.-)
Clustering-based subsystems	no subcategory	Bacterial RNA-metabolizing Zn-dependent hydrolases	Trk system potassium uptake protein TrkA

Clustering-based subsystems	no subcategory	Bacterial RNA-metabolizing Zn-dependent hydrolases	TsaB protein, required for threonylcarbamoyladenosine (t(6)A) formation in tRNA
Clustering-based subsystems	no subcategory	Bacterial RNA-metabolizing Zn-dependent hydrolases	TsaD/Kae1/Qri7 protein, required for threonylcarbamoyladenosine t(6)A37 formation in tRNA
Clustering-based subsystems	no subcategory	Biotin carboxylase and ubiquinone oxidoreductase subunit cluster	3-dehydroquinate dehydratase II (EC 4.2.1.10)
Clustering-based subsystems	no subcategory	Biotin carboxylase and ubiquinone oxidoreductase subunit cluster	Biotin carboxyl carrier protein of acetyl-CoA carboxylase
Clustering-based subsystems	no subcategory	Biotin carboxylase and ubiquinone oxidoreductase subunit cluster	Biotin carboxylase of acetyl-CoA carboxylase (EC 6.3.4.14)
Clustering-based subsystems	no subcategory	Biotin carboxylase and ubiquinone oxidoreductase subunit cluster	Leucyl/phenylalanyl-tRNA--protein transferase (EC 2.3.2.6)
Clustering-based subsystems	no subcategory	Biotin carboxylase and ubiquinone oxidoreductase subunit cluster	NADH:ubiquinone oxidoreductase 17.2 kD subunit
Clustering-based subsystems	no subcategory	CBSS-1352.1.peg.856	Transcriptional regulator, PadR family
Clustering-based subsystems	no subcategory	CBSS-138119.3.peg.2719	Ribosome-binding factor A
Clustering-based subsystems	no subcategory	CBSS-138119.3.peg.2719	Translation initiation factor 2
Clustering-based subsystems	no subcategory	CBSS-138119.3.peg.2719	tRNA pseudouridine synthase B (EC 4.2.1.70)
Clustering-based subsystems	no subcategory	CBSS-176279.3.peg.868	50S ribosomal protein acetyltransferase
Clustering-based subsystems	no subcategory	CBSS-176279.3.peg.868	GTP-binding protein Obg
Clustering-based subsystems	no subcategory	CBSS-176279.3.peg.868	LSU ribosomal protein L21p
Clustering-based subsystems	no subcategory	CBSS-176279.3.peg.868	LSU ribosomal protein L27p
Clustering-based subsystems	no subcategory	CBSS-176299.4.peg.1996A	ATP/GTP-binding site motif A
Clustering-based subsystems	no subcategory	CBSS-176299.4.peg.1996A	Bis(5'-nucleosyl)-tetraphosphatase (asymmetrical) (EC 3.6.1.17)
Clustering-based subsystems	no subcategory	CBSS-176299.4.peg.1996A	Endoribonuclease L-PSP
Clustering-based subsystems	no subcategory	CBSS-176299.4.peg.1996A	FIG110192: hypothetical protein
Clustering-based subsystems	no subcategory	CBSS-176299.4.peg.1996A	Glycerophosphoryl diester phosphodiesterase (EC 3.1.4.46)
Clustering-based subsystems	no subcategory	CBSS-216600.3.peg.802	Peptide chain release factor 1
Clustering-based subsystems	no subcategory	CBSS-216600.3.peg.802	Protein-N(5)-glutamine methyltransferase PrmC, methylates polypeptide chain release factors RF1 and RF2
Clustering-based subsystems	no subcategory	CBSS-224911.1.peg.435	Adenosine (5')-pentaphospho-(5")-adenosine pyrophosphohydrolase (EC 3.6.1.-)
Clustering-based subsystems	no subcategory	CBSS-224911.1.peg.435	Carboxyl-terminal protease (EC 3.4.21.102)
Clustering-based subsystems	no subcategory	CBSS-224911.1.peg.435	Periplasmic septal ring factor with murein hydrolase activity EnvC/YibP
Clustering-based subsystems	no subcategory	CBSS-224911.1.peg.435	Putative periplasmic protein YibQ, distant homology with nucleoside diphosphatase and polysaccharide deacetylase
Clustering-based subsystems	no subcategory	CBSS-272943.3.peg.1367	DNA topoisomerase I (EC 5.99.1.2)
Clustering-based subsystems	no subcategory	CBSS-272943.3.peg.1367	Rossmann fold nucleotide-binding protein Smf possibly involved in DNA uptake
Clustering-based subsystems	no subcategory	CBSS-290633.1.peg.1906	GTP-binding protein EngA
Clustering-based subsystems	no subcategory	CBSS-290633.1.peg.1906	Mlr7403 protein
Clustering-based subsystems	no subcategory	CBSS-290633.1.peg.1906	Outer membrane protein YfgL, lipoprotein component of the protein assembly complex (forms a complex with YaeT, YfiO, and NlpB)
Clustering-based subsystems	no subcategory	CBSS-312309.3.peg.1965	Methionine aminopeptidase (EC 3.4.11.18)
Clustering-based subsystems	no subcategory	CBSS-312309.3.peg.1965	SSU ribosomal protein S2p (SAe)
Clustering-based subsystems	no subcategory	CBSS-312309.3.peg.1965	Translation elongation factor Ts
Clustering-based subsystems	no subcategory	CBSS-312309.3.peg.1965	Uridine monophosphate kinase (EC 2.7.4.22)
Clustering-based subsystems	no subcategory	CBSS-312309.3.peg.1965	[Protein-PII] uridylyltransferase (EC 2.7.7.59)
Clustering-based subsystems	no subcategory	CBSS-316056.14.peg.4707	FIG026765: hypothetical protein
Clustering-based subsystems	no subcategory	CBSS-316056.14.peg.4707	NAD-dependent glyceraldehyde-3-phosphate dehydrogenase (EC 1.2.1.12)
Clustering-based subsystems	no subcategory	CBSS-316056.14.peg.4707	Phosphoglycerate kinase (EC 2.7.2.3)
Clustering-based subsystems	no subcategory	CBSS-316056.14.peg.4707	Transketolase (EC 2.2.1.1)
Clustering-based subsystems	no subcategory	CBSS-316057.3.peg.3521	Glutamate-ammonia-ligase adenylyltransferase (EC 2.7.7.42)
Clustering-based subsystems	no subcategory	CBSS-316057.3.peg.3521	Large exoproteins involved in heme utilization or adhesion
Clustering-based subsystems	no subcategory	CBSS-316057.3.peg.3521	Thiol peroxidase, Bcp-type (EC 1.11.1.15)
Clustering-based subsystems	no subcategory	CBSS-316057.3.peg.563	Cytochrome c oxidase polypeptide II (EC 1.9.3.1)
Clustering-based subsystems	no subcategory	CBSS-316057.3.peg.563	Cytochrome c oxidase polypeptide III (EC 1.9.3.1)
Clustering-based subsystems	no subcategory	CBSS-316057.3.peg.563	Cytochrome oxidase biogenesis protein Cox11-CtaG, copper delivery to Cox1
Clustering-based subsystems	no subcategory	CBSS-316057.3.peg.563	Heme O synthase, protoheme IX farnesyltransferase (EC 2.5.1.-) COX10-CtaB
Clustering-based subsystems	no subcategory	CBSS-316057.3.peg.563	TldD protein, part of TldE/TldD proteolytic complex
Clustering-based subsystems	no subcategory	CBSS-316057.3.peg.563	conserved hypothetical protein in cyt c oxidase gene clusters
Clustering-based subsystems	no subcategory	CBSS-323097.3.peg.2594	FIG004453: protein YceG like
Clustering-based subsystems	no subcategory	CBSS-323097.3.peg.2594	Guanylate kinase (EC 2.7.4.8)
Clustering-based subsystems	no subcategory	CBSS-323097.3.peg.2594	Protein YicC
Clustering-based subsystems	no subcategory	CBSS-331978.3.peg.2915	Preprotein translocase subunit SecG (TC 3.A.5.1.1)
Clustering-based subsystems	no subcategory	CBSS-331978.3.peg.2915	Triosephosphate isomerase (EC 5.3.1.1)
Clustering-based subsystems	no subcategory	CBSS-349161.4.peg.2427	Glycyl-tRNA synthetase alpha chain (EC 6.1.1.14)
Clustering-based subsystems	no subcategory	CBSS-349161.4.peg.2427	Glycyl-tRNA synthetase beta chain (EC 6.1.1.14)
Clustering-based subsystems	no subcategory	CBSS-349161.4.peg.2427	Pyruvate,phosphate dikinase (EC 2.7.9.1)
Clustering-based subsystems	no subcategory	CBSS-366602.3.peg.5141	16S rRNA m(5)C 967 methyltransferase (EC 2.1.1.-)
Clustering-based subsystems	no subcategory	CBSS-366602.3.peg.5141	Heparinase II/III-like
Clustering-based subsystems	no subcategory	CBSS-366602.3.peg.5141	IMP cyclohydrolase (EC 3.5.4.10)
Clustering-based subsystems	no subcategory	CBSS-366602.3.peg.5141	Phosphoribosylaminoimidazolecarboxamide formyltransferase (EC 2.1.2.3)
Clustering-based subsystems	no subcategory	CBSS-56780.10.peg.1536	Magnesium and cobalt efflux protein CorC
Clustering-based subsystems	no subcategory	CBSS-56780.10.peg.1536	Metal-dependent hydrolase YbeY, involved in rRNA and/or ribosome maturation and assembly
Clustering-based subsystems	no subcategory	CBSS-56780.10.peg.1536	Phosphate starvation-inducible protein PhoH, predicted ATPase
Clustering-based subsystems	no subcategory	CBSS-87626.3.peg.3639	2-octaprenyl-3-methyl-6-methoxy-1,4-benzoquinol hydroxylase (EC 1.14.13.-)

Clustering-based subsystems	no subcategory	CBSS-87626.3.peg.3639	Aminomethyltransferase (glycine cleavage system T protein) (EC 2.1.2.10)
Clustering-based subsystems	no subcategory	CBSS-87626.3.peg.3639	Xaa-Pro aminopeptidase (EC 3.4.11.9)
Clustering-based subsystems	no subcategory	CTP synthase (EC 6.3.4.2) cluster	CTP synthase (EC 6.3.4.2)
Clustering-based subsystems	no subcategory	CTP synthase (EC 6.3.4.2) cluster	Inner membrane protein translocase component YidC, long form
Clustering-based subsystems	no subcategory	ClpAS cluster	ATP-dependent Clp protease ATP-binding subunit ClpA
Clustering-based subsystems	no subcategory	ClpAS cluster	ATP-dependent Clp protease adaptor protein ClpS
Clustering-based subsystems	no subcategory	Conserved gene cluster associated with Met-tRNA formyltransferase	16S rRNA m(5)C 967 methyltransferase (EC 2.1.1.-)
Clustering-based subsystems	no subcategory	Conserved gene cluster associated with Met-tRNA formyltransferase	DNA topoisomerase I (EC 5.99.1.2)
Clustering-based subsystems	no subcategory	Conserved gene cluster associated with Met-tRNA formyltransferase	Helicase PriA essential for oriC/DnaA-independent DNA replication
Clustering-based subsystems	no subcategory	Conserved gene cluster associated with Met-tRNA formyltransferase	Methionyl-tRNA formyltransferase (EC 2.1.2.9)
Clustering-based subsystems	no subcategory	Conserved gene cluster associated with Met-tRNA formyltransferase	Peptide deformylase (EC 3.5.1.88)
Clustering-based subsystems	no subcategory	Conserved gene cluster associated with Met-tRNA formyltransferase	Ribonuclease HII (EC 3.1.26.4)
Clustering-based subsystems	no subcategory	Conserved gene cluster associated with Met-tRNA formyltransferase	Ribosomal RNA large subunit methyltransferase N (EC 2.1.1.-)
Clustering-based subsystems	no subcategory	Conserved gene cluster associated with Met-tRNA formyltransferase	Ribosomal-protein-S18p-alanine acetyltransferase (EC 2.3.1.-)
Clustering-based subsystems	no subcategory	Conserved gene cluster associated with Met-tRNA formyltransferase	Ribulose-phosphate 3-epimerase (EC 5.1.3.1)
Clustering-based subsystems	no subcategory	Conserved gene cluster associated with Met-tRNA formyltransferase	Rossmann fold nucleotide-binding protein Smf possibly involved in DNA uptake
Clustering-based subsystems	no subcategory	Conserved gene cluster associated with Met-tRNA formyltransferase	Trk system potassium uptake protein TrkA
Clustering-based subsystems	no subcategory	Conserved gene cluster associated with Met-tRNA formyltransferase	TsaB protein, required for threonylcarbamoyladenosine (t(6)A) formation in tRNA
Clustering-based subsystems	no subcategory	Conserved gene cluster associated with Met-tRNA formyltransferase	TsaD/Kae1/Qri7 protein, required for threonylcarbamoyladenosine t(6)A37 formation in tRNA
Clustering-based subsystems	no subcategory	DNA gyrase subunits	DNA gyrase subunit A (EC 5.99.1.3)
Clustering-based subsystems	no subcategory	DNA gyrase subunits	DNA gyrase subunit B (EC 5.99.1.3)
Clustering-based subsystems	no subcategory	DNA replication cluster 1	Chromosomal replication initiator protein DnaA
Clustering-based subsystems	no subcategory	DNA replication cluster 1	DNA gyrase subunit A (EC 5.99.1.3)
Clustering-based subsystems	no subcategory	DNA replication cluster 1	DNA gyrase subunit B (EC 5.99.1.3)
Clustering-based subsystems	no subcategory	DNA replication cluster 1	DNA polymerase III beta subunit (EC 2.7.7.7)
Clustering-based subsystems	no subcategory	DNA replication cluster 1	DNA recombination and repair protein RecF
Clustering-based subsystems	no subcategory	DNA replication cluster 1	Ribosome-associated heat shock protein implicated in the recycling of the 50S subunit (S4 paralog)
Clustering-based subsystems	no subcategory	DNA replication cluster 1	Zn-ribbon-containing, possibly RNA-binding protein and truncated derivatives
Clustering-based subsystems	no subcategory	ECSIG4-SIG7	4-hydroxythreonine-4-phosphate dehydrogenase (EC 1.1.1.262)
Clustering-based subsystems	no subcategory	ECSIG4-SIG7	Organic solvent tolerance protein precursor
Clustering-based subsystems	no subcategory	ECSIG4-SIG7	SSU rRNA (adenine(1518)-N(6)/adenine(1519)-N(6))-dimethyltransferase (EC 2.1.1.182)
Clustering-based subsystems	no subcategory	ECSIG4-SIG7	Survival protein SurA precursor (Peptidyl-prolyl cis-trans isomerase SurA) (EC 5.2.1.8)
Clustering-based subsystems	no subcategory	Glutaredoxin 3 containing cluster 2	Competence protein F homolog, phosphoribosyltransferase domain
Clustering-based subsystems	no subcategory	Glutaredoxin 3 containing cluster 2	FIG004851: hypothetical protein
Clustering-based subsystems	no subcategory	Glutaredoxin 3 containing cluster 2	FIG147869: Carbon-nitrogen hydrolase
Clustering-based subsystems	no subcategory	Glutaredoxin 3 containing cluster 2	Glutaredoxin 3 (Grx2)
Clustering-based subsystems	no subcategory	Glutaredoxin 3 containing cluster 2	SAM-dependent methyltransferase, BioC-like
Clustering-based subsystems	no subcategory	LSU ribosomal proteins cluster	LSU ribosomal protein L10p (P0)
Clustering-based subsystems	no subcategory	LSU ribosomal proteins cluster	LSU ribosomal protein L11p (L12e)
Clustering-based subsystems	no subcategory	LSU ribosomal proteins cluster	LSU ribosomal protein L1p (L10Ae)
Clustering-based subsystems	no subcategory	LSU ribosomal proteins cluster	LSU ribosomal protein L7/L12 (P1/P2)
Clustering-based subsystems	no subcategory	LSU ribosomal proteins cluster	Preprotein translocase subunit SecE (TC 3.A.5.1.1)
Clustering-based subsystems	no subcategory	LSU ribosomal proteins cluster	Transcription antitermination protein NusG
Clustering-based subsystems	no subcategory	Lipoic acid synthesis cluster	Lipoate synthase
Clustering-based subsystems	no subcategory	Lipoic acid synthesis cluster	Octanoate-[acyl-carrier-protein]-protein-N-octanoyltransferase
Clustering-based subsystems	no subcategory	NusA-TFII Cluster	COG2740: Predicted nucleic-acid-binding protein implicated in transcription termination
Clustering-based subsystems	no subcategory	NusA-TFII Cluster	FIG000325: clustered with transcription termination protein NusA
Clustering-based subsystems	no subcategory	NusA-TFII Cluster	Ribosome-binding factor A
Clustering-based subsystems	no subcategory	NusA-TFII Cluster	Transcription termination protein NusA
Clustering-based subsystems	no subcategory	NusA-TFII Cluster	Translation initiation factor 2
Clustering-based subsystems	no subcategory	NusA-TFII Cluster	ribosomal protein L7Ae family protein
Clustering-based subsystems	no subcategory	Purine salvage cluster	Exodeoxyribonuclease VII large subunit (EC 3.1.11.6)
Clustering-based subsystems	no subcategory	Purine salvage cluster	Exodeoxyribonuclease VII small subunit (EC 3.1.11.6)
Clustering-based subsystems	no subcategory	Purine salvage cluster	Inosine-5'-monophosphate dehydrogenase (EC 1.1.1.205)
Clustering-based subsystems	no subcategory	RNA modification and chromosome partitioning cluster	Chromosome (plasmid) partitioning protein ParA
Clustering-based subsystems	no subcategory	RNA modification and chromosome partitioning cluster	Chromosome (plasmid) partitioning protein ParB
Clustering-based subsystems	no subcategory	RNA modification and chromosome partitioning cluster	GTPase and tRNA-U34 5-formylation enzyme TrmE
Clustering-based subsystems	no subcategory	RNA modification and chromosome partitioning cluster	rRNA small subunit 7-methylguanosine (m7G) methyltransferase GidB
Clustering-based subsystems	no subcategory	RNA modification and chromosome partitioning cluster	tRNA uridine 5-carboxymethylaminomethyl modification enzyme GidA
Clustering-based subsystems	no subcategory	RNA modification cluster	GTPase and tRNA-U34 5-formylation enzyme TrmE
Clustering-based subsystems	no subcategory	RNA modification cluster	Inner membrane protein translocase component YidC, long form
Clustering-based subsystems	no subcategory	RNA modification cluster	LSU ribosomal protein L34p
Clustering-based subsystems	no subcategory	RNA modification cluster	Ribonuclease P protein component (EC 3.1.26.5)

Clustering-based subsystems	no subcategory	Ribosome recycling related cluster	ATP-dependent Clp protease ATP-binding subunit ClpA
Clustering-based subsystems	no subcategory	Ribosome recycling related cluster	Ribosome recycling factor
Clustering-based subsystems	no subcategory	Ribosome recycling related cluster	SSU ribosomal protein S2p (SAe)
Clustering-based subsystems	no subcategory	Ribosome recycling related cluster	Translation elongation factor Ts
Clustering-based subsystems	no subcategory	Ribosome recycling related cluster	Uridine monophosphate kinase (EC 2.7.4.22)
Clustering-based subsystems	no subcategory	Stationary phase repair cluster	2-C-methyl-D-erythritol 2,4-cyclodiphosphate synthase (EC 4.6.1.12)
Clustering-based subsystems	no subcategory	Stationary phase repair cluster	2-C-methyl-D-erythritol 4-phosphate cytidylyltransferase (EC 2.7.7.60)
Clustering-based subsystems	no subcategory	Stationary phase repair cluster	5-nucleotidase SurE (EC 3.1.3.5)
Clustering-based subsystems	no subcategory	Stationary phase repair cluster	Cell division protein DivIC (FtsB), stabilizes FtsL against RasP cleavage
Clustering-based subsystems	no subcategory	Stationary phase repair cluster	Cell division protein FtsL
Clustering-based subsystems	no subcategory	Stationary phase repair cluster	Protein-L-isoaspartate O-methyltransferase (EC 2.1.1.77)
Clustering-based subsystems	no subcategory	Ubiquinol-cytochrome C chaperone locus	Conserved hypothetical protein, gene in Ubiquinol-cytochrome C chaperone locus
Clustering-based subsystems	no subcategory	Ubiquinol-cytochrome C chaperone locus	Outer membrane lipoprotein OmlA
Clustering-based subsystems	no subcategory	Ubiquinol-cytochrome C chaperone locus	Ubiquinol-cytochrome C chaperone
Clustering-based subsystems	no subcategory	tRNA-methylthiotransferase containing cluster	Apolipoprotein N-acyltransferase (EC 2.3.1.-)
Clustering-based subsystems	no subcategory	tRNA-methylthiotransferase containing cluster	Copper homeostasis protein CutE
Clustering-based subsystems	no subcategory	tRNA-methylthiotransferase containing cluster	Magnesium and cobalt efflux protein CorC
Clustering-based subsystems	no subcategory	tRNA-methylthiotransferase containing cluster	Metal-dependent hydrolase YbeY, involved in rRNA and/or ribosome maturation and assembly
Clustering-based subsystems	no subcategory	tRNA-methylthiotransferase containing cluster	Phosphate starvation-inducible protein PhoH, predicted ATPase
Clustering-based subsystems	no subcategory	tRNA-methylthiotransferase containing cluster	tRNA-i(6)A37 methylthiotransferase
Clustering-based subsystems	pH adaptation potassium efflux	CBSS-272569.1.peg.3198	PH adaptation potassium efflux system protein B1
Clustering-based subsystems	pH adaptation potassium efflux	CBSS-272569.1.peg.3198	PH adaptation potassium efflux system protein C
Clustering-based subsystems	proteosome related	Cluster-based Subsystem Grouping Hypotheticals - perhaps Proteosome Related	Twin-arginine translocation protein TatA
Clustering-based subsystems	proteosome related	Cluster-based Subsystem Grouping Hypotheticals - perhaps Proteosome Related	Twin-arginine translocation protein TatC
Cofactors, Vitamins, Prosthetic Groups, Pigments	Biotin	Biotin biosynthesis	3-ketoacyl-CoA thiolase (EC 2.3.1.16)
Cofactors, Vitamins, Prosthetic Groups, Pigments	Biotin	Biotin biosynthesis	8-amino-7-oxononanoate synthase (EC 2.3.1.47)
Cofactors, Vitamins, Prosthetic Groups, Pigments	Biotin	Biotin biosynthesis	Adenosylmethionine-8-amino-7-oxononanoate aminotransferase (EC 2.6.1.62)
Cofactors, Vitamins, Prosthetic Groups, Pigments	Biotin	Biotin biosynthesis	Biotin synthase (EC 2.8.1.6)
Cofactors, Vitamins, Prosthetic Groups, Pigments	Biotin	Biotin biosynthesis	Biotin-protein ligase (EC 6.3.4.15)
Cofactors, Vitamins, Prosthetic Groups, Pigments	Biotin	Biotin biosynthesis	Dethiobiotin synthetase (EC 6.3.3.3)
Cofactors, Vitamins, Prosthetic Groups, Pigments	Biotin	Biotin biosynthesis	Long-chain-fatty-acid--CoA ligase (EC 6.2.1.3)
Cofactors, Vitamins, Prosthetic Groups, Pigments	Biotin	Biotin biosynthesis Experimental	8-amino-7-oxononanoate synthase (EC 2.3.1.47)
Cofactors, Vitamins, Prosthetic Groups, Pigments	Biotin	Biotin biosynthesis Experimental	Adenosylmethionine-8-amino-7-oxononanoate aminotransferase (EC 2.6.1.62)
Cofactors, Vitamins, Prosthetic Groups, Pigments	Biotin	Biotin biosynthesis Experimental	Biotin synthase (EC 2.8.1.6)
Cofactors, Vitamins, Prosthetic Groups, Pigments	Biotin	Biotin biosynthesis Experimental	Competence protein F homolog, phosphoribosyltransferase domain
Cofactors, Vitamins, Prosthetic Groups, Pigments	Biotin	Biotin biosynthesis Experimental	Dethiobiotin synthetase (EC 6.3.3.3)
Cofactors, Vitamins, Prosthetic Groups, Pigments	Biotin	Biotin biosynthesis Experimental	FIG147869: Carbon-nitrogen hydrolase
Cofactors, Vitamins, Prosthetic Groups, Pigments	Biotin	Biotin biosynthesis Experimental	Glutaredoxin 3 (Grx2)
Cofactors, Vitamins, Prosthetic Groups, Pigments	Biotin	Biotin biosynthesis Experimental	SAM-dependent methyltransferase, BioC-like
Cofactors, Vitamins, Prosthetic Groups, Pigments	Biotin	Biotin synthesis cluster	3-ketoacyl-CoA thiolase (EC 2.3.1.16)
Cofactors, Vitamins, Prosthetic Groups, Pigments	Biotin	Biotin synthesis cluster	8-amino-7-oxononanoate synthase (EC 2.3.1.47)
Cofactors, Vitamins, Prosthetic Groups, Pigments	Biotin	Biotin synthesis cluster	Adenosylmethionine-8-amino-7-oxononanoate aminotransferase (EC 2.6.1.62)
Cofactors, Vitamins, Prosthetic Groups, Pigments	Biotin	Biotin synthesis cluster	Biotin synthase (EC 2.8.1.6)
Cofactors, Vitamins, Prosthetic Groups, Pigments	Biotin	Biotin synthesis cluster	Biotin-protein ligase (EC 6.3.4.15)
Cofactors, Vitamins, Prosthetic Groups, Pigments	Biotin	Biotin synthesis cluster	Dethiobiotin synthetase (EC 6.3.3.3)
Cofactors, Vitamins, Prosthetic Groups, Pigments	Biotin	Biotin synthesis cluster	Long-chain-fatty-acid--CoA ligase (EC 6.2.1.3)
Cofactors, Vitamins, Prosthetic Groups, Pigments	Biotin	Biotin synthesis cluster	SAM-dependent methyltransferase, BioC-like
Cofactors, Vitamins, Prosthetic Groups, Pigments	Coenzyme A	Coenzyme A Biosynthesis	3-methyl-2-oxobutanoate hydroxymethyltransferase (EC 2.1.2.11)
Cofactors, Vitamins, Prosthetic Groups, Pigments	Coenzyme A	Coenzyme A Biosynthesis	Dephospho-CoA kinase (EC 2.7.1.24)
Cofactors, Vitamins, Prosthetic Groups, Pigments	Coenzyme A	Coenzyme A Biosynthesis	Ketol-acid reductoisomerase (EC 1.1.1.86)
Cofactors, Vitamins, Prosthetic Groups, Pigments	Coenzyme A	Coenzyme A Biosynthesis	Pantoate--beta-alanine ligase (EC 6.3.2.1)
Cofactors, Vitamins, Prosthetic Groups, Pigments	Coenzyme A	Coenzyme A Biosynthesis	Phosphopantetheine adenylyltransferase (EC 2.7.7.3)

Cofactors, Vitamins, Prosthetic Groups, Pigments	Coenzyme A	Coenzyme A Biosynthesis cluster	3-methyl-2-oxobutanoate hydroxymethyltransferase (EC 2.1.2.11)
Cofactors, Vitamins, Prosthetic Groups, Pigments	Coenzyme A	Coenzyme A Biosynthesis cluster	Pantoate--beta-alanine ligase (EC 6.3.2.1)
Cofactors, Vitamins, Prosthetic Groups, Pigments	Folate and pterines	5-FCL-like protein	5,10-methylenetetrahydrofolate reductase (EC 1.5.1.20)
Cofactors, Vitamins, Prosthetic Groups, Pigments	Folate and pterines	5-FCL-like protein	5-formyltetrahydrofolate cyclo-ligase (EC 6.3.3.2)
Cofactors, Vitamins, Prosthetic Groups, Pigments	Folate and pterines	5-FCL-like protein	Alcohol dehydrogenase (EC 1.1.1.1)
Cofactors, Vitamins, Prosthetic Groups, Pigments	Folate and pterines	5-FCL-like protein	Butyryl-CoA dehydrogenase (EC 1.3.8.1)
Cofactors, Vitamins, Prosthetic Groups, Pigments	Folate and pterines	5-FCL-like protein	Dihydrofolate reductase (EC 1.5.1.3)
Cofactors, Vitamins, Prosthetic Groups, Pigments	Folate and pterines	5-FCL-like protein	Dihydrolipoamide acetyltransferase component of pyruvate dehydrogenase complex (EC 2.3.1.12)
Cofactors, Vitamins, Prosthetic Groups, Pigments	Folate and pterines	5-FCL-like protein	Isocitrate dehydrogenase [NADP] (EC 1.1.1.42)
Cofactors, Vitamins, Prosthetic Groups, Pigments	Folate and pterines	5-FCL-like protein	Phosphoribosylaminoimidazolecarboxamide formyltransferase (EC 2.1.2.3)
Cofactors, Vitamins, Prosthetic Groups, Pigments	Folate and pterines	5-FCL-like protein	Phosphoribosylglycinamide formyltransferase (EC 2.1.2.2)
Cofactors, Vitamins, Prosthetic Groups, Pigments	Folate and pterines	5-FCL-like protein	Serine hydroxymethyltransferase (EC 2.1.2.1)
Cofactors, Vitamins, Prosthetic Groups, Pigments	Folate and pterines	5-FCL-like protein	Succinate dehydrogenase iron-sulfur protein (EC 1.3.99.1)
Cofactors, Vitamins, Prosthetic Groups, Pigments	Folate and pterines	5-FCL-like protein	Thiamin-phosphate pyrophosphorylase (EC 2.5.1.3)
Cofactors, Vitamins, Prosthetic Groups, Pigments	Folate and pterines	5-FCL-like protein	Thiamine-monophosphate kinase (EC 2.7.4.16)
Cofactors, Vitamins, Prosthetic Groups, Pigments	Folate and pterines	Folate Biosynthesis	2-amino-4-hydroxy-6-hydroxymethyldihydropteridine pyrophosphokinase (EC 2.7.6.3)
Cofactors, Vitamins, Prosthetic Groups, Pigments	Folate and pterines	Folate Biosynthesis	5-formyltetrahydrofolate cyclo-ligase (EC 6.3.3.2)
Cofactors, Vitamins, Prosthetic Groups, Pigments	Folate and pterines	Folate Biosynthesis	Aminodeoxychorismate lyase (EC 4.1.3.38)
Cofactors, Vitamins, Prosthetic Groups, Pigments	Folate and pterines	Folate Biosynthesis	Dihydrofolate reductase (EC 1.5.1.3)
Cofactors, Vitamins, Prosthetic Groups, Pigments	Folate and pterines	Folate Biosynthesis	Dihydrofolate synthase (EC 6.3.2.12)
Cofactors, Vitamins, Prosthetic Groups, Pigments	Folate and pterines	Folate Biosynthesis	Dihydropteroate synthase (EC 2.5.1.15)
Cofactors, Vitamins, Prosthetic Groups, Pigments	Folate and pterines	Folate Biosynthesis	Folylpolyglutamate synthase (EC 6.3.2.17)
Cofactors, Vitamins, Prosthetic Groups, Pigments	Folate and pterines	Folate Biosynthesis	GTP cyclohydrolase I (EC 3.5.4.16) type 1
Cofactors, Vitamins, Prosthetic Groups, Pigments	Folate and pterines	Folate Biosynthesis	Para-aminobenzoate synthase, amidotransferase component (EC 2.6.1.85)
Cofactors, Vitamins, Prosthetic Groups, Pigments	Folate and pterines	Folate Biosynthesis	Para-aminobenzoate synthase, aminase component (EC 2.6.1.85)
Cofactors, Vitamins, Prosthetic Groups, Pigments	Folate and pterines	Folate Biosynthesis	Thymidylate synthase (EC 2.1.1.45)
Cofactors, Vitamins, Prosthetic Groups, Pigments	Folate and pterines	Folate biosynthesis cluster	2-amino-4-hydroxy-6-hydroxymethyldihydropteridine pyrophosphokinase (EC 2.7.6.3)
Cofactors, Vitamins, Prosthetic Groups, Pigments	Folate and pterines	Folate biosynthesis cluster	Cell division protein FtsH (EC 3.4.24.-)
Cofactors, Vitamins, Prosthetic Groups, Pigments	Folate and pterines	Folate biosynthesis cluster	Dihydropteroate synthase (EC 2.5.1.15)
Cofactors, Vitamins, Prosthetic Groups, Pigments	Folate and pterines	Folate biosynthesis cluster	GTP cyclohydrolase I (EC 3.5.4.16) type 1
Cofactors, Vitamins, Prosthetic Groups, Pigments	Folate and pterines	Folate biosynthesis cluster	Pantoate--beta-alanine ligase (EC 6.3.2.1)
Cofactors, Vitamins, Prosthetic Groups, Pigments	Folate and pterines	Folate biosynthesis cluster	tRNA(Ile)-lysidine synthetase (EC 6.3.4.19)
Cofactors, Vitamins, Prosthetic Groups, Pigments	Folate and pterines	Pterin carbinolamine dehydratase	Fumarylacetoacetase (EC 3.7.1.2)
Cofactors, Vitamins, Prosthetic Groups, Pigments	Folate and pterines	Pterin carbinolamine dehydratase	Fumarylacetoacetate hydrolase family protein
Cofactors, Vitamins, Prosthetic Groups, Pigments	Folate and pterines	Pterin carbinolamine dehydratase	Maleylacetoacetate isomerase (EC 5.2.1.2)
Cofactors, Vitamins, Prosthetic Groups, Pigments	Folate and pterines	Pterin carbinolamine dehydratase	Pterin-4-alpha-carbinolamine dehydratase (EC 4.2.1.96)
Cofactors, Vitamins, Prosthetic Groups, Pigments	Lipoic acid	Lipoic acid metabolism	Lipoate synthase
Cofactors, Vitamins, Prosthetic Groups, Pigments	Lipoic acid	Lipoic acid metabolism	Octanoate-[acyl-carrier-protein]-protein-N-octanoyltransferase
Cofactors, Vitamins, Prosthetic Groups, Pigments	NAD and NADP	NAD and NADP cofactor biosynthesis global	ADP-ribose pyrophosphatase (EC 3.6.1.13)
Cofactors, Vitamins, Prosthetic Groups, Pigments	NAD and NADP	NAD and NADP cofactor biosynthesis global	Glutamine amidotransferase chain of NAD synthetase
Cofactors, Vitamins, Prosthetic Groups, Pigments	NAD and NADP	NAD and NADP cofactor biosynthesis global	L-aspartate oxidase (EC 1.4.3.16)
Cofactors, Vitamins, Prosthetic Groups, Pigments	NAD and NADP	NAD and NADP cofactor biosynthesis global	NAD kinase (EC 2.7.1.23)
Cofactors, Vitamins, Prosthetic Groups, Pigments	NAD and NADP	NAD and NADP cofactor biosynthesis global	NAD synthetase (EC 6.3.1.5)
Cofactors, Vitamins, Prosthetic Groups, Pigments	NAD and NADP	NAD and NADP cofactor biosynthesis global	Nicotinate-nucleotide adenylyltransferase (EC 2.7.7.18)
Cofactors, Vitamins, Prosthetic Groups, Pigments	NAD and NADP	NAD and NADP cofactor biosynthesis global	Quinolinate phosphoribosyltransferase [decarboxylating] (EC 2.4.2.19)
Cofactors, Vitamins, Prosthetic Groups, Pigments	NAD and NADP	NAD and NADP cofactor biosynthesis global	Quinolinate synthetase (EC 2.5.1.72)
Cofactors, Vitamins, Prosthetic Groups, Pigments	Pyridoxine	Pyridoxin(Vitamin B6) Degradation Pathway	Pyridoxal-5'-phosphate phosphatase (EC 3.1.3.74), Alphaproteobacterial type

Cofactors, Vitamins, Prosthetic Groups, Pigments	Pyridoxine	Pyridoxin (Vitamin B6) Biosynthesis	1-deoxy-D-xylulose 5-phosphate synthase (EC 2.2.1.7)
Cofactors, Vitamins, Prosthetic Groups, Pigments	Pyridoxine	Pyridoxin (Vitamin B6) Biosynthesis	4-hydroxythreonine-4-phosphate dehydrogenase (EC 1.1.1.262)
Cofactors, Vitamins, Prosthetic Groups, Pigments	Pyridoxine	Pyridoxin (Vitamin B6) Biosynthesis	D-3-phosphoglycerate dehydrogenase (EC 1.1.1.95)
Cofactors, Vitamins, Prosthetic Groups, Pigments	Pyridoxine	Pyridoxin (Vitamin B6) Biosynthesis	NAD-dependent glyceraldehyde-3-phosphate dehydrogenase (EC 1.2.1.12)
Cofactors, Vitamins, Prosthetic Groups, Pigments	Pyridoxine	Pyridoxin (Vitamin B6) Biosynthesis	Phosphoserine aminotransferase (EC 2.6.1.52)
Cofactors, Vitamins, Prosthetic Groups, Pigments	Pyridoxine	Pyridoxin (Vitamin B6) Biosynthesis	Pyridoxamine 5'-phosphate oxidase (EC 1.4.3.5)
Cofactors, Vitamins, Prosthetic Groups, Pigments	Pyridoxine	Pyridoxin (Vitamin B6) Biosynthesis	Pyridoxine 5'-phosphate synthase (EC 2.6.99.2)
Cofactors, Vitamins, Prosthetic Groups, Pigments	Quinone cofactors	Ubiquinone Biosynthesis	2-octaprenyl-3-methyl-6-methoxy-1,4-benzoquinol hydroxylase (EC 1.14.13.-)
Cofactors, Vitamins, Prosthetic Groups, Pigments	Quinone cofactors	Ubiquinone Biosynthesis	4-hydroxybenzoate polyprenyltransferase (EC 2.5.1.39)
Cofactors, Vitamins, Prosthetic Groups, Pigments	Quinone cofactors	Ubiquinone Biosynthesis	Ubiquinone biosynthesis monooxygenase UbiB
Cofactors, Vitamins, Prosthetic Groups, Pigments	Quinone cofactors	Ubiquinone Biosynthesis	Ubiquinone/menaquinone biosynthesis methyltransferase UbiE (EC 2.1.1.-)
Cofactors, Vitamins, Prosthetic Groups, Pigments	Quinone cofactors	Ubiquinone Biosynthesis - gjo	2-octaprenyl-3-methyl-6-methoxy-1,4-benzoquinol hydroxylase (EC 1.14.13.-)
Cofactors, Vitamins, Prosthetic Groups, Pigments	Quinone cofactors	Ubiquinone Biosynthesis - gjo	4-hydroxybenzoate polyprenyltransferase (EC 2.5.1.39)
Cofactors, Vitamins, Prosthetic Groups, Pigments	Quinone cofactors	Ubiquinone Biosynthesis - gjo	Ubiquinone biosynthesis monooxygenase UbiB
Cofactors, Vitamins, Prosthetic Groups, Pigments	Quinone cofactors	Ubiquinone Biosynthesis - gjo	Ubiquinone/menaquinone biosynthesis methyltransferase UbiE (EC 2.1.1.-)
Cofactors, Vitamins, Prosthetic Groups, Pigments	Riboflavin, FMN, FAD	Riboflavin, FMN and FAD metabolism	3,4-dihydroxy-2-butanone 4-phosphate synthase (EC 4.1.99.12)
Cofactors, Vitamins, Prosthetic Groups, Pigments	Riboflavin, FMN, FAD	Riboflavin, FMN and FAD metabolism	5-amino-6-(5-phosphoribosylamino)uracil reductase (EC 1.1.1.193)
Cofactors, Vitamins, Prosthetic Groups, Pigments	Riboflavin, FMN, FAD	Riboflavin, FMN and FAD metabolism	6,7-dimethyl-8-ribityllumazine synthase (EC 2.5.1.78)
Cofactors, Vitamins, Prosthetic Groups, Pigments	Riboflavin, FMN, FAD	Riboflavin, FMN and FAD metabolism	Diaminohydroxyphosphoribosylaminopyrimidine deaminase (EC 3.5.4.26)
Cofactors, Vitamins, Prosthetic Groups, Pigments	Riboflavin, FMN, FAD	Riboflavin, FMN and FAD metabolism	FMN adenylyltransferase (EC 2.7.7.2)
Cofactors, Vitamins, Prosthetic Groups, Pigments	Riboflavin, FMN, FAD	Riboflavin, FMN and FAD metabolism	GTP cyclohydrolase II (EC 3.5.4.25)
Cofactors, Vitamins, Prosthetic Groups, Pigments	Riboflavin, FMN, FAD	Riboflavin, FMN and FAD metabolism	Riboflavin kinase (EC 2.7.1.26)
Cofactors, Vitamins, Prosthetic Groups, Pigments	Riboflavin, FMN, FAD	Riboflavin, FMN and FAD metabolism	Riboflavin synthase eubacterial/eukaryotic (EC 2.5.1.9)
Cofactors, Vitamins, Prosthetic Groups, Pigments	Riboflavin, FMN, FAD	Riboflavin, FMN and FAD metabolism in plants	3,4-dihydroxy-2-butanone 4-phosphate synthase (EC 4.1.99.12)
Cofactors, Vitamins, Prosthetic Groups, Pigments	Riboflavin, FMN, FAD	Riboflavin, FMN and FAD metabolism in plants	5-amino-6-(5-phosphoribosylamino)uracil reductase (EC 1.1.1.193)
Cofactors, Vitamins, Prosthetic Groups, Pigments	Riboflavin, FMN, FAD	Riboflavin, FMN and FAD metabolism in plants	6,7-dimethyl-8-ribityllumazine synthase (EC 2.5.1.78)
Cofactors, Vitamins, Prosthetic Groups, Pigments	Riboflavin, FMN, FAD	Riboflavin, FMN and FAD metabolism in plants	Diaminohydroxyphosphoribosylaminopyrimidine deaminase (EC 3.5.4.26)
Cofactors, Vitamins, Prosthetic Groups, Pigments	Riboflavin, FMN, FAD	Riboflavin, FMN and FAD metabolism in plants	FIG000859: hypothetical protein YebC
Cofactors, Vitamins, Prosthetic Groups, Pigments	Riboflavin, FMN, FAD	Riboflavin, FMN and FAD metabolism in plants	FMN adenylyltransferase (EC 2.7.7.2)
Cofactors, Vitamins, Prosthetic Groups, Pigments	Riboflavin, FMN, FAD	Riboflavin, FMN and FAD metabolism in plants	GTP cyclohydrolase II (EC 3.5.4.25)
Cofactors, Vitamins, Prosthetic Groups, Pigments	Riboflavin, FMN, FAD	Riboflavin, FMN and FAD metabolism in plants	Molybdopterin binding motif, CinA N-terminal domain
Cofactors, Vitamins, Prosthetic Groups, Pigments	Riboflavin, FMN, FAD	Riboflavin, FMN and FAD metabolism in plants	Riboflavin kinase (EC 2.7.1.26)
Cofactors, Vitamins, Prosthetic Groups, Pigments	Riboflavin, FMN, FAD	Riboflavin, FMN and FAD metabolism in plants	Riboflavin synthase eubacterial/eukaryotic (EC 2.5.1.9)
Cofactors, Vitamins, Prosthetic Groups, Pigments	Riboflavin, FMN, FAD	Riboflavin, FMN and FAD metabolism in plants	tRNA pseudouridine synthase B (EC 4.2.1.70)
Cofactors, Vitamins, Prosthetic Groups, Pigments	Tetrapyrroles	Heme and Siroheme Biosynthesis	5-aminolevulinate synthase (EC 2.3.1.37)
Cofactors, Vitamins, Prosthetic Groups, Pigments	Tetrapyrroles	Heme and Siroheme Biosynthesis	Coproporphyrinogen III oxidase, aerobic (EC 1.3.3.3)
Cofactors, Vitamins, Prosthetic Groups, Pigments	Tetrapyrroles	Heme and Siroheme Biosynthesis	Ferrochelatase, protoheme ferro-lyase (EC 4.99.1.1)
Cofactors, Vitamins, Prosthetic Groups, Pigments	Tetrapyrroles	Heme and Siroheme Biosynthesis	Glutamyl-tRNA synthetase (EC 6.1.1.17)
Cofactors, Vitamins, Prosthetic Groups, Pigments	Tetrapyrroles	Heme and Siroheme Biosynthesis	Porphobilinogen deaminase (EC 2.5.1.61)
Cofactors, Vitamins, Prosthetic Groups, Pigments	Tetrapyrroles	Heme and Siroheme Biosynthesis	Porphobilinogen synthase (EC 4.2.1.24)
Cofactors, Vitamins, Prosthetic Groups, Pigments	Tetrapyrroles	Heme and Siroheme Biosynthesis	Precorrin-2 oxidase (EC 1.3.1.76)
Cofactors, Vitamins, Prosthetic Groups, Pigments	Tetrapyrroles	Heme and Siroheme Biosynthesis	Protoporphyrinogen IX oxidase, novel form, HemJ (EC 1.3.-.-)
Cofactors, Vitamins, Prosthetic Groups, Pigments	Tetrapyrroles	Heme and Siroheme Biosynthesis	Radical SAM family enzyme, similar to coproporphyrinogen III oxidase, oxygen-independent, clustered with nucleoside-triphosphatase RdgB
Cofactors, Vitamins, Prosthetic Groups, Pigments	Tetrapyrroles	Heme and Siroheme Biosynthesis	Uroporphyrinogen III decarboxylase (EC 4.1.1.37)
Cofactors, Vitamins, Prosthetic Groups, Pigments	Tetrapyrroles	Heme and Siroheme Biosynthesis	Uroporphyrinogen-III methyltransferase (EC 2.1.1.107)
Cofactors, Vitamins, Prosthetic Groups, Pigments	Tetrapyrroles	Heme and Siroheme Biosynthesis	Uroporphyrinogen-III synthase (EC 4.2.1.75)

Cofactors, Vitamins, Prosthetic Groups, Pigments	no subcategory	Thiamin biosynthesis	1-deoxy-D-xylulose 5-phosphate synthase (EC 2.2.1.7)
Cofactors, Vitamins, Prosthetic Groups, Pigments	no subcategory	Thiamin biosynthesis	Hydroxymethylpyrimidine ABC transporter, transmembrane component
Cofactors, Vitamins, Prosthetic Groups, Pigments	no subcategory	Thiamin biosynthesis	Thiamin-phosphate pyrophosphorylase (EC 2.5.1.3)
Cofactors, Vitamins, Prosthetic Groups, Pigments	no subcategory	Thiamin biosynthesis	Thiamine-monophosphate kinase (EC 2.7.4.16)
Cofactors, Vitamins, Prosthetic Groups, Pigments	no subcategory	Thiamin biosynthesis	Thiazole biosynthesis protein ThiG
DNA Metabolism	DNA recombination	RuvABC plus a hypothetical	Crossover junction endodeoxyribonuclease RuvC (EC 3.1.22.4)
DNA Metabolism	DNA recombination	RuvABC plus a hypothetical	FIG000859: hypothetical protein YebC
DNA Metabolism	DNA recombination	RuvABC plus a hypothetical	Holliday junction DNA helicase RuvA
DNA Metabolism	DNA recombination	RuvABC plus a hypothetical	Holliday junction DNA helicase RuvB
DNA Metabolism	DNA repair	2-phosphoglycolate salvage	Phosphoglycolate phosphatase (EC 3.1.3.18)
DNA Metabolism	DNA repair	2-phosphoglycolate salvage	Similar to phosphoglycolate phosphatase, clustered with ribosomal large subunit pseudouridine synthase C
DNA Metabolism	DNA repair	DNA Repair Base Excision	DNA ligase (EC 6.5.1.2)
DNA Metabolism	DNA repair	DNA Repair Base Excision	DNA polymerase I (EC 2.7.7.7)
DNA Metabolism	DNA repair	DNA Repair Base Excision	DNA-3-methyladenine glycosylase (EC 3.2.2.20)
DNA Metabolism	DNA repair	DNA Repair Base Excision	Endonuclease III (EC 4.2.99.18)
DNA Metabolism	DNA repair	DNA Repair Base Excision	Formamidopyrimidine-DNA glycosylase (EC 3.2.2.23)
DNA Metabolism	DNA repair	DNA Repair Base Excision	Single-stranded-DNA-specific exonuclease RecJ (EC 3.1.-.-)
DNA Metabolism	DNA repair	DNA repair, UvrABC system	Excinuclease ABC subunit A
DNA Metabolism	DNA repair	DNA repair, bacterial	A/G-specific adenine glycosylase (EC 3.2.2.-)
DNA Metabolism	DNA repair	DNA repair, bacterial	Alkylated DNA repair protein AlkB
DNA Metabolism	DNA repair	DNA repair, bacterial	DNA polymerase IV (EC 2.7.7.7)
DNA Metabolism	DNA repair	DNA repair, bacterial	DNA recombination protein RmuC
DNA Metabolism	DNA repair	DNA repair, bacterial	DNA repair protein RadA
DNA Metabolism	DNA repair	DNA repair, bacterial	DNA repair protein RadC
DNA Metabolism	DNA repair	DNA repair, bacterial	DNA repair protein RecN
DNA Metabolism	DNA repair	DNA repair, bacterial	Error-prone repair protein ImuA
DNA Metabolism	DNA repair	DNA repair, bacterial	Exodeoxyribonuclease III (EC 3.1.11.2)
DNA Metabolism	DNA repair	DNA repair, bacterial	Exodeoxyribonuclease VII large subunit (EC 3.1.11.6)
DNA Metabolism	DNA repair	DNA repair, bacterial	Exodeoxyribonuclease VII small subunit (EC 3.1.11.6)
DNA Metabolism	DNA repair	DNA repair, bacterial	Methylated-DNA--protein-cysteine methyltransferase (EC 2.1.1.63)
DNA Metabolism	DNA repair	DNA repair, bacterial	RecA protein
DNA Metabolism	DNA repair	DNA repair, bacterial	SOS-response repressor and protease LexA (EC 3.4.21.88)
DNA Metabolism	DNA repair	DNA repair, bacterial	Single-stranded DNA-binding protein
DNA Metabolism	DNA repair	DNA repair, bacterial DinG and relatives	DinG family ATP-dependent helicase YoaA
DNA Metabolism	DNA repair	DNA repair, bacterial MutL-MutS system	DNA mismatch repair protein MutL
DNA Metabolism	DNA repair	DNA repair, bacterial MutL-MutS system	DNA mismatch repair protein MutS
DNA Metabolism	DNA repair	DNA repair, bacterial RecFOR pathway	ATP-dependent DNA helicase RecQ
DNA Metabolism	DNA repair	DNA repair, bacterial RecFOR pathway	DNA recombination and repair protein RecF
DNA Metabolism	DNA repair	DNA repair, bacterial RecFOR pathway	DNA recombination and repair protein RecO
DNA Metabolism	DNA repair	DNA repair, bacterial RecFOR pathway	RecA protein
DNA Metabolism	DNA repair	DNA repair, bacterial RecFOR pathway	Recombination protein RecR
DNA Metabolism	DNA repair	DNA repair, bacterial RecFOR pathway	Single-stranded DNA-binding protein
DNA Metabolism	DNA repair	DNA repair, bacterial RecFOR pathway	Single-stranded-DNA-specific exonuclease RecJ (EC 3.1.-.-)
DNA Metabolism	DNA repair	DNA repair, bacterial UvrD and related helicases	ATP-dependent DNA helicase UvrD/PcrA
DNA Metabolism	DNA repair	DNA repair, bacterial photolyase	Deoxyribodipyrimidine photolyase (EC 4.1.99.3)
DNA Metabolism	DNA repair	Uracil-DNA glycosylase	Uracil-DNA glycosylase, family 4
DNA Metabolism	DNA repair	Uracil-DNA glycosylase	Uracil-DNA glycosylase, family 5
DNA Metabolism	DNA replication	DNA-replication	ATP-dependent DNA helicase RecG (EC 3.6.1.-)
DNA Metabolism	DNA replication	DNA-replication	ATP-dependent DNA helicase RecQ
DNA Metabolism	DNA replication	DNA-replication	Chromosomal replication initiator protein DnaA
DNA Metabolism	DNA replication	DNA-replication	Crossover junction endodeoxyribonuclease RuvC (EC 3.1.22.4)
DNA Metabolism	DNA replication	DNA-replication	DNA polymerase I (EC 2.7.7.7)
DNA Metabolism	DNA replication	DNA-replication	DNA polymerase III alpha subunit (EC 2.7.7.7)
DNA Metabolism	DNA replication	DNA-replication	DNA polymerase III beta subunit (EC 2.7.7.7)
DNA Metabolism	DNA replication	DNA-replication	DNA polymerase III chi subunit (EC 2.7.7.7)
DNA Metabolism	DNA replication	DNA-replication	DNA polymerase III delta prime subunit (EC 2.7.7.7)
DNA Metabolism	DNA replication	DNA-replication	DNA polymerase III delta subunit (EC 2.7.7.7)
DNA Metabolism	DNA replication	DNA-replication	DNA polymerase III epsilon subunit (EC 2.7.7.7)
DNA Metabolism	DNA replication	DNA-replication	DNA polymerase III subunits gamma and tau (EC 2.7.7.7)
DNA Metabolism	DNA replication	DNA-replication	DNA primase (EC 2.7.7.-)
DNA Metabolism	DNA replication	DNA-replication	DNA repair protein RecN
DNA Metabolism	DNA replication	DNA-replication	Helicase PriA essential for oriC/DnaA-independent DNA replication
DNA Metabolism	DNA replication	DNA-replication	Holliday junction DNA helicase RuvA
DNA Metabolism	DNA replication	DNA-replication	Holliday junction DNA helicase RuvB
DNA Metabolism	DNA replication	DNA-replication	RecA protein
DNA Metabolism	DNA replication	DNA-replication	Recombination protein RecR
DNA Metabolism	DNA replication	DNA-replication	Replicative DNA helicase (EC 3.6.1.-)
DNA Metabolism	DNA replication	DNA-replication	Single-stranded-DNA-specific exonuclease RecJ (EC 3.1.-.-)
DNA Metabolism	DNA replication	DNA-replication	Transcription-repair coupling factor
DNA Metabolism	DNA replication	DNA topoisomerases, Type I, ATP-independent	DNA topoisomerase I (EC 5.99.1.2)
DNA Metabolism	DNA replication	DNA topoisomerases, Type II, ATP-dependent	DNA gyrase subunit A (EC 5.99.1.3)
DNA Metabolism	DNA replication	DNA topoisomerases, Type II, ATP-dependent	DNA gyrase subunit B (EC 5.99.1.3)
DNA Metabolism	DNA replication	DNA topoisomerases, Type II, ATP-dependent	Topoisomerase IV subunit A (EC 5.99.1.-)

DNA Metabolism	DNA replication	DNA topoisomerases, Type II, ATP-dependent	Topoisomerase IV subunit B (EC 5.99.1.-)
DNA Metabolism	DNA uptake, competence	DNA processing cluster	DNA polymerase III subunits gamma and tau (EC 2.7.7.7)
DNA Metabolism	DNA uptake, competence	DNA processing cluster	FIG000557: hypothetical protein co-occurring with RecR
DNA Metabolism	DNA uptake, competence	DNA processing cluster	Recombination protein RecR
DNA Metabolism	no subcategory	DNA structural proteins, bacterial	Chromosome partition protein smc
DNA Metabolism	no subcategory	DNA structural proteins, bacterial	DNA-binding protein HU-beta
DNA Metabolism	no subcategory	DNA structural proteins, bacterial	Integration host factor alpha subunit
DNA Metabolism	no subcategory	DNA structural proteins, bacterial	Integration host factor beta subunit
DNA Metabolism	no subcategory	Restriction-Modification System	Putative predicted metal-dependent hydrolase
DNA Metabolism	no subcategory	Restriction-Modification System	Type I restriction-modification system, DNA-methyltransferase subunit M (EC 2.1.1.72)
DNA Metabolism	no subcategory	Restriction-Modification System	Type I restriction-modification system, restriction subunit R (EC 3.1.21.3)
DNA Metabolism	no subcategory	Restriction-Modification System	Type I restriction-modification system, specificity subunit S (EC 3.1.21.3)
DNA Metabolism	no subcategory	YcfH	Putative deoxyribonuclease YcfH
Fatty Acids, Lipids, and Isoprenoids	Fatty acids	Acyl-CoA thioesterase II	Acyl-CoA thioesterase II (EC 3.1.2.-)
Fatty Acids, Lipids, and Isoprenoids	Fatty acids	Acyl-CoA thioesterase II	TesB-like acyl-CoA thioesterase 2
Fatty Acids, Lipids, and Isoprenoids	Fatty acids	Acyl-CoA thioesterase II	TesB-like acyl-CoA thioesterase 5
Fatty Acids, Lipids, and Isoprenoids	Fatty acids	Fatty Acid Biosynthesis FASII	3-hydroxyacyl-[acyl-carrier-protein] dehydratase, FabA form (EC 4.2.1.59)
Fatty Acids, Lipids, and Isoprenoids	Fatty acids	Fatty Acid Biosynthesis FASII	3-hydroxyacyl-[acyl-carrier-protein] dehydratase, FabZ form (EC 4.2.1.59)
Fatty Acids, Lipids, and Isoprenoids	Fatty acids	Fatty Acid Biosynthesis FASII	3-oxoacyl-[acyl-carrier protein] reductase (EC 1.1.1.100)
Fatty Acids, Lipids, and Isoprenoids	Fatty acids	Fatty Acid Biosynthesis FASII	3-oxoacyl-[acyl-carrier-protein] synthase, KASI (EC 2.3.1.41)
Fatty Acids, Lipids, and Isoprenoids	Fatty acids	Fatty Acid Biosynthesis FASII	Acetyl-coenzyme A carboxyl transferase alpha chain (EC 6.4.1.2)
Fatty Acids, Lipids, and Isoprenoids	Fatty acids	Fatty Acid Biosynthesis FASII	Acetyl-coenzyme A carboxyl transferase beta chain (EC 6.4.1.2)
Fatty Acids, Lipids, and Isoprenoids	Fatty acids	Fatty Acid Biosynthesis FASII	Acyl carrier protein
Fatty Acids, Lipids, and Isoprenoids	Fatty acids	Fatty Acid Biosynthesis FASII	Biotin carboxyl carrier protein of acetyl-CoA carboxylase
Fatty Acids, Lipids, and Isoprenoids	Fatty acids	Fatty Acid Biosynthesis FASII	Biotin carboxylase of acetyl-CoA carboxylase (EC 6.3.4.14)
Fatty Acids, Lipids, and Isoprenoids	Fatty acids	Fatty Acid Biosynthesis FASII	Enoyl-[acyl-carrier-protein] reductase [FMN] (EC 1.3.1.9)
Fatty Acids, Lipids, and Isoprenoids	Fatty acids	Fatty Acid Biosynthesis FASII	Enoyl-[acyl-carrier-protein] reductase [NADH] (EC 1.3.1.9)
Fatty Acids, Lipids, and Isoprenoids	Fatty acids	Fatty Acid Biosynthesis FASII	Holo-[acyl-carrier protein] synthase (EC 2.7.8.7)
Fatty Acids, Lipids, and Isoprenoids	Fatty acids	Fatty Acid Biosynthesis FASII	Malonyl CoA-acyl carrier protein transacylase (EC 2.3.1.39)
Fatty Acids, Lipids, and Isoprenoids	Fatty acids	Fatty acid metabolism cluster	3-hydroxyacyl-CoA dehydrogenase (EC 1.1.1.35)
Fatty Acids, Lipids, and Isoprenoids	Fatty acids	Fatty acid metabolism cluster	3-hydroxybutyryl-CoA epimerase (EC 5.1.2.3)
Fatty Acids, Lipids, and Isoprenoids	Fatty acids	Fatty acid metabolism cluster	3-ketoacyl-CoA thiolase (EC 2.3.1.16)
Fatty Acids, Lipids, and Isoprenoids	Fatty acids	Fatty acid metabolism cluster	Enoyl-CoA hydratase (EC 4.2.1.17)
Fatty Acids, Lipids, and Isoprenoids	Fatty acids	Fatty acid metabolism cluster	Long-chain-fatty-acid--CoA ligase (EC 6.2.1.3)
Fatty Acids, Lipids, and Isoprenoids	Isoprenoids	Isoprenoid Biosynthesis	(2E,6E)-farnesyl diphosphate synthase (EC 2.5.1.10)
Fatty Acids, Lipids, and Isoprenoids	Isoprenoids	Isoprenoid Biosynthesis	1-deoxy-D-xylulose 5-phosphate reductoisomerase (EC 1.1.1.267)
Fatty Acids, Lipids, and Isoprenoids	Isoprenoids	Isoprenoid Biosynthesis	1-deoxy-D-xylulose 5-phosphate synthase (EC 2.2.1.7)
Fatty Acids, Lipids, and Isoprenoids	Isoprenoids	Isoprenoid Biosynthesis	1-hydroxy-2-methyl-2-(E)-butenyl 4-diphosphate synthase (EC 1.17.7.1)
Fatty Acids, Lipids, and Isoprenoids	Isoprenoids	Isoprenoid Biosynthesis	2-C-methyl-D-erythritol 2,4-cyclodiphosphate synthase (EC 4.6.1.12)
Fatty Acids, Lipids, and Isoprenoids	Isoprenoids	Isoprenoid Biosynthesis	2-C-methyl-D-erythritol 4-phosphate cytidylyltransferase (EC 2.7.7.60)
Fatty Acids, Lipids, and Isoprenoids	Isoprenoids	Isoprenoid Biosynthesis	4-diphosphocytidyl-2-C-methyl-D-erythritol kinase (EC 2.7.1.148)
Fatty Acids, Lipids, and Isoprenoids	Isoprenoids	Isoprenoid Biosynthesis	4-hydroxy-3-methylbut-2-enyl diphosphate reductase (EC 1.17.1.2)
Fatty Acids, Lipids, and Isoprenoids	Isoprenoids	Isoprenoid Biosynthesis	Acetyl-CoA acetyltransferase (EC 2.3.1.9)
Fatty Acids, Lipids, and Isoprenoids	Isoprenoids	Isoprenoid Biosynthesis	Dimethylallyltransferase (EC 2.5.1.1)
Fatty Acids, Lipids, and Isoprenoids	Isoprenoids	Isoprenoid Biosynthesis	Hydroxymethylglutaryl-CoA synthase (EC 2.3.3.10)
Fatty Acids, Lipids, and Isoprenoids	Isoprenoids	Isoprenoid Biosynthesis	Undecaprenyl diphosphate synthase (EC 2.5.1.31)
Fatty Acids, Lipids, and Isoprenoids	Isoprenoids	Isoprenoid Biosynthesis: Interconversions	(2E,6E)-farnesyl diphosphate synthase (EC 2.5.1.10)
Fatty Acids, Lipids, and Isoprenoids	Isoprenoids	Isoprenoinds for Quinones	(2E,6E)-farnesyl diphosphate synthase (EC 2.5.1.10)
Fatty Acids, Lipids, and Isoprenoids	Isoprenoids	Isoprenoinds for Quinones	Decaprenyl diphosphate synthase (EC 2.5.1.91)
Fatty Acids, Lipids, and Isoprenoids	Isoprenoids	Isoprenoinds for Quinones	Dimethylallyltransferase (EC 2.5.1.1)

Fatty Acids, Lipids, and Isoprenoids	Isoprenoids	Isoprenoinds for Quinones	Octaprenyl diphosphate synthase (EC 2.5.1.90)
Fatty Acids, Lipids, and Isoprenoids	Isoprenoids	Isoprenoinds for Quinones	Undecaprenyl diphosphate synthase (EC 2.5.1.31)
Fatty Acids, Lipids, and Isoprenoids	Isoprenoids	Nonmevalonate Branch of Isoprenoid Biosynthesis	1-deoxy-D-xylulose 5-phosphate reductoisomerase (EC 1.1.1.267)
Fatty Acids, Lipids, and Isoprenoids	Isoprenoids	Nonmevalonate Branch of Isoprenoid Biosynthesis	1-deoxy-D-xylulose 5-phosphate synthase (EC 2.2.1.7)
Fatty Acids, Lipids, and Isoprenoids	Isoprenoids	Nonmevalonate Branch of Isoprenoid Biosynthesis	1-hydroxy-2-methyl-2-(E)-butenyl 4-diphosphate synthase (EC 1.17.7.1)
Fatty Acids, Lipids, and Isoprenoids	Isoprenoids	Nonmevalonate Branch of Isoprenoid Biosynthesis	2-C-methyl-D-erythritol 2,4-cyclodiphosphate synthase (EC 4.6.1.12)
Fatty Acids, Lipids, and Isoprenoids	Isoprenoids	Nonmevalonate Branch of Isoprenoid Biosynthesis	2-C-methyl-D-erythritol 4-phosphate cytidylyltransferase (EC 2.7.7.60)
Fatty Acids, Lipids, and Isoprenoids	Isoprenoids	Nonmevalonate Branch of Isoprenoid Biosynthesis	4-diphosphocytidyl-2-C-methyl-D-erythritol kinase (EC 2.7.1.148)
Fatty Acids, Lipids, and Isoprenoids	Isoprenoids	Nonmevalonate Branch of Isoprenoid Biosynthesis	4-hydroxy-3-methylbut-2-enyl diphosphate reductase (EC 1.17.1.2)
Fatty Acids, Lipids, and Isoprenoids	Isoprenoids	Polyprenyl Diphosphate Biosynthesis	(2E,6E)-farnesyl diphosphate synthase (EC 2.5.1.10)
Fatty Acids, Lipids, and Isoprenoids	Isoprenoids	Polyprenyl Diphosphate Biosynthesis	Octaprenyl diphosphate synthase (EC 2.5.1.90)
Fatty Acids, Lipids, and Isoprenoids	Isoprenoids	Polyprenyl Diphosphate Biosynthesis	Undecaprenyl diphosphate synthase (EC 2.5.1.31)
Fatty Acids, Lipids, and Isoprenoids	Phospholipids	Glycerolipid and Glycerophospholipid Metabolism in Bacteria	1-acyl-sn-glycerol-3-phosphate acyltransferase (EC 2.3.1.51)
Fatty Acids, Lipids, and Isoprenoids	Phospholipids	Glycerolipid and Glycerophospholipid Metabolism in Bacteria	Acyl carrier protein
Fatty Acids, Lipids, and Isoprenoids	Phospholipids	Glycerolipid and Glycerophospholipid Metabolism in Bacteria	Acyl-phosphate:glycerol-3-phosphate O-acyltransferase PlsY
Fatty Acids, Lipids, and Isoprenoids	Phospholipids	Glycerolipid and Glycerophospholipid Metabolism in Bacteria	Alcohol dehydrogenase (EC 1.1.1.1)
Fatty Acids, Lipids, and Isoprenoids	Phospholipids	Glycerolipid and Glycerophospholipid Metabolism in Bacteria	Aldehyde dehydrogenase (EC 1.2.1.3)
Fatty Acids, Lipids, and Isoprenoids	Phospholipids	Glycerolipid and Glycerophospholipid Metabolism in Bacteria	CDP-diacylglycerol--glycerol-3-phosphate 3-phosphatidyltransferase (EC 2.7.8.5)
Fatty Acids, Lipids, and Isoprenoids	Phospholipids	Glycerolipid and Glycerophospholipid Metabolism in Bacteria	CDP-diacylglycerol--serine O-phosphatidyltransferase (EC 2.7.8.8)
Fatty Acids, Lipids, and Isoprenoids	Phospholipids	Glycerolipid and Glycerophospholipid Metabolism in Bacteria	Glycerol kinase (EC 2.7.1.30)
Fatty Acids, Lipids, and Isoprenoids	Phospholipids	Glycerolipid and Glycerophospholipid Metabolism in Bacteria	Glycerol-3-phosphate dehydrogenase (EC 1.1.5.3)
Fatty Acids, Lipids, and Isoprenoids	Phospholipids	Glycerolipid and Glycerophospholipid Metabolism in Bacteria	Glycerol-3-phosphate dehydrogenase [NAD(P)+] (EC 1.1.1.94)
Fatty Acids, Lipids, and Isoprenoids	Phospholipids	Glycerolipid and Glycerophospholipid Metabolism in Bacteria	Phosphate:acyl-ACP acyltransferase PlsX
Fatty Acids, Lipids, and Isoprenoids	Phospholipids	Glycerolipid and Glycerophospholipid Metabolism in Bacteria	Phosphatidate cytidylyltransferase (EC 2.7.7.41)
Fatty Acids, Lipids, and Isoprenoids	Phospholipids	Glycerolipid and Glycerophospholipid Metabolism in Bacteria	Phosphatidylglycerophosphatase A (EC 3.1.3.27)
Fatty Acids, Lipids, and Isoprenoids	Phospholipids	Glycerolipid and Glycerophospholipid Metabolism in Bacteria	Phosphatidylglycerophosphatase B (EC 3.1.3.27)
Fatty Acids, Lipids, and Isoprenoids	Phospholipids	Glycerolipid and Glycerophospholipid Metabolism in Bacteria	Phosphatidylserine decarboxylase (EC 4.1.1.65)
Fatty Acids, Lipids, and Isoprenoids	Triacylglycerols	Triacylglycerol metabolism	Lysophospholipase (EC 3.1.1.5)
Fatty Acids, Lipids, and Isoprenoids	Triacylglycerols	Triacylglycerol metabolism	Lysophospholipase L2 (EC 3.1.1.5)
Fatty Acids, Lipids, and Isoprenoids	no subcategory	Polyhydroxybutyrate metabolism	3-hydroxyacyl-CoA dehydrogenase (EC 1.1.1.35)
Fatty Acids, Lipids, and Isoprenoids	no subcategory	Polyhydroxybutyrate metabolism	3-hydroxybutyryl-CoA dehydrogenase (EC 1.1.1.157)
Fatty Acids, Lipids, and Isoprenoids	no subcategory	Polyhydroxybutyrate metabolism	3-hydroxybutyryl-CoA epimerase (EC 5.1.2.3)
Fatty Acids, Lipids, and Isoprenoids	no subcategory	Polyhydroxybutyrate metabolism	3-ketoacyl-CoA thiolase (EC 2.3.1.16)
Fatty Acids, Lipids, and Isoprenoids	no subcategory	Polyhydroxybutyrate metabolism	Acetoacetyl-CoA synthetase (EC 6.2.1.16)
Fatty Acids, Lipids, and Isoprenoids	no subcategory	Polyhydroxybutyrate metabolism	Acetyl-CoA acetyltransferase (EC 2.3.1.9)
Fatty Acids, Lipids, and Isoprenoids	no subcategory	Polyhydroxybutyrate metabolism	Enoyl-CoA hydratase (EC 4.2.1.17)
Fatty Acids, Lipids, and Isoprenoids	no subcategory	Polyhydroxybutyrate metabolism	Poly(3-hydroxyalkanoate) depolymerase
Fatty Acids, Lipids, and Isoprenoids	no subcategory	Polyhydroxybutyrate metabolism	Polyhydroxyalkanoic acid synthase
Iron acquisition and metabolism	no subcategory	Encapsulating protein for DyP-type peroxidase and ferritin-like protein oligomers	Uncharacterized protein COG3461
Iron acquisition and metabolism	no subcategory	Hemin transport system	Ferric siderophore transport system, periplasmic binding protein TonB
Iron acquisition and metabolism	no subcategory	Iron acquisition in Streptococcus	Ferric iron ABC transporter, ATP-binding protein
Iron acquisition and metabolism	no subcategory	Iron acquisition in Streptococcus	Ferric iron ABC transporter, iron-binding protein
Iron acquisition and metabolism	no subcategory	Iron acquisition in Streptococcus	Ferric iron ABC transporter, permease protein
Membrane Transport	Cation transporters	Copper Transport System	Copper-translocating P-type ATPase (EC 3.6.3.4)
Membrane Transport	Cation transporters	Copper transport and blue copper proteins	Copper tolerance protein
Membrane Transport	Cation transporters	Magnesium transport	Magnesium and cobalt efflux protein CorC
Membrane Transport	Cation transporters	Magnesium transport	Magnesium and cobalt transport protein CorA
Membrane Transport	Cation transporters	Magnesium transport	Mg/Co/Ni transporter MgtE
Membrane Transport	Protein secretion system, Type II	Widespread colonization island	Flp pilus assembly protein CpaD
Membrane Transport	Protein secretion system, Type II	Widespread colonization island	Flp pilus assembly protein RcpC/CpaB

Membrane Transport	Protein secretion system, Type II	Widespread colonization island	Flp pilus assembly protein TadB
Membrane Transport	Protein secretion system, Type II	Widespread colonization island	Flp pilus assembly protein TadD, contains TPR repeat
Membrane Transport	Protein secretion system, Type II	Widespread colonization island	Flp pilus assembly protein, pilin Flp
Membrane Transport	Protein secretion system, Type II	Widespread colonization island	Similar to TadZ/CpaE, associated with Flp pilus assembly
Membrane Transport	Protein secretion system, Type II	Widespread colonization island	Similar to secretin RcpA/CpaC, associated with Flp pilus assembly
Membrane Transport	Protein secretion system, Type II	Widespread colonization island	Type II/IV secretion system ATP hydrolase TadA/VirB11/CpaF, TadA subfamily
Membrane Transport	Protein secretion system, Type II	Widespread colonization island	Type II/IV secretion system ATPase TadZ/CpaE, associated with Flp pilus assembly
Membrane Transport	Protein secretion system, Type II	Widespread colonization island	Type II/IV secretion system protein TadC, associated with Flp pilus assembly
Membrane Transport	Protein secretion system, Type II	Widespread colonization island	Type II/IV secretion system secretin RcpA/CpaC, associated with Flp pilus assembly
Membrane Transport	Protein secretion system, Type II	Widespread colonization island	Type IV prepilin peptidase TadV/CpaA
Membrane Transport	Protein translocation across cytoplasmic membrane	Bacterial signal recognition particle (SRP)	Signal recognition particle receptor protein FtsY (=alpha subunit) (TC 3.A.5.1.1)
Membrane Transport	Protein translocation across cytoplasmic membrane	Bacterial signal recognition particle (SRP)	Signal recognition particle, subunit Ffh SRP54 (TC 3.A.5.1.1)
Membrane Transport	Protein translocation across cytoplasmic membrane	Twin-arginine translocation system	Twin-arginine translocation protein TatA
Membrane Transport	Protein translocation across cytoplasmic membrane	Twin-arginine translocation system	Twin-arginine translocation protein TatB
Membrane Transport	Protein translocation across cytoplasmic membrane	Twin-arginine translocation system	Twin-arginine translocation protein TatC
Membrane Transport	TRAP transporters	TRAP Transporter unknown substrate 6	TRAP dicarboxylate transporter, DctM subunit, unknown substrate 6
Membrane Transport	TRAP transporters	TRAP Transporter unknown substrate 6	TRAP dicarboxylate transporter, DctQ subunit, unknown substrate 6
Membrane Transport	TRAP transporters	TRAP Transporter unknown substrate 6	TRAP transporter solute receptor, unknown substrate 6
Membrane Transport	TRAP transporters	TRAP Transporter unknown substrate 9	TRAP transporter solute receptor, TAXI family precursor
Metabolism of Aromatic Compounds	Metabolism of central aromatic intermediates	N-heterocyclic aromatic compound degradation	Isoquinoline 1-oxidoreductase alpha subunit (EC 1.3.99.16)
Metabolism of Aromatic Compounds	Metabolism of central aromatic intermediates	N-heterocyclic aromatic compound degradation	Isoquinoline 1-oxidoreductase beta subunit (EC 1.3.99.16)
Metabolism of Aromatic Compounds	Metabolism of central aromatic intermediates	Salicylate and gentisate catabolism	Fumarylacetoacetase (EC 3.7.1.2)
Metabolism of Aromatic Compounds	Metabolism of central aromatic intermediates	Salicylate and gentisate catabolism	Fumarylacetoacetate hydrolase family protein
Metabolism of Aromatic Compounds	Metabolism of central aromatic intermediates	Salicylate and gentisate catabolism	Maleylacetoacetate isomerase (EC 5.2.1.2)
Metabolism of Aromatic Compounds	Peripheral pathways for catabolism of aromatic compounds	Quinate degradation	3-dehydroquinate dehydratase II (EC 4.2.1.10)
Metabolism of Aromatic Compounds	no subcategory	Gentisare degradation	Fumarylacetoacetate hydrolase family protein
Metabolism of Aromatic Compounds	no subcategory	Gentisare degradation	Maleylacetoacetate isomerase (EC 5.2.1.2)
Miscellaneous	Plant-Prokaryote DOE project	Iron-sulfur cluster assembly	Cysteine desulfurase (EC 2.8.1.7)
Miscellaneous	Plant-Prokaryote DOE project	Iron-sulfur cluster assembly	Cysteine desulfurase (EC 2.8.1.7), SufS subfamily
Miscellaneous	Plant-Prokaryote DOE project	Iron-sulfur cluster assembly	Ferredoxin, 2Fe-2S
Miscellaneous	Plant-Prokaryote DOE project	Iron-sulfur cluster assembly	Folate-dependent protein for Fe/S cluster synthesis/repair in oxidative stress
Miscellaneous	Plant-Prokaryote DOE project	Iron-sulfur cluster assembly	Iron binding protein SufA for iron-sulfur cluster assembly
Miscellaneous	Plant-Prokaryote DOE project	Iron-sulfur cluster assembly	Iron-sulfur cluster assembly ATPase protein SufC
Miscellaneous	Plant-Prokaryote DOE project	Iron-sulfur cluster assembly	Iron-sulfur cluster assembly protein SufB
Miscellaneous	Plant-Prokaryote DOE project	Iron-sulfur cluster assembly	Iron-sulfur cluster assembly protein SufD
Miscellaneous	Plant-Prokaryote DOE project	Iron-sulfur cluster assembly	Iron-sulfur cluster regulator IscR
Miscellaneous	Plant-Prokaryote DOE project	Iron-sulfur cluster assembly	Putative iron-sulfur cluster assembly scaffold protein for SUF system, SufE2
Miscellaneous	Plant-Prokaryote DOE project	Iron-sulfur cluster assembly	Scaffold protein for [4Fe-4S] cluster assembly ApbC, MRP-like
Miscellaneous	Plant-Prokaryote DOE project	Iron-sulfur cluster assembly	Sulfur acceptor protein SufE for iron-sulfur cluster assembly
Miscellaneous	Plant-Prokaryote DOE project	Iron-sulfur cluster assembly	probable iron binding protein from the HesB_IscA_SufA family
Miscellaneous	Plant-Prokaryote DOE project	Scaffold proteins for [4Fe-4S] cluster assembly (MRP family)	Glutathione S-transferase family protein
Miscellaneous	Plant-Prokaryote DOE project	Scaffold proteins for [4Fe-4S] cluster assembly (MRP family)	HflC protein
Miscellaneous	Plant-Prokaryote DOE project	Scaffold proteins for [4Fe-4S] cluster assembly (MRP family)	HflK protein
Miscellaneous	Plant-Prokaryote DOE project	Scaffold proteins for [4Fe-4S] cluster assembly (MRP family)	HtrA protease/chaperone protein
Miscellaneous	Plant-Prokaryote DOE project	Scaffold proteins for [4Fe-4S] cluster assembly (MRP family)	Methionyl-tRNA synthetase (EC 6.1.1.10)
Miscellaneous	Plant-Prokaryote DOE project	Scaffold proteins for [4Fe-4S] cluster assembly (MRP family)	S-adenosylmethionine:tRNA ribosyltransferase-isomerase (EC 5.-.-.-)
Miscellaneous	Plant-Prokaryote DOE project	Scaffold proteins for [4Fe-4S] cluster assembly (MRP family)	Scaffold protein for [4Fe-4S] cluster assembly ApbC, MRP-like
Miscellaneous	Plant-Prokaryote DOE project	Scaffold proteins for [4Fe-4S] cluster assembly (MRP family)	TsaE protein, required for threonylcarbamoyladenosine t(6)A37 formation in tRNA
Miscellaneous	Plant-Prokaryote DOE project	Scaffold proteins for [4Fe-4S] cluster assembly (MRP family)	tRNA-guanine transglycosylase (EC 2.4.2.29)
Miscellaneous	no subcategory	Broadly distributed proteins not in subsystems	DUF1022 domain-containing protein
Miscellaneous	no subcategory	Broadly distributed proteins not in subsystems	FIG002473: Protein YcaR in KDO2-Lipid A biosynthesis cluster
Miscellaneous	no subcategory	Broadly distributed proteins not in subsystems	Putative oxidoreductase YncB
Miscellaneous	no subcategory	Broadly distributed proteins not in subsystems	UPF0028 protein YchK
Miscellaneous	no subcategory	Broadly distributed proteins not in subsystems	YciL protein

Miscellaneous	no subcategory	Broadly distributed proteins not in subsystems	YrbA protein
Motility and Chemotaxis	Flagellar motility in Prokaryota	Flagellar motility	Chemotaxis regulator - transmits chemoreceptor signals to flagelllar motor components CheY
Motility and Chemotaxis	Flagellar motility in Prokaryota	Flagellar motility	Flagellar L-ring protein FlgH
Motility and Chemotaxis	Flagellar motility in Prokaryota	Flagellar motility	Flagellar basal-body rod modification protein FlgD
Motility and Chemotaxis	Flagellar motility in Prokaryota	Flagellar motility	Flagellar biosynthesis protein FlhA
Motility and Chemotaxis	Flagellar motility in Prokaryota	Flagellar motility	Flagellar biosynthesis protein FlhB
Motility and Chemotaxis	Flagellar motility in Prokaryota	Flagellar motility	Flagellar biosynthesis protein FliR
Motility and Chemotaxis	Flagellar motility in Prokaryota	Flagellar motility	Flagellar motor rotation protein MotA
Motility and Chemotaxis	Flagellar motility in Prokaryota	Flagellar motility	Flagellar motor rotation protein MotB
Motility and Chemotaxis	Flagellar motility in Prokaryota	Flagellar motility	Flagellar motor switch protein FliM
Motility and Chemotaxis	Flagellar motility in Prokaryota	Flagellar motility	Flagellar motor switch protein FliN
Motility and Chemotaxis	Flagellar motility in Prokaryota	Flagellar motility	Flagellum-specific ATP synthase FliI
Motility and Chemotaxis	Flagellar motility in Prokaryota	Flagellar motility	RNA polymerase sigma-54 factor RpoN
Nitrogen Metabolism	no subcategory	Ammonia assimilation	Ammonium transporter
Nitrogen Metabolism	no subcategory	Ammonia assimilation	Ferredoxin-dependent glutamate synthase (EC 1.4.7.1)
Nitrogen Metabolism	no subcategory	Ammonia assimilation	Glutamate synthase [NADPH] large chain (EC 1.4.1.13)
Nitrogen Metabolism	no subcategory	Ammonia assimilation	Glutamate synthase [NADPH] small chain (EC 1.4.1.13)
Nitrogen Metabolism	no subcategory	Ammonia assimilation	Glutamate-ammonia-ligase adenylyltransferase (EC 2.7.7.42)
Nitrogen Metabolism	no subcategory	Ammonia assimilation	Glutamine synthetase type I (EC 6.3.1.2)
Nitrogen Metabolism	no subcategory	Ammonia assimilation	Nitrogen regulatory protein P-II
Nitrogen Metabolism	no subcategory	Ammonia assimilation	[Protein-PII] uridylyltransferase (EC 2.7.7.59)
Nucleosides and Nucleotides	Detoxification	Housecleaning nucleoside triphosphate pyrophosphatases	Deoxyuridine 5'-triphosphate nucleotidohydrolase (EC 3.6.1.23)
Nucleosides and Nucleotides	Detoxification	Housecleaning nucleoside triphosphate pyrophosphatases	Nucleoside 5-triphosphatase RdgB (dHAPTP, dITP, XTP-specific) (EC 3.6.1.15)
Nucleosides and Nucleotides	Detoxification	Nucleoside triphosphate pyrophosphohydrolase MazG	Nucleoside triphosphate pyrophosphohydrolase MazG (EC 3.6.1.8)
Nucleosides and Nucleotides	Detoxification	Nudix proteins (nucleoside triphosphate hydrolases)	5-methyl-dCTP pyrophosphohydrolase (EC 3.6.1.-)
Nucleosides and Nucleotides	Detoxification	Nudix proteins (nucleoside triphosphate hydrolases)	ADP-ribose pyrophosphatase (EC 3.6.1.13)
Nucleosides and Nucleotides	Detoxification	Nudix proteins (nucleoside triphosphate hydrolases)	Adenosine (5')-pentaphospho-(5'')-adenosine pyrophosphohydrolase (EC 3.6.1.-)
Nucleosides and Nucleotides	Detoxification	Nudix proteins (nucleoside triphosphate hydrolases)	Deoxyuridine 5'-triphosphate nucleotidohydrolase (EC 3.6.1.23)
Nucleosides and Nucleotides	Detoxification	Nudix proteins (nucleoside triphosphate hydrolases)	Hypothetical nudix hydrolase YeaB
Nucleosides and Nucleotides	Detoxification	Nudix proteins (nucleoside triphosphate hydrolases)	NADH pyrophosphatase (EC 3.6.1.22)
Nucleosides and Nucleotides	Purines	CBSS-314260.3.peg.2133	Adenylosuccinate lyase (EC 4.3.2.2)
Nucleosides and Nucleotides	Purines	CBSS-314260.3.peg.2133	FIG040666: hypothetical protein perhaps implicated in de Novo purine biosynthesis
Nucleosides and Nucleotides	Purines	CBSS-314260.3.peg.2133	Phosphoribosylaminoimidazole-succinocarboxamide synthase (EC 6.3.2.6)
Nucleosides and Nucleotides	Purines	CBSS-314260.3.peg.2133	Phosphoribosylformylglycinamidine synthase, PurS subunit (EC 6.3.5.3)
Nucleosides and Nucleotides	Purines	CBSS-314260.3.peg.2133	Phosphoribosylformylglycinamidine synthase, glutamine amidotransferase subunit (EC 6.3.5.3)
Nucleosides and Nucleotides	Purines	CBSS-314260.3.peg.2133	Phosphoribosylformylglycinamidine synthase, synthetase subunit (EC 6.3.5.3)
Nucleosides and Nucleotides	Purines	De Novo Purine Biosynthesis	Adenylosuccinate lyase (EC 4.3.2.2)
Nucleosides and Nucleotides	Purines	De Novo Purine Biosynthesis	Amidophosphoribosyltransferase (EC 2.4.2.14)
Nucleosides and Nucleotides	Purines	De Novo Purine Biosynthesis	IMP cyclohydrolase (EC 3.5.4.10)
Nucleosides and Nucleotides	Purines	De Novo Purine Biosynthesis	Phosphoribosylamine--glycine ligase (EC 6.3.4.13)
Nucleosides and Nucleotides	Purines	De Novo Purine Biosynthesis	Phosphoribosylaminoimidazole carboxylase ATPase subunit (EC 4.1.1.21)
Nucleosides and Nucleotides	Purines	De Novo Purine Biosynthesis	Phosphoribosylaminoimidazole carboxylase catalytic subunit (EC 4.1.1.21)
Nucleosides and Nucleotides	Purines	De Novo Purine Biosynthesis	Phosphoribosylaminoimidazole-succinocarboxamide synthase (EC 6.3.2.6)
Nucleosides and Nucleotides	Purines	De Novo Purine Biosynthesis	Phosphoribosylaminoimidazolecarboxamide formyltransferase (EC 2.1.2.3)
Nucleosides and Nucleotides	Purines	De Novo Purine Biosynthesis	Phosphoribosylformylglycinamidine cyclo-ligase (EC 6.3.3.1)
Nucleosides and Nucleotides	Purines	De Novo Purine Biosynthesis	Phosphoribosylformylglycinamidine synthase, PurS subunit (EC 6.3.5.3)
Nucleosides and Nucleotides	Purines	De Novo Purine Biosynthesis	Phosphoribosylformylglycinamidine synthase, glutamine amidotransferase subunit (EC 6.3.5.3)
Nucleosides and Nucleotides	Purines	De Novo Purine Biosynthesis	Phosphoribosylformylglycinamidine synthase, synthetase subunit (EC 6.3.5.3)
Nucleosides and Nucleotides	Purines	De Novo Purine Biosynthesis	Phosphoribosylglycinamide formyltransferase (EC 2.1.2.2)
Nucleosides and Nucleotides	Purines	De Novo Purine Biosynthesis	Phosphoribosylglycinamide formyltransferase 2 (EC 2.1.2.-)
Nucleosides and Nucleotides	Pyrimidines	De Novo Pyrimidine Synthesis	Aspartate carbamoyltransferase (EC 2.1.3.2)
Nucleosides and Nucleotides	Pyrimidines	De Novo Pyrimidine Synthesis	Carbamoyl-phosphate synthase large chain (EC 6.3.5.5)
Nucleosides and Nucleotides	Pyrimidines	De Novo Pyrimidine Synthesis	Carbamoyl-phosphate synthase small chain (EC 6.3.5.5)
Nucleosides and Nucleotides	Pyrimidines	De Novo Pyrimidine Synthesis	Dihydroorotase (EC 3.5.2.3)
Nucleosides and Nucleotides	Pyrimidines	De Novo Pyrimidine Synthesis	Dihydroorotate dehydrogenase (EC 1.3.3.1)
Nucleosides and Nucleotides	Pyrimidines	De Novo Pyrimidine Synthesis	Orotate phosphoribosyltransferase (EC 2.4.2.10)
Nucleosides and Nucleotides	Pyrimidines	De Novo Pyrimidine Synthesis	Orotidine 5'-phosphate decarboxylase (EC 4.1.1.23)
Nucleosides and Nucleotides	Pyrimidines	pyrimidine conversions	Bis(5'-nucleosyl)-tetraphosphatase (asymmetrical) (EC 3.6.1.17)
Nucleosides and Nucleotides	Pyrimidines	pyrimidine conversions	CTP synthase (EC 6.3.4.2)
Nucleosides and Nucleotides	Pyrimidines	pyrimidine conversions	Cytidylate kinase (EC 2.7.4.25)
Nucleosides and Nucleotides	Pyrimidines	pyrimidine conversions	Deoxycytidine triphosphate deaminase (EC 3.5.4.13)
Nucleosides and Nucleotides	Pyrimidines	pyrimidine conversions	Nucleoside diphosphate kinase (EC 2.7.4.6)
Nucleosides and Nucleotides	Pyrimidines	pyrimidine conversions	Thioredoxin reductase (EC 1.8.1.9)
Nucleosides and Nucleotides	Pyrimidines	pyrimidine conversions	Thymidylate kinase (EC 2.7.4.9)
Nucleosides and Nucleotides	Pyrimidines	pyrimidine conversions	Thymidylate synthase (EC 2.1.1.45)

Nucleosides and Nucleotides	no subcategory	Ribonucleotide reduction	Ribonucleotide reductase of class II (coenzyme B12-dependent) (EC 1.17.4.1)
Nucleosides and Nucleotides	no subcategory	Ribonucleotide reduction	Ribonucleotide reductase of class Ia (aerobic), alpha subunit (EC 1.17.4.1)
Nucleosides and Nucleotides	no subcategory	Ribonucleotide reduction	Ribonucleotide reductase of class Ia (aerobic), beta subunit (EC 1.17.4.1)
Nucleosides and Nucleotides	no subcategory	Ribonucleotide reduction	Ribonucleotide reductase transcriptional regulator NrdR
Phosphorus Metabolism	no subcategory	High affinity phosphate transporter and control of PHO regulon	Phosphate ABC transporter, periplasmic phosphate-binding protein PstS (TC 3.A.1.7.1)
Phosphorus Metabolism	no subcategory	High affinity phosphate transporter and control of PHO regulon	Phosphate regulon sensor protein PhoR (SphS) (EC 2.7.13.3)
Phosphorus Metabolism	no subcategory	High affinity phosphate transporter and control of PHO regulon	Phosphate regulon transcriptional regulatory protein PhoB (SphR)
Phosphorus Metabolism	no subcategory	High affinity phosphate transporter and control of PHO regulon	Phosphate transport ATP-binding protein PstB (TC 3.A.1.7.1)
Phosphorus Metabolism	no subcategory	High affinity phosphate transporter and control of PHO regulon	Phosphate transport system permease protein PstA (TC 3.A.1.7.1)
Phosphorus Metabolism	no subcategory	High affinity phosphate transporter and control of PHO regulon	Phosphate transport system permease protein PstC (TC 3.A.1.7.1)
Phosphorus Metabolism	no subcategory	High affinity phosphate transporter and control of PHO regulon	Phosphate transport system regulatory protein PhoU
Phosphorus Metabolism	no subcategory	High affinity phosphate transporter and control of PHO regulon	Polyphosphate kinase (EC 2.7.4.1)
Phosphorus Metabolism	no subcategory	Phosphate metabolism	Exopolyphosphatase (EC 3.6.1.11)
Phosphorus Metabolism	no subcategory	Phosphate metabolism	Inorganic pyrophosphatase (EC 3.6.1.1)
Phosphorus Metabolism	no subcategory	Phosphate metabolism	NAD(P) transhydrogenase alpha subunit (EC 1.6.1.2)
Phosphorus Metabolism	no subcategory	Phosphate metabolism	NAD(P) transhydrogenase subunit beta (EC 1.6.1.2)
Phosphorus Metabolism	no subcategory	Phosphate metabolism	Phosphate ABC transporter, periplasmic phosphate-binding protein PstS (TC 3.A.1.7.1)
Phosphorus Metabolism	no subcategory	Phosphate metabolism	Phosphate regulon sensor protein PhoR (SphS) (EC 2.7.13.3)
Phosphorus Metabolism	no subcategory	Phosphate metabolism	Phosphate regulon transcriptional regulatory protein PhoB (SphR)
Phosphorus Metabolism	no subcategory	Phosphate metabolism	Phosphate starvation-inducible protein PhoH, predicted ATPase
Phosphorus Metabolism	no subcategory	Phosphate metabolism	Phosphate transport ATP-binding protein PstB (TC 3.A.1.7.1)
Phosphorus Metabolism	no subcategory	Phosphate metabolism	Phosphate transport system permease protein PstA (TC 3.A.1.7.1)
Phosphorus Metabolism	no subcategory	Phosphate metabolism	Phosphate transport system permease protein PstC (TC 3.A.1.7.1)
Phosphorus Metabolism	no subcategory	Phosphate metabolism	Phosphate transport system regulatory protein PhoU
Phosphorus Metabolism	no subcategory	Phosphate metabolism	Polyphosphate kinase (EC 2.7.4.1)
Phosphorus Metabolism	no subcategory	Phosphate metabolism	Pyrophosphate-energized proton pump (EC 3.6.1.1)
Phosphorus Metabolism	no subcategory	Phosphate metabolism	response regulator in two-component regulatory system with PhoQ
Phosphorus Metabolism	no subcategory	Polyphosphate	Exopolyphosphatase (EC 3.6.1.11)
Phosphorus Metabolism	no subcategory	Polyphosphate	Polyphosphate kinase (EC 2.7.4.1)
Potassium metabolism	no subcategory	Hyperosmotic potassium uptake	Potassium uptake protein TrkH
Potassium metabolism	no subcategory	Hyperosmotic potassium uptake	Trk system potassium uptake protein TrkA
Protein Metabolism	Protein biosynthesis	Glycyl-tRNA synthetase	Glycyl-tRNA synthetase alpha chain (EC 6.1.1.14)
Protein Metabolism	Protein biosynthesis	Glycyl-tRNA synthetase	Glycyl-tRNA synthetase beta chain (EC 6.1.1.14)
Protein Metabolism	Protein biosynthesis	Programmed frameshift	Peptide chain release factor 2
Protein Metabolism	Protein biosynthesis	Ribosome LSU bacterial	LSU ribosomal protein L10p (P0)
Protein Metabolism	Protein biosynthesis	Ribosome LSU bacterial	LSU ribosomal protein L11p (L12e)
Protein Metabolism	Protein biosynthesis	Ribosome LSU bacterial	LSU ribosomal protein L13p (L13Ae)
Protein Metabolism	Protein biosynthesis	Ribosome LSU bacterial	LSU ribosomal protein L14p (L23e)
Protein Metabolism	Protein biosynthesis	Ribosome LSU bacterial	LSU ribosomal protein L15p (L27Ae)
Protein Metabolism	Protein biosynthesis	Ribosome LSU bacterial	LSU ribosomal protein L16p (L10e)
Protein Metabolism	Protein biosynthesis	Ribosome LSU bacterial	LSU ribosomal protein L17p
Protein Metabolism	Protein biosynthesis	Ribosome LSU bacterial	LSU ribosomal protein L18p (L5e)
Protein Metabolism	Protein biosynthesis	Ribosome LSU bacterial	LSU ribosomal protein L19p
Protein Metabolism	Protein biosynthesis	Ribosome LSU bacterial	LSU ribosomal protein L1p (L10Ae)
Protein Metabolism	Protein biosynthesis	Ribosome LSU bacterial	LSU ribosomal protein L20p
Protein Metabolism	Protein biosynthesis	Ribosome LSU bacterial	LSU ribosomal protein L21p
Protein Metabolism	Protein biosynthesis	Ribosome LSU bacterial	LSU ribosomal protein L22p (L17e)
Protein Metabolism	Protein biosynthesis	Ribosome LSU bacterial	LSU ribosomal protein L23p (L23Ae)
Protein Metabolism	Protein biosynthesis	Ribosome LSU bacterial	LSU ribosomal protein L24p (L26e)
Protein Metabolism	Protein biosynthesis	Ribosome LSU bacterial	LSU ribosomal protein L27p
Protein Metabolism	Protein biosynthesis	Ribosome LSU bacterial	LSU ribosomal protein L28p
Protein Metabolism	Protein biosynthesis	Ribosome LSU bacterial	LSU ribosomal protein L29p (L35e)
Protein Metabolism	Protein biosynthesis	Ribosome LSU bacterial	LSU ribosomal protein L2p (L8e)
Protein Metabolism	Protein biosynthesis	Ribosome LSU bacterial	LSU ribosomal protein L30p (L7e)
Protein Metabolism	Protein biosynthesis	Ribosome LSU bacterial	LSU ribosomal protein L31p
Protein Metabolism	Protein biosynthesis	Ribosome LSU bacterial	LSU ribosomal protein L31p, zinc-independent
Protein Metabolism	Protein biosynthesis	Ribosome LSU bacterial	LSU ribosomal protein L32p
Protein Metabolism	Protein biosynthesis	Ribosome LSU bacterial	LSU ribosomal protein L33p
Protein Metabolism	Protein biosynthesis	Ribosome LSU bacterial	LSU ribosomal protein L33p, zinc-independent
Protein Metabolism	Protein biosynthesis	Ribosome LSU bacterial	LSU ribosomal protein L34p
Protein Metabolism	Protein biosynthesis	Ribosome LSU bacterial	LSU ribosomal protein L35p
Protein Metabolism	Protein biosynthesis	Ribosome LSU bacterial	LSU ribosomal protein L36p
Protein Metabolism	Protein biosynthesis	Ribosome LSU bacterial	LSU ribosomal protein L3p (L3e)
Protein Metabolism	Protein biosynthesis	Ribosome LSU bacterial	LSU ribosomal protein L4p (L1e)
Protein Metabolism	Protein biosynthesis	Ribosome LSU bacterial	LSU ribosomal protein L5p (L11e)
Protein Metabolism	Protein biosynthesis	Ribosome LSU bacterial	LSU ribosomal protein L6p (L9e)
Protein Metabolism	Protein biosynthesis	Ribosome LSU bacterial	LSU ribosomal protein L7/L12 (P1/P2)
Protein Metabolism	Protein biosynthesis	Ribosome LSU bacterial	LSU ribosomal protein L9p
Protein Metabolism	Protein biosynthesis	Ribosome SSU bacterial	SSU ribosomal protein S10p (S20e)
Protein Metabolism	Protein biosynthesis	Ribosome SSU bacterial	SSU ribosomal protein S11p (S14e)
Protein Metabolism	Protein biosynthesis	Ribosome SSU bacterial	SSU ribosomal protein S12p (S23e)
Protein Metabolism	Protein biosynthesis	Ribosome SSU bacterial	SSU ribosomal protein S14p (S29e)
Protein Metabolism	Protein biosynthesis	Ribosome SSU bacterial	SSU ribosomal protein S14p (S29e), zinc-independent
Protein Metabolism	Protein biosynthesis	Ribosome SSU bacterial	SSU ribosomal protein S15p (S13e)
Protein Metabolism	Protein biosynthesis	Ribosome SSU bacterial	SSU ribosomal protein S16p

Protein Metabolism	Protein biosynthesis	Ribosome SSU bacterial	SSU ribosomal protein S17p (S11e)
Protein Metabolism	Protein biosynthesis	Ribosome SSU bacterial	SSU ribosomal protein S18p
Protein Metabolism	Protein biosynthesis	Ribosome SSU bacterial	SSU ribosomal protein S18p, zinc-independent
Protein Metabolism	Protein biosynthesis	Ribosome SSU bacterial	SSU ribosomal protein S19p (S15e)
Protein Metabolism	Protein biosynthesis	Ribosome SSU bacterial	SSU ribosomal protein S1p
Protein Metabolism	Protein biosynthesis	Ribosome SSU bacterial	SSU ribosomal protein S20p
Protein Metabolism	Protein biosynthesis	Ribosome SSU bacterial	SSU ribosomal protein S21p
Protein Metabolism	Protein biosynthesis	Ribosome SSU bacterial	SSU ribosomal protein S2p (SAe)
Protein Metabolism	Protein biosynthesis	Ribosome SSU bacterial	SSU ribosomal protein S3p (S3e)
Protein Metabolism	Protein biosynthesis	Ribosome SSU bacterial	SSU ribosomal protein S4p (S9e)
Protein Metabolism	Protein biosynthesis	Ribosome SSU bacterial	SSU ribosomal protein S5p (S2e)
Protein Metabolism	Protein biosynthesis	Ribosome SSU bacterial	SSU ribosomal protein S6p
Protein Metabolism	Protein biosynthesis	Ribosome SSU bacterial	SSU ribosomal protein S7p (S5e)
Protein Metabolism	Protein biosynthesis	Ribosome SSU bacterial	SSU ribosomal protein S8p (S15Ae)
Protein Metabolism	Protein biosynthesis	Ribosome SSU bacterial	SSU ribosomal protein S9p (S16e)
Protein Metabolism	Protein biosynthesis	Ribosome activity modulation	Ribosomal subunit interface protein
Protein Metabolism	Protein biosynthesis	Ribosome biogenesis bacterial	16S rRNA processing protein RimM
Protein Metabolism	Protein biosynthesis	Ribosome biogenesis bacterial	LSU m3Psi1915 methyltransferase RlmH
Protein Metabolism	Protein biosynthesis	Ribosome biogenesis bacterial	Ribonuclease E (EC 3.1.26.12)
Protein Metabolism	Protein biosynthesis	Ribosome biogenesis bacterial	Ribosomal large subunit pseudouridine synthase A (EC 4.2.1.70)
Protein Metabolism	Protein biosynthesis	Ribosome biogenesis bacterial	Ribosomal large subunit pseudouridine synthase C (EC 4.2.1.70)
Protein Metabolism	Protein biosynthesis	Ribosome biogenesis bacterial	Ribosomal large subunit pseudouridine synthase D (EC 4.2.1.70)
Protein Metabolism	Protein biosynthesis	Ribosome biogenesis bacterial	Ribosomal protein L11 methyltransferase (EC 2.1.1.-)
Protein Metabolism	Protein biosynthesis	Ribosome biogenesis bacterial	Ribosomal-protein-S18p-alanine acetyltransferase (EC 2.3.1.-)
Protein Metabolism	Protein biosynthesis	Ribosome biogenesis bacterial	Ribosomal-protein-S5p-alanine acetyltransferase
Protein Metabolism	Protein biosynthesis	Ribosome biogenesis bacterial	SSU rRNA (adenine(1518)-N(6)/adenine(1519)-N(6))-dimethyltransferase (EC 2.1.1.182)
Protein Metabolism	Protein biosynthesis	Ribosome biogenesis bacterial	TsaB protein, required for threonylcarbamoyladenosine (t(6)A) formation in tRNA
Protein Metabolism	Protein biosynthesis	Ribosome biogenesis bacterial	tRNA (Guanine37-N1) -methyltransferase (EC 2.1.1.31)
Protein Metabolism	Protein biosynthesis	Translation elongation factors bacterial	Translation elongation factor G
Protein Metabolism	Protein biosynthesis	Translation elongation factors bacterial	Translation elongation factor LepA
Protein Metabolism	Protein biosynthesis	Translation elongation factors bacterial	Translation elongation factor P
Protein Metabolism	Protein biosynthesis	Translation elongation factors bacterial	Translation elongation factor Ts
Protein Metabolism	Protein biosynthesis	Translation elongation factors bacterial	Translation elongation factor Tu
Protein Metabolism	Protein biosynthesis	Translation initiation factors bacterial	Methionyl-tRNA formyltransferase (EC 2.1.2.9)
Protein Metabolism	Protein biosynthesis	Translation initiation factors bacterial	Ribosome-binding factor A
Protein Metabolism	Protein biosynthesis	Translation initiation factors bacterial	Translation initiation factor 1
Protein Metabolism	Protein biosynthesis	Translation initiation factors bacterial	Translation initiation factor 2
Protein Metabolism	Protein biosynthesis	Translation initiation factors bacterial	Translation initiation factor 3
Protein Metabolism	Protein biosynthesis	tRNA aminoacylation, Ala	Alanyl-tRNA synthetase (EC 6.1.1.7)
Protein Metabolism	Protein biosynthesis	tRNA aminoacylation, Arg	Arginyl-tRNA synthetase (EC 6.1.1.19)
Protein Metabolism	Protein biosynthesis	tRNA aminoacylation, Asp and Asn	Aspartyl-tRNA synthetase (EC 6.1.1.12)
Protein Metabolism	Protein biosynthesis	tRNA aminoacylation, Asp and Asn	Aspartyl-tRNA(Asn) amidotransferase subunit A (EC 6.3.5.6)
Protein Metabolism	Protein biosynthesis	tRNA aminoacylation, Asp and Asn	Aspartyl-tRNA(Asn) amidotransferase subunit B (EC 6.3.5.6)
Protein Metabolism	Protein biosynthesis	tRNA aminoacylation, Asp and Asn	Aspartyl-tRNA(Asn) amidotransferase subunit C (EC 6.3.5.6)
Protein Metabolism	Protein biosynthesis	tRNA aminoacylation, Asp and Asn	Aspartyl-tRNA(Asn) synthetase (EC 6.1.1.23)
Protein Metabolism	Protein biosynthesis	tRNA aminoacylation, Cys	Cysteinyl-tRNA synthetase (EC 6.1.1.16)
Protein Metabolism	Protein biosynthesis	tRNA aminoacylation, Glu and Gln	Glutamyl-tRNA synthetase (EC 6.1.1.17)
Protein Metabolism	Protein biosynthesis	tRNA aminoacylation, Glu and Gln	Glutamyl-tRNA(Gln) amidotransferase subunit A (EC 6.3.5.7)
Protein Metabolism	Protein biosynthesis	tRNA aminoacylation, Glu and Gln	Glutamyl-tRNA(Gln) amidotransferase subunit B (EC 6.3.5.7)
Protein Metabolism	Protein biosynthesis	tRNA aminoacylation, Glu and Gln	Glutamyl-tRNA(Gln) amidotransferase subunit C (EC 6.3.5.7)
Protein Metabolism	Protein biosynthesis	tRNA aminoacylation, Glu and Gln	Glutamyl-tRNA(Gln) synthetase (EC 6.1.1.24)
Protein Metabolism	Protein biosynthesis	tRNA aminoacylation, Gly	Glycyl-tRNA synthetase alpha chain (EC 6.1.1.14)
Protein Metabolism	Protein biosynthesis	tRNA aminoacylation, Gly	Glycyl-tRNA synthetase beta chain (EC 6.1.1.14)
Protein Metabolism	Protein biosynthesis	tRNA aminoacylation, His	Histidyl-tRNA synthetase (EC 6.1.1.21)
Protein Metabolism	Protein biosynthesis	tRNA aminoacylation, Ile	Isoleucyl-tRNA synthetase (EC 6.1.1.5)
Protein Metabolism	Protein biosynthesis	tRNA aminoacylation, Leu	Leucyl-tRNA synthetase (EC 6.1.1.4)
Protein Metabolism	Protein biosynthesis	tRNA aminoacylation, Lys	Lysyl-tRNA synthetase (class I) (EC 6.1.1.6)
Protein Metabolism	Protein biosynthesis	tRNA aminoacylation, Met	Methionyl-tRNA synthetase (EC 6.1.1.10)
Protein Metabolism	Protein biosynthesis	tRNA aminoacylation, Phe	Phenylalanyl-tRNA synthetase alpha chain (EC 6.1.1.20)
Protein Metabolism	Protein biosynthesis	tRNA aminoacylation, Phe	Phenylalanyl-tRNA synthetase beta chain (EC 6.1.1.20)
Protein Metabolism	Protein biosynthesis	tRNA aminoacylation, Pro	Prolyl-tRNA synthetase (EC 6.1.1.15), bacterial type
Protein Metabolism	Protein biosynthesis	tRNA aminoacylation, Ser	Seryl-tRNA synthetase (EC 6.1.1.11)
Protein Metabolism	Protein biosynthesis	tRNA aminoacylation, Thr	Threonyl-tRNA synthetase (EC 6.1.1.3)
Protein Metabolism	Protein biosynthesis	tRNA aminoacylation, Trp	Tryptophanyl-tRNA synthetase (EC 6.1.1.2)
Protein Metabolism	Protein biosynthesis	tRNA aminoacylation, Tyr	Tyrosyl-tRNA synthetase (EC 6.1.1.1)
Protein Metabolism	Protein biosynthesis	tRNA aminoacylation, Val	Valyl-tRNA synthetase (EC 6.1.1.9)
Protein Metabolism	Protein degradation	Aminopeptidases (EC 3.4.11.-)	Cytosol aminopeptidase PepA (EC 3.4.11.1)
Protein Metabolism	Protein degradation	Aminopeptidases (EC 3.4.11.-)	Membrane alanine aminopeptidase N (EC 3.4.11.2)
Protein Metabolism	Protein degradation	Aminopeptidases (EC 3.4.11.-)	Peptidase B (EC 3.4.11.23)
Protein Metabolism	Protein degradation	Aminopeptidases (EC 3.4.11.-)	Xaa-Pro aminopeptidase (EC 3.4.11.9)
Protein Metabolism	Protein degradation	Metallocarboxypeptidases (EC 3.4.17.-)	D-alanyl-D-alanine carboxypeptidase (EC 3.4.16.4)
Protein Metabolism	Protein degradation	Protein degradation	Arginine-tRNA-protein transferase (EC 2.3.2.8)
Protein Metabolism	Protein degradation	Protein degradation	Leucyl/phenylalanyl-tRNA--protein transferase (EC 2.3.2.6)
Protein Metabolism	Protein degradation	Proteolysis in bacteria, ATP-dependent	ATP-dependent Clp protease ATP-binding subunit ClpA
Protein Metabolism	Protein degradation	Proteolysis in bacteria, ATP-dependent	ATP-dependent Clp protease ATP-binding subunit ClpX
Protein Metabolism	Protein degradation	Proteolysis in bacteria, ATP-dependent	ATP-dependent Clp protease adaptor protein ClpS
Protein Metabolism	Protein degradation	Proteolysis in bacteria, ATP-dependent	ATP-dependent Clp protease proteolytic subunit (EC 3.4.21.92)
Protein Metabolism	Protein degradation	Proteolysis in bacteria, ATP-dependent	ATP-dependent hsl protease ATP-binding subunit HslU
Protein Metabolism	Protein degradation	Proteolysis in bacteria, ATP-dependent	ATP-dependent protease HslV (EC 3.4.25.-)
Protein Metabolism	Protein degradation	Proteolysis in bacteria, ATP-dependent	ATP-dependent protease La (EC 3.4.21.53) Type I

Protein Metabolism	Protein degradation	Proteolysis in bacteria, ATP-dependent	ClpB protein
Protein Metabolism	Protein degradation	Proteolysis in bacteria, ATP-dependent	DNA repair protein RadA
Protein Metabolism	Protein degradation	Putative TldE-TldD proteolytic complex	TldD protein, part of TldE/TldD proteolytic complex
Protein Metabolism	Protein degradation	Putative TldE-TldD proteolytic complex	TldE protein, part of TldE/TldD proteolytic complex
Protein Metabolism	Protein folding	GroEL GroES	Chaperone protein DnaJ
Protein Metabolism	Protein folding	GroEL GroES	Chaperone protein DnaK
Protein Metabolism	Protein folding	GroEL GroES	Heat shock protein 60 family chaperone GroEL
Protein Metabolism	Protein folding	GroEL GroES	Heat shock protein 60 family co-chaperone GroES
Protein Metabolism	Protein folding	GroEL GroES	Heat shock protein GrpE
Protein Metabolism	Protein folding	GroEL GroES	Heat-inducible transcription repressor HrcA
Protein Metabolism	Protein folding	Peptidyl-prolyl cis-trans isomerase	Foldase protein PrsA precursor (EC 5.2.1.8)
Protein Metabolism	Protein folding	Peptidyl-prolyl cis-trans isomerase	Peptidyl-prolyl cis-trans isomerase PpiD (EC 5.2.1.8)
Protein Metabolism	Protein folding	Peptidyl-prolyl cis-trans isomerase	Survival protein SurA precursor (Peptidyl-prolyl cis-trans isomerase SurA) (EC 5.2.1.8)
Protein Metabolism	Protein folding	Periplasmic disulfide interchange	Cytochrome c-type biogenesis protein CcdA (DsbD analog)
Protein Metabolism	Protein folding	Periplasmic disulfide interchange	Cytochrome c-type biogenesis protein CcmG/DsbE, thiol:disulfide oxidoreductase
Protein Metabolism	Protein folding	Periplasmic disulfide interchange	Periplasmic thiol:disulfide interchange protein DsbA
Protein Metabolism	Protein folding	Periplasmic disulfide interchange	Periplasmic thiol:disulfide oxidoreductase DsbB, required for DsbA reoxidation
Protein Metabolism	Protein folding	Protein chaperones	Chaperone protein DnaJ
Protein Metabolism	Protein folding	Protein chaperones	Chaperone protein DnaK
Protein Metabolism	Protein folding	Protein chaperones	ClpB protein
Protein Metabolism	Protein folding	Protein chaperones	Heat shock protein GrpE
Protein Metabolism	Protein processing and modification	Peptide methionine sulfoxide reductase	Peptide methionine sulfoxide reductase MsrA (EC 1.8.4.11)
Protein Metabolism	Protein processing and modification	Peptide methionine sulfoxide reductase	Peptide methionine sulfoxide reductase MsrB (EC 1.8.4.12)
Protein Metabolism	Protein processing and modification	Protein-L-isoaspartate O-methyltransferase	Protein-L-isoaspartate O-methyltransferase (EC 2.1.1.77)
Protein Metabolism	Protein processing and modification	Ribosomal protein S5p acylation	Ribosomal-protein-S5p-alanine acetyltransferase
Protein Metabolism	Protein processing and modification	Ribosomal protein S5p acylation	SSU ribosomal protein S5p (S2e)
Protein Metabolism	Protein processing and modification	Signal peptidase	Lipoprotein signal peptidase (EC 3.4.23.36)
RNA Metabolism	RNA processing and modification	16S rRNA modification within P site of ribosome	Cell division protein FtsI [Peptidoglycan synthetase] (EC 2.4.1.129)
RNA Metabolism	RNA processing and modification	16S rRNA modification within P site of ribosome	Cell division protein MraZ
RNA Metabolism	RNA processing and modification	16S rRNA modification within P site of ribosome	Penicillin-binding protein 2 (PBP-2)
RNA Metabolism	RNA processing and modification	16S rRNA modification within P site of ribosome	rRNA small subunit methyltransferase H
RNA Metabolism	RNA processing and modification	16S rRNA modification within P site of ribosome	rRNA small subunit methyltransferase I
RNA Metabolism	RNA processing and modification	ATP-dependent RNA helicases, bacterial	ATP-dependent RNA helicase RhlE
RNA Metabolism	RNA processing and modification	Methylthiotransferases	tRNA-i(6)A37 methylthiotransferase
RNA Metabolism	RNA processing and modification	Methylthiotransferases	tRNA-t(6)A37 methylthiotransferase
RNA Metabolism	RNA processing and modification	Possible RNA modification and stress response cluster	4-hydroxythreonine-4-phosphate dehydrogenase (EC 1.1.1.262)
RNA Metabolism	RNA processing and modification	Possible RNA modification and stress response cluster	ApaG protein
RNA Metabolism	RNA processing and modification	Possible RNA modification and stress response cluster	DnaJ-like protein DjlA
RNA Metabolism	RNA processing and modification	Possible RNA modification and stress response cluster	Nucleotidyl transferase possibly involved in threonylcarbamoyladenosine formation
RNA Metabolism	RNA processing and modification	Possible RNA modification and stress response cluster	Organic solvent tolerance protein precursor
RNA Metabolism	RNA processing and modification	Possible RNA modification and stress response cluster	Outer membrane protein Imp, required for envelope biogenesis
RNA Metabolism	RNA processing and modification	Possible RNA modification and stress response cluster	Ribosomal-protein-S18p-alanine acetyltransferase (EC 2.3.1.-)
RNA Metabolism	RNA processing and modification	Possible RNA modification and stress response cluster	SSU rRNA (adenine(1518)-N(6)/adenine(1519)-N(6))-dimethyltransferase (EC 2.1.1.182)
RNA Metabolism	RNA processing and modification	Possible RNA modification and stress response cluster	Survival protein SurA precursor (Peptidyl-prolyl cis-trans isomerase SurA) (EC 5.2.1.8)
RNA Metabolism	RNA processing and modification	Possible RNA modification and stress response cluster	TsaB protein, required for threonylcarbamoyladenosine (t(6)A) formation in tRNA
RNA Metabolism	RNA processing and modification	Possible RNA modification and stress response cluster	TsaD/Kae1/Qri7 protein, required for threonylcarbamoyladenosine t(6)A37 formation in tRNA
RNA Metabolism	RNA processing and modification	Possible RNA modification and stress response cluster	TsaE protein, required for threonylcarbamoyladenosine t(6)A37 formation in tRNA
RNA Metabolism	RNA processing and modification	Queuosine-Archaeosine Biosynthesis	GTP cyclohydrolase I (EC 3.5.4.16) type 1
RNA Metabolism	RNA processing and modification	Queuosine-Archaeosine Biosynthesis	Peptidyl-prolyl cis-trans isomerase (EC 5.2.1.8)
RNA Metabolism	RNA processing and modification	Queuosine-Archaeosine Biosynthesis	Permease of the drug/metabolite transporter (DMT) superfamily
RNA Metabolism	RNA processing and modification	Queuosine-Archaeosine Biosynthesis	Queuosine Biosynthesis QueC ATPase
RNA Metabolism	RNA processing and modification	Queuosine-Archaeosine Biosynthesis	Queuosine Biosynthesis QueE Radical SAM
RNA Metabolism	RNA processing and modification	Queuosine-Archaeosine Biosynthesis	Queuosine biosynthesis QueD, PTPS-I
RNA Metabolism	RNA processing and modification	Queuosine-Archaeosine Biosynthesis	Radical SAM family enzyme, similar to coproporphyrinogen III oxidase, oxygen-independent, clustered with nucleoside-triphosphatase RdgB
RNA Metabolism	RNA processing and modification	Queuosine-Archaeosine Biosynthesis	S-adenosylmethionine:tRNA ribosyltransferase-isomerase (EC 5.-.-.-)
RNA Metabolism	RNA processing and modification	Queuosine-Archaeosine Biosynthesis	glutamyl-Q-tRNA synthetase
RNA Metabolism	RNA processing and modification	Queuosine-Archaeosine Biosynthesis	tRNA-guanine transglycosylase (EC 2.4.2.29)
RNA Metabolism	RNA processing and modification	RNA methylation	16S rRNA (guanine(966)-N(2))-methyltransferase (EC 2.1.1.171)
RNA Metabolism	RNA processing and modification	RNA methylation	23S rRNA (Uracil-5-) -methyltransferase RumA (EC 2.1.1.-)
RNA Metabolism	RNA processing and modification	RNA methylation	23S rRNA (guanosine-2'-O-) -methyltransferase rlmB (EC 2.1.1.-)
RNA Metabolism	RNA processing and modification	RNA methylation	LSU m3Psi1915 methyltransferase RlmH
RNA Metabolism	RNA processing and modification	RNA methylation	Ribosomal RNA large subunit methyltransferase E (EC 2.1.1.-)

RNA Metabolism	RNA processing and modification	RNA methylation	Ribosomal RNA large subunit methyltransferase N (EC 2.1.1.-)
RNA Metabolism	RNA processing and modification	RNA methylation	Ribosomal RNA small subunit methyltransferase E (EC 2.1.1.-)
RNA Metabolism	RNA processing and modification	RNA methylation	SSU rRNA (adenine(1518)-N(6)/adenine(1519)-N(6))-dimethyltransferase (EC 2.1.1.182)
RNA Metabolism	RNA processing and modification	RNA methylation	rRNA small subunit 7-methylguanosine (m7G) methyltransferase GidB
RNA Metabolism	RNA processing and modification	RNA methylation	tRNA (Guanine37-N1) -methyltransferase (EC 2.1.1.31)
RNA Metabolism	RNA processing and modification	RNA methylation	tRNA (adenine37-N(6))-methyltransferase TrmN6 (EC 2.1.1.223)
RNA Metabolism	RNA processing and modification	RNA methylation	tRNA (cytidine(34)-2'-O)-methyltransferase (EC 2.1.1.207)
RNA Metabolism	RNA processing and modification	RNA methylation	tRNA (guanine46-N7-)-methyltransferase (EC 2.1.1.33)
RNA Metabolism	RNA processing and modification	RNA methylation	tRNA-specific 2-thiouridylase MnmA
RNA Metabolism	RNA processing and modification	RNA methylation	tRNA:Cm32/Um32 methyltransferase
RNA Metabolism	RNA processing and modification	RNA processing and degradation, bacterial	3'-to-5' exoribonuclease RNase R
RNA Metabolism	RNA processing and modification	RNA processing and degradation, bacterial	Cytoplasmic axial filament protein CafA and Ribonuclease G (EC 3.1.4.-)
RNA Metabolism	RNA processing and modification	RNA processing and degradation, bacterial	Ribonuclease E (EC 3.1.26.12)
RNA Metabolism	RNA processing and modification	RNA processing and degradation, bacterial	Ribonuclease E inhibitor RraA
RNA Metabolism	RNA processing and modification	RNA processing and degradation, bacterial	Ribonuclease III (EC 3.1.26.3)
RNA Metabolism	RNA processing and modification	RNA processing orphans	2'-5' RNA ligase
RNA Metabolism	RNA processing and modification	RNA pseudouridine syntheses	Ribosomal large subunit pseudouridine synthase A (EC 4.2.1.70)
RNA Metabolism	RNA processing and modification	RNA pseudouridine syntheses	Ribosomal large subunit pseudouridine synthase C (EC 4.2.1.70)
RNA Metabolism	RNA processing and modification	RNA pseudouridine syntheses	Ribosomal large subunit pseudouridine synthase D (EC 4.2.1.70)
RNA Metabolism	RNA processing and modification	RNA pseudouridine syntheses	tRNA pseudouridine synthase A (EC 4.2.1.70)
RNA Metabolism	RNA processing and modification	RNA pseudouridine syntheses	tRNA pseudouridine synthase B (EC 4.2.1.70)
RNA Metabolism	RNA processing and modification	Ribonuclease H	Ribonuclease HI (EC 3.1.26.4)
RNA Metabolism	RNA processing and modification	Ribonuclease H	Ribonuclease HII (EC 3.1.26.4)
RNA Metabolism	RNA processing and modification	Ribonucleases in Bacillus	Metallo-beta-lactamase family protein, RNA-specific
RNA Metabolism	RNA processing and modification	Ribonucleases in Bacillus	Ribonuclease HII (EC 3.1.26.4)
RNA Metabolism	RNA processing and modification	mnm5U34 biosynthesis bacteria	Cysteine desulfurase (EC 2.8.1.7)
RNA Metabolism	RNA processing and modification	mnm5U34 biosynthesis bacteria	Cysteine desulfurase (EC 2.8.1.7), SufS subfamily
RNA Metabolism	RNA processing and modification	mnm5U34 biosynthesis bacteria	GTPase and tRNA-U34 5-formylation enzyme TrmE
RNA Metabolism	RNA processing and modification	mnm5U34 biosynthesis bacteria	tRNA 5-methylaminomethyl-2-thiouridine synthase TusA
RNA Metabolism	RNA processing and modification	mnm5U34 biosynthesis bacteria	tRNA uridine 5-carboxymethylaminomethyl modification enzyme GidA
RNA Metabolism	RNA processing and modification	tRNA modification Bacteria	Cysteine desulfurase (EC 2.8.1.7)
RNA Metabolism	RNA processing and modification	tRNA modification Bacteria	Cysteine desulfurase (EC 2.8.1.7), SufS subfamily
RNA Metabolism	RNA processing and modification	tRNA modification Bacteria	FIG004453: protein YceG like
RNA Metabolism	RNA processing and modification	tRNA modification Bacteria	Ferredoxin, 2Fe-2S
RNA Metabolism	RNA processing and modification	tRNA modification Bacteria	GTP cyclohydrolase I (EC 3.5.4.16) type 1
RNA Metabolism	RNA processing and modification	tRNA modification Bacteria	GTPase and tRNA-U34 5-formylation enzyme TrmE
RNA Metabolism	RNA processing and modification	tRNA modification Bacteria	Iron-sulfur cluster assembly ATPase protein SufC
RNA Metabolism	RNA processing and modification	tRNA modification Bacteria	Iron-sulfur cluster assembly protein SufB
RNA Metabolism	RNA processing and modification	tRNA modification Bacteria	Iron-sulfur cluster assembly protein SufD
RNA Metabolism	RNA processing and modification	tRNA modification Bacteria	LSU m3Psi1915 methyltransferase RlmH
RNA Metabolism	RNA processing and modification	tRNA modification Bacteria	Queuosine Biosynthesis QueC ATPase
RNA Metabolism	RNA processing and modification	tRNA modification Bacteria	Queuosine Biosynthesis QueE Radical SAM
RNA Metabolism	RNA processing and modification	tRNA modification Bacteria	Queuosine biosynthesis QueD, PTPS-I
RNA Metabolism	RNA processing and modification	tRNA modification Bacteria	RNA binding methyltransferase FtsJ like
RNA Metabolism	RNA processing and modification	tRNA modification Bacteria	Rhodanese-related sulfurtransferases
RNA Metabolism	RNA processing and modification	tRNA modification Bacteria	Ribosomal large subunit pseudouridine synthase A (EC 4.2.1.70)
RNA Metabolism	RNA processing and modification	tRNA modification Bacteria	S-adenosylmethionine:tRNA ribosyltransferase-isomerase (EC 5.-.-.-)
RNA Metabolism	RNA processing and modification	tRNA modification Bacteria	glutamyl-Q-tRNA synthetase
RNA Metabolism	RNA processing and modification	tRNA modification Bacteria	tRNA (Guanine37-N1) -methyltransferase (EC 2.1.1.31)
RNA Metabolism	RNA processing and modification	tRNA modification Bacteria	tRNA (guanine46-N7-)-methyltransferase (EC 2.1.1.33)
RNA Metabolism	RNA processing and modification	tRNA modification Bacteria	tRNA 5-methylaminomethyl-2-thiouridine synthase TusA
RNA Metabolism	RNA processing and modification	tRNA modification Bacteria	tRNA dihydrouridine synthase B (EC 1.-.-.-)
RNA Metabolism	RNA processing and modification	tRNA modification Bacteria	tRNA dimethylallyltransferase (EC 2.5.1.75)
RNA Metabolism	RNA processing and modification	tRNA modification Bacteria	tRNA pseudouridine synthase A (EC 4.2.1.70)
RNA Metabolism	RNA processing and modification	tRNA modification Bacteria	tRNA pseudouridine synthase B (EC 4.2.1.70)
RNA Metabolism	RNA processing and modification	tRNA modification Bacteria	tRNA uridine 5-carboxymethylaminomethyl modification enzyme GidA
RNA Metabolism	RNA processing and modification	tRNA modification Bacteria	tRNA(Ile)-lysidine synthetase (EC 6.3.4.19)
RNA Metabolism	RNA processing and modification	tRNA modification Bacteria	tRNA-guanine transglycosylase (EC 2.4.2.29)
RNA Metabolism	RNA processing and modification	tRNA modification Bacteria	tRNA-i(6)A37 methylthiotransferase
RNA Metabolism	RNA processing and modification	tRNA modification Bacteria	tRNA-specific adenosine-34 deaminase (EC 3.5.4.-)
RNA Metabolism	RNA processing and modification	tRNA modification Bacteria	tRNA:Cm32/Um32 methyltransferase
RNA Metabolism	RNA processing and modification	tRNA nucleotidyltransferase	tRNA nucleotidyltransferase (EC 2.7.7.21) (EC 2.7.7.25)
RNA Metabolism	Transcription	RNA polymerase bacterial	DNA-directed RNA polymerase alpha subunit (EC 2.7.7.6)
RNA Metabolism	Transcription	RNA polymerase bacterial	DNA-directed RNA polymerase beta subunit (EC 2.7.7.6)
RNA Metabolism	Transcription	RNA polymerase bacterial	DNA-directed RNA polymerase beta' subunit (EC 2.7.7.6)
RNA Metabolism	Transcription	RNA polymerase bacterial	DNA-directed RNA polymerase omega subunit (EC 2.7.7.6)
RNA Metabolism	Transcription	Transcription factors bacterial	COG2740: Predicted nucleic-acid-binding protein implicated in transcription termination
RNA Metabolism	Transcription	Transcription factors bacterial	FIG000325: clustered with transcription termination protein NusA

RNA Metabolism	Transcription	Transcription factors bacterial	Transcription antitermination protein NusG
RNA Metabolism	Transcription	Transcription factors bacterial	Transcription elongation factor GreA
RNA Metabolism	Transcription	Transcription factors bacterial	Transcription termination factor Rho
RNA Metabolism	Transcription	Transcription factors bacterial	Transcription termination protein NusA
RNA Metabolism	Transcription	Transcription factors bacterial	Transcription termination protein NusB
RNA Metabolism	Transcription	Transcription factors bacterial	Transcription-repair coupling factor
RNA Metabolism	Transcription	Transcription factors bacterial	ribosomal protein L7Ae family protein
RNA Metabolism	Transcription	Transcription initiation, bacterial sigma factors	RNA polymerase sigma factor RpoD
RNA Metabolism	Transcription	Transcription initiation, bacterial sigma factors	RNA polymerase sigma factor RpoE
RNA Metabolism	Transcription	Transcription initiation, bacterial sigma factors	RNA polymerase sigma factor RpoH
RNA Metabolism	Transcription	Transcription initiation, bacterial sigma factors	RNA polymerase sigma factor RpoH-related protein
RNA Metabolism	Transcription	Transcription initiation, bacterial sigma factors	RNA polymerase sigma-54 factor RpoN
RNA Metabolism	Transcription	Transcription initiation, bacterial sigma factors	Serine protease precursor MucD/AlgY associated with sigma factor RpoE
Regulation and Cell signaling	Programmed Cell Death and Toxin-antitoxin Systems	Murein hydrolase regulation and cell death	Antiholin-like protein LrgA
Regulation and Cell signaling	Programmed Cell Death and Toxin-antitoxin Systems	Murein hydrolase regulation and cell death	LrgA-associated membrane protein LrgB
Regulation and Cell signaling	Programmed Cell Death and Toxin-antitoxin Systems	Murein hydrolase regulation and cell death	Preprotein translocase subunit SecG (TC 3.A.5.1.1)
Regulation and Cell signaling	no subcategory	Global Two-component Regulator PrrBA in Proteobacteria	Dna binding response regulator PrrA (RegA)
Regulation and Cell signaling	no subcategory	Global Two-component Regulator PrrBA in Proteobacteria	Sensor histidine kinase PrrB (RegB) (EC 2.7.3.-)
Regulation and Cell signaling	no subcategory	HPr catabolite repression system	HPr kinase/phosphorylase (EC 2.7.1.-) (EC 2.7.4.-)
Regulation and Cell signaling	no subcategory	Stringent Response, (p)ppGpp metabolism	Guanosine-3',5'-bis(diphosphate) 3'-pyrophosphohydrolase (EC 3.1.7.2)
Regulation and Cell signaling	no subcategory	The Chv regulatory system of Alphaproteobacteria	DNA-binding response regulator ChvI
Regulation and Cell signaling	no subcategory	The Chv regulatory system of Alphaproteobacteria	HPr kinase/phosphorylase (EC 2.7.1.-) (EC 2.7.4.-)
Regulation and Cell signaling	no subcategory	The Chv regulatory system of Alphaproteobacteria	PTS system permease (IIAMan), nitrogen regulatory IIA protein
Regulation and Cell signaling	no subcategory	The Chv regulatory system of Alphaproteobacteria	Phosphocarrier protein, nitrogen regulation associated
Regulation and Cell signaling	no subcategory	The Chv regulatory system of Alphaproteobacteria	Sensor histidine kinase ChvG (EC 2.7.3.-)
Regulons	Atomic Regulons	ar-104-EC Molybdenum cofactor biosynthesis moaABCDE	Molybdenum cofactor biosynthesis protein MoaA
Regulons	Atomic Regulons	ar-104-EC Molybdenum cofactor biosynthesis moaABCDE	Molybdenum cofactor biosynthesis protein MoaB
Regulons	Atomic Regulons	ar-104-EC Molybdenum cofactor biosynthesis moaABCDE	Molybdenum cofactor biosynthesis protein MoaC
Respiration	Electron accepting reactions	Anaerobic respiratory reductases	Arsenate reductase (EC 1.20.4.1)
Respiration	Electron accepting reactions	Anaerobic respiratory reductases	Butyryl-CoA dehydrogenase (EC 1.3.8.1)
Respiration	Electron accepting reactions	Anaerobic respiratory reductases	Electron transfer flavoprotein-ubiquinone oxidoreductase (EC 1.5.5.1)
Respiration	Electron accepting reactions	Anaerobic respiratory reductases	Ferredoxin reductase
Respiration	Electron accepting reactions	Anaerobic respiratory reductases	Flavodoxin reductases (ferredoxin-NADPH reductases) family 1
Respiration	Electron accepting reactions	Terminal cytochrome C oxidases	Cytochrome c oxidase polypeptide I (EC 1.9.3.1)
Respiration	Electron accepting reactions	Terminal cytochrome C oxidases	Cytochrome c oxidase polypeptide II (EC 1.9.3.1)
Respiration	Electron accepting reactions	Terminal cytochrome C oxidases	Cytochrome c oxidase polypeptide III (EC 1.9.3.1)
Respiration	Electron accepting reactions	Terminal cytochrome C oxidases	Cytochrome c oxidase subunit CcoN (EC 1.9.3.1)
Respiration	Electron accepting reactions	Terminal cytochrome C oxidases	Cytochrome c oxidase subunit CcoO (EC 1.9.3.1)
Respiration	Electron accepting reactions	Terminal cytochrome C oxidases	Cytochrome c oxidase subunit CcoP (EC 1.9.3.1)
Respiration	Electron accepting reactions	Terminal cytochrome C oxidases	Type cbb3 cytochrome oxidase biogenesis protein CcoG, involved in Cu oxidation
Respiration	Electron accepting reactions	Terminal cytochrome C oxidases	Type cbb3 cytochrome oxidase biogenesis protein CcoS, involved in heme b insertion
Respiration	Electron accepting reactions	Terminal cytochrome C oxidases	membrane c-type cytochrome cy
Respiration	Electron donating reactions	NADH ubiquinone oxidoreductase	NADH ubiquinone oxidoreductase chain A (EC 1.6.5.3)
Respiration	Electron donating reactions	NADH ubiquinone oxidoreductase	NADH-ubiquinone oxidoreductase chain B (EC 1.6.5.3)
Respiration	Electron donating reactions	NADH ubiquinone oxidoreductase	NADH-ubiquinone oxidoreductase chain C (EC 1.6.5.3)
Respiration	Electron donating reactions	NADH ubiquinone oxidoreductase	NADH-ubiquinone oxidoreductase chain D (EC 1.6.5.3)
Respiration	Electron donating reactions	NADH ubiquinone oxidoreductase	NADH-ubiquinone oxidoreductase chain E (EC 1.6.5.3)
Respiration	Electron donating reactions	NADH ubiquinone oxidoreductase	NADH-ubiquinone oxidoreductase chain F (EC 1.6.5.3)
Respiration	Electron donating reactions	NADH ubiquinone oxidoreductase	NADH-ubiquinone oxidoreductase chain G (EC 1.6.5.3)
Respiration	Electron donating reactions	NADH ubiquinone oxidoreductase	NADH-ubiquinone oxidoreductase chain H (EC 1.6.5.3)
Respiration	Electron donating reactions	NADH ubiquinone oxidoreductase	NADH-ubiquinone oxidoreductase chain I (EC 1.6.5.3)
Respiration	Electron donating reactions	NADH ubiquinone oxidoreductase	NADH-ubiquinone oxidoreductase chain J (EC 1.6.5.3)
Respiration	Electron donating reactions	NADH ubiquinone oxidoreductase	NADH-ubiquinone oxidoreductase chain K (EC 1.6.5.3)
Respiration	Electron donating reactions	NADH ubiquinone oxidoreductase	NADH-ubiquinone oxidoreductase chain L (EC 1.6.5.3)
Respiration	Electron donating reactions	NADH ubiquinone oxidoreductase	NADH-ubiquinone oxidoreductase chain M (EC 1.6.5.3)
Respiration	Electron donating reactions	NADH ubiquinone oxidoreductase	NADH-ubiquinone oxidoreductase chain N (EC 1.6.5.3)
Respiration	Electron donating reactions	Respiratory Complex I	NADH ubiquinone oxidoreductase chain A (EC 1.6.5.3)
Respiration	Electron donating reactions	Respiratory Complex I	NADH-ubiquinone oxidoreductase chain B (EC 1.6.5.3)
Respiration	Electron donating reactions	Respiratory Complex I	NADH-ubiquinone oxidoreductase chain C (EC 1.6.5.3)
Respiration	Electron donating reactions	Respiratory Complex I	NADH-ubiquinone oxidoreductase chain D (EC 1.6.5.3)
Respiration	Electron donating reactions	Respiratory Complex I	NADH-ubiquinone oxidoreductase chain E (EC 1.6.5.3)
Respiration	Electron donating reactions	Respiratory Complex I	NADH-ubiquinone oxidoreductase chain F (EC 1.6.5.3)
Respiration	Electron donating reactions	Respiratory Complex I	NADH-ubiquinone oxidoreductase chain G (EC 1.6.5.3)
Respiration	Electron donating reactions	Respiratory Complex I	NADH-ubiquinone oxidoreductase chain H (EC 1.6.5.3)
Respiration	Electron donating reactions	Respiratory Complex I	NADH-ubiquinone oxidoreductase chain I (EC 1.6.5.3)
Respiration	Electron donating reactions	Respiratory Complex I	NADH-ubiquinone oxidoreductase chain J (EC 1.6.5.3)
Respiration	Electron donating reactions	Respiratory Complex I	NADH-ubiquinone oxidoreductase chain K (EC 1.6.5.3)
Respiration	Electron donating reactions	Respiratory Complex I	NADH-ubiquinone oxidoreductase chain L (EC 1.6.5.3)
Respiration	Electron donating reactions	Respiratory Complex I	NADH-ubiquinone oxidoreductase chain M (EC 1.6.5.3)
Respiration	Electron donating reactions	Respiratory Complex I	NADH-ubiquinone oxidoreductase chain N (EC 1.6.5.3)

Respiration	Electron donating reactions	Respiratory Complex I	NADH:ubiquinone oxidoreductase 17.2 kD subunit
Respiration	Electron donating reactions	Succinate dehydrogenase	Succinate dehydrogenase cytochrome b-556 subunit
Respiration	Electron donating reactions	Succinate dehydrogenase	Succinate dehydrogenase flavoprotein subunit (EC 1.3.99.1)
Respiration	Electron donating reactions	Succinate dehydrogenase	Succinate dehydrogenase hydrophobic membrane anchor protein
Respiration	Electron donating reactions	Succinate dehydrogenase	Succinate dehydrogenase iron-sulfur protein (EC 1.3.99.1)
Respiration	no subcategory	Biogenesis of c-type cytochromes	ABC transporter involved in cytochrome c biogenesis, ATPase component CcmA
Respiration	no subcategory	Biogenesis of c-type cytochromes	ABC transporter involved in cytochrome c biogenesis, CcmB subunit
Respiration	no subcategory	Biogenesis of c-type cytochromes	Cytochrome c heme lyase subunit CcmF
Respiration	no subcategory	Biogenesis of c-type cytochromes	Cytochrome c heme lyase subunit CcmH
Respiration	no subcategory	Biogenesis of c-type cytochromes	Cytochrome c heme lyase subunit CcmL
Respiration	no subcategory	Biogenesis of c-type cytochromes	Cytochrome c-type biogenesis protein CcdA (DsbD analog)
Respiration	no subcategory	Biogenesis of c-type cytochromes	Cytochrome c-type biogenesis protein CcmC, putative heme lyase for CcmE
Respiration	no subcategory	Biogenesis of c-type cytochromes	Cytochrome c-type biogenesis protein CcmE, heme chaperone
Respiration	no subcategory	Biogenesis of c-type cytochromes	Cytochrome c-type biogenesis protein CcmG/DsbE, thiol:disulfide oxidoreductase
Respiration	no subcategory	Biogenesis of c-type cytochromes	Periplasmic thiol:disulfide interchange protein DsbA
Respiration	no subcategory	Biogenesis of c-type cytochromes	Periplasmic thiol:disulfide oxidoreductase DsbB, required for DsbA reoxidation
Respiration	no subcategory	Biogenesis of c-type cytochromes	Thiol:disulfide oxidoreductase TlpA
Respiration	no subcategory	Biogenesis of cbb3-type cytochrome c oxidases	Type cbb3 cytochrome oxidase biogenesis protein CcoG, involved in Cu oxidation
Respiration	no subcategory	Biogenesis of cbb3-type cytochrome c oxidases	Type cbb3 cytochrome oxidase biogenesis protein CcoH
Respiration	no subcategory	Biogenesis of cbb3-type cytochrome c oxidases	Type cbb3 cytochrome oxidase biogenesis protein CcoI
Respiration	no subcategory	Biogenesis of cbb3-type cytochrome c oxidases	Type cbb3 cytochrome oxidase biogenesis protein CcoS, involved in heme b insertion
Respiration	no subcategory	Biogenesis of cytochrome c oxidases	Copper metallochaperone, bacterial analog of Cox17 protein
Respiration	no subcategory	Biogenesis of cytochrome c oxidases	Cytochrome oxidase biogenesis protein Cox11-CtaG, copper delivery to Cox1
Respiration	no subcategory	Biogenesis of cytochrome c oxidases	Cytochrome oxidase biogenesis protein Sco1/SenC/PrrC, putative copper metallochaperone
Respiration	no subcategory	Biogenesis of cytochrome c oxidases	Heme A synthase, cytochrome oxidase biogenesis protein Cox15-CtaA
Respiration	no subcategory	Biogenesis of cytochrome c oxidases	Heme O synthase, protoheme IX farnesyltransferase (EC 2.5.1.-) COX10-CtaB
Respiration	no subcategory	Quinone oxidoreductase family	Putative Zn-dependent oxidoreductase PA5234
Respiration	no subcategory	Quinone oxidoreductase family	Putative oxidoreductase YncB
Respiration	no subcategory	Quinone oxidoreductase family	Quinone oxidoreductase (EC 1.6.5.5)
Respiration	no subcategory	Soluble cytochromes and functionally related electron carriers	Ferredoxin, 2Fe-2S
Secondary Metabolism	Plant Hormones	Auxin biosynthesis	Anthranilate phosphoribosyltransferase (EC 2.4.2.18)
Secondary Metabolism	Plant Hormones	Auxin biosynthesis	Phosphoribosylanthranilate isomerase (EC 5.3.1.24)
Secondary Metabolism	Plant Hormones	Auxin biosynthesis	Tryptophan synthase alpha chain (EC 4.2.1.20)
Secondary Metabolism	Plant Hormones	Auxin biosynthesis	Tryptophan synthase beta chain (EC 4.2.1.20)
Stress Response	Cold shock	Cold shock, CspA family of proteins	Cold shock protein CspA
Stress Response	Detoxification	Glutathione-dependent pathway of formaldehyde detoxification	S-(hydroxymethyl)glutathione dehydrogenase (EC 1.1.1.284)
Stress Response	Heat shock	Heat shock dnaK gene cluster extended	Chaperone protein DnaJ
Stress Response	Heat shock	Heat shock dnaK gene cluster extended	Chaperone protein DnaK
Stress Response	Heat shock	Heat shock dnaK gene cluster extended	Glutathione synthetase (EC 6.3.2.3)
Stress Response	Heat shock	Heat shock dnaK gene cluster extended	Heat shock protein GrpE
Stress Response	Heat shock	Heat shock dnaK gene cluster extended	Heat-inducible transcription repressor HrcA
Stress Response	Heat shock	Heat shock dnaK gene cluster extended	Nucleoside 5-triphosphatase RdgB (dHAPTP, dITP, XTP-specific) (EC 3.6.1.15)
Stress Response	Heat shock	Heat shock dnaK gene cluster extended	RNA polymerase sigma factor RpoH
Stress Response	Heat shock	Heat shock dnaK gene cluster extended	Radical SAM family enzyme, similar to coproporphyrinogen III oxidase, oxygen-independent, clustered with nucleoside-triphosphatase RdgB
Stress Response	Heat shock	Heat shock dnaK gene cluster extended	Ribonuclease PH (EC 2.7.7.56)
Stress Response	Heat shock	Heat shock dnaK gene cluster extended	Ribosomal RNA small subunit methyltransferase E (EC 2.1.1.-)
Stress Response	Heat shock	Heat shock dnaK gene cluster extended	Ribosomal protein L11 methyltransferase (EC 2.1.1.-)
Stress Response	Heat shock	Heat shock dnaK gene cluster extended	Ribosome-associated heat shock protein implicated in the recycling of the 50S subunit (S4 paralog)
Stress Response	Heat shock	Heat shock dnaK gene cluster extended	Translation elongation factor LepA
Stress Response	Heat shock	Heat shock dnaK gene cluster extended	rRNA small subunit methyltransferase I
Stress Response	Heat shock	Heat shock dnaK gene cluster extended	tRNA-t(6)A37 methylthiotransferase
Stress Response	Heat shock	Heat shock dnaK gene cluster extended	tmRNA-binding protein SmpB
Stress Response	Osmotic stress	Osmoregulation	Outer membrane protein A precursor
Stress Response	Oxidative stress	Cluster containing Glutathione synthetase	Glutathione synthetase (EC 6.3.2.3)
Stress Response	Oxidative stress	Cluster containing Glutathione synthetase	Putative Holliday junction resolvase YggF
Stress Response	Oxidative stress	Cluster containing Glutathione synthetase	Ribosomal RNA small subunit methyltransferase E (EC 2.1.1.-)
Stress Response	Oxidative stress	Cluster containing Glutathione synthetase	UPF0301 protein YqgE
Stress Response	Oxidative stress	Glutaredoxins	Glutaredoxin 3 (Grx2)
Stress Response	Oxidative stress	Glutaredoxins	Periplasmic septal ring factor with murein hydrolase activity EnvC/YibP
Stress Response	Oxidative stress	Glutathione: Biosynthesis and gamma-glutamyl cycle	Gamma-glutamyltranspeptidase (EC 2.3.2.2)
Stress Response	Oxidative stress	Glutathione: Biosynthesis and gamma-glutamyl cycle	Glutamate--cysteine ligase (EC 6.3.2.2)
Stress Response	Oxidative stress	Glutathione: Biosynthesis and gamma-glutamyl cycle	Glutathione synthetase (EC 6.3.2.3)
Stress Response	Oxidative stress	Glutathione: Non-redox reactions	Glutathione S-transferase (EC 2.5.1.18)

Stress Response	Oxidative stress	Glutathione: Non-redox reactions	Glutathione S-transferase family protein
Stress Response	Oxidative stress	Glutathione: Non-redox reactions	Glutathione S-transferase, unnamed subgroup (EC 2.5.1.18)
Stress Response	Oxidative stress	Glutathione: Non-redox reactions	Glutathione S-transferase, zeta (EC 2.5.1.18)
Stress Response	Oxidative stress	Glutathione: Non-redox reactions	Hydroxyacylglutathione hydrolase (EC 3.1.2.6)
Stress Response	Oxidative stress	Glutathione: Non-redox reactions	Lactoylglutathione lyase (EC 4.4.1.5)
Stress Response	Oxidative stress	Glutathione: Non-redox reactions	SAM-dependent methyltransferase 2, in cluster with Hydroxyacylglutathione hydrolase (EC 3.1.2.6)
Stress Response	Oxidative stress	Glutathione: Non-redox reactions	Uncharacterized glutathione S-transferase-like protein
Stress Response	Oxidative stress	Glutathione: Redox cycle	Glutaredoxin 3 (Grx2)
Stress Response	Oxidative stress	Glutathione: Redox cycle	Glutathione reductase (EC 1.8.1.7)
Stress Response	Oxidative stress	Protection from Reactive Oxygen Species	Catalase (EC 1.11.1.6)
Stress Response	Oxidative stress	Protection from Reactive Oxygen Species	Peroxidase (EC 1.11.1.7)
Stress Response	Oxidative stress	Protection from Reactive Oxygen Species	Superoxide dismutase [Cu-Zn] precursor (EC 1.15.1.1)
Stress Response	Oxidative stress	Protection from Reactive Oxygen Species	Superoxide dismutase [Fe] (EC 1.15.1.1)
Stress Response	Oxidative stress	Rubrerythrin	Alkyl hydroperoxide reductase subunit C-like protein
Stress Response	Oxidative stress	Rubrerythrin	Fe-S oxidoreductase-like protein in Rubrerythrin cluster
Stress Response	Oxidative stress	Rubrerythrin	Rubrerythrin
Stress Response	Periplasmic Stress	Periplasmic Stress Response	HtrA protease/chaperone protein
Stress Response	Periplasmic Stress	Periplasmic Stress Response	Survival protein SurA precursor (Peptidyl-prolyl cis-trans isomerase SurA) (EC 5.2.1.8)
Stress Response	no subcategory	Hfl operon	GTP-binding protein HflX
Stress Response	no subcategory	Hfl operon	HflC protein
Stress Response	no subcategory	Hfl operon	HflK protein
Stress Response	no subcategory	Hfl operon	Putative inner membrane protein YjeT (clustered with HflC)
Stress Response	no subcategory	Hfl operon	RNA-binding protein Hfq
Sulfur Metabolism	Inorganic sulfur assimilation	Inorganic Sulfur Assimilation	3'(2'),5'-bisphosphate nucleotidase (EC 3.1.3.7)
Sulfur Metabolism	Inorganic sulfur assimilation	Inorganic Sulfur Assimilation	Adenylyl-sulfate reductase [thioredoxin] (EC 1.8.4.10)
Sulfur Metabolism	Inorganic sulfur assimilation	Inorganic Sulfur Assimilation	Adenylylsulfate kinase (EC 2.7.1.25)
Sulfur Metabolism	Inorganic sulfur assimilation	Inorganic Sulfur Assimilation	Conserved hypothetical protein probably involved in sulfate reduction
Sulfur Metabolism	Inorganic sulfur assimilation	Inorganic Sulfur Assimilation	Oxidoreductase probably involved in sulfite reduction
Sulfur Metabolism	Inorganic sulfur assimilation	Inorganic Sulfur Assimilation	Phosphoadenylyl-sulfate reductase [thioredoxin] (EC 1.8.4.8)
Sulfur Metabolism	Inorganic sulfur assimilation	Inorganic Sulfur Assimilation	Sulfate adenylyltransferase subunit 1 (EC 2.7.7.4)
Sulfur Metabolism	Inorganic sulfur assimilation	Inorganic Sulfur Assimilation	Sulfate adenylyltransferase subunit 2 (EC 2.7.7.4)
Sulfur Metabolism	Inorganic sulfur assimilation	Inorganic Sulfur Assimilation	Sulfite reductase [NADPH] hemoprotein beta-component (EC 1.8.1.2)
Sulfur Metabolism	no subcategory	Thioredoxin-disulfide reductase	Alkyl hydroperoxide reductase subunit C-like protein
Sulfur Metabolism	no subcategory	Thioredoxin-disulfide reductase	Thiol peroxidase, Bcp-type (EC 1.11.1.15)
Sulfur Metabolism	no subcategory	Thioredoxin-disulfide reductase	Thioredoxin reductase (EC 1.8.1.9)
Virulence, Disease and Defense	Bacteriocins, ribosomally synthesized antibacterial peptides	Colicin V and Bacteriocin Production Cluster	Acetyl-coenzyme A carboxyl transferase beta chain (EC 6.4.1.2)
Virulence, Disease and Defense	Bacteriocins, ribosomally synthesized antibacterial peptides	Colicin V and Bacteriocin Production Cluster	Amidophosphoribosyltransferase (EC 2.4.2.14)
Virulence, Disease and Defense	Bacteriocins, ribosomally synthesized antibacterial peptides	Colicin V and Bacteriocin Production Cluster	Colicin V production protein
Virulence, Disease and Defense	Bacteriocins, ribosomally synthesized antibacterial peptides	Colicin V and Bacteriocin Production Cluster	Dihydrofolate synthase (EC 6.3.2.12)
Virulence, Disease and Defense	Bacteriocins, ribosomally synthesized antibacterial peptides	Colicin V and Bacteriocin Production Cluster	Folylpolyglutamate synthase (EC 6.3.2.17)
Virulence, Disease and Defense	Bacteriocins, ribosomally synthesized antibacterial peptides	Colicin V and Bacteriocin Production Cluster	tRNA pseudouridine synthase A (EC 4.2.1.70)
Virulence, Disease and Defense	Invasion and intracellular resistance	Mycobacterium virulence operon involved in DNA transcription	DNA-directed RNA polymerase beta subunit (EC 2.7.7.6)
Virulence, Disease and Defense	Invasion and intracellular resistance	Mycobacterium virulence operon involved in DNA transcription	DNA-directed RNA polymerase beta' subunit (EC 2.7.7.6)
Virulence, Disease and Defense	Invasion and intracellular resistance	Mycobacterium virulence operon involved in protein synthesis (LSU ribosomal proteins)	LSU ribosomal protein L20p
Virulence, Disease and Defense	Invasion and intracellular resistance	Mycobacterium virulence operon involved in protein synthesis (LSU ribosomal proteins)	LSU ribosomal protein L35p
Virulence, Disease and Defense	Invasion and intracellular resistance	Mycobacterium virulence operon involved in protein synthesis (LSU ribosomal proteins)	Translation initiation factor 3
Virulence, Disease and Defense	Invasion and intracellular resistance	Mycobacterium virulence operon involved in protein synthesis (SSU ribosomal proteins)	SSU ribosomal protein S12p (S23e)
Virulence, Disease and Defense	Invasion and intracellular resistance	Mycobacterium virulence operon involved in protein synthesis (SSU ribosomal proteins)	SSU ribosomal protein S7p (S5e)
Virulence, Disease and Defense	Invasion and intracellular resistance	Mycobacterium virulence operon involved in protein synthesis (SSU ribosomal proteins)	Translation elongation factor G
Virulence, Disease and Defense	Invasion and intracellular resistance	Mycobacterium virulence operon involved in protein synthesis (SSU ribosomal proteins)	Translation elongation factor Tu
Virulence, Disease and Defense	Invasion and intracellular resistance	Mycobacterium virulence operon possibly involved in quinolinate biosynthesis	L-aspartate oxidase (EC 1.4.3.16)
Virulence, Disease and Defense	Invasion and intracellular resistance	Mycobacterium virulence operon possibly involved in quinolinate biosynthesis	Quinolinate phosphoribosyltransferase [decarboxylating] (EC 2.4.2.19)
Virulence, Disease and Defense	Invasion and intracellular resistance	Mycobacterium virulence operon possibly involved in quinolinate biosynthesis	Quinolinate synthetase (EC 2.5.1.72)
Virulence, Disease and Defense	Resistance to antibiotics and toxic compounds	Arsenic resistance	Arsenate reductase (EC 1.20.4.1)
Virulence, Disease and Defense	Resistance to antibiotics and toxic compounds	Beta-lactamase	Beta-lactamase class C and other penicillin binding proteins
Virulence, Disease and Defense	Resistance to antibiotics and toxic compounds	Beta-lactamase	Metal-dependent hydrolases of the beta-lactamase superfamily I
Virulence, Disease and Defense	Resistance to antibiotics and toxic compounds	Cobalt-zinc-cadmium resistance	Cation efflux system protein CusA

Virulence, Disease and Defense	Resistance to antibiotics and toxic compounds	Cobalt-zinc-cadmium resistance	Cobalt-zinc-cadmium resistance protein
Virulence, Disease and Defense	Resistance to antibiotics and toxic compounds	Cobalt-zinc-cadmium resistance	Cobalt-zinc-cadmium resistance protein CzcA
Virulence, Disease and Defense	Resistance to antibiotics and toxic compounds	Cobalt-zinc-cadmium resistance	Cobalt-zinc-cadmium resistance protein CzcD
Virulence, Disease and Defense	Resistance to antibiotics and toxic compounds	Cobalt-zinc-cadmium resistance	Probable Co/Zn/Cd efflux system membrane fusion protein
Virulence, Disease and Defense	Resistance to antibiotics and toxic compounds	Cobalt-zinc-cadmium resistance	Transcriptional regulator, MerR family
Virulence, Disease and Defense	Resistance to antibiotics and toxic compounds	Copper homeostasis	Copper resistance protein B
Virulence, Disease and Defense	Resistance to antibiotics and toxic compounds	Copper homeostasis	Copper tolerance protein
Virulence, Disease and Defense	Resistance to antibiotics and toxic compounds	Copper homeostasis	Copper-translocating P-type ATPase (EC 3.6.3.4)
Virulence, Disease and Defense	Resistance to antibiotics and toxic compounds	Copper homeostasis	Cu(I)-responsive transcriptional regulator
Virulence, Disease and Defense	Resistance to antibiotics and toxic compounds	Copper homeostasis	Cytochrome c heme lyase subunit CcmF
Virulence, Disease and Defense	Resistance to antibiotics and toxic compounds	Copper homeostasis	Cytochrome c heme lyase subunit CcmH
Virulence, Disease and Defense	Resistance to antibiotics and toxic compounds	Copper homeostasis	Multicopper oxidase
Virulence, Disease and Defense	Resistance to antibiotics and toxic compounds	Copper homeostasis: copper tolerance	Copper homeostasis protein CutE
Virulence, Disease and Defense	Resistance to antibiotics and toxic compounds	Copper homeostasis: copper tolerance	Magnesium and cobalt efflux protein CorC
Virulence, Disease and Defense	Resistance to antibiotics and toxic compounds	Copper homeostasis: copper tolerance	Periplasmic divalent cation tolerance protein CutA
Virulence, Disease and Defense	Resistance to antibiotics and toxic compounds	Mercuric reductase	Mercuric ion reductase (EC 1.16.1.1)
Virulence, Disease and Defense	Resistance to antibiotics and toxic compounds	Mercury resistance operon	Mercuric ion reductase (EC 1.16.1.1)
Virulence, Disease and Defense	Resistance to antibiotics and toxic compounds	Multidrug Resistance Efflux Pumps	Acriflavin resistance protein
Virulence, Disease and Defense	Resistance to antibiotics and toxic compounds	Multidrug Resistance Efflux Pumps	Membrane fusion protein of RND family multidrug efflux pump
Virulence, Disease and Defense	Resistance to antibiotics and toxic compounds	Multidrug Resistance Efflux Pumps	Type I secretion outer membrane protein, TolC precursor
Virulence, Disease and Defense	Resistance to antibiotics and toxic compounds	Resistance to fluoroquinolones	DNA gyrase subunit A (EC 5.99.1.3)
Virulence, Disease and Defense	Resistance to antibiotics and toxic compounds	Resistance to fluoroquinolones	DNA gyrase subunit B (EC 5.99.1.3)
Virulence, Disease and Defense	Resistance to antibiotics and toxic compounds	Resistance to fluoroquinolones	Topoisomerase IV subunit A (EC 5.99.1.-)
Virulence, Disease and Defense	Resistance to antibiotics and toxic compounds	Resistance to fluoroquinolones	Topoisomerase IV subunit B (EC 5.99.1.-)
